# Supplementary material for: Epigenetic regulation limits competence of pluripotent stem cell‐derived oocytes
Source: EMBO J. 2023 Oct 18;42(23):e113955. doi: 10.15252/embj.2023113955 (PMC10690455; doi:10.15252/embj.2023113955)
Supplement: Supplementary file 1 — Appendix [file EMBJ-42-e113955-s004.pdf]

Appendix

Epigenetic regulation limits competence of pluripotent stem cell-derived oocytes

Eishi Aizawa, Evgeniy A. Ozonov, Yumiko K. Kawamura, Charles-Etienne Dumeau, So Nagaoka, Tomoya S. Kitajima, Mitinori Saitou, Antoine H. F. M. Peters*, Anton Wutz*

*Correspondence: antoine.peters@fmi.ch (A.H.F.M.P.), awutz@ethz.ch (A.W.)

Table of Contents:

Appendix Figure S1 .... Page 2

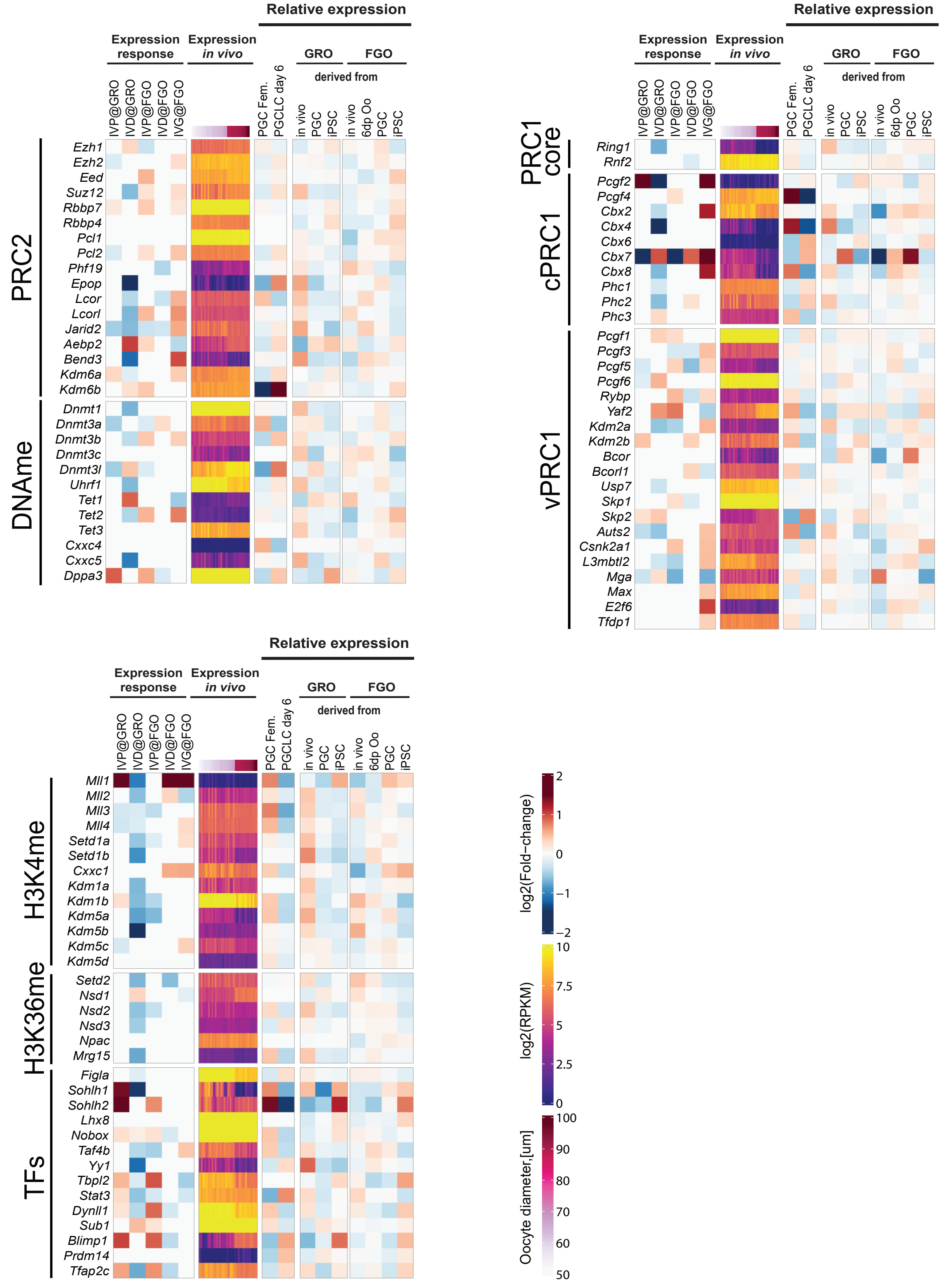

Appendix Figure S1. Expression of genes involved in epigenetic regulation and transcriptional reprogramming

Expression responses to each stage of *in vitro* development of genes involved in epigenetic regulation and transcriptional reprogramming (heatmap *Expression response*, all |*log*2(*Fold-change*)| with *FDR* ≥ 5% are considered non-significant and set to 0). In addition, expression in *in vivo* oocytes (heatmap Expression *in vivo*), and relative expression in PGC and PGCLC, GRO and FGO are displayed (group of heatmaps Relative expression, data for biological replicates or single oocytes belonging to particular cohorts were merged). Relative expression for each gene was calculated as difference between expression level (*log*2(RPKM)) and average expression calculated separately for PGC and PGCLC, GRO, and FGO.
