# Supplementary material for: Epigenetic regulation limits competence of pluripotent stem cell‐derived oocytes
Source: EMBO J. 2023 Oct 18;42(23):e113955. doi: 10.15252/embj.2023113955 (PMC10690455; doi:10.15252/embj.2023113955)
Supplement: Supplementary file 2 — Expanded View Figures PDF [file EMBJ-42-e113955-s012.pdf]

Expanded View Figures

Figure EV1. Development of PSC-derived oocytes during IVP and IVD, and assessment of FBS.

A Differentiation of BVSC-ESCs to PGCLCs. Expression of Blimp1-Venus and Stella-ECFP as well as bright-field images are shown from day 0 to 8 of the culture. Blimp1-Venus and Stella-ECFP started their expression at days 4 and 6, respectively. Scale bar, 100 μm.

B Representative development of a BVSC-ESC-derived rOvary from day 10 to 31 of the culture. Stella-ECFP expression transiently decreased around day 17, followed by emergence of round oocytes expressing Stella-ECFP. Scale bar, 100 μm.

C A representative morphology of BVSC-iPSC-derived follicles at day 31, and *in vivo*-derived follicles at 3, 6 and 9 dpp. Stella-ECFP expression (cyan) was merged with the bright-field image of BVSC-iPSC-derived follicles. Scale bar, 50 μm.

D Representative morphology of rOvaries at day 31 of the culture. Nine FBS and one serum replacement (a–j) were tested to assess follicle formation by the IVD culture. Bright-field images merged with Stella-ECFP (cyan) are shown. The commercial companies and catalog numbers of respective FBS and a serum replacement are as follows: a, Life Technologies, A3161001; b, Life Technologies, A3160801; c, GE Healthcare, SH30071.02; d, GE Healthcare, SV30160.02; e, Sigma, F0926; f, Life Technologies, A3160901; g, PAN Biotech, P30-1702; h, GE Healthcare, SH30084.02; i, Life Technologies, 10828-028; j, Equitech-Bio, SBSU30-0500. Scale bar, 200 μm.

E At day 31 of the culture, each rOvary was mechanically dissected by 30G needles to isolate single secondary follicles. The ratio of successful follicle isolation was calculated based on the number of isolated single secondary follicles divided by the number of secondary follicles attempted to isolate, which are shown in brackets. The data is based on two independent experiments.

Source data are available online for this figure.

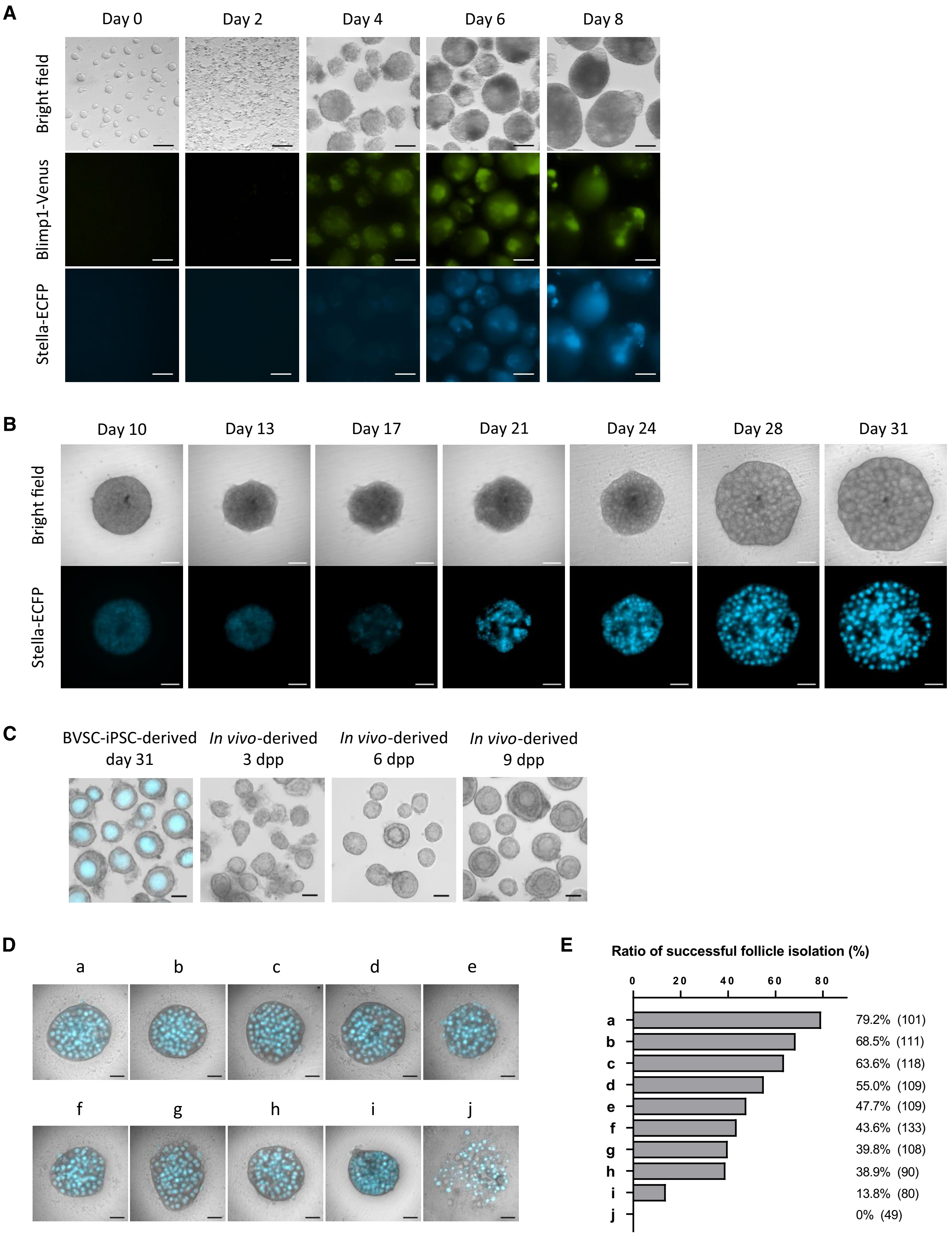

Figure EV1.

© 2023 The Authors

Figure EV2. Post-IVD development of PSC-derived oocytes and abnormal development of rOvaries/oocytes.

A IVG of BVSC-iPSC-derived follicles isolated from a rOvary. Bright-field images were merged with Stella-ECFP expression (cyan). Cultured follicles were treated with collagenase at day 33 of the culture. Some follicles expansively developed at day 43. Scale bar, 200 μm.

B Size of developed follicles at day 44 of the culture. The largest diameter of BVSC-iPSC-derived follicles in two conditions was measured; 1–3 follicles (left) and 4–10 follicles (right) placed on the same transwell membrane during IVG. Brackets represent number of counted follicles. The data of 4–10 follicles is identical to the data of iPSC-derived follicle in Fig 3A.

C MII oocytes containing first polar bodies derived from BVSC-ESCs at day 45 of the culture. Oocytes without Stella-ECFP expression were presumably derived from PGCs in E12.5 gonads contaminated at day 8 of the culture. Scale bar, 100 μm.

D Development of outgrowths from rOvaries. Bright-field images were merged with Stella-ECFP (left, cyan) and CAG-GFP (right, green). PGCLCs positive for both Blimp1-Venus and Stella-ECFP or for both SSEA1 and integrin-β3 were used for BVSC-iPSC-derived (left) or GFP-ESC-derived (right) rOvary respectively. Scale bar, 200 μm.

E Genotyping of outgrowths sampled from different six BVSC-iPSC-derived rOvaries. Arrowheads indicate amplified fragments targeting Blimp1-Venus, Stella-ECFP and endogenous Prdm14, respectively. All six outgrowths carried Blimp1-Venus and Stella-ECFP reporters, indicating the outgrowths were derived from BVSC-iPSCs.

F BVSC-iPSC-derived oocytes with cells on the inner side of zona pellucida, harvested at day 45 of the culture. The parts in white square (left) were enlarged to right images. While Stella-ECFP was detected in the ooplasm and polar body, the contaminating cells were negative for Stella-ECFP. Scale bar, 50 μm.

G A BVSC-iPSC-derived 2-cell embryo with cells on the inner side of zona pellucida, harvested 1 day after PA of the oocyte at day 45 of the culture. The part in a black square (left top) was enlarged to an image (right top). The asterisk indicates cells on the inner side of zona pellucida. Arrowheads indicate branched zona pellucida. Scale bar, 20 μm.

H Preimplantation development of BVSC-iPSC-derived oocytes after PA. Bright-field images were merged with Stella-ECFP expression (cyan). Embryos without Stella-ECFP expression presumably developed from E12.5 PGCs, which were possibly mixed with gonadal somatic cells for co-culture at day 8. Arrowheads indicate morulae. Scale bar, 50 μm.

Source data are available online for this figure.

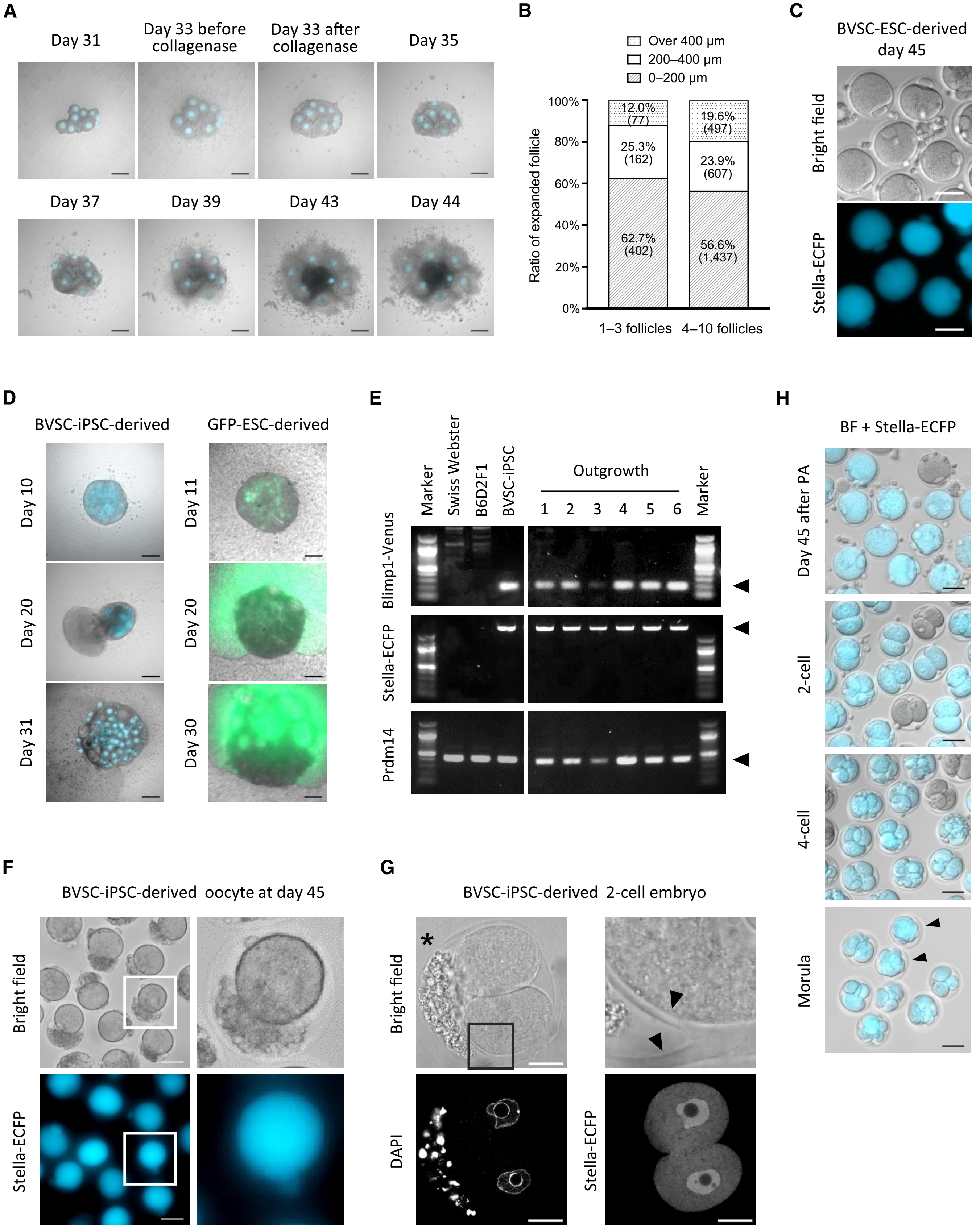

Figure EV2.

 © 2023 The Authors

Figure EV3. Immunostaining analysis of histone modification in 2-cell parthenotes and 5mC/5hmC in oocytes.

A Representative staining of H3K4me3 and H3K4ac in PA 2-cell embryos. Scale bar, 20 μm.
B Quantitative data of H3K4me3 and H3K4ac staining in PA 2-cell embryos. $N = 15$ (*in vivo*-derived) and 16 (iPSC-derived).
C Representative staining of H3K27me3 and H3K27ac in PA 2-cell embryos. Scale bar, 20 μm.
D Quantitative data of H3K27me3 and H3K27ac staining in PA 2-cell embryos. $N = 12$ (*in vivo*-derived) and 14 (iPSC-derived).
E Representative staining of 5mC and 5hmC in E12.5 gonad-derived oocytes after IVD (NSN oocytes) and IVG (SN oocytes). Scale bar, 10 μm.
F Quantitative data of 5mC and 5hmC in E12.5 gonad-derived oocytes after IVD (NSN oocytes) and IVG (SN oocytes). $N = 18$ (*in vivo*-derived, NSN), 18 (gonad-derived, NSN), 18 (*in vivo*-derived, SN) and 18 (gonad-derived, SN).

Data information: Bars represent mean ± SD. Statistical analysis was performed using an unpaired two-tailed *t*-test. *$P < 0.05$; **$P < 0.01$; ns, non-significant.
Source data are available online for this figure.

© 2023 The Authors

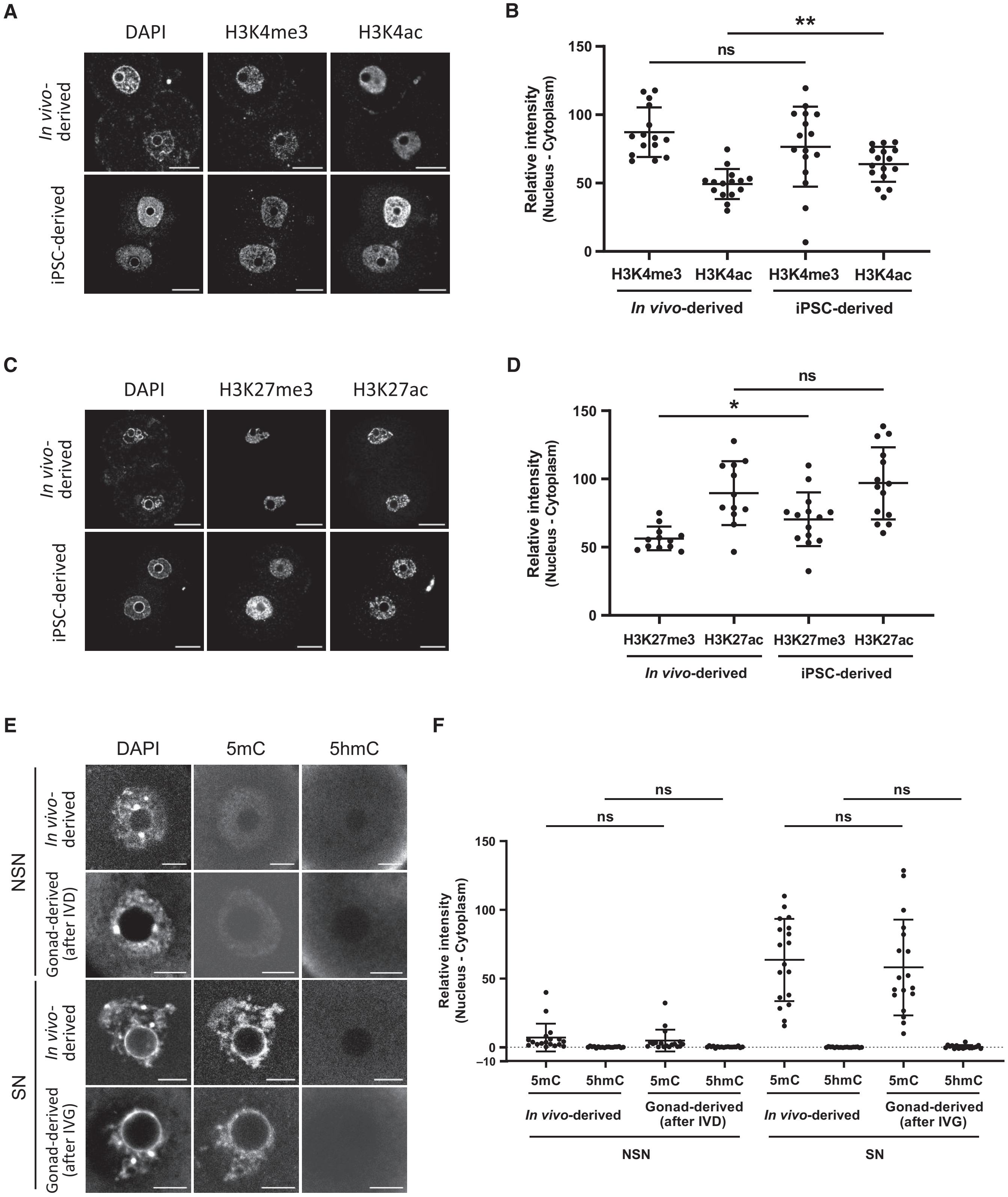

Figure EV3.

© 2023 The Authors

Figure EV4. Promoter features of affected genes and comparison of genes differentially expressed in PGCLCs compared to PGCs.

A Principal Component Analysis (PCA) of bulk RNA-Seq samples from this study for d6 PGCLC (red), PGC in E12.5 female (blue) and published RNA-Seq datasets for d6 PGCLC (pink; Ohta *et al*, 2017) and PGC in E12.5 female (light blue; Sasaki *et al*, 2015). PCA was performed on relative expression calculated for the dataset from this study and datasets from Ohta *et al* (2017) and Sasaki *et al* (2015) separately.

B Scatter plot showing expression of genes in E12.5 PGCs versus d6 PGCLCs and numbers of differentially expressed genes (with FDR $\leq$ 5% and $|\log2(\text{Fold-change})| \geq 2$).

C Scatter plots illustrating correlation between expression differences between d6 PGCLC and E12.5 female PGC profiled in this study and by authors (Sasaki *et al*, 2015) and (Ohta *et al*, 2017). Numbers of genes commonly up- and down-regulated genes in the two studies are marked in red and blue respectively (with FDR $\leq$ 5% and $|\log2(\text{Fold-change})| \geq 2$). Numbers of genes with conflicting results between two studies are marked in black.

D Gene ontology enrichment (GO) analysis for down-regulated genes in PGCLC compared to PGC (related to Dataset EV2). Bubbles representing GO terms are scaled according to enrichments, colored according to statistical significance and positioned relative to each other to reflect similarities between significantly affected genes with corresponding GO terms (see Materials and Methods).

E GO enrichment analysis for genes with statistically significant expression response to *in vitro* development (related to Dataset EV5).

F Results of χ^2 tests and enrichments of genes controlled by CpG island (CGI) promoters and non-CpG island promoters (non-CGI). Enrichments with χ^2 test *P*-value bigger than 1% are considered statistically not significant and displayed as dots.

G Scatter plot illustrating enrichments (*X*-axis, log2 scale) and statistical significance (*Y*-axis, −log10 (adjusted *P*-value)) of transcription factor motifs in promoters of genes which are up- or down-regulated by each stage of *in vitro* development and display particular expression dynamics from GRO to FGO *in vivo* (Fig 5C and E).

H Scatter plots showing expression differences between E12.5 PGCs and d6 PGCLCs versus expression responses to each stage of *in vitro* development.

Source data are available online for this figure.

© 2023 The Authors

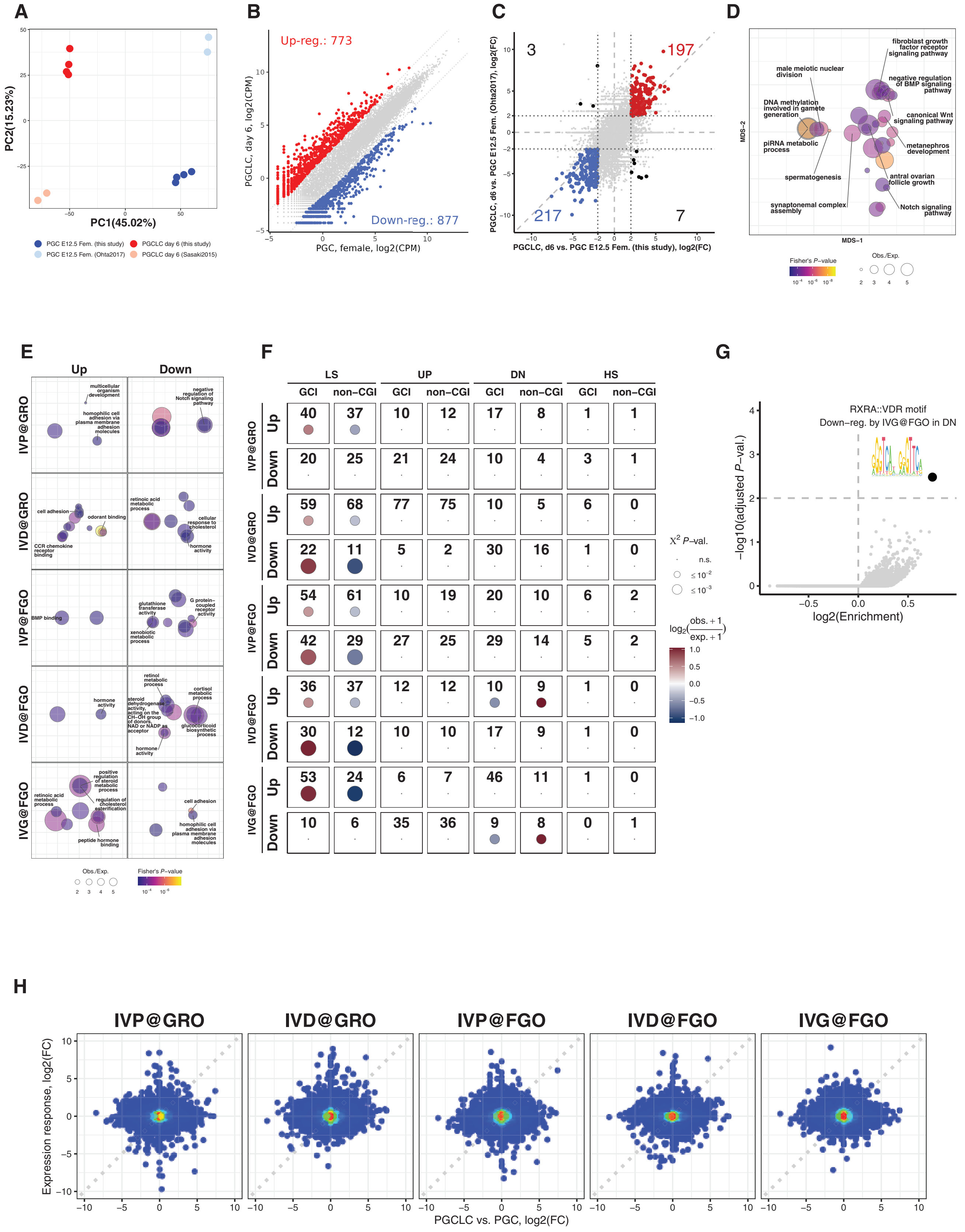

Figure EV4.

© 2023 The Authors

Figure EV5. Investigation of chromatin features of promoters of genes affected by *in vitro* PGCLC and oogenesis procedure.

A Boxplots illustrating differences in sizes of oocytes belonging to different cohorts of GRO (GRO^{iPSC}, GRO^{PGC}, $GRO^{in\ vivo}$) and FGO (FGO^{iPSC}, FGO^{PGC}, $FGO^{6dpp\ Oo}$ and $FGO^{in\ vivo}$). Numbers of oocytes in each cohort are displayed below each boxplot.

B Investigation of possible confounding effect of oocyte size differences on results of differential expression analysis in GRO. Each point represents relative expression of a gene (*Y*-axis) in a particular GRO with corresponding size in *X*-axis. Left panel shows relative expression of all genes showing up-regulation dynamics (UP group) in GROs of different cohorts (GRO^{iPSC} in red, GRO^{PGC} in green, $GRO^{in\ vivo}$ in blue), middle and rights panels represent relative expression of genes belonging to UP2 and UP3 clusters shown in Fig 6B respectively.

C Enrichments (scaled and centered to Z-scores) of H3K27me3 in non-growing oocytes and FGO (data from Hanna *et al*, 2018b), H2AK119Ub1 in growing oocytes at day 7 and FGO (data from Mei *et al*, 2021) and H3K4me3 in non-growing oocytes and FGO (data from Hanna *et al*, 2018b) at CGI and non-CGI promoters (±1.5 kb) of genes with specific expression dynamics *in vivo* (LS, UP, DN, HS groups, see Fig 5C) and affected genes in UP cluster from Fig 6B. Upper/lower whiskers extend to the largest/smallest values no further than 1.5*IQR from the upper/lower hinge, where IQR is interquartile range or distance between 25th and 75th percentiles. Outliers are not displayed. The notches extend $1.5 * \mathrm{IQR}/\sqrt{n}$ where *n* are numbers of CGI and non-CGI genes belonging to each group displayed below boxplots for H3K27me3 in NGO and the same for other stages and PTMs.

D Enrichments (scaled and centered to Z-scores) of H3K27me3 and H3K4me3 marks in PGC E13.5 (data from Kawabata *et al*, 2019) and d6 PGCLC (data from Kurimoto *et al*, 2015) at CGI and non-CGI promoters (±1.5 kb) of genes with specific expression dynamics *in vivo* (LS, UP, DN, HS groups, see Fig 5C) and affected genes in UP cluster from Fig 6B. Definitions of the central line, hinges, whiskers, notches as well numbers of genes in each group are the same as in Fig EV5C.

E Analogous to (C) but for affected genes in DN cluster from Fig 6B.

F Analogous to (D) but for affected genes in DN cluster from Fig 6B.

G Analogous to (C) but for affected genes in LS cluster from Fig 6B.

H Analogous to (D) but for affected genes in LS cluster from Fig 6B.

I Genomic snapshot of a representative gene *Psd2* belonging to the UP2 cluster (see Fig 6B) and distribution of several chromatin marks.

J Genomic snapshot of a representative gene *Lgi2* belonging to the UP3 cluster (see Fig 6B) and distribution of several chromatin marks.

Source data are available online for this figure.

© 2023 The Authors

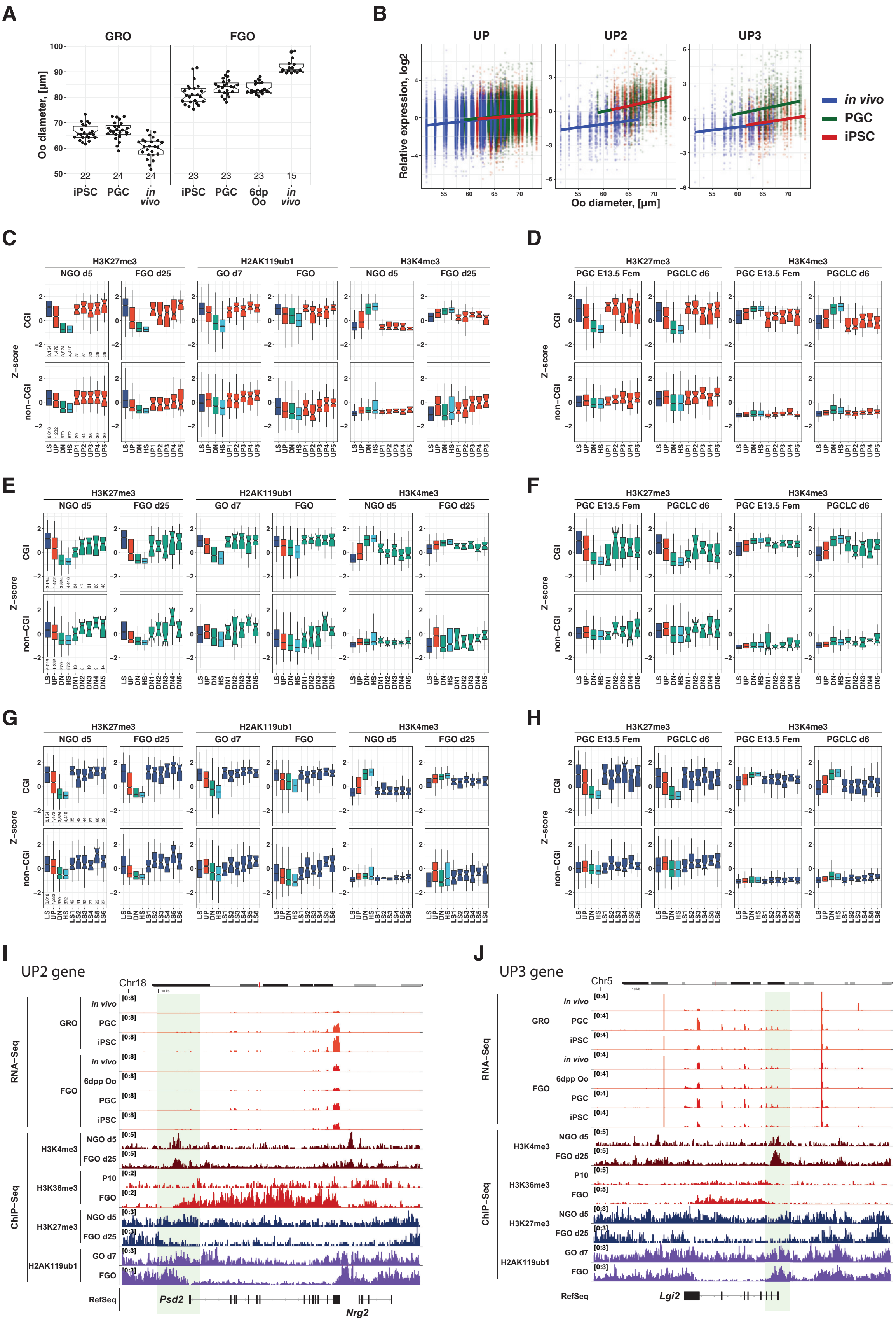

Figure EV5.

© 2023 The Authors
