# Supplementary material for: Epigenetic regulation limits competence of pluripotent stem cell‐derived oocytes
Source: EMBO J. 2023 Oct 18;42(23):e113955. doi: 10.15252/embj.2023113955 (PMC10690455; doi:10.15252/embj.2023113955)
Supplement: Supplementary file 13 — PDF+ [file EMBJ-42-e113955-s015.pdf]

Article

SOURCE DATA | TRANSPARENT PROCESS | OPEN ACCESS

THE EMBO JOURNAL

Epigenetic regulation limits competence of pluripotent stem cell-derived oocytes

Eishi Aizawa[1,2], Evgeniy A Ozonov[3], Yumiko K Kawamura[3], Charles-Etienne Dumeau[1], So Nagaoka[4], Tomoya S Kitajima[2], Mitinori Saitou[5,6,7], Antoine HFM Peters[3,8,*] & Anton Wutz[1,**]

Abstract

Recent studies have reported the differentiation of pluripotent cells into oocytes *in vitro*. However, the developmental competence of *in vitro*-generated oocytes remains low. Here, we perform a comprehensive comparison of mouse germ cell development *in vitro* over all culture steps versus *in vivo* with the goal to understand mechanisms underlying poor oocyte quality. We show that the *in vitro* differentiation of primordial germ cells to growing oocytes and subsequent follicle growth is critical for competence for preimplantation development. Systematic transcriptome analysis of single oocytes that were subjected to different culture steps identifies genes that are normally upregulated during oocyte growth to be susceptible for misregulation during *in vitro* oogenesis. Many misregulated genes are Polycomb targets. Deregulation of Polycomb repression is therefore a key cause and the earliest defect known in *in vitro* oocyte differentiation. Conversely, structurally normal *in vitro*-derived oocytes fail at zygotic genome activation and show abnormal acquisition of 5-hydroxymethylcytosine on maternal chromosomes. Our data identify epigenetic regulation at an early stage of oogenesis limiting developmental competence and suggest opportunities for future improvements.

Keywords epigenetics; germline; *in vitro* culture; oocyte; pluripotent stem cell
Subject Categories Chromatin, Transcription & Genomics; Development; Stem Cells & Regenerative Medicine
DOI 10.15252/embj.2023113955 | Received 7 March 2023 | Revised 18 September 2023 | Accepted 18 September 2023 | Published online 18 October 2023
The EMBO Journal (2023) 42: e113955

Introduction

In animals with sexual reproduction, germ cells are the source of totipotent cells, from which new individuals can develop. Although oocytes and spermatozoa transmit their genomes and epigenetic information to the offspring, the oocyte also provides cytoplasmic components that are crucial for the development of the embryo after fertilization. Mutations in germ cells are inherited by the offspring and drive genetic variation in species and can cause embryonic lethality or disorders (Ellegren & Galtier, 2016). How gametes develop to facilitate a totipotent configuration after fertilization remains to be elucidated. In mammals, studying the female germline is challenging as only a small number of germ cells develop to mature oocytes. In addition, tracing germ cell development in the embryo is difficult. For overcoming experimental limitations, *in vitro* culture systems for developing oocytes have been considered for over half a century (Odor & Blandau, 1971). At the beginning of the 2000s, several studies reported the generation of germ cells and mature gametes from pluripotent stem cells (PSCs) including embryonic stem cells (ESCs), induced pluripotent stem cells (iPSCs), epiblast stem cells and embryonic germ cells (Toyooka *et al*, 2003; Nayernia *et al*, 2006; Qing *et al*, 2007; Eguizabal *et al*, 2009; Ohinata *et al*, 2009). Especially, two studies succeeded in generating primordial germ cell-like cells (PGCLCs), which gave rise to functional spermatozoa and oocytes, from mouse ESCs and iPSCs by 2-step culture using a cocktail of growth factors (Hayashi *et al*, 2011, 2012). Subsequently, Hikabe *et al* (2016) reported the complete development of female germ cells in culture, thereby enabling the generation of mature metaphase II (MII) oocytes from mouse PSCs including ESCs and iPSCs. This important advance has been recently applied to studying mechanisms of female germ cell development including the dormant state in primordial follicles, effects of sex chromosomes and transcription factors (TFs) involved

1 Institute of Molecular Health Sciences, Swiss Federal Institute of Technology, ETH Zurich, Zurich, Switzerland
2 RIKEN Center for Biosystems Dynamics Research, Kobe, Japan
3 Friedrich Miescher Institute for Biomedical Research, Basel, Switzerland
4 Department of Embryology, Nara Medical University, Nara, Japan
5 Institute for the Advanced Study of Human Biology (ASHBi), Kyoto University, Kyoto, Japan
6 Department of Anatomy and Cell Biology, Graduate School of Medicine, Kyoto University, Kyoto, Japan
7 Center for iPS Cell Research and Application (CiRA), Kyoto University, Kyoto, Japan
8 Faculty of Sciences, University of Basel, Basel, Switzerland
*Corresponding author. Tel: +41 61 69 78757; E-mail: antoine.peters@fmi.ch
**Corresponding author. Tel: +41 44 63 30848; E-mail: awutz@ethz.ch

© 2023 The Authors. Published under the terms of the CC BY 4.0 license.

in oocyte growth (Nagamatsu *et al*, 2019; Shimamoto *et al*, 2019; Hamada *et al*, 2020; Hamazaki *et al*, 2021). It has also been used to study the kinetics and efficiency of X-chromosome inactivation and reactivation in female germ cells (Severino *et al*, 2022). However, oocytes developed *in vitro* have variable potential for embryogenesis. The success rate of full-term development from 2-cell embryos generated from *in vitro*-derived MII oocytes is substantially lower (0.9%, 26/2,753) than that of embryos generated using oocytes from superovulated mice (61.7%, 37/60; Hikabe *et al*, 2016).

During gametogenesis, germ cells undergo extensive epigenetic reprogramming. Following the specification of primordial germ cells (PGCs) and during their migration to gonads between embryonic day (E) 6.5 and E13.5, global CpG methylation levels rapidly decrease (Seisenberger *et al*, 2012). In parallel, global changes of histone modifications occur. In particular, reduced histone H3 lysine 9 dimethylation (H3K9me2) and elevated histone H3 lysine 27 trimethylation (H3K27me3) are associated with the PGC genome (Seki *et al*, 2005, 2007; Hajkova *et al*, 2008). Female PGCs enter meiosis around E13.5 and maintain their DNA largely devoid of methylation. Shortly after birth, primordial follicles emerge, which contain oocytes in meiotic arrest until ovulation (Smallwood *et al*, 2011; Shirane *et al*, 2013). The subsequent establishment of proper DNA methylation in the oocyte genome is important for controlling imprinted expression during embryogenesis (Kaneda *et al*, 2004). Allelic DNA methylation established at imprinting control regions in gametes regulates parental allele-specific expression of imprinted genes in embryos (Tucci *et al*, 2019). After primordial follicles exit the dormant state, *de novo* DNA methylation is established in growing oocytes (GROs) by the *de novo* DNA methyltransferases, DNMT3A and DNMT3L, in a dynamic interplay with opposing histone methylation pathways (Tucci *et al*, 2019; Stäubli & Peters, 2021). While histone H3 lysine 36 di- and tri-methylation (H3K36me2/me3) recruit DNMT3A/3L to chromatin, histone H3 lysine 4 di- and tri-methylation (H3K4me2/me3) inhibits DNMT3A/3L catalytic function in oocytes (Ooi *et al*, 2007; Ciccone *et al*, 2009; Zhang *et al*, 2010; Stewart *et al*, 2015). Polycomb group proteins also contribute to defining developmental competence by silencing differentiation-inducing genes and mediating spatial interactions between genome regions that are marked by H3K27me3 (Posfai *et al*, 2012; Du *et al*, 2020). Polycomb group proteins are observed in two major chromatin modifying Polycomb Repressive Complexes, PRC1 and PRC2, which catalyze mono-ubiquitination of histone H2A at lysine 119 (H2AK119ub1) and H3K27me3, respectively (Blackledge & Klose, 2021). Recently, marking of broad genomic regions with H3K27me3 in oocyte genomes was identified to regulate paternal X-chromosome inactivation as well as non-canonical imprinting, causing maternal allele-specific repression of dozens of genes in preimplantation embryos and extraembryonic placental tissues (Inoue *et al*, 2017a, 2017b; Chen *et al*, 2019; Hanna *et al*, 2019). In GROs, PRC1 functions upstream of PRC2 to define maternal H3K27me3-dependent imprints (Mei *et al*, 2021). Establishing the proper chromatin configuration during oocyte growth is, thus, a crucial factor for oocyte quality and developmental competence.

Here, we perform a detailed comparison between oocyte development *in vitro* and *in vivo* for identifying potential causes that impair the integrity of oocytes during culture. We first recapitulate oocyte development from PSCs *in vitro* with overall similar rates as previous studies (Hikabe *et al*, 2016). We then compare *in vitro* oocyte development from different developmental starting points of gametogenesis to define critical culture steps. Our data show that the differentiation from PGCs and PGCLCs to GROs and the subsequent growth of follicles are critical for specifying competence of fully grown oocytes (FGOs) for preimplantation development. Developmental failure of a large fraction of preimplantation embryos from *in vitro*-derived oocytes can be explained by failure of zygotic genome activation (ZGA) and abnormal acquisition of 5-hydroxymethylcytosine (5hmC), which further correlated with inactive pyruvate dehydrogenase (PDH) and the mislocalization of STELLA in the cytoplasm, respectively. Comprehensive transcriptome analysis of individual *in vitro* culture-derived versus *in vivo*-generated oocytes identified frequent transcriptional deregulation of genes that are normally repressed by Polycomb group proteins as new molecular factors that are misregulated in *in vitro*-generated oocytes. Our study emphasizes epigenetic regulation at an early step of oocyte differentiation as crucial for successful preimplantation development and identifies specific culture steps for attempts of improvement.

Results

In vitro culture facilitates full female germ cell development from PSCs

We used mouse ESC and iPSC lines that carried Blimp1-Venus and Stella-ECFP reporters (BVSC-ESC and BVSC-iPSC lines; Ohinata *et al*, 2008; Hayashi *et al*, 2012; Hikabe *et al*, 2016) as well as an ESC line with a ubiquitously expressed CAG-EGFP reporter (GFP-ESC line) to monitor *in vitro* development of PSCs to mature MII oocytes following a previous report (Hikabe *et al*, 2016). This protocol comprises four developmental steps over a span of 45 days. Starting from PSCs, we performed successive *in vitro* PGC differentiation (IVP), *in vitro* oocyte differentiation (IVD), *in vitro* growth (IVG) and *in vitro* maturation (IVM) of oocytes (Fig 1A).

During 8 days of IVP PSCs differentiate first into epiblast-like cells (EpiLCs) for 2 days and subsequently into PGCLCs. In BVSC-ESC and BVSC-iPSC lines, expression of Blimp1-Venus and Stella-ECFP was observed from days 4 and 6, respectively (Fig EV1A). Flow cytometry analysis showed that 1.1 and 5.1% of cells in day 8 embryoid bodies (EBs) generated from BVSC-ESCs and BVSC-iPSCs, respectively, were double-positive for both Blimp1-Venus and Stella-ECFP (Fig 1B). Immunostaining for SSEA1 and integrin β3, two surface markers for PGCLCs (Hayashi *et al*, 2011), revealed 4.3 and 6.0% double-positive cells in BVSC-ESC and GFP-ESC-derived EBs, further confirming the induction of PGC cell fate. Oogenesis requires interactive signals between oocytes and the surrounding gonadal somatic cells, which results in the formation of follicles (Frost *et al*, 2021; O'Connell & Pepling, 2021). IVD mimics *in vivo* development, by aggregating PGCLCs with somatic cells isolated from E12.5 female gonads in low-binding plates. We prepared such reconstituted ovaries (rOvaries) and cultured them on membranes of transwell plates for 21 days, resulting in the emergence of oocytes derived from BVSC-ESCs and BVSC-iPSCs at day 31 of the culture (Figs 1C and EV1B). Some oocytes were observed that lacked Stella-ECFP expression. These oocytes likely originated from incomplete depletion of germ cells from the gonadal somatic cells

 © 2023 The Authors

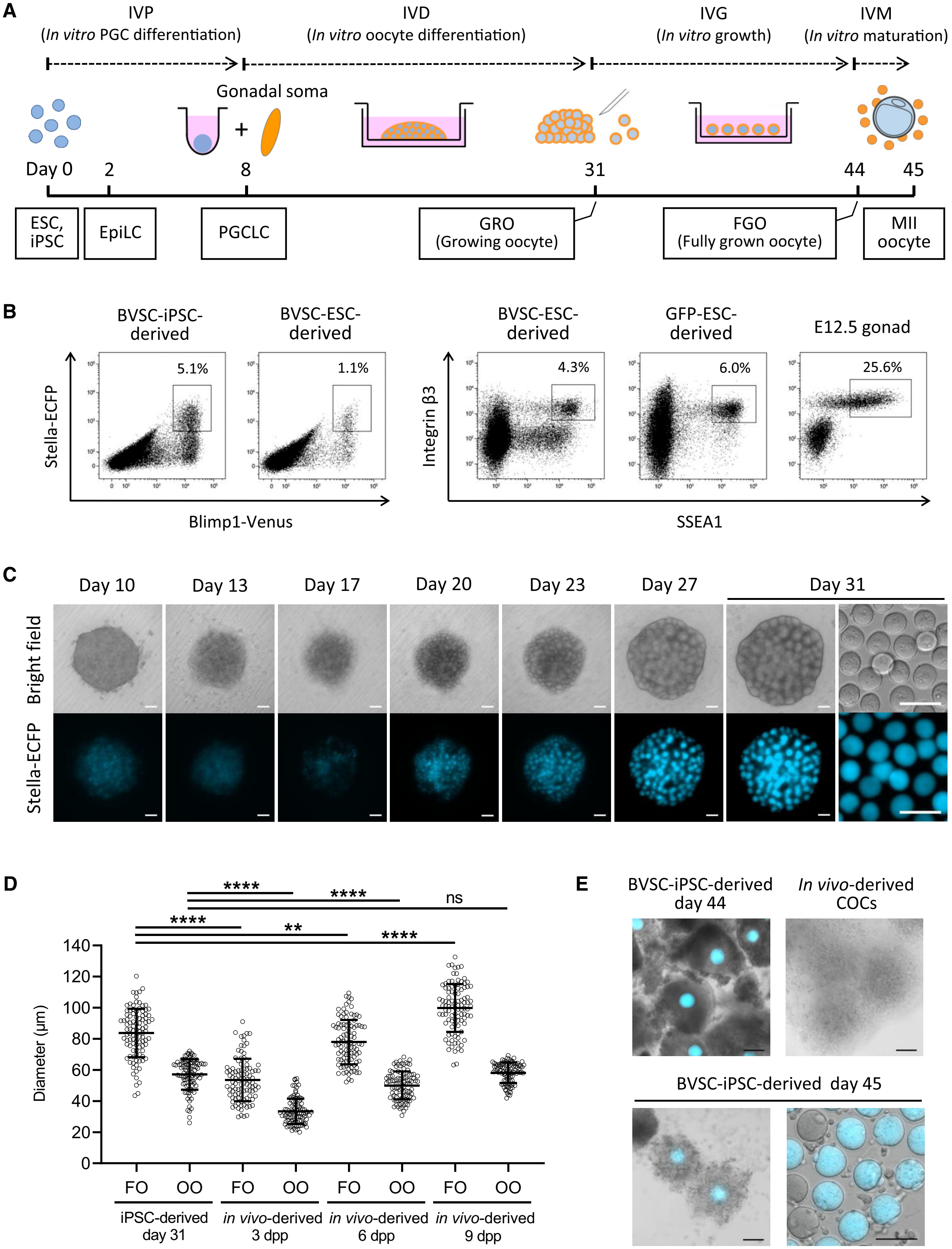
A
IVP
(In vitro PGC differentiation)
IVD
(In vitro oocyte differentiation)
IVG
(In vitro growth)
IVM
(In vitro maturation)
Gonadal soma
Day 0
2
8
31
44
45
ESC, iPSC
EpiLC
PGCLC
GRO (Growing oocyte)
FGO (Fully grown oocyte)
MII oocyte
B
BVSC-iPSC-derived
BVSC-ESC-derived
5.1%
1.1%
Stella-ECFP
Blimp1-Venus
BVSC-ESC-derived
GFP-ESC-derived
E12.5 gonad
4.3%
6.0%
25.6%
Integrin β3
SSEA1
C
Day 10
Day 13
Day 17
Day 20
Day 23
Day 27
Day 31
Bright field
Stella-ECFP
D
Diameter (µm)
****
****
ns
****
**
****
FO
OO
iPSC-derived day 31
in vivo-derived 3 dpp
in vivo-derived 6 dpp
in vivo-derived 9 dpp
E
BVSC-iPSC-derived day 44
In vivo-derived COCs
BVSC-iPSC-derived day 45

Figure 1.

Figure 1. Development of MII oocytes from iPSCs by *in vitro* culture.

A A schematic illustration of the *in vitro* culture system for the entire development of mouse female germ cells. Developmental stages (dashed line), the culture period (solid line) and developing cell types (square) are shown.

B A representative flow cytometry analysis of embryoid bodies derived from BVSC-ESC, BVSC-iPSC and GFP-ESC lines at day 8 of the culture. Female E12.5 gonads were analyzed as a control.

C Development of a BVSC-iPSC-derived rOvary from day 10 to 31 in culture. At day 31, oocytes were harvested from the rOvary (right). Scale bar, 100 μm.

D Oocyte and follicle diameters of iPSC-derived follicles at day 31, and *in vivo*-derived follicles at 3, 6 and 9 dpp. $N = 100$ oocytes or follicles in each condition. Bars represent mean ± SD. Statistical analysis was performed using an unpaired two-tailed *t*-test. FO, follicle; OO, oocyte. ****$P < 0.0001$; **$P < 0.01$; ns, non-significant.

E IVM of iPSC-derived follicles. Follicles at day 44 before IVM (left top), expanded follicles at day 45 after IVM (left bottom) and collected MII oocytes at day 45 (right bottom) are shown by merging bright-field images with Stella-ECFP expression (cyan). Bright-field image of *in vivo*-derived COCs collected after superovulation of a mouse is shown as control (right top). COCs, cumulus-oocyte complexes. Scale bar, 100 μm.

Source data are available online for this figure.

that we used to form rOvaries, as has been observed previously (Hikabe *et al*, 2016; Yoshino *et al*, 2021).

To estimate the developmental stage of oocytes and follicles, we compared the diameters of BVSC-iPSC-derived oocytes and follicles to *in vivo*-grown oocytes and follicles (Figs 1D and EV1C). These measurements showed that the size of iPSC-derived oocytes at day 31 (mean, 57.1 μm) was closest to those of 9 days postpartum (dpp) oocytes (mean, 58.2 μm). The size of iPSC-derived follicles (mean, 83.7 μm) was comparatively close to the size of 6 dpp follicles (mean, 78.0 μm). These results indicate that most iPSC-derived oocytes at day 31 correspond to GROs in primary and secondary follicles that are prevalent in prepubertal ovaries. The data further suggests a reduced rate of proliferation and/or growth of granulosa cells surrounding the GRO during IVD.

To enable further development of primary and secondary follicles to antral and preovulatory stages following the IVG protocol, we mechanically separated follicles in rOvaries at day 31 as described previously (Hikabe *et al*, 2016). Separation of some of the *in vitro*-derived follicles in rOvaries caused denudation of GROs from follicles indicating a fragile follicular structure. In contrast, denudation rarely occurred during dissection of follicles from 6 or 9 dpp ovaries. To overcome the problem of denudation, we tested nine batches of commercial fetal bovine serum (FBS) and a serum replacement for IVG culture (Fig EV1D) and performed isolation of single follicles from rOvaries. Our data show a strong influence of the serum on frequencies of denudation of GROs (Fig EV1E). We identified a commercial FBS (Life Technologies, A3161001), which enabled efficient isolation of intact follicles (79.2%) for successive experiments. We further evaluated dissection of rOvaries into clusters of either 1–3 or 4–10 follicles for IVG (Fig EV2A and B). At day 44 of IVG, each follicle was categorized into three groups according to its diameter (0–200; 200–400; over 400 μm) by measuring the longest part in a follicle under a stereomicroscope. The development of follicles was consistent with reports that showed diameters of some follicles reached over 400 μm after IVG (Hikabe *et al*, 2016; Morohaku *et al*, 2016, 2017). We observed a higher proportion of follicles with their diameters over 400 μm in experiments using larger groups of 4–10 follicles (19.6%) compared to 1–3 follicles (12.0%).

At day 44 of the culture, follicles with diameters greater than 200 μm were harvested and subjected to IVM. After IVM, oocytes and surrounding somatic cells formed expanded cumulus-oocyte complexes (COCs) with a similar morphology to *in vivo*-derived COCs (Fig 1E). Oocytes with first polar bodies, representing MII oocytes, were observed in COCs derived from BVSC-ESCs and BVSC-iPSCs at frequencies of 22.1% (52/235) and 12.7% (722/5,701), respectively (Table EV1, and Figs 1E and EV2C). Overall, we could recapitulate *in vitro* oogenesis with similar efficiency as previous reports.

Abnormalities of rOvaries and oocytes associated with *in vitro* development

In some rOvaries, we encountered abnormal development of oocytes and follicles during IVD (Table EV2 and Fig EV2D). Small cells protruded from rOvaries in about 16% of samples when we used PGCLCs expressing Blimp1-Venus and Stella-ECFP, and overgrew the culture. When GFP-ESC-derived PGCLCs were used for generating rOvaries the outgrowth of cell protrusion expressed GFP, indicating the outgrowth originated from GFP-ESCs (Fig EV2D). To confirm their origin, six outgrowths were sampled from different BVSC-iPSC-derived rOvaries. Genotyping revealed that all the outgrowths carried Blimp1-Venus and Stella-ECFP reporters, indicating these outgrowths were indeed derived from BVSC-iPSCs (Fig EV2E). Also, outgrowths were observed in all rOvaries when we used PGCLCs that were sorted from GFP-ESC and BVSC-ESC lines for SSEA1 and integrin β3 expression (Table EV2). This result suggests that sorting for SSEA1 and integrin β3 expression did not sufficiently enrich PGCLCs competent for follicle formation.

Following IVM, most of PSC-derived MII oocytes at day 45 of *in vitro* culture were almost indistinguishable from *in vivo*-grown MII oocytes (Figs 1E and EV2C). In 7% of PSC-derived oocytes (75/1,043) at day 45 we observed small cells on the inner side of the zona pellucida (Fig EV2F and G), and some oocytes had a split zona pellucida into two branches that resulted in embryos with contaminating cells (Fig EV2G). In contrast to the overgrowth of rOvaries, the contaminating cells within and abnormal morphology of the zona pellucida did not affect oocyte development.

IVD and IVG critically define embryonic competence of oocytes

We next assessed the competence of BVSC-iPSC-derived COCs for preimplantation development after IVM and fertilization with sperm (Fig 2A and B, and Table EV1). At 9 h from the start of *in vitro* fertilization (IVF), two pronuclei were observed in 70.3% (52/74) of iPSC-derived oocytes and in 73.2% (52/71) of *in vivo*-derived oocytes, indicating the similar competence of fertilization in these oocytes (Fig 2B). Zygotes from iPSC-derived oocytes progressed through cleavage divisions and developed into blastocysts in 1.7% of cases (14/838). We also parthenogenetically activated BVSC-

 © 2023 The Authors

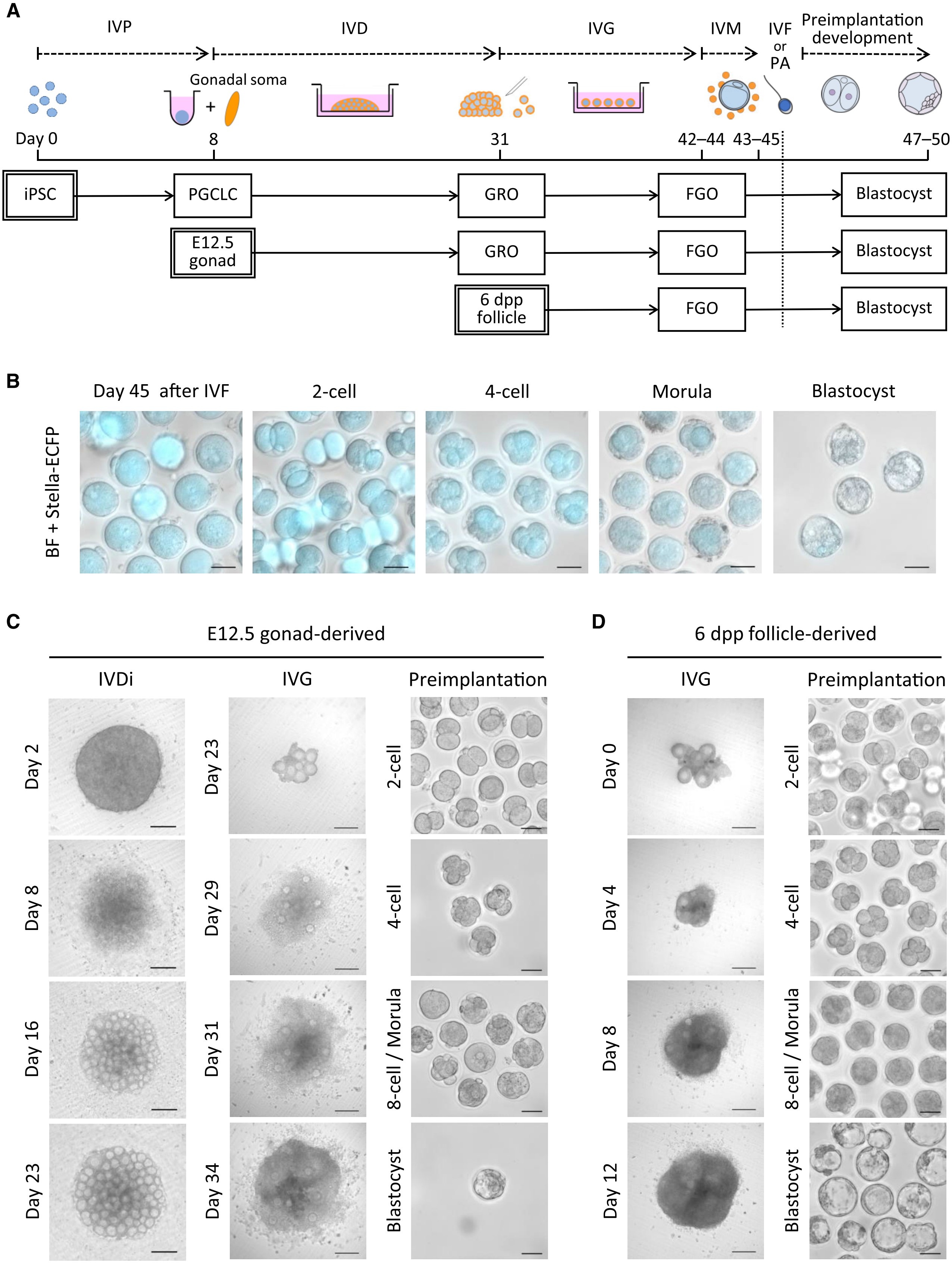
A
IVP
IVD
IVG
IVM
IVF
or
PA
Preimplantation
development
Gonadal soma
Day 0
8
31
42–44
43–45
47–50
iPSC
PGCLC
GRO
FGO
Blastocyst
E12.5
gonad
6 dpp
follicle
B
Day 45 after IVF
2-cell
4-cell
Morula
Blastocyst
BF + Stella-ECFP
C
E12.5 gonad-derived
IVDi
IVG
Preimplantation
Day 2
Day 8
Day 16
Day 23
Day 29
Day 31
Day 34
8-cell / Morula
D
6 dpp follicle-derived
Day 0
Day 4
Day 12

Figure 2.

Figure 2. Comparison of *in vitro* development of iPSC-, E12.5 gonad- and 6 dpp follicle-derived oocytes and embryos.

A A scheme to assess developmental stages of the *in vitro* culture. A similar culture protocol was applied to three different cell types (iPSCs, E12.5 gonads, and 6 dpp follicles), starting from each developmental stage (IVP, IVD and IVG). After IVM, oocytes derived from each cell type were subjected to IVF or parthenogenetic activation (PA), and preimplantation development was followed *in vitro*.

B Preimplantation development of BVSC-iPSC-derived oocytes after IVF. Stella-ECFP expression (cyan) was merged with bright-field images. Scale bar, 50 µm.

C Development of E12.5 gonad-derived rOvary, follicles and embryos after IVF. Scale bar, 200 µm (IVD and IVG) and 50 µm (preimplantation development).

D Development of 6 dpp follicles and 6 dpp follicle-derived embryos after IVF. Scale bar, 200 µm (IVG) and 50 µm (preimplantation development).

Source data are available online for this figure.

iPSC-derived oocytes at day 45 to measure their developmental competence without factors from spermatozoa (Fig EV2H and Table EV1). Using both methods, we found that iPSC-derived oocytes exhibited notably lower developmental rates at all stages of preimplantation development than control *in vivo*-derived oocytes.

To identify which *in vitro* culture steps are critical for defining oogenic and embryonic developmental competence we performed a comparative assessment of the different stages of *in vitro* culture versus *in vivo* development (Fig 2A). For this, we cultured E12.5 female gonads and 6 dpp follicles in accordance with corresponding *in vitro* culture protocols and compared their developmental competence to those of *in vivo* generated follicles. We chose 6 dpp follicles given that their overall size is comparable to that of PSC-derived follicles after the IVD, even though oocytes from 6 dpp follicles are smaller than those of day 31 PSC-derived oocytes (Fig 1D). This experimental design allowed us to relate developmental efficiency to gene expression profiles of individual oocytes, and to assess the impact of IVP, IVD and IVG on oocyte development by comparing *in vitro* culture and *in vivo*-grown germ cells of different developmental stages. E12.5 gonads were dissociated to form rOvaries followed by the IVD culture (Fig 2C). E12.5 gonad-derived follicles in rOvaries were dissected at day 23 and subsequently cultured following IVG and IVM. We also dissected follicles from 6 dpp ovaries and cultured them through the IVG and IVM steps (Fig 2D). After IVM, COCs derived from either E12.5 gonads or 6 dpp ovaries were subjected to IVF to assess their competence for preimplantation development.

Comparison of follicle expansion during the IVG demonstrated that about 20% of BVSC-iPSC-derived (19.6%) and E12.5 gonad-derived follicles (22.7%) reached a diameter of over 400 µm. In contrast, double the number of 6 dpp ovary-derived follicles reached a diameter of over 400 µm (48.7%; Fig 3A). We next monitored preimplantation development and observed development of blastocyst embryos at a rate of 1.7 and 0.9% for BVSC-iPSC-derived and E12.5 gonad-derived oocytes (Fig 3B and Table EV1). In contrast, 6 dpp follicle-derived oocytes were 8-fold more likely to develop blastocysts (15.0%). Furthermore, E12.5 gonad-derived and BVSC-iPSC-derived oocytes showed significant decreases in the transitions from oocyte to 2-cell embryo and from 2- to 4-cell embryo compared to 6 dpp follicle-derived oocytes (Fig 3C–E). These results have two implications. Firstly, rOvaries generated from BVSC-iPSC-derived PGCLCs and E12.5 gonad-derived PGCs possess comparatively similar developmental competences for IVG and preimplantation development. This indicates that PGCLC development *in vitro* is comparable to PGC development *in vivo*, at least for the current IVD settings. Secondly, a substantially reduced level of follicle expansion was observed for BVSC-iPSC-derived and E12.5 gonad-derived follicles compared to 6 dpp ovary-derived follicles during IVG. This result indicates that currently used IVD culture conditions negatively affect oocyte growth and limit embryonic competence. Given the relatively low blastocyst rate of even 6 dpp follicle-derived oocytes (15%; Fig 3B), we conclude that conditions for reconstitution and culture of oocyte–granulosa aggregates (IVD) as well as subsequent growth (IVG) require further optimization.

Impaired zygotic genome activation and epigenetic regulation is associated with *in vitro* culture of oocytes

The rate of preimplantation development to blastocysts varied widely from 55.6 to 0% among the six culture conditions (Fig 3B and Table EV1). Parthenogenetic activation (PA) and development of haploid embryos from BVSC-iPSC-derived oocytes was the only condition which did not result in blastocyst formation. Although 66.9% of BVSC-iPSC-derived haploid parthenotes developed from MII oocytes to 2-cell embryos (483/722; Table EV1), most 2-cell parthenotes notably failed to develop into 4-cell embryos (8.9%, 43/483). This result suggests that BVSC-iPSC-derived oocytes lack or misexpress factors which are critical for the maternal-to-zygotic transition and development beyond the 2-cell stage.

Figure 3. Developmental efficiency of IVG and preimplantation development of PSC-, E12.5 gonad- and 6 dpp follicle-derived oocytes.

A Summary of follicle development by IVG. The largest diameters of BVSC-iPSC-derived, E12.5 gonad-derived and 6 dpp follicle-derived follicles were measured at day 44, day 34 and day 12 of the culture, respectively. Numbers in brackets represent the number of counted follicles. The data of iPSC-derived follicles is identical to one of the data in Fig EV2B.

B Summary of preimplantation development expressed as a ratio with the number of counted oocytes in each sample set to 100%. Data are from 3 (*in vivo*-derived, IVF), 2 (6 dpp follicle-derived, IVF), 2 (E12.5 gonad-derived, IVF), 3 (BVSC-iPSC-derived, IVF), 2 (*in vivo*-derived, PA) and 18 (BVSC-iPSC-derived, PA) independent experiments. PA, parthenogenetic activation.

C–E Developmental ratio from all oocytes to 2-cell embryos (C), from 2-cell to 4-cell embryos (D), and from 4-cell to 8-cell embryos/morulae (E). $N = 3$ (*in vivo*-derived, IVF), 2 (6 dpp-follicle-derived, IVF), 2 (E12.5 gonad-derived, IVF), 3 (BVSC-iPSC-derived, IVF), 2 (*in vivo*-derived, PA) and 18 (BVSC-iPSC-derived, PA) independent experiments. Error bars represent SD. Statistical analysis was performed using an unpaired two-tailed *t*-test. *$P < 0.05$; **$P < 0.01$; ns, non-significant.

Source data are available online for this figure.

© 2023 The Authors

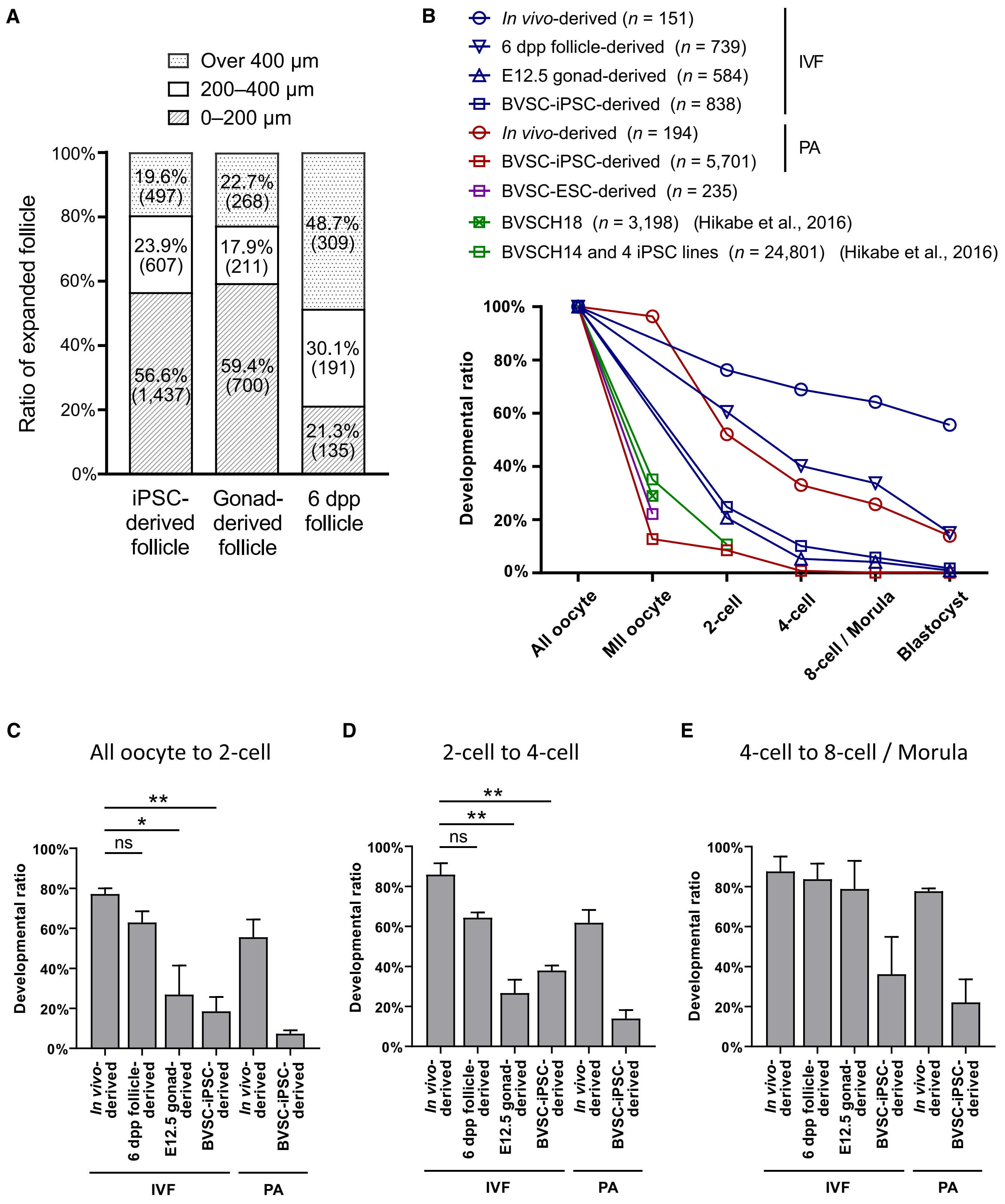
A
Over 400 μm
200–400 μm
0–200 μm
Ratio of expanded follicle
100%
80%
60%
40%
20%
0%
19.6% (497)
23.9% (607)
56.6% (1,437)
22.7% (268)
17.9% (211)
59.4% (700)
48.7% (309)
30.1% (191)
21.3% (135)
iPSC-derived follicle
Gonad-derived follicle
6 dpp follicle
B
In vivo-derived (n = 151)
6 dpp follicle-derived (n = 739)
E12.5 gonad-derived (n = 584)
BVSC-iPSC-derived (n = 838)
IVF
In vivo-derived (n = 194)
BVSC-iPSC-derived (n = 5,701)
PA
BVSC-ESC-derived (n = 235)
BVSCH18 (n = 3,198) (Hikabe et al., 2016)
BVSCH14 and 4 iPSC lines (n = 24,801) (Hikabe et al., 2016)
Developmental ratio
All oocyte
MII oocyte
2-cell
4-cell
8-cell / Morula
Blastocyst
C
All oocyte to 2-cell
D
2-cell to 4-cell
E
4-cell to 8-cell / Morula
**
*
ns
In vivo-derived
6 dpp follicle-derived
E12.5 gonad-derived
BVSC-iPSC-derived
In vivo-derived
BVSC-iPSC-derived
IVF
PA

Figure 3.

Early embryonic development is initially supported by maternal factors that accumulate in the egg during oogenesis and are progressively replaced by factors expressed in embryos (Zhang *et al*, 2022a). To investigate this point further, we performed immunostaining analysis for key factors of the maternal-to-zygotic transition (Figs 4 and EV3). To exclude effects from sperm, we studied parthenogenetically activated (PA) 2-cell embryos. We applied a Bromouridine-triphosphate (BrUTP) assay to quantify the extent of nascent transcription. Three-quarters of BVSC-iPSC-derived PA 2-cell embryos (18/24) lacked any BrUTP staining indicating that they failed to activate transcription of the embryonic genome (Fig 4A and B). Pyruvate dehydrogenase (PDH) has been reported to serve an important role in ZGA. It localizes in its active non-phosphorylated form in the nucleus of 2-cell embryos (Nagaraj *et al*, 2017). While all *in vivo* oocyte-derived 2-cell embryos showed nuclear enrichment of PDH, nuclear PDH levels were greatly reduced or even absent in BVSC-iPSC-derived 2-cell embryos (Fig 4C and D). Hence, such abnormal PDH localization may contribute to the observed ZGA failure.

We next asked if epigenetic marks of maternal chromosomes could provide hints for a potential cause of defects of culture-derived oocytes. Promoter occupancy of H3K4me3 and H3K27me3 have been associated with active and repressed genes, respectively, and both modifications are inherited on maternal chromatin from eggs to 2-cell embryos (Stäubli & Peters, 2021). Immunostaining analysis demonstrated a small increase of H3K27me3 levels in BVSC-iPSC-derived PA 2-cell embryos, while levels of H3K4me3 and H3K27 acetylation (H3K27ac), which marks active enhancers (Hanna *et al*, 2018a), were comparable to those in *in vivo*-derived controls (Fig EV3A–D). Furthermore, we observed increased levels of H3K4 acetylation (H3K4ac) in BVSC-iPSC-derived 2-cell embryos, which might negatively regulate the loading of the chromosome passenger complex during metaphase (Niedzialkowska *et al*, 2022). DNA methylation of the gametic genomes undergoes dramatic changes after fertilization. Although oocyte-derived 5-methylcytosine (5mC) undergoes passive demethylation over successive cell divisions in the early embryo, the paternal genome undergoes active DNA demethylation in zygotes, in part through TET-mediated conversion of 5mC to 5hmC (Nakamura *et al*, 2007, 2012; Amouroux *et al*, 2016). Immunostaining of 5mC and 5hmC in BVSC-iPSC-derived PA 2-cell embryos revealed an unexpected increase in 5hmC levels on maternal chromosomes, while 5mC levels were comparable to those in PA embryos derived from *in vivo*-grown oocytes (Fig 4E and F). To assess ectopic acquisition of 5hmC during oocyte development *in vitro*, we analyzed 5mC and 5hmC levels in E12.5 gonad-derived oocytes after IVD and IVG. Oocytes undergo chromatin remodeling from a non-surrounded nucleolus (NSN) to a surrounded nucleolus (SN) state during their growth (Zhang *et al*, 2022b). Most E12.5 gonad-derived oocytes after IVD and IVG exhibited NSN and SN states, respectively. Therefore, we selected only NSN oocytes after IVD and SN oocytes after IVG for analysis of 5mC and 5hmC (Fig EV3E and F). In both *in vivo*-derived and gonad-derived oocytes, 5mC levels were detected in NSN and SN oocytes, respectively, with no difference between culture conditions. In contrast, 5hmC levels were undetectable in both NSN and SN oocytes. These results indicate that 5hmC in iPSC-derived embryos was acquired after oocyte development. Binding of the STELLA protein to H3K9me2 on maternal chromosomes has been reported to prevent TET3-mediated oxidation of 5mC into 5hmC in fertilized eggs (Nakamura *et al*, 2012). Importantly, immunostaining analysis of H3K9me2 and STELLA revealed that some BVSC-iPSC-derived 2-cell embryos exhibited weak nuclear localization of STELLA (Fig 4G and H). Reduced localization of STELLA in the nucleus may underly enhanced acquisition of 5hmC after activation of *in vitro*-derived oocytes.

Genes normally up-regulated during oocyte growth are particularly vulnerable for misregulation during *in vitro* oogenesis

To identify possible causes underlying the overall low oogenic and embryonic competence of *in vitro*-derived oocytes, we performed extensive comparative transcriptome analysis between *in vivo* and *in vitro* grown germ cells, at different stages of their development. Firstly, we performed RNA-Seq analysis of BVSC-iPSC-derived PGCLCs at day 8 of *in vitro* culture (termed d6PGCLCs, representing PGCLCs 6 days after initiating IVP from EpiLCs) and PGCs isolated from E12.5 female gonads by fluorescence-activated cell sorting (FACS) for both SSEA1 and integrin β3 expression (Fig 1B). Principal component analysis revealed reproducible gene expression differences between d6PGCLCs and E12.5 PGCs which correlated well to previously published RNA-Seq datasets (Sasaki *et al*, 2015; Ohta *et al*, 2017; Fig EV4A–C). We identified 773 and 877 up- and down-regulated genes between d6PGCLCs and E12.5 PGCs respectively (Fig EV4B and Dataset EV1). Gene ontology analysis revealed that genes down-regulated in PGCLCs serve in various female and male germ cell development-related functions, in signaling pathways, in embryonic development and transcriptional processes (Fig EV4D

Figure 4. Immunostaining analysis of key factors for the maternal-to-zygotic transition.

A Representative staining of nascent transcripts after BrUTP incorporation in PA 2-cell embryos.
B Quantitative data of nascent transcripts after BrUTP incorporation in PA 2-cell embryos. $N = 16$ (*in vivo*-derived) and 24 (BVSC-iPSC-derived).
C Representative staining of pPDH and total PDH in PA 2-cell embryos.
D Quantitative data of pPDH and total PDH staining in PA 2-cell embryos. $N = 19$ (*in vivo*-derived) and 19 (BVSC-iPSC-derived).
E Representative staining of 5mC and 5hmC in PA 2-cell embryos. A fertilized zygote is shown as control.
F Quantitative data of 5mC and 5hmC staining in PA 2-cell embryos. $N = 21$ (*in vivo*-derived) and 18 (BVSC-iPSC-derived).
G Representative staining of H3K9me2 and STELLA in PA 2-cell embryos. Expression of Stella-ECFP is also shown in BVSC-iPSC-derived embryos.
H Quantitative data of H3K9me2 and STELLA staining in PA 2-cell embryos. $N = 18$ (*in vivo*-derived) and 24 (BVSC-iPSC-derived).

Data information: Bars represent mean ± SD. Statistical analysis was performed using an unpaired two-tailed *t*-test. Scale bar, 20 µm. $^{*}P < 0.05$; $^{****}P < 0.0001$; ns, non-significant.
Source data are available online for this figure.

© 2023 The Authors

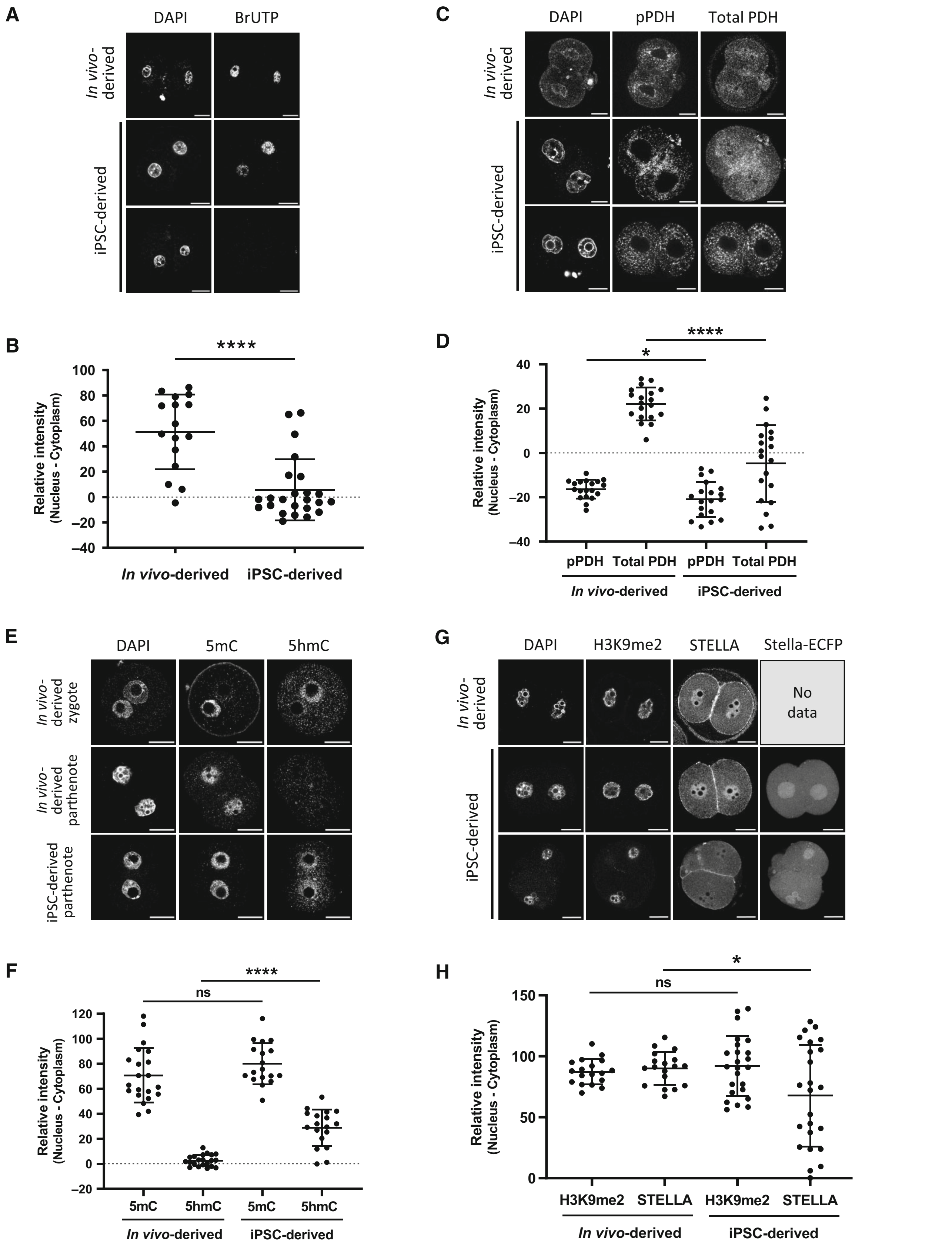
A
DAPI
BrUTP
In vivo-derived
iPSC-derived
B
****
Relative intensity (Nucleus - Cytoplasm)
In vivo-derived
iPSC-derived
C
DAPI
pPDH
Total PDH
In vivo-derived
iPSC-derived
D
****
*
Relative intensity (Nucleus - Cytoplasm)
pPDH
Total PDH
pPDH
Total PDH
In vivo-derived
iPSC-derived
E
DAPI
5mC
5hmC
In vivo-derived zygote
In vivo-derived parthenote
iPSC-derived parthenote
G
DAPI
H3K9me2
STELLA
Stella-ECFP
No data
In vivo-derived
iPSC-derived
F
****
ns
Relative intensity (Nucleus - Cytoplasm)
5mC
5hmC
5mC
5hmC
In vivo-derived
iPSC-derived
H
*
ns
Relative intensity (Nucleus - Cytoplasm)
H3K9me2
STELLA
H3K9me2
STELLA
In vivo-derived
iPSC-derived

Figure 4.

and Dataset EV2), hinting toward immature repression by Polycomb Repressive Complexes (PRCs) of such genes (Blackledge & Klose, 2021).

Secondly, we performed RNA-Seq and linear modeling analysis of single oocytes at the GRO and FGO stages to quantify and molecularly dissect the impact of different steps of IVP, IVD and IVG on gene expression. The single-cell nature of the experiment also allows to assess the transcriptional heterogeneity among and between *in vitro* and *in vivo*-grown oocytes. To do so, we directly isolated GROs and FGOs from mice or produced them *in vitro* using BVSC-iPSCs, E12.5 gonads, and 6 dpp follicles as sources of starting material (Fig 5A). Among the 162 separately sequenced GRO and FGO oocytes (Datasets EV3 and EV4), 27% of variance in expression could be assigned to differences between GROs and FGOs. Moreover, 8% of the variance directly relates to differences in *in vitro* culture conditions (Fig 5B). Expression differences between GROs derived from BVSC-iPSCs (GRO^{iPSC}) and from E12.5 embryonic gonads (GRO^{PGC}) can presumably be attributed to culture differences between *in vitro* PGCLCs versus *in vivo* PGC development, since both cohorts GRO^{iPSC} and GRO^{PGC} underwent the same IVD. We refer to such effect as "IVP@GRO" (Fig 5A). Similarly, expression differences between E12.5 gonad-derived GROs (GRO^{PGC}), having undergone IVD, versus *in vivo*-derived GROs ($GRO^{in\ vivo}$) can be attributed to the IVD stage (IVD@GRO in Fig 5A). To simplify our model, we assumed that expression differences between iPSC-derived GROs (GRO^{iPSC}) and *in vivo*-derived GROs ($GRO^{in\ vivo}$) are linear combinations of both effects of IVP and IVD (i.e. IVP@GRO + IVD@GRO). A similar approach was taken to quantify effects resulting from IVP, IVD or IVG and impacting expression in FGOs (@FGO). We utilized a generalized linear model to fit corresponding coefficients for each effect and performed likelihood ratio tests to find genes with statistically significant response to each step of *in vitro* development in GROs and FGOs (with FDR $\leq$ 5% and $|\log2(\text{Fold-change})| \geq 2$). Using these criteria, we identified around 100 to 300 genes up- or down-regulated in GROs or FGOs, in response to IVP, IVD or IVG (Fig 5C and Dataset EV4). Gene ontology analysis identified down-regulation of genes involved in Notch signaling and upregulation of genes involved in cell adhesion (e.g. *Cdh12*, *Cdh6*, *Cntn5*, *Dsc3*) resulting from IVP as measured in GROs (IVP@GRO; Fig EV4E and Dataset EV5). The impact of IVP on FGOs resulted in down-regulation of genes involved in Xenobiotic metabolic processes (e.g. *Cypb1b1*, *Cyp2c66*). IVD results in down-regulation of genes in retinoid metabolism in GROs and FGOs (e.g. *Adh1*, *Aldh1a1*) while genes with roles in cell adhesion, olfactory function and chemokine signaling (*Ccl1*, *Ccl4*, *Cx3cl1*, *Cxcl13*) are overrepresented among upregulated genes in IVD@GRO. IVG impacts on cell adhesion and retinoid metabolism as well, yet in an opposite manner as during IVD, as measured in FGOs (Dataset EV5).

While the cellular impact of altered expression of different genes remains to be explored, we next aimed to understand possible modes of regulation underlying altered gene expression during *in vitro* development in the context of transcriptional changes normally occurring during *in vivo* oogenesis. Therefore, we first clustered all genes (see Materials and Methods) into four groups according to their dynamics of expression during oocyte growth *in vivo* (Fig 5D). We named these groups LS for genes with low stable expression, UP (up-regulated from GRO to FGO), DN (down-regulated from GRO to FGO), and HS (high stable expression between GRO and FGO). We then investigated which genes in each group were differentially expressed by any of the steps of *in vitro* culture (Fig 5C and D). Among all differentially expressed genes, we observed a significant, 2.7-fold over-representation of genes which are normally up-regulated during the growth of GROs to the FGO stage *in vivo* (UP group; Fig 5E). In addition, we observed that genes with stable high expression both in GROs and FGOs (HS group) were significantly under-represented among affected genes.

To further investigate which gene expression dynamics *in vivo* characterize genes affected by each *in vitro* culture step we performed χ^2 tests for significant over-representation of genes with LS, UP, DN and HS dynamics among up- and down-regulated genes by the modeled effects. Interestingly, we observed that among genes normally up-regulated from GRO to FGO ("UP"), 45 were down-regulated in GROs by IVP, 152 were up-regulated in GROs by IVD, and 71 down-regulated in FGO following IVG (Fig 5F).

Next, we investigated promoter features, such as presence of CpG islands (CGIs) at promoters of genes affected by *in vitro* culture (Sendžikaitė & Kelsey, 2019). Interestingly, we did not observe significant enrichment of either CGI or non-CGI promoters among affected genes that normally display UP dynamics (Fig 5D and E). In contrast, most genes affected by each stage with LS dynamics were significantly enriched with CGI-driven promoters (Fig EV4F). We also performed enrichment analysis of transcription factor motifs

Figure 5. Modeling and quantification of effects of the *in vitro* development on gene expression.

A Experimental design and illustration of the effects of *in vitro* versus *in vivo* culture used as covariates in a Generalized Linear Model for gene expression. Expression for each gene at corresponding stage (GRO or FGO) is modeled as linear combination of effects of *in vitro* culture stages relative to *in vivo*-derived oocytes.

B PCA of single oocyte RNA-Seq data used to identify gene expression responses to stages of *in vitro* development. Each point corresponds to a single oocyte scaled, colored and shaped according to corresponding size (μm), cohort and developmental stage respectively.

C Gene expression responses to effects of *in vitro* culture. *X*-axis represents expression of genes for *in vivo*-derived oocytes at corresponding stage and *Y*-axis represents the quantified expression responses of each gene (log2(Fold-change)) for each stage. Colored points and numbers in red and blue represent genes whose expression is significantly (with FDR $\leq$ 5% and $|\log2(\text{Fold-change})| \geq 2$) affected by a corresponding stage of the *in vitro* development.

D Grouping of genes according to the dynamics of expression between GROs and FGOs. All genes were classified into four groups, representing genes with Low Stable expression (LS group), up-regulated from GRO to FGO (UP group), down-regulated from GRO to FGO (DN group), and genes with High Stable expression (HS group).

E Genes which are upregulated in FGOs relative to GROs are overrepresented among genes affected by any of the stages of the *in vitro* culture. Pie chart represents numbers and fraction of genes belonging to each group in (D) among all genes (left pie chart "All genes") and genes which are affected in any of the stages of the *in vitro* culture (right pie chart "Affected genes"). Results of χ^2 tests and enrichments of each group among affected genes are displayed below the pie charts.

F Results of χ^2 tests and enrichments of each group among genes with different response (up-regulation or down-regulation) to each stage of the *in vitro* culture. Groups which have χ^2 test *P*-value larger than 1% are displayed as dots and considered statistically not significant (n.s.).

Source data are available online for this figure.

© 2023 The Authors

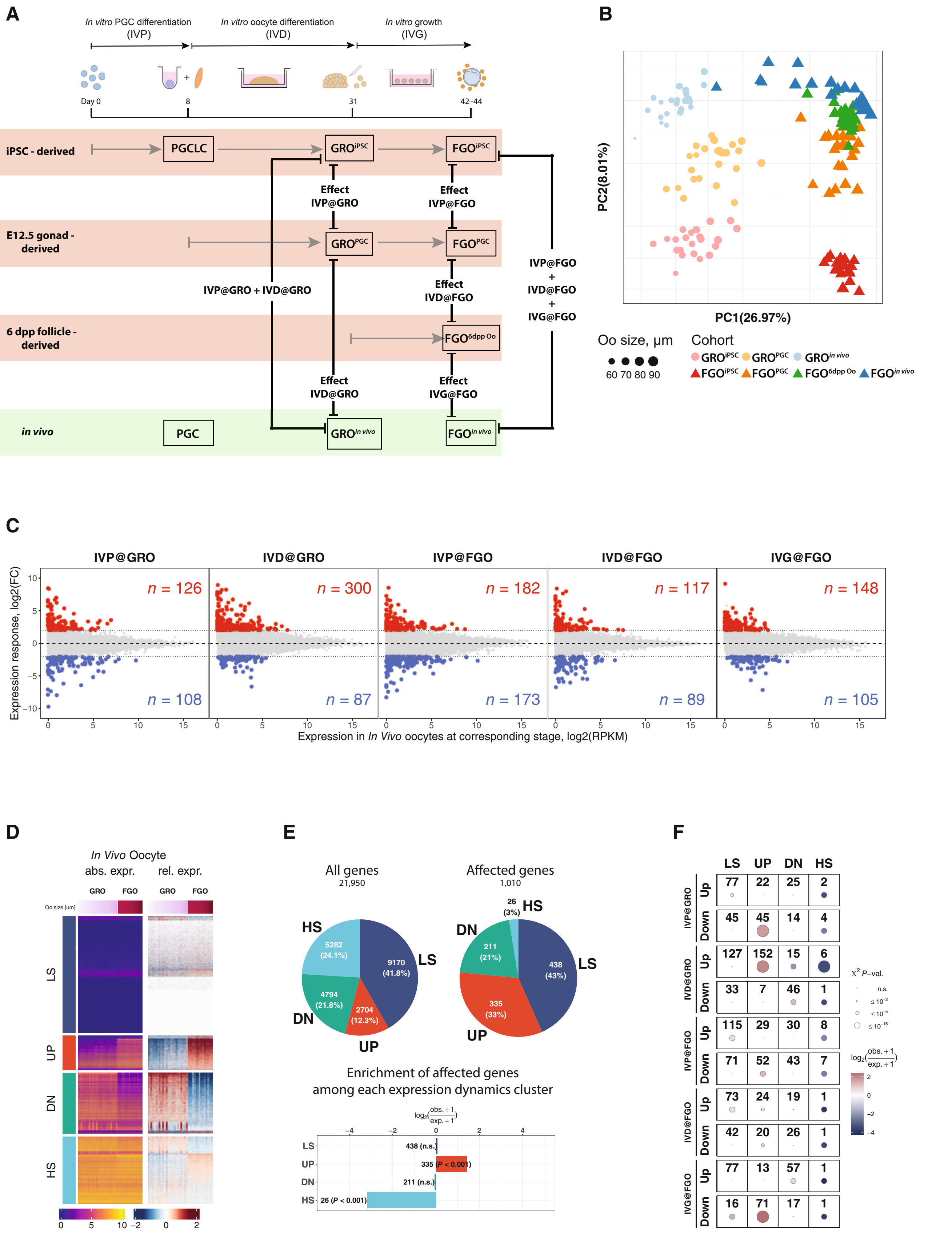
A
In vitro PGC differentiation (IVP)
In vitro oocyte differentiation (IVD)
In vitro growth (IVG)
Day 0
8
31
42–44
iPSC - derived
PGCLC
GRO iPSC
FGO iPSC
E12.5 gonad - derived
GRO PGC
FGO PGC
6 dpp follicle - derived
FGO 6dpp Oo
in vivo
PGC
GRO in vivo
FGO in vivo
Effect IVP@GRO
Effect IVP@FGO
Effect IVD@FGO
Effect IVD@GRO
Effect IVG@FGO
IVP@GRO + IVD@GRO
IVP@FGO + IVD@FGO + IVG@FGO
B
PC1(26.97%)
PC2(8.01%)
Oo size, µm
60 70 80 90
Cohort
C
IVP@GRO
IVD@GRO
IVP@FGO
IVD@FGO
IVG@FGO
n = 126
n = 108
n = 300
n = 87
n = 182
n = 173
n = 117
n = 89
n = 148
n = 105
Expression response, log2(FC)
Expression in In Vivo oocytes at corresponding stage, log2(RPKM)
D
In Vivo Oocyte
abs. expr.
rel. expr.
GRO
FGO
Oo size [um]
LS
UP
DN
HS
E
All genes
21,950
Affected genes
1,010
HS
5282 (24.1%)
9170 (41.8%)
LS
4794 (21.8%)
DN
2704 (12.3%)
UP
26 (3%)
211 (21%)
438 (43%)
335 (33%)
Enrichment of affected genes among each expression dynamics cluster
438 (n.s.)
335 (P < 0.001)
211 (n.s.)
26 (P < 0.001)
F
LS UP DN HS
IVP@GRO Up 77 22 25 2
IVP@GRO Down 45 45 14 4
IVD@GRO Up 127 152 15 6
IVD@GRO Down 33 7 46 1
IVP@FGO Up 115 29 30 8
IVP@FGO Down 71 52 43 7
IVD@FGO Up 73 24 19 1
IVD@FGO Down 42 20 26 1
IVG@FGO Up 77 13 57 1
IVG@FGO Down 16 71 17 1
X² P-val.
n.s.

Figure 5.

within promoters of genes with statistically significant expression response to steps of *in vitro* culture and particular expression dynamics from GRO to FGO *in vivo* (Fig 5F) using R/Bioconductor package *monaLisa* (Machlab *et al*, 2022) and transcription factor binding profile database *JASPAR2020* (Fornes *et al*, 2020). Our analysis did, however, reveal only one TF motif with a false discovery rate slightly below our cutoff (FDR $\leq$ 1%; Fig EV4G and Dataset EV6). The RXRA::VDR motif is enriched within promoters of genes which are down-regulated by IVG@FGO and belong to DN group (Figs 5F and EV4G) and represents binding specificity of heterodimer between retinoid X receptor alpha (*Rxra*) and vitamin D3 receptor (*Vdr*). However, the *Rxra* gene does not have a significantly altered expression response to IVG@FGO and the *Vdr* gene is not expressed in any of the oocyte cohorts (Dataset EV4), hence it is unlikely that these genes are responsible for observed expression responses of targets to IVG@FGO. Nevertheless, our analysis does not rule out the possibility of other transcription factors with similar sequence specificity to RXRA::VDR motif or transcription factors with sequence specificities not included in JASPAR2020 database to play a role. We finally asked whether any correspondence exists between genes differently expressed in PGCLCs and PGCs and genes affected during *in vitro* oogenesis (Figs 5C and EV4B). For genes up- and down-regulated in d6PGCLCs relative to E12.5 PGCs, we did not observe apparent correspondences to the effects of *in vitro* culture in GROs and FGOs (Fig EV4H). In summary, our analysis indicates that the observed step-specific effects of *in vitro* culture on gene expression are unlikely explained by either CpG promoter composition, enrichment of TF motifs or by expression differences between PGCLCs and PGCs.

In vitro differentiation results in premature activation of gene expression

We next investigated chromatin modifications at promoters of genes that were affected by the *in vitro* culture. We performed non-parametric Wilcoxon tests for a panel of publicly available ChIP-seq datasets to investigate differences in enrichments for selected chromatin marks between promoters of affected and unaffected genes with the same expression dynamics *in vivo* (groups LS, UP, DN, and HS) and presence or absence of a CGI promoter (Fig 6A). Remarkably, CGI promoters ($\pm$1.5 kb around TSS) of affected genes with UP and DN dynamics were enriched with repressive histone marks catalyzed by PRCs, such as H3K27me3 and H2AK119ub1, and depleted for active marks, such as H3K4me3 and H3K27ac, in PGCs, PGCLCs as well as in oocytes including GRO and FGO. This suggests that CGI-driven genes which showed expression changes during normal development from GRO to FGO (groups UP and DN) and were aberrantly expressed in the *in vitro* system are generally controlled by PRCs during oogenesis.

To further elucidate the characteristics of genes affected by the *in vitro* development we applied k-means clustering for log-fold-change expression responses (right columns, “relative expression,” Fig 6B) to assign affected genes into groups characterized by combinations of gene expression responses to the *in vitro* culture treatment (left columns, Fig 6B). Interestingly, clusters UP2 and UP3, which showed up-regulated expression from GRO to FGO *in vivo* (UP-group), contained genes (Dataset EV7) whose response to the IVD at GRO was highly positive, indicating aberrant premature activation of these genes during IVD in iPSC- and E12.5 gonad-derived oocytes.

Figure 6. Dynamics of epigenetic chromatin marks during oogenesis is distinct at CGI promoters of genes affected by *in vitro* culture procedure.

A Differences of histone post-translational modifications (PTMs) in PGC, PGCLC, NGO at day 5 and FGO (data from Kurimoto *et al*, 2015; Hanna *et al*, 2018b; Kawabata *et al*, 2019; Mei *et al*, 2021) at promoters of affected genes compared to non-affected genes in the same group. Results of Mann–Whitney tests are displayed for comparison of enrichment of each chromatin mark at promoters of affected genes compared to non-affected genes belonging to the same group. Analyses were separately done for CGI and non-CGI promoter genes.

B K-means clustering of genes according to combination of gene expression responses to stages of the *in vitro* protocol (heatmap Expression response). Expression in *in vivo* oocytes is shown to illustrate dynamics from GRO to FGO (heatmap Expression *in vivo*). Relative expression of genes in oocytes belonging to different cohorts in GRO (GRO^{iPSC}, GRO^{PGC}, $GRO^{in\ vivo}$) and FGO (FGO^{iPSC}, FGO^{PGC}, $FGO^{6dpp\ Oo}$ and $FGO^{in\ vivo}$) is also depicted. Relative expression for each gene was calculated as the difference between expression (log2(RPKM)) in each oocyte and average expression across all oocytes for GRO and FGO separately.

C Boxplots displaying enrichments of histone PTMs catalyzed by repressive Polycomb group complexes at CGI and non-CGI promoters of genes with different expression dynamics during *in vivo* development (LS, UP, DN and HS, see Fig 5C) and affected genes in UP group sub-clusters, defined based on combination of expression responses (as shown in Fig 6B; H3K27me3 data from Hanna *et al*, 2018b, H2AK119ub1 data from Mei *et al*, 2021). Lower hinge, central line and upper hinge represent 25th, 50th (median) and 75th percentiles respectively. Upper/lower whiskers extend to the largest/smallest values no further than 1.5*IQR from the upper/lower hinge, where IQR is interquartile range or distance between 25th and 75th percentiles. Outliers are not displayed. The notches extend $1.5 * IQR/\sqrt{n}$ where *n* are numbers of CGI and non-CGI genes belonging to each group displayed below boxplots for H3K27me3 in NGO and the same for other stages and PTMs.

D Boxplots displaying enrichments of histone PTMs catalyzed by repressive Polycomb group complexes at CGI and non-CGI promoters of genes with different expression dynamics during *in vivo* development (LS, UP, DN and HS, see Fig 5C) and affected genes in DN group sub-clusters, defined based on combination of expression responses (as shown in Fig 6B; H3K27me3 data from Hanna *et al*, 2018b, H2AK119ub1 data from Mei *et al*, 2021). Definitions of the central line, hinges, whiskers and notches are the same as in Fig 6C. Numbers of CGI and non-CGI genes in each group are displayed below boxplots for H3K27me3 in NGO and the same for other stages and PTMs.

E Genomic snapshot of a representative gene *Pla2g12b* belonging to UP2 cluster (see Fig 6B) and distribution of several chromatin marks. The analysis suggests premature activation of the gene in GRO caused by the IVD of the protocol, possibly due to premature removal of the Polycomb group epigenetic marks H3K27me3 or H2AK119ub1.

F Expression responses to each stage of *in vitro* development of genes involved in epigenetic regulation and transcriptional reprogramming (heatmap *Expression response*, only genes significantly affected by *in vitro* development or significantly differentially expressed in PGC and PGCLC (FDR $\leq$ 5% and |log2(Fold-change)| $\geq$ 1), all log2(Fold-change) with FDR $\geq$ 5% are considered non-significant and set to 0). In addition, expression in *in vivo* oocytes (heatmap *Expression in vivo*), and relative expression in PGC and PGCLC, GRO and FGO are displayed (group of heatmaps *Relative expression*, data for biological replicates or single oocytes belonging to particular cohorts were merged). Relative expression for each gene was calculated as difference between expression level (log2(RPKM)) and average expression calculated separately for PGC and PGCLC, GRO, and FGO.

Source data are available online for this figure.

© 2023 The Authors

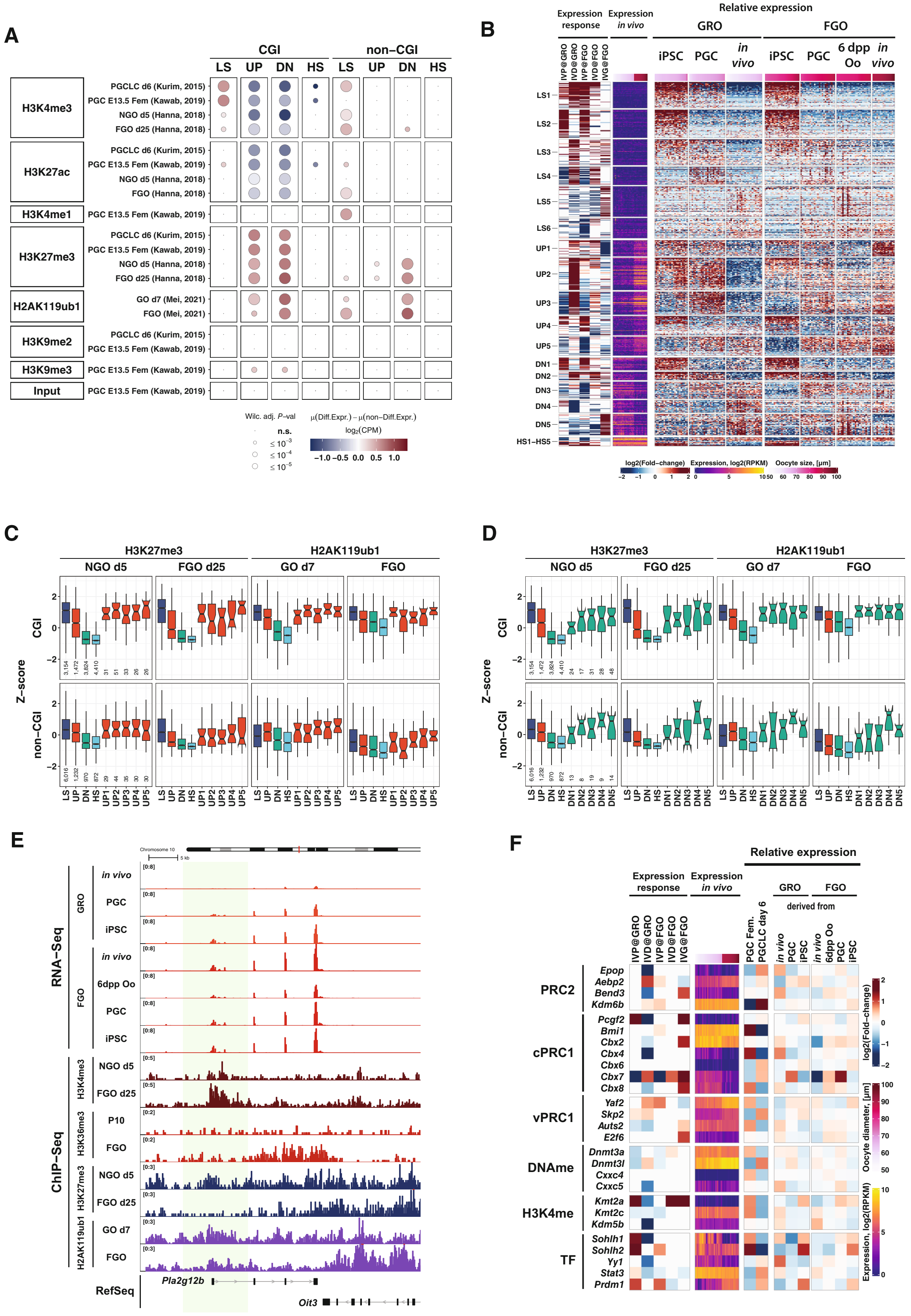

A
B
C
D
E
F
CGI
non-CGI
H3K4me3
H3K27ac
H3K4me1
H3K27me3
H2AK119ub1
H3K9me2
H3K9me3
Input
Relative expression
GRO
FGO
RNA-Seq
ChIP-Seq
RefSeq
Pla2g12b
Oit3
PRC2
cPRC1
vPRC1
DNAme
H3K4me
TF

Figure 6.

The differences in expression of genes belonging to clusters UP2 and UP3 may possibly be confounded by slight differences in growth of oocytes during their development *in vitro* and *in vivo*, given that oocytes in the GRO^{iPSC} and GRO^{PGC} cohorts were larger in size compared to oocytes in the *in vivo*-grown $GRO^{in\ vivo}$ cohort (Fig EV5A). When taking oocyte diameter into account, our analysis clearly shows that up-regulated expression of genes belonging to UP2 and UP3 in GRO^{iPSC} and GRO^{PGC} cohorts was also observed in those oocytes having the same size as oocytes in the $GRO^{in\ vivo}$ cohort (Fig EV5B). Moreover, if expression differences observed for UP2 and UP3 genes would have been confounded by differences in oocyte sizes in the three cohorts, we should have observed a general up-regulation of all genes with up-regulation dynamics from GRO to FGO, which we did not (UP group; Fig EV5B). In summary, genes in UP2 and UP3 are differently affected by IVD compared to all genes in the UP group. The data suggests that the IVD procedure leads to premature activation of these genes in GRO^{PGC} and partially in GRO^{iPSC} cohorts.

Polycomb-marked genes undergo premature activation during IVD

As we observed a noticeable difference in enrichments of PRC1 and PRC2 chromatin marks at promoters of affected genes belonging to the UP and DN groups, we hypothesized that these genes undergo chromatin remodeling during normal oocyte development. We quantified ChIP-seq enrichments at CGI and non-CGI promoters of genes (±1.5 Kb around TSS) belonging to clusters formed by gene expression dynamics and magnitude of response to *in vitro* differentiation (see Fig 6B, e.g. UP1-UP5, DN1-DN5, etc) and compared them to enrichments of all analyzed genes with different expression dynamics, i.e. LS, UP, DN and HS groups (Figs 6C and D, and EV5C–H). As expected, we found that promoters of genes in the LS group which exhibited low stable expression both in GRO and FGO were highly enriched with repressive histone marks, such as H3K27me3 and H2AK119ub1, and showed low enrichments of marks associated with transcriptional activity, such as H3K4me3, both in day 5 non-growing oocytes (NGOs) and FGO (Hanna *et al*, 2018b) compared to genes in the UP, DN and HS groups (Figs 6C and EV5C). In contrast, promoters of genes which were highly expressed both in GRO and FGO (HS group) were highly enriched with H3K4me3 compared to genes in the LS, UP and DN groups (Fig EV5C).

When we compared enrichments of H3K27me3 and H3K4me3 histone marks at promoters of affected genes to all genes with UP expression dynamics *in vivo*, we observed higher and lower enrichments, respectively, for all clusters regulated by CGI promoters in NGOs (Figs 6C and EV5C; Hanna *et al*, 2018b) as well as in E13.5 PGCs (Kawabata *et al*, 2019) and d6 PGCLCs (Kurimoto *et al*, 2015; Fig EV5D). These observations are consistent with our measurements of enrichment of CGI promoters with histone marks (Fig 6A) and suggested that CGI genes that are affected by the *in vitro* culture are enriched for PRC targets. Further, when we analyzed H3K27me3 enrichments in FGO and compared them to NGO oocytes (Fig 6C), we observed that CGI genes in clusters UP1-UP5 exhibited cluster-specific changes in H3K27me3 enrichments, with genes with CGI promoters in UP2 showing a most noticeable removal of H3K27me3 from their promoters. In addition, CGI and non-CGI-affected genes in DN group (i.e. DN1-DN5 clusters) showed various dynamics of H3K27me3 enrichment from NGO to FGO (Fig 6D). These observations suggest likely aberrant effects of PRC-mediated repression of genes belonging to UP2 and partially to UP3 clusters during the IVD treatment. We speculate, that genes belonging to UP2 and UP3 clusters were enriched for PRC1/2 targets in PGCs, PGCLCs as well as in NGOs and were subject to removal of repressive H3K27me3 histone marks to become up-regulated in the FGO *in vivo* (Figs 6E, and EV5I and J). The IVD treatment possibly caused instability and premature removal of these repressive marks from CGI promoters in UP group leading to premature activation of these genes.

Following this hypothesis, we investigated whether expression of genes involved in epigenetic regulation and transcriptional reprogramming was affected by *in vitro* oocyte development (Fig 6F and Appendix Fig S1). Remarkably, we observed that the expression of several components of the canonical PRC1 complex was highly variable between growth conditions. For example, *Bmi1/Pcgf4* was downregulated in PGCLC versus PGC development while regulation of *Pcgf2* was labile in the IVD and IVG. The five *Cbx2*, *4*, *6*, *7*, and *8* genes were variably regulated under the *in vitro* culture as well (Fig 6F). Likewise, components of variant PRC1 complexes such as *Yaf2*, *Kdm2b*, *Bcorl1*, *Skp2* and *Auts2* were to some extent also variably expressed (Appendix Fig S1). The role of such PRC1 complex members in the control of specific genes remains to be explored. Genetic loss-of-function studies revealed functional redundancy as well as critical roles of major PRC1 core components in PGC and oocyte development (Posfai *et al*, 2012; Yokobayashi *et al*, 2013). For members of the PRC2 complex, we observed deregulated expression of *Jarid2* and *Aebp2*, encoding two proteins involved in reading PRC1-catalyzed H2AK119ub1 and promoting PRC2 catalytic activity toward H3K27me3 (Kasinath *et al*, 2021). Expression of the *Kdm6b* H3K27me3 demethylase was also variable between culture conditions. Finally, *Bend3* encoding a protein implicated in PRC2 recruitment to paternal constitutive heterochromatin in early mouse embryos was significantly downregulated in IVD and upregulated in IVG (Saksouk *et al*, 2014). Taken together, we hypothesize that alterations in the expression of particular PRC2 and PRC1 components may impact the temporal dynamics of repression of target genes during *in vitro* germ cell development.

Discussion

In this study, we recapitulate the differentiation of mouse PSCs to MII oocytes in culture with a comparable efficiency as previously reported (Hikabe *et al*, 2016). While the procedure yields many morphologically well-developed oocytes, competence to support early embryonic development is limited. Our analysis identifies the inability of parthenogenic eggs to initiate transcription as a major roadblock for successful embryonic development (Fig 4). Given the importance of nuclear PDH in regulating ZGA (Nagaraj *et al*, 2017), the reduced nuclear localization of PDH in PA embryos may also contribute to their low embryonic competence. Moreover, we observed abnormal acquisition of 5hmC and a failure of the STELLA protein to localize in the nucleus in BVSC-iPSC-derived parthenotes. These data are compatible with a report that *Stella*-null embryos showed ectopic appearance of 5hmC in maternal chromatin, which induced abnormal accumulation of γH2AX and subsequent growth

 © 2023 The Authors

retardation (Nakatani *et al*, 2015). Ectopic acquisition of 5hmC in PSC-derived embryos likely results from TET3-mediated conversion of 5mC, even though 5mC levels in PSC-derived 2-cell embryos were comparable to that in *in vivo*-derived embryos. Such differential response may in part be due to different sensitivity of antibodies for the respective epitopes. Possibly, reduced nuclear STELLA levels may impact on passive DNA demethylation. STELLA-mediated demethylation is achieved via inhibition of UHRF1 chromatin binding, thereby preventing DNMT1-mediated maintenance methylation, and is attenuated by nuclear export of STELLA (Li *et al*, 2018; Du *et al*, 2019). In mouse zygotes, cytoplasmic-localized STELLA undergoes ubiquitin-induced proteolytic cleavage (Shin *et al*, 2017). Proteosome-mediated degradation is incomplete and results in the association of a N-terminal STELLA fragment with early and recycling endosomal vesicles. Genetic experiments indicate that such cytoplasmic function is important for endo/exocytosis and required for pre-implantation development. Based on the reduced STELLA protein levels in iPSC-derived zygotes, we speculate that factors and processes involving the ubiquitin-proteosome system or endo/exocytosis are in part deregulated upon *in vitro* oocyte generation. Since most *in vitro*-derived oocytes are morphologically indistinguishable from *in vivo*-derived oocytes, identification of intracellular abnormalities in *in vitro*-derived oocytes provides molecular markers for further improving the culture system.

IVD culture step has a crucial effect on oocyte quality

Our data indicate that the low competence for preimplantation development results predominantly from IVD, and to a lesser extent from IVG. For IVD, E12.5 gonads are dissociated, and gonadal somatic cells are mixed with d6 PGCLCs to form rOvaries. Transcriptional analyses have shown that d6 PGCLCs are comparable to migrating PGCs at E9.5 (Hayashi *et al*, 2011, 2012). Therefore, rOvaries consisting of d6 PGCLCs and E12.5 gonadal somatic cells contain developmentally heterochronic cell populations. Such heterochrony might cause aberrant follicle development during IVD. Ohta *et al* (2017) reported the *in vitro* expansion of PGCLCs advanced their epigenetic reprogramming, including global DNA demethylation and up-regulation of H3K27me3, as in gonadal PGCs. Therefore, it is possible for the improvement of IVD to incorporate the extended culture of PGCLCs before formation of rOvaries, which presumably bridge the developmental gap between PGCLCs and gonadal somatic cells. Nevertheless, our experimental data indicated inconsiderable differences in developmental competence between iPSC-derived oocytes, developed in rOvaries consisting of d6 PGCLCs and E12.5 gonadal somatic cells, and E12.5 gonad-derived oocytes, developed in rOvaries consisting of E12.5 PGCs and E12.5 gonadal somatic cells (Fig 3 and Table EV1). Considering these data, not only the heterochrony between d6 PGCLC and E12.5 gonadal somatic cells but also the act of disrupting and reconstituting cellular interactions between PGCLCs and gonadal cells and culture conditions may impact on developmental competence.

PGCs develop into oocytes through formation of cysts and subsequently into follicles by close interaction with surrounding gonadal cells (O'Connell & Pepling, 2021; Niu & Spradling, 2022). In the IVD system, E12.5 gonads are dissociated, and gonadal somatic cells are mixed with PGCLCs to form rOvaries at day 8 of the culture. It is possible that components in gonads, especially the basement membrane and extracellular matrix (ECM), which contribute to achieve proper cell–cell interaction, were damaged or lost during this procedure. Transcriptome analysis using transplanted gonads/ovaries suggested development of GROs during the perinatal period is markedly subject to the ECM, which is also involved in oocyte dormancy (Nagamatsu *et al*, 2019). Our single oocyte transcriptome analysis revealed upregulation of genes involved in cell adhesion in GROs due to IVP and IVD and downregulation during IVG, possibly suggesting stage-specific adaptation toward an altered extracellular environment (Fig EV4E). Targeting the lack of extracellular components in rOvaries might improve the culture system and lead to *in vitro*-derived oocytes with higher embryonic competence after fertilization.

Transcriptome comparison and modeling identifies differential expression of Polycomb target genes during IVD between *in vitro* and *in vivo*-grown germ cells

In our study, we generated a large number of single oocyte RNA-Seq datasets as a resource for understanding the influence of each culture step on oocyte quality. For this, we benchmark the performance of *in vitro* and *in vivo*-grown germ cells at different developmental stages. Our comparative expression analysis demonstrated that genes normally upregulated during oocyte growth are particularly vulnerable to *in vitro* culture conditions, leading to either aberrant up- or down-regulation in a development-specific manner. Our epigenomic analysis indicates that many of such deregulated genes are normally controlled by PRC2 and PRC1 complexes catalyzing repressive H3K27me3 and H2AK119ub1 histone modifications thereby formatting repressive chromatin states. Since such modifications can also be removed by respective histone demethylases and de-ubiquitinating enzymes expressed in PGCs and oocytes (Fig 6F and Appendix Fig S1), *in vitro* culturing conditions likely result in altered expression of Polycomb target genes during oocyte growth. Genetic loss-of-function studies combined with spindle transfer experiments have identified a critical role for major PRC1 core components in oocyte development and specifying the maternal contribution required for zygotic transcription, timing of embryonic replication and embryonic development (Posfai *et al*, 2012). Moreover, Hikabe *et al* (2016) reported that embryos obtained from *in vitro*-generated oocytes were characterized by enlarged placentae. This finding is reminiscent of a failure in establishing non-canonical imprinted repression through PRC2-mediated H3K27me3 within the maternal genome in *in vitro*-derived oocytes (Inoue *et al*, 2017a; Matoba *et al*, 2022). Also, a recent report identified a crucial role of PRC1-mediated epigenetic programming in controlling the dictyate arrest of oocytes and primordial follicle reserve, consistent with the observation of non-arrest at primordial follicle stages under the *in vitro* culture (Shimamoto *et al*, 2019; Hu *et al*, 2022). Hence, deregulation of Polycomb repression during *in vitro* oogenesis may directly or indirectly alter the maternal load of transcripts and proteins, as well as formatting the chromatin landscape in oocytes that normally confer embryonic competence, and possibly regulate ZGA upon fertilization.

The *in vitro* culture system for generating oocytes from PSCs has enormous potential for understanding germline development. Recently, follicles have been generated entirely from mouse PSCs without the use of donor tissues *in vitro* (Yoshino *et al*, 2021). Also,

© 2023 The Authors

functional oocytes were generated from PSCs of male mice by the *in vitro* culture (Murakami *et al*, 2023). Thus, wider application for mechanistic studies as well as new avenues in assisted reproduction is anticipated (Saitou & Hayashi, 2021; Cyranoski *et al*, 2023). Similar technology is already considered for obtaining human germ cells (Irie *et al*, 2015; Sasaki *et al*, 2015; Yamashiro *et al*, 2018; Hwang *et al*, 2020), and PGCLCs from endangered species to rescue animals from extinction (Hayashi *et al*, 2022). Our work provides molecular insights into *in vitro* oogenesis and identifies critical steps to direct efforts for future improvement.

Materials and Methods

Reagents and Tools table

Reagent/resource	Reference or source	Identifier or catalog number
**Experimental models**		
C57BL/6J (*Mus musculus*)	The Jackson Laboratory	Strain #: 000664
CAST/EiJ (*M. musculus*)	The Jackson Laboratory	Strain #: 000928
DBA/2J (*M. musculus*)	The Jackson Laboratory	Strain #: 000671
129S6/SvEvTac (*M. musculus*)	Taconic Biosciences	Strain #: 129SVE-F, 129SVE-M
Swiss Webster (*M. musculus*)	Taconic Biosciences	Strain #: SW-F, SW-M
Mouse ESC line (GFP-ESC)	In house	
Mouse ESC line (BVSC-ESC)	Hayashi *et al* (2012)	
Mouse iPSC line (BVSC-iPSC)	Katsuhiko Hayashi provided; Hikabe *et al* (2016)	
**Antibodies**		
5-Hydroxymethylcytosine	Active Motif	Cat #: 39069, RRID: AB_10013602
Anti-5-Methylcytosine Mouse mAB	Millipore	Cat #: NA81-50UG, RRID:AB_213180
Anti-BrdU	Merck	Cat #: B8434-25UL, RRID:AB_476811
Anti-Histone H3 (acetyl K4)	Abcam	Cat #: ab176799, RRID:AB_2891335
Anti-Human/Mouse SSEA-1 Alexa Fluor 660	eBioscience	Cat #: 50-8813-42, RRID:AB_11219681
Anti-Pyruvate Dehydrogenase	Abcam	Cat #: ab177461, RRID:AB_2756339
Anti-SSEA-1 (CD15) MicroBeads	Miltenyl Biotec	Cat #: 130-094-530, RRID:AB_2814656
Anti-STELLA antibody	Abcam	Cat #: ab19878, RRID: AB_2246120
CD31 MicroBeads	Miltenyl Biotec	Cat #: 130-097-418, RRID:AB_2814657
Donkey anti mouse IgG 647	Jackson ImmunoResearch	Cat #: 715-605-150, RRID:AB_2340862
Donkey anti mouse IgG Cy3	Jackson ImmunoResearch	Cat #: 715-165-150, RRID:AB_2340813
Donkey anti rabbit IgG 647	Jackson ImmunoResearch	Cat #: 711-605-152, RRID:AB_2492288
Donkey anti rabbit IgG Cy3	Jackson ImmunoResearch	Cat #: 711-165-152, RRID:AB_2307443
Histone H3K27ac	Active Motif	Cat #: 39685, RRID: AB_2793305
Histone H3K27me3	Active Motif	Cat #: 39155, RRID: AB_2561020

 © 2023 The Authors

Reagents and Tools table (continued)

Reagent/resource	Reference or source	Identifier or catalog number
Histone H3K4me3	Active Motif	Cat #: 61379, RRID: AB_2793611
PDH-E1alpha (D-6)	Santa Cruz Biotechnology	Cat #: sc-377092, RRID:AB_2716767
PE anti-mouse/rat CD61	BioLegend	Cat #: 104307, RRID: AB_313084
**Oligonucleotides and sequence-based reagents**		
Blimp1-mVenus-5: ACT CAT CTC AGA AGA GGA TCT G	Microsynth; Hayashi *et al* (2012)	
Blimp1-mVenus-3: CAC AGT CGA GGC TGA TCT CG	Microsynth; Hayashi *et al* (2012)	
Prdm14 WT-5: AAG GTT CTG GGA ACT GGA TGT C	Microsynth; Hayashi *et al* (2012)	
Prdm14 WT-3: CAC AAT ATG CTG GCA TGC GTT C	Microsynth; Hayashi *et al* (2012)	
Stella-CFP-5: CGA GCT AGC TTT TGA GGC TT	Microsynth; Hayashi *et al* (2012)	
Stella-CFP-3: AAC TTG TGG CCG TTT ACG TC	Microsynth; Hayashi *et al* (2012)	
SRY2: TCT TAA ACT CTG AAG AAG AGA C	Microsynth	
SRY4: GTC TTG CCT GTA TGT GAT GG	Microsynth	
Xist-14: GTA GAT ATG GCT GTT GTC AC	Microsynth	
Xist-16: CTC CAT CCA AGT TCT TTC TG	Microsynth	
TSO (template-switching oligos): AAG CAG TGG TAT CAA CGC AGA GTA CAT rGrG+G	Exiquon; Picelli *et al* (2014)	
Oligo-dT30VN: AAG CAG TGG TAT CAA CGC AGA GTA CTT TTT TTT TTT TTT TTT TTT TTT TTT TTT TVN	Microsynth; Picelli *et al* (2014)	
ISPCR primers: AAG CAG TGG TAT CAA CGC AGA GT	Microsynth; Picelli *et al* (2014)	
Nextera XT Index Kit v2 Set A (96 indexes, 384 samples)	Illumina	FC-131-2001
Nextera XT Index Kit v2 Set B (96 indexes, 384 samples)	Illumina	FC-131-2002
**Chemicals, enzymes and other reagents**		
Activin A	Peprotech	Cat #: 120-14
AMPure XP beads	Beckman Coulter	Cat #: A63881
Betaine Solution	Merck	Cat #: B0300-1VL
bFGF	Peprotech	Cat #: 100-18B
BMP15	Abcam	Cat #: ab127067
BMP4	Peprotech	Cat #: 315-27
CHIR99021	Axon Medchem	Cat #: 1386
Collagenase	Worthington Biochemicals	Cat #: LS004196
DNA polymerase	Thermo Fisher	Cat #: F549S
EGF	Peprotech	Cat #: 315-09
ERCC RNA Spike-In Mix	Thermo Fisher Scientific	Cat #: 4456740
FSH	MSD	Puregon 600
Fulvestrant	Merck	Cat #: I4409
GDF9	R&D systems	Cat #: 739-G9-010
KAPA HiFi HotStart Ready mix	Roche	Cat #: KK2601
KSOM	Merck	Cat #: MR-020P-5F
LIF	Homemade	N/A
ORIGIO Sequential Fert	CooperSurgical	Cat #: 83020010
PD0325901	Axon Medchem	Cat #: 1408
Phusion High-Fidelity DNA Polymerase	Thermo Fisher Scientific	Cat #: F530L
SCF	Peprotech	Cat #: 250-03

© 2023 The Authors

Reagents and Tools table (continued)

Reagent/resource	Reference or source	Identifier or catalog number
SUPERase-In Rnase inhibitor	Thermo Fisher Scientific	Cat #: AM2696
Superscript-II	Thermo Fisher Scientific	Cat #: 18064014
Tn5-transposase	FMI homemade	N/A
**Software**		
edgeR	McCarthy *et al* (2012), https://bioconductor.org/packages/release/bioc/html/edgeR.html	
FastQC (v0.11.8)	Babraham Bioinformatics; Krueger (2015), https://www.bioinformatics.babraham.ac.uk/projects/fastqc/	
Fiji	https://imagej.net/software/fiji/	
Graphpad Prism 9	GraphPad Software, www.graphpad.com	
Kaluza	Beckman Coulter, www.beckman.com/flow-cytometry/software/kaluza	
monaLisa	Machlab *et al* (2022), https://bioconductor.org/packages/release/bioc/html/monaLisa.html	
org.Mm.eg.db (v3.15.0)	Bioconductor, https://bioconductor.org/packages/release/data/annotation/html/org.Mm.eg.db.html	
QuasR	Gaidatzis *et al* (2015), https://bioconductor.org/packages/release/bioc/html/QuasR.html	
R	R Core Team, https://www.R-project.org/	
Samtools	Samtools, http://www.htslib.org	
SRA-Toolkit	NIH, https://hpc.nih.gov/apps/sratoolkit.html	
STAR aligner (v2.5.0a)	Dobin *et al* (2013), https://github.com/alexdobin/STAR	
topGO (v2.48.0)	Alexa & Rahnenfuhrer (2022), https://bioconductor.org/packages/release/bioc/html/topGO.html	
TxDb.Mmusculus.UCSC.mm10.knownGene (v3.2.2)	Bioconductor, https://bioconductor.org/packages/release/data/annotation/html/TxDb.Mmusculus.UCSC.mm10.knownGene.html	
ZEN Microscopy Software	Zeiss, www.zeiss.com/zen	
**Other**		
Deposited data: RNA-Seq of single *in vivo* and *in vitro* oocytes and bulk female E12.5 PGC and day 6 PGCLC	This paper	GEO:GSE223479
Deposited data: ChIP-Seq for day 6 PGCLC	Kurimoto *et al* (2015)	GEO:GSE60204
Deposited data: ChIP-Seq for female E13.5 PGC	Kawabata *et al* (2019)	DDBJ:DRA006633
Deposited data: CUT&RUN for H2AK119ub1 during oogenesis	Mei *et al* (2021)	GEO:GSE153496
Deposited data: RNA-Seq for day 6 PGCLC	Sasaki *et al* (2015)	GEO:GSE67259
Deposited data: RNA-Seq for female E12.5 PGC	Ohta *et al* (2017)	GEO:GSE87644
Deposited data: ULI-nChIP-seq for NGO day 5 and FGO day 25	Hanna *et al* (2018a, 2018b)	GEO:GSE93941
NovaSeq 6000 S1 Reagent Kit v1.5 (100 cycles)	Illumina	Cat #: 20028319
RNase-Free DNase Set	Qiagen	Cat #: 79254
RNeasy Mini Kit	Qiagen	Cat #: 74104
Sera-Mag Select	Cytiva	Cat #: 29343045
TapeStation D1000 ScreenTape	Agilent	Cat #: 5067-5583
TapeStation RNA ScreenTape	Agilent	Cat #: 5067-5576
TruSeq Stranded mRNA Library Prep	Illumina	Cat #: 20020594
Sphericalplate 5D	Kugelmeiers	
Transwell-COL membrane insert	Corning	Cat #: 3492

© 2023 The Authors

Methods and Protocols

Mice

C57BL/6J, CAST/EiJ and DBA/2J mice were purchased from Jackson Laboratory. Swiss Webster and 129S6/SvEvTac mice were purchased from Taconic Biosciences. C57BL/6J females were mated with DBA/2J males to obtain hybrid mice (B6D2F1). All mice were housed in the animal facility of ETH Zurich. All animal experiments were performed under the license ZH152/17 in accordance with the standards and regulations of the Cantonal Ethics Commission Zurich.

Cell lines

C57BL/6J females were mated with 129S6/SvEvTac males to obtain hybrid embryos to establish ESC lines. After genotyping of sex (Aizawa *et al*, 2020), a female ESC line was co-transfected with a piggyBac vector carrying a CAG-EGFP-IRES-hygro transgene and a hyperactive piggyBac transposase expression plasmid (Yusa *et al*, 2011) using lipofectamine 2000 by following a manufacturer's protocol. Subsequently, single cells with EGFP expression were sorted by FACS. After cell growth, a single colony was used to establish the GFP-ESC line. The female BVSC-ESC line bearing Blimp1-Venus and Stella-ECFP transgenes was established as previously described (Hayashi *et al*, 2012). The female BVSC-iPSC line established from adult tail tip fibroblasts, also called iPS TTF_4FC6 (Hikabe *et al*, 2016), was a gift from Katsuhiko Hayashi. All these three cell lines were maintained feeder-free on ornithine- and laminin-coated plates using 2i + LIF medium (Hayashi & Saitou, 2013).

In vitro PGC differentiation (IVP)

Differentiation of PSCs into EpiLCs and PGCLCs was induced by following a previously published protocol with a few modifications (Hayashi & Saitou, 2013). For EpiLC differentiation, 3.4×10^5 PSCs per well were plated on a 6-well plate coated with 16.7 mg/ml human plasma fibronectin in EpiLC medium. For PGCLC differentiation, 2.25×10^5 EpiLCs per well were plated on a Spherical plates 5D plate (Kugelmeiers) with PGCLC medium containing BMP4 (500 ng/ml), SCF (100 ng/ml), LIF (1,000 IU/ml) and EGF (50 ng/ml) without BMP8a. The medium was changed at day 1 with EpiLC medium and at day 6 with GK15 medium supplemented with SCF (50 ng/ml), LIF (500 IU/ml) and EGF (25 ng/ml). Images of cells were acquired using an Olympus MVX10 Stereo-Zoom microscope equipped with an Olympus DP73 camera using the cellSens software.

In vitro oocyte differentiation (IVD)

The IVD culture condition was adapted from previously published protocols with some modifications (Hikabe *et al*, 2016; Hayashi *et al*, 2017). At day 8 of the culture, PGCLCs, positive for both Blimp1-Venus and Stella-ECFP or for both SSEA1 and integrin β3, were sorted using a FACSAria III (BD Bioscience). The collected PGCLCs were resorted once more for purification using the same gate by FACSAria III. At the same time, E12.5 female embryonic gonads, derived from outbred Swiss Webster mice, were harvested. To isolate gonadal somatic cells, PGCs were depleted by magnetic-activated cell sorting using both SSEA1 and CD31 antibodies coupled to magnetic beads (130-094-530 and 130-097-418, Miltenyi Biotech) in accordance with the manufacturer's protocol. PGCLCs were aggregated with isolated gonadal somatic cells in a low-binding 96-well plate (174929, Thermo Scientific) for 2 days in GK15 medium supplemented with 1 μM retinoic acid. 5,000 PGCLCs and 50,000 gonadal somatic cells were co-cultured to produce one rOvary. At day 10 of the culture, rOvaries were placed on Transwell-COL membranes (3492, Corning) in a 6-well plate, in which the membrane is contacted with the surface of α-IVD medium composed of αMEM supplemented with 2% FBS (A3161001, Thermo Fisher), 150 μM ascorbic acid (Merck), 55 μM β-mercaptoethanol (Thermo Fisher), 2 mM GlutaMAX (Thermo Fisher) and penicillin–streptomycin (Thermo Fisher). At day 12 of the culture, rOvaries were soaked in the medium by adding 2 ml of α-IVD medium per a well. At day 14, half of medium was changed to Stem-IVD medium composed of StemPro-34 SFM (Thermo Fisher) supplemented with 10% FBS, 150 μM ascorbic acid, 55 μM β-mercaptoethanol, 2 mM GlutaMAX and penicillin–streptomycin. At day 17, the medium was replaced with Stem-IVD medium supplemented with 600 nM fulvestrant (Merck). At day 21, the medium was changed to Stem-IVD medium without fulvestrant. Half of the medium was changed at days 12, 19, 21, 23, 25, 27 and 29.

For comparing the development, E12.5 gonad-derived rOvaries were also prepared. We dissociated E12.5 gonads and then aggregated all gonadal cells including PGCs in low-binding plates to reconstitute rOvaries for 2 days in GK15 medium supplemented with 1 μM retinoic acid. 55,000 gonadal cells were used to produce one rOvary. Then, rOvaries were placed on Transwell-COL membranes by following the similar IVD protocol as PSC-derived rOvaries for 21 days.

Samples were imaged under the microscope (Axio Observer Z1, Zeiss) equipped with an ORCA-Flash4.0 camera (Hamamatsu Photonics K.K.). Images were processed using Zeiss Zen Pro 2.0 software.

In vitro growth (IVG)

The IVG culture condition was adapted from previously published protocols with a few modifications (Hikabe *et al*, 2016; Hayashi *et al*, 2017). At day 31 of the culture, follicles in rOvaries were mechanically dissociated into either 1–3 or 4–10 follicles using 30G needles. Dissociated follicles were kept on the Transwell-COL membranes contacted with IVG medium composed of αMEM supplemented with 2% polyvinylpyrrolidone (PVP360, Merck), 5% FBS, 100 mIU/ml follicle-stimulating hormone (FSH; Puregon, MSD), 150 μM ascorbic acid, 55 μM β-mercaptoethanol, 2 mM GlutaMAX, 55 μg/ml sodium pyruvate and penicillin–streptomycin. Both 1–3 and 4–10 follicles were cultured together on the same Transwell-COL membranes to evaluate the development of follicles. At day 33, follicles were treated with collagenase by following previously published protocols (Hikabe *et al*, 2016; Morohaku *et al*, 2016; Hayashi *et al*, 2017). Briefly, follicles were incubated in 0.1% collagenase type I (Worthington Biochemicals) on the Transwell-COL membrane for 15 min. Then, follicles were washed three times by adding 1.5 ml of αMEM supplemented with 5% FBS under the membrane and aspirating the medium. Subsequently, the Transwell-COL membrane with follicles was placed into a new 6-well plate filled with 1.3 ml of IVG medium. From day 31 to 34, the IVG medium was supplemented with 15 ng/ml BMP15 (ab127067, Abcam) and 15 ng/ml GDF9 (739-G9-010, R&D Systems). At day 34, 2 ml of IVG

© 2023 The Authors

medium was added over the Transwell-COL membrane so that follicles were soaked in the medium. Half of the medium was replaced with fresh medium at day 36, 38, 40 and 42.

Follicles dissected from E12.5 gonad-derived rOvaries and from 6 dpp ovaries were also subjected to the IVG at day 23 of the culture and after the dissection of 6 dpp ovaries, respectively. The same IVG protocol including the collagenase treatment was applied to 4–10 follicles dissected from the rOvaries or from 6 dpp ovaries as follicles from PSC-derived rOvaries. The duration of the IVG was 11–12 days, which were 1–2 days shorter than that for PSC-derived follicles since dying cells emerged in several follicles when they were cultured for 13 days.

In vitro maturation (IVM)

The IVM culture condition was adapted from previously published protocols (Hikabe *et al*, 2016; Hayashi *et al*, 2017). At day 44 of the culture, oocytes with surrounding granulosa and cumulus cells were harvested from expanded follicles with a diameter of roughly over 200 μm at the longest axis using a fine glass capillary. These complexes were transferred to IVM medium composed of αMEM supplemented with 5% FBS, 100 mIU/ml FSH, 4 ng/ml EGF (315-09, PeproTech), 1.2 IU/ml human chorionic gonadotropin (HCG; C1063, Merck), 25 μg/ml sodium pyruvate and penicillin–streptomycin. At 16 h of the culture, swollen COCs were subjected to IVF or PA. The same IVM protocol was applied to expanded follicles derived from E12.5 gonads and 6 dpp ovaries at day 34–35 and at day 11–12 of the culture, respectively.

In vitro fertilization (IVF) and preimplantation development

Spermatozoa were collected from the cauda epididymis of male mice. B6D2F1 males were used for assessment of preimplantation development, and CAST/EiJ males were used for transcription analysis of embryos. Collected spermatozoa were capacitated by incubation for 1 h in Sequential Fert (83020010, ORIGIO). After capacitation, spermatozoa were incubated with COCs after the IVM in Sequential Fert for 6 h. The zygotes were collected and transferred to KSOM medium (MR-020P, Merck) for preimplantation development. After 2 days of the culture, embryos were transferred to fresh KSOM medium. The embryos were counted every single day to measure their developmental ratio. Statistical analysis was performed by GraphPad Prism 8 software using an unpaired two-tailed *t*-test.

Parthenogenetic activation (PA)

COCs were placed in M2 medium (M7167, Merck) and stripped from cumulus cells by treating with 0.1% hyaluronidase (H4272, Merck). MII oocytes were identified by their morphology with first polar body extrusion. All the oocytes harvested from COCs were transferred to activation medium composed of KSOM medium supplemented with 5 mM strontium chloride (13909, Merck) and 2 mM EGTA (A0878, AppliChem; Kishigami & Wakayama, 2007). After 6 h, activated embryos were transferred to KSOM medium for subsequent preimplantation development. After 2 days of the culture, embryos were transferred to fresh KSOM medium.

Assessment of follicle development

After the IVG culture, expansion of follicles derived from BVSC-iPSC, E12.5 gonad and 6 dpp follicle was assessed at day 44, day 34 and day 12 of the culture, respectively. Since follicles were not round, the longest part in each follicle was measured as a diameter under a stereomicroscope (SMZ745, Nikon) with an eyepiece reticle. Each follicle was categorized into one of three groups depending on its diameter: 0–200; 200–400; over 400 μm.

Genotyping

DNA extraction of cells was performed as previously described (Aizawa *et al*, 2020). PCR was performed using Phusion Hot Start II DNA Polymerase (Thermo Fisher Scientific) following the manufacturer's protocol. PCR products were separated by electrophoresis on 1.5% agarose gels and stained with ethidium bromide for visualization under a UV transilluminator. The following primer sequences were used for genotyping of sex and cell line identification: Blimp1-mVenus-5, 5′-ACT CAT CTC AGA AGA GGA TCT G-3′; Blimp1-mVenus-3, 5′-CAC AGT CGA GGC TGA TCT CG-3′; Prdm14 WT-5, 5′-AAG GTT CTG GGA ACT GGA TGT C-3′; Prdm14 WT-3, 5′-CAC AAT ATG CTG GCA TGC GTT C-3′; Stella-ECFP-5, 5′-CGA GCT AGC TTT TGA GGC TT-3′; Stella-ECFP-3, 5′-AAC TTG TGG CCG TTT ACG TC-3′; SRY2, 5′-TCT TAA ACT CTG AAG AAG AGA C-3′; SRY4, 5′-GTC TTG CCT GTA TGT GAT GG-3′; Xist-14, 5′-GTA GAT ATG GCT GTT GTC AC-3′; Xist-16, 5′-CTC CAT CCA AGT TCT TTC TG-3′.

Immunostaining analysis

PSC-derived 2-cell embryos were prepared by PA of oocytes after IVM. Control 2-cell embryos were prepared by activation of oocytes harvested from superovulated C57BL/6J females. Control 1-cell zygotes were prepared by IVF of oocytes and spermatozoa harvested from superovulated C57BL/6J females and B6D2F1 males, respectively.

For immunostaining of histone marks and STELLA, 2-cell embryos were collected at 48 h after HCG injection or the start of IVM. 2-cell embryos for immunostaining of PDH were collected at 44 h after HCG injection or the start of IVM. Immunostaining was performed by following a published protocol (Nagaraj *et al*, 2017). Embryos were fixed in 4% paraformaldehyde in PBS for 30 min at room temperature, permeabilized for 30 min in PBS with 0.4% Triton X-100 (PBST4), blocked in PBST4 with 3% bovine serum albumin (PBST4A) for 30 min and incubated with the desired primary antibody in PBST4A overnight at 4°C. The embryos were washed in PBST4 four times for 10 min each, blocked with PBST4A, and incubated with the appropriate secondary antibody and DAPI overnight at 4°C. The embryos were washed three times for 10 min each in PBST4, then deposited on glass slides and mounted in Vectashield (Vector Laboratories).

BrUTP incorporation assay was performed by following a published protocol (Suzuki *et al*, 2015) with a few modifications. 2-cell embryos were collected at 53 h after HCG injection or the start of IVM. BrUTP incorporation was performed by electroporation using the Super Electroporator NEPA 21 (NEPAGENE) as previously described (Dumeau *et al*, 2019). Embryos were washed in PBS and then transferred in a line on the glass chamber between electrodes filled with PBS containing 10 mM BrUTP (Merck). The poring pulse (voltage: 30 V, pulse length: 3 ms, pulse interval: 100 ms, number of pulses: 6, +) and the transfer pulse (voltage: 5 V, pulse length: 50 ms, pulse interval: 50 ms, number of pulses: 5, ±) were applied. The embryos were washed twice and cultured in KSOM for 1 h. Subsequently, fixation and immunostaining of embryos followed

 © 2023 The Authors

the protocol for histone marks or PDH described above. Anti-BrdU antibody (B8434, Merck) was used as the primary antibody.

For immunostaining of 5mC and 5hmC, 2-cell embryos were collected at 48 h after HCG injection or start of IVM. Also, NSN and SN oocytes were collected respectively from E12.5 gonad-derived oocytes after IVD and IVG for *in vitro*-derived samples, and from 6 dpp follicles and 4-week-old follicles for *in vivo*-derived samples. Immunostaining was performed by following a published protocol (Nakamura *et al*, 2007) with a few modifications. Embryos and oocytes were treated with 2 M HCl for 20 min and subsequently washed with 0.05% Tween 20 in PBS (PBST5) after permeabilization. The embryos and oocytes were blocked for 1 h in 1% bovine serum albumin and 0.05% Tween 20 in PBS (PBST5A), and then incubated overnight in anti-5mC and anti-5hmC antibodies (NA81-50UG, Millipore; 39069, Active Motif). The following day the embryos and oocytes were washed in PBST5 four times for 10 min each, blocked with PBST5A, and incubated with the appropriate secondary antibody and DAPI overnight at 4°C. Embryos and oocytes were washed three times for 10 min each in PBST5, then deposited on glass slides and mounted in Vectashield.

After immunostaining, images of 2-cell embryos were captured using a Leica TCS SP8 confocal microscope equipped with a sCMOS camera (Hamamatsu Orca Flash 4.0). Images of oocytes were captured by a Zeiss LSM780 confocal microscope equipped with a 40× water-immersion C-Apochromat 1.2 NA objective (Zeiss). Processing and quantification of images were performed using Fiji software (https://imagej.net/software/fiji/). For quantification of fluorescent signals, regions of nuclei and cytoplasm of 2-cell embryos and oocytes were manually selected using DAPI signals. The mean intensity of two nuclei (2-cell embryos) or a nucleus (oocytes) was subtracted with the mean intensity of cytoplasm of two blastomeres (2-cell embryos) or an oocyte. Quantified data was compiled and analyzed by GraphPad Prism 8 software using an unpaired two-tailed *t*-test. Data were considered significant if $P < 0.05$.

Isolation and sequencing of PGC and PGCLC transcriptomes

E12.5 PGCs and BVSC-iPSC-derived PGCLCs were used for their transcriptome analysis with bulk RNA-seq (Fig 5A). To prepare pooled PGCs, E12.5 female embryonic gonads were harvested from inbred C57BL/6J fetuses. PGCs, positive for both SSEA1 and integrin β3, were collected using a FACSAria III (BD Bioscience). D6 PGCLCs derived from a BVSC-iPSC line were also collected at day 8 of the culture by following the same protocol as the start of the IVD culture. Four replicates of respective PGC and PGCLC pools, consisting of 30,000 to 340,000 cells, were prepared from independent experiments. Total RNA was extracted using RNeasy Mini Kit (Qiagen) according to the manufacturer's protocol, including removal of genomic DNA. The RNA quality was determined by 2200 TapeStation (Agilent Technologies). All samples had a RIN value of greater than 8. Extracted RNA was prepared for sequencing using the TruSeq Stranded mRNA (Illumina) following the manufacturer's protocol. Briefly, 100 to 1,000 ng of total RNA was poly-A-enriched and reverse-transcribed into double-stranded cDNA. The cDNA samples were fragmented, end-repaired, and adenylated before ligation of TruSeq adapters. Fragments containing TruSeq adapters for multiplexing on both ends were selectively enriched with PCR. Sequencing was performed on an Illumina NovaSeq 6000 (Illumina), with sequencing depth of 20 million reads per sample and sequencing configuration of single-end 100 bp.

Isolation and sequencing of single oocyte transcriptomes

RNA sequencing libraries for single oocytes were prepared according to the Smartseq2 protocol (Picelli *et al*, 2014). For each experimental condition, 20–25 independent libraries were prepared. Firstly, each GRO and FGO derived from BVSC-iPSCs, E12.5 gonads, 6 dpp follicles and *in vivo* samples (Fig 5A) was dissociated from follicular somatic cells using 30G needles, followed by treatment with Accumax (Innovative Cell Technologies) for 5 min and by pipetting to remove somatic cells completely. The zona pellucida of each GRO and FGO was removed by acid Tyrode's solution (Merck) and samples were individually washed in PBS supplemented with 0.01% PVA (PBS-PVA) and lysed in the lysis buffer composed of 0.09% Triton-X 100, 2 U SUPERase IN RNase inhibitor (Invitrogen, AM2694), 2.5 μM Oligo-dT primer (Microsynth AG), dNTP mix (2.5 mM each, Promega), ERCC RNA Spike-In Mix (1: 3.2×10^7, Thermo Fischer Scientific 4456740) in individual tubes of a 8-well strip, then immediately frozen on the dry ice and kept at −80°C for longer storage. Next, sample lysate was denatured at 72°C for 3 min and quickly chilled on ice. The reverse transcription mix was composed of 100 U SuperScript II reverse transcriptase (Thermo Fischer Scientific, 18064014), 5 U SUPERase IN RNase inhibitor (Thermo Fischer Scientific, AM2696), 1× Superscript II first-strand buffer, 5 mM DTT (provided with SuperScript II reverse transcriptase), 1 M Betaine (Sigma, B0300-1VL), 6 mM $MgCl_2$, 1 μM template-switching oligos (TSOs; "AAGCAGTGGTATCAACGCAGAGTACATrGrG+G"; Exiqon) was added to obtain a total volume 10 μl and the reverse transcription was performed in PCR machine. Then PCR preamplification was performed in a total volume of 25 μl by adding 1× KAPA HiFi HotStart Ready Mix (KAPA Biosystems, KK2602), 0.1 μM ISPCR primers (Microsynth AG). The preamplification PCR cycle numbers were 15–16. Pre-amplified cDNA was purified with SPRI AMPure XP beads (Beckman, sample to beads ratio 1:1) and eluted in 15 μl Buffer EB (QIAGEN). 1 ng of pre-amplified cDNA was used for the tagmentation reaction (55°C, 7 min) using a homemade Tn5 tagmentation mix (1× TAPS-DMF buffer, Tn5-transposase) in total volume of 20 μl. The reaction was stopped by adding 5 μl of 0.2% SDS (Invitrogen, 24730020) and kept at 25°C for 7 min. Adapter-ligated fragment amplification was done using Nextera XT index kit (Illumina) in a total volume of 50 μl (1× Phusion HF Buffer, 2 U of Phusion High Fidelity DNA Polymerase (Thermo Fischer Scientific, F530L), dNTP mix (0.3 mM each, Promega)) with 9–10 cycles of PCR. Library was purified by SPRI AMPure XP beads (sample to beads ratio 1:1) and eluted in 12 μl Buffer EB. Sequencing was performed on an Illumina HiSeq 2500 machine with single-end 75-bp read length (Illumina).

Transcriptional analysis

Alignment of RNA-Seq data

RNA-Seq datasets were aligned to a custom genome containing the *Mus musculus* genome assembly (GRCm38/mm10 Dec. 2011) and ERCC92 sequences using STAR (Dobin *et al*, 2013) with parameters "-outFilterMultimapNmax 300-outMultimapperOrder Random-outSAMmultNmax 1-alignIntronMin 20-alignIntronMax 1000000," allowing multimappers with up to 300 matches in the genome and choosing positions for multimappers randomly (Dataset EV4).

Selection of exonic and promoter regions for genes and quantification of gene expression

A random transcript isoform for each gene was chosen from Bioconductor annotation package TxDb.Mmusculus.UCSC.mm10.knownGene (version 3.2.2) and promoter regions were constructed as regions 1.5 kb upstream and downstream from transcription start site. Any overlaps between promoters were removed by choosing a random promoter among promoters which overlap. Resulting sets of non-overlapping promoter and exonic genomic regions for the random transcript isoforms for each gene were used in further analyses and referred as "all genes" in the text.

Expression quantification for selected exonic genomic regions was done using QuasR R package (Gaidatzis *et al*, 2015) selecting only uniquely mapped reads (mapqMin = 255). RPKM values for genes were calculated by normalizing exonic read counts to total exonic length of each gene and total number of reads mapping to all exonic regions in each library. RPKM values were log2 transformed using formula log2(RPKM + psc) − log2(psc) where pseudo-count psc was set to 0.1.

Principal component analysis of PGCLC and PGC samples

To compare RNA-Seq data for day 6 PGCLC and E12.5 female PGCs generated for this study with previously published datasets we downloaded from the GEO repository RNA-seq datasets for E12.5 female PGC (GSE87644; Ohta *et al*, 2017) and for day 6 PGCLC (GSE67259; Sasaki *et al*, 2015). Relative to mean expression values for each study separately were calculated by subtracting average log2(RPKM) values across samples. Using relative expression values for samples generated for this study and for previously published datasets the Principal Component Analysis (PCA) was done using R function *prcomp* (Fig EV4A).

Differential expression analysis of PGCLC and PGC samples

R package *edgeR* (McCarthy *et al*, 2012) was used to study gene expression differences between day 6 PGCLC and E12.5 female PGCs both for previously published and generated for this study datasets. Generalized Linear Model was fit using cell type (PGC or PGCLC) as covariates. Statistical significance was estimated using log-likelihood tests and the Benjamini–Hochberg method was used to correct for multiple testing.

Estimating of gene expression responses to stages of in vitro *development*

To minimize potential biases from outlier samples we selected samples based on oocyte sizes for further analysis (Dataset EV4). Genes with CPM higher than one in at least three samples were used for the analysis. Samples for GROs and FGOs were separated, and for each stage, the gene expression responses were estimated by fitting generalized linear model with *edgeR* using model matrix encoding stages of *in vitro* or *in vivo* development underwent by each sample, i.e. IVP for oocytes in cohorts GRO^{iPSC}, FGO^{iPSC} or *in_vivo* for oocytes in cohorts GRO^{PGC}, FGO^{PGC}, FGO$^{6dpp\ Oo}$, GRO$^{in\ vivo}$ and FGO$^{in\ vivo}$; IVD for oocytes in cohorts GRO^{iPSC}, FGO^{iPSC}, GRO^{PGC}, FGO^{PGC} or *in_vivo* for cohorts FGO$^{6dpp\ Oo}$, GRO$^{in\ vivo}$ and FGO$^{in\ vivo}$; IVG for cohorts FGO^{iPSC}, FGO^{PGC}, FGO$^{6dpp\ Oo}$ and *in_vivo* for cohort FGO$^{in\ vivo}$. The expression responses which in this model are represented by coefficients for IVP, IVD and IVG for each gene were fit and statistical significance was estimated using log-likelihood test and corrected for multiple testing using the Benjamini–Hochberg method.

Gene ontology enrichment analysis

Enrichment analysis for Gene Ontology (GO) terms was done using R package *topGO* (version 2.48.0; Alexa & Rahnenfuhrer, 2022) with parameters *method="weight01"* and *statistic="fisher"* extracting GO gene annotation from the Bioconductor Annotation Package *org.Mm.eg.db* (version 3.15.0; Carlson, 2022).

Visualization of GO enrichments was done by calculation of pairwise Jaccard distances between significant GO terms based on intersections and unions of significantly affected gene sets having corresponding GO term annotations. After pairwise Jaccard distances between GO terms were calculated we applied multidimensional scaling (MDS) using R function *cmdscale* and represented GO terms on a 2D plot where size was scaled by obs./Exp. ratio, color was chosen to reflect statistical significance and relative position reflects similarities in gene sets (Fig EV4D and E).

Investigation of promoter features of affected genes

Previously selected promoter regions were classified into CGI and non-CGI promoters by calculating observed versus expected ratios of CpG dinucleotides and fitting Gaussian mixture model with two components.

Enrichment analysis for transcription factor motifs at promoter regions was performed using function *calcBinnedMotifEnrR* in R package *monaLisa* (Machlab *et al*, 2022) for which genes were binned according to combination of type of expression response (up-regulated or down-regulated) and type of expression dynamics *in vivo* (LS, UP, DN and HS groups in Fig 5C) for each stage of *in vitro* development separately (IVP@GRO, IVD@GRO, IVP@FGO, IVD@FGO and IVG@FGO). As background set, we used option "otherBins" for *calcBinnedMotifEnrR* function. We used a set of position weight matrices (PWMs) from the *JASPAR2020* Bioconductor R package for vertebrates which encompasses 746 PWMs.

Alignment and analysis of ChIP-seq data

Previously published ChIP-Seq or Cut&Run samples for PGCLC as well as for different stages of *in vivo* development were downloaded from the GEO repository (see Dataset EV4 for list of published datasets and accession IDs used in this study). The quality of the data was assessed using FastQC (v0.11.8) and adapters were trimmed using TrimGalore (v0.6.2) (Krueger, 2015). All datasets were aligned to mm10 mouse genome using STAR with parameters "-alignIntronMin 1-alignIntronMax 1-alignEndsType EndToEnd-alignMatesGapMax 1000-outFilterMatchNminOverLread 0.85-outFilterMultimapNmax 300-outMultimapperOrder Random-outSAMmultNmax 1" and possible PCR duplicates were removed using samtools (Li *et al*, 2009).

Quantification for previously selected promoter regions was done using QuasR R package (Gaidatzis *et al*, 2015) selecting only uniquely mapped reads (mapqMin = 255). Log2(CPM) values calculated by adding a pseudo-count of 16 to promoter read counts, normalizing to total number of mapped reads in each sample and log2 transformation.

 © 2023 The Authors

Statistical analysis

Statistical tests were performed to analyze oocyte/follicle sizes, developmental ratio of embryos and immunostaining data using GraphPad Prism 8 software (Figs 1, 3, 4 and EV3). Differences between two groups were determined by unpaired two-tailed *t*-tests. *P*-values < 0.05 were considered statistically significant.

No statistical methods were applied to pre-determine cohort sizes in single-cell RNA-seq experiment. *edgeR* was used to perform differential expression analysis of the RNA-seq data. To estimate gene expression responses, a generalized linear model was fit as described above. Statistical significance was estimated using log-likelihood tests and the Benjamini–Hochberg method was used to correct for multiple testing. Gene expressions with FDR $\leq 5\%$ and $|\log2(\text{Fold-change})| \geq 2$ were considered statistically significant.

Statistical analysis of gene enrichment for Figs 5E and F, and EV4 was done using the χ^2 test implemented in the R function *chisq.test*. Enrichments were calculated as log2-ratios between observed and expected counts after adding pseudocount of 1. Enrichments with *P*-value $\leq 1\%$ were considered statistically significant.

Statistical analysis of differences in histone PTMs for Fig 6A was done using the two-sided Mann–Whitney test implemented in the R function *wilcox.test* and the Benjamini–Hochberg method was used to correct for multiple testing. Differences with adj.*P*-value $\leq 0.1\%$ were considered statistically significant.

Statistical analysis of GO term enrichments was done using Fisher's test in the R package topGO. Enrichments of GO terms were calculated as ratios between observed and expected numbers of genes. Figure EV4D consists of GO terms with enrichments higher than 2 and *P*-values lower than 0.01%. Figure EV4E consists of enrichments higher than 1.1 and *P*-values lower than 0.1%.

Statistical analysis of transcription factor motif enrichments was done using one-sided Fisher's test with Benjamini–Hochberg multiple testing correction method in the R package *monaLisa*. Enrichments with FDR $\leq 1\%$ were considered statistically significant.

Data availability

RNA-seq datasets produced in this study were deposited to Gene Expression Omnibus (GEO): GSE223479 (http://www.ncbi.nlm.nih.gov/geo/query/acc.cgi?acc=GSE223479).

Expanded View for this article is available online.

Acknowledgements
We are grateful for the advice on the *in vitro* culture protocol provided by Orie Hikabe and Katsuhiko Hayashi (Osaka University, Japan). A BVSC-iPSC line was kindly provided by Katsuhiko Hayashi. We thank the ETH Phenomics Center (ETH Zurich), Flow Cytometry Core Facility (ETH Zurich), Scientific Center for Optical and Electron Microscopy (ETH Zurich), and Functional Genomics Center Zurich for their technical assistance. We thank Sebastien Smallwood and colleagues (Functional Genomics Platform FMI, Basel) for technical assistance. This study was supported by an EMBO Short-Term Fellowship (ASTF 207-2016) and Swiss National Science Foundation (grant 31003A_152814/1). This research has received funding from the Novartis Research Foundation and the European Research Council (ERC) under the European Union's Horizon 2020 research and innovation program (grant agreement ERC-AdG 695288—Totipotency).

Author contributions

Eishi Aizawa: Conceptualization; data curation; formal analysis; funding acquisition; investigation; methodology; writing – original draft. **Evgeniy A Ozonov:** Conceptualization; data curation; software; formal analysis; investigation; methodology; writing – original draft. **Yumiko K Kawamura:** Resources; data curation; formal analysis; investigation; methodology. **Charles-Etienne Dumeau:** Resources; data curation; formal analysis; investigation; methodology. **So Nagaoka:** Resources; supervision; methodology. **Tomoya S Kitajima:** Resources; supervision; methodology. **Mitinori Saitou:** Conceptualization; resources; supervision; investigation; project administration; writing – review and editing. **Antoine HFM Peters:** Conceptualization; formal analysis; funding acquisition; investigation; writing – original draft; project administration. **Anton Wutz:** Conceptualization; formal analysis; funding acquisition; investigation; writing – original draft; project administration.

Disclosure and competing interests statement

The authors declare that they have no conflict of interest.

References

Aizawa E, Dumeau C-E, Freimann R, Di Minin G, Wutz A (2020) Polyploidy of semi-cloned embryos generated from parthenogenetic haploid embryonic stem cells. *PLoS ONE* 15: e0233072

Alexa A, Rahnenfuhrer J (2022) topGO: enrichment analysis for gene ontology

Amouroux R, Nashun B, Shirane K, Nakagawa S, Hill PW, D'Souza Z, Nakayama M, Matsuda M, Turp A, Ndjetehe E *et al* (2016) De novo DNA methylation drives 5hmC accumulation in mouse zygotes. *Nat Cell Biol* 18: 225–233

Blackledge NP, Klose RJ (2021) The molecular principles of gene regulation by Polycomb repressive complexes. *Nat Rev Mol Cell Biol* 22: 815–833

Carlson M (2022) org.Mm.eg.db: genome wide annotation for mouse

Chen Z, Yin Q, Inoue A, Zhang C, Zhang Y (2019) Allelic H3K27me3 to allelic DNA methylation switch maintains noncanonical imprinting in extraembryonic cells. *Sci Adv* 5: eaay7246

Ciccone DN, Su H, Hevi S, Gay F, Lei H, Bajko J, Xu G, Li E, Chen T (2009) KDM1B is a histone H3K4 demethylase required to establish maternal genomic imprints. *Nature* 461: 415–418

Cyranoski D, Contreras JL, Carrington VT (2023) Intellectual property and assisted reproductive technology. *Nat Biotechnol* 41: 14–20

Dobin A, Davis CA, Schlesinger F, Drenkow J, Zaleski C, Jha S, Batut P, Chaisson M, Gingeras TR (2013) STAR: ultrafast universal RNA-seq aligner. *Bioinformatics* 29: 15–21

Du W, Dong Q, Zhang Z, Liu B, Zhou T, Xu RM, Wang H, Zhu B, Li Y (2019) Stella protein facilitates DNA demethylation by disrupting the chromatin association of the RING finger-type E3 ubiquitin ligase UHRF1. *J Biol Chem* 294: 8907–8917

Du Z, Zheng H, Kawamura YK, Zhang K, Gassler J, Powell S, Xu Q, Lin Z, Xu K, Zhou Q *et al* (2020) Polycomb group proteins regulate chromatin architecture in mouse oocytes and early embryos. *Mol Cell* 77: 825–839

Dumeau CE, Monfort A, Kissling L, Swarts DC, Jinek M, Wutz A (2019) Introducing gene deletions by mouse zygote electroporation of Cas12a/Cpf1. *Transgenic Res* 28: 525–535

Eguizabal C, Shovlin TC, Durcova-Hills G, Surani A, McLaren A (2009) Generation of primordial germ cells from pluripotent stem cells. *Differentiation* 78: 116–123

© 2023 The Authors

Ellegren H, Galtier N (2016) Determinants of genetic diversity. *Nat Rev Genet* 17: 422–433

Fornes O, Castro-Mondragon JA, Khan A, van der Lee R, Zhang X, Richmond PA, Modi BP, Correard S, Gheorghe M, Baranasic D *et al* (2020) JASPAR 2020: update of the open-access database of transcription factor binding profiles. *Nucleic Acids Res* 48: D87–D92

Frost ER, Taylor G, Baker MA, Lovell-Badge R, Sutherland JM (2021) Establishing and maintaining fertility: the importance of cell cycle arrest. *Genes Dev* 35: 619–634

Gaidatzis D, Lerch A, Hahne F, Stadler MB (2015) QuasR: quantification and annotation of short reads in R. *Bioinformatics* 31: 1130–1132

Hajkova P, Ancelin K, Waldmann T, Lacoste N, Lange UC, Cesari F, Lee C, Almouzni G, Schneider R, Surani MA (2008) Chromatin dynamics during epigenetic reprogramming in the mouse germ line. *Nature* 452: 877–881

Hamada N, Hamazaki N, Shimamoto S, Hikabe O, Nagamatsu G, Takada Y, Kato K, Hayashi K (2020) Germ cell-intrinsic effects of sex chromosomes on early oocyte differentiation in mice. *PLoS Genet* 16: e1008676

Hamazaki N, Kyogoku H, Araki H, Miura F, Horikawa C, Hamada N, Shimamoto S, Hikabe O, Nakashima K, Kitajima TS *et al* (2021) Reconstitution of the oocyte transcriptional network with transcription factors. *Nature* 589: 264–269

Hanna CW, Demond H, Kelsey G (2018a) Epigenetic regulation in development: is the mouse a good model for the human? *Hum Reprod Update* 24: 556–576

Hanna CW, Taudt A, Huang J, Gahurova L, Kranz A, Andrews S, Dean W, Stewart AF, Colomé-Tatché M, Kelsey G (2018b) MLL2 conveys transcription-independent H3K4 trimethylation in oocytes. *Nat Struct Mol Biol* 25: 73–82

Hanna CW, Pérez-Palacios R, Gahurova L, Schubert M, Krueger F, Biggins L, Andrews S, Colomé-Tatché M, Bourc'his D, Dean W *et al* (2019) Endogenous retroviral insertions drive non-canonical imprinting in extra-embryonic tissues. *Genome Biol* 20: 225

Hayashi K, Saitou M (2013) Generation of eggs from mouse embryonic stem cells and induced pluripotent stem cells. *Nat Protoc* 8: 1513–1524

Hayashi K, Ohta H, Kurimoto K, Aramaki S, Saitou M (2011) Reconstitution of the mouse germ cell specification pathway in culture by pluripotent stem cells. *Cell* 146: 519–532

Hayashi K, Ogushi S, Kurimoto K, Shimamoto S, Ohta H, Saitou M (2012) Offspring from oocytes derived from in vitro primordial germ cell-like cells in mice. *Science* 338: 971–975

Hayashi K, Hikabe O, Obata Y, Hirao Y (2017) Reconstitution of mouse oogenesis in a dish from pluripotent stem cells. *Nat Protoc* 12: 1733–1744

Hayashi M, Zywitza V, Naitou Y, Hamazaki N, Goeritz F, Hermes R, Holtze S, Lazzari G, Galli C, Stejskal J *et al* (2022) Robust induction of primordial germ cells of white rhinoceros on the brink of extinction. *Sci Adv* 8: eabp9683

Hikabe O, Hamazaki N, Nagamatsu G, Obata Y, Hirao Y, Hamada N, Shimamoto S, Imamura T, Nakashima K, Saitou M *et al* (2016) Reconstitution in vitro of the entire cycle of the mouse female germ line. *Nature* 539: 299–303

Hu M, Yeh YH, Munakata Y, Abe H, Sakashita A, Maezawa S, Vidal M, Koseki H, Hunter N, Schultz RM *et al* (2022) PRC1-mediated epigenetic programming is required to generate the ovarian reserve. *Nat Commun* 13: 4510

Hwang YS, Suzuki S, Seita Y, Ito J, Sakata Y, Aso H, Sato K, Hermann BP, Sasaki K (2020) Reconstitution of prospermatogonial specification in vitro from human induced pluripotent stem cells. *Nat Commun* 11: 5656

Inoue A, Jiang L, Lu F, Suzuki T, Zhang Y (2017a) Maternal H3K27me3 controls DNA methylation-independent imprinting. *Nature* 547: 419–424

Inoue A, Jiang L, Lu F, Zhang Y (2017b) Genomic imprinting of Xist by maternal H3K27me3. *Genes Dev* 31: 1927–1932

Irie N, Weinberger L, Tang WW, Kobayashi T, Viukov S, Manor YS, Dietmann S, Hanna JH, Surani MA (2015) SOX17 is a critical specifier of human primordial germ cell fate. *Cell* 160: 253–268

Kaneda M, Okano M, Hata K, Sado T, Tsujimoto N, Li E, Sasaki H (2004) Essential role for de novo DNA methyltransferase Dnmt3a in paternal and maternal imprinting. *Nature* 429: 900–903

Kasinath V, Beck C, Sauer P, Poepsel S, Kosmatka J, Faini M, Toso D, Aebersold R, Nogales E (2021) JARID2 and AEBP2 regulate PRC2 in the presence of H2AK119ub1 and other histone modifications. *Science* 371: eabc3393

Kawabata Y, Kamio A, Jincho Y, Sakashita A, Takashima T, Kobayashi H, Matsui Y, Kono T (2019) Sex-specific histone modifications in mouse fetal and neonatal germ cells. *Epigenomics* 11: 543–561

Kishigami S, Wakayama T (2007) Efficient strontium-induced activation of mouse oocytes in standard culture media by chelating calcium. *J Reprod Dev* 53: 1207–1215

Krueger F (2015) Trim Galore: a wrapper tool around Cutadapt and FastQC to consistently apply quality and adapter trimming to FastQ files

Kurimoto K, Yabuta Y, Hayashi K, Ohta H, Kiyonari H, Mitani T, Moritoki Y, Kohri K, Kimura H, Yamamoto T *et al* (2015) Quantitative dynamics of chromatin remodeling during germ cell specification from mouse embryonic stem cells. *Cell Stem Cell* 16: 517–532

Li H, Handsaker B, Wysoker A, Fennell T, Ruan J, Homer N, Marth G, Abecasis G, Durbin R, Proc GPD (2009) The sequence alignment/map format and SAMtools. *Bioinformatics* 25: 2078–2079

Li Y, Zhang Z, Chen J, Liu W, Lai W, Liu B, Li X, Liu L, Xu S, Dong Q *et al* (2018) Stella safeguards the oocyte methylome by preventing de novo methylation mediated by DNMT1. *Nature* 564: 136–140

Machlab D, Burger L, Soneson C, Rijli FM, Schubeler D, Stadler MB (2022) monaLisa: an R/Bioconductor package for identifying regulatory motifs. *Bioinformatics* 38: 2624–2625

Matoba S, Kozuka C, Miura K, Inoue K, Kumon M, Hayashi R, Ohhata T, Ogura A, Inoue A (2022) Noncanonical imprinting sustains embryonic development and restrains placental overgrowth. *Genes Dev* 36: 483–494

McCarthy DJ, Chen Y, Smyth GK (2012) Differential expression analysis of multifactor RNA-Seq experiments with respect to biological variation. *Nucleic Acids Res* 40: 4288–4297

Mei H, Kozuka C, Hayashi R, Kumon M, Koseki H, Inoue A (2021) H2AK119ub1 guides maternal inheritance and zygotic deposition of H3K27me3 in mouse embryos. *Nat Genet* 53: 539–550

Morohaku K, Tanimoto R, Sasaki K, Kawahara-Miki R, Kono T, Hayashi K, Hirao Y, Obata Y (2016) Complete in vitro generation of fertile oocytes from mouse primordial germ cells. *Proc Natl Acad Sci USA* 113: 9021–9026

Morohaku K, Hirao Y, Obata Y (2017) Development of fertile mouse oocytes from mitotic germ cells in vitro. *Nat Protoc* 12: 1817–1829

Murakami K, Hamazaki N, Hamada N, Nagamatsu G, Okamoto I, Ohta H, Nosaka Y, Ishikura Y, Kitajima TS, Semba Y *et al* (2023) Generation of functional oocytes from male mice in vitro. *Nature* 615: 900–906

Nagamatsu G, Shimamoto S, Hamazaki N, Nishimura Y, Hayashi K (2019) Mechanical stress accompanied with nuclear rotation is involved in the dormant state of mouse oocytes. *Sci Adv* 5: eaav9960

Nagaraj R, Sharpley MS, Chi F, Braas D, Zhou Y, Kim R, Clark AT, Banerjee U (2017) Nuclear localization of mitochondrial TCA cycle enzymes as a critical step in mammalian zygotic genome activation. *Cell* 168: 210–223

© 2023 The Authors

Nakamura T, Arai Y, Umehara H, Masuhara M, Kimura T, Taniguchi H, Sekimoto T, Ikawa M, Yoneda Y, Okabe M *et al* (2007) PGC7/Stella protects against DNA demethylation in early embryogenesis. *Nat Cell Biol* 9: 64 – 71

Nakamura T, Liu YJ, Nakashima H, Umehara H, Inoue K, Matoba S, Tachibana M, Ogura A, Shinkai Y, Nakano T (2012) PGC7 binds histone H3K9me2 to protect against conversion of 5mC to 5hmC in early embryos. *Nature* 486: 415 – 419

Nakatani T, Yamagata K, Kimura T, Oda M, Nakashima H, Hori M, Sekita Y, Arakawa T, Nakamura T, Nakano T (2015) Stella preserves maternal chromosome integrity by inhibiting 5hmC-induced γH2AX accumulation. *EMBO Rep* 16: 582 – 589

Nayernia K, Nolte J, Michelmann HW, Lee JH, Rathsack K, Drusenheimer N, Dev A, Wulf G, Ehrmann IE, Elliott DJ *et al* (2006) In vitro-differentiated embryonic stem cells give rise to male gametes that can generate offspring mice. *Dev Cell* 11: 125 – 132

Niedzialkowska E, Liu L, Kuscu C, Mayo Z, Minor W, Strahl BD, Adli M, Stukenberg PT (2022) Tip60 acetylation of histone H3K4 temporally controls chromosome passenger complex localization. *Mol Biol Cell* 33: br15

Niu W, Spradling AC (2022) Mouse oocytes develop in cysts with the help of nurse cells. *Cell* 185: 2576 – 2590

O'Connell JM, Pepling ME (2021) Primordial follicle formation – some assembly required. *Curr Opin Endocr Metab Res* 18: 118 – 127

Odor DL, Blandau RJ (1971) Organ cultures of fetal mouse ovaries. I. Light microscopic structure. *Am J Anat* 131: 387 – 414

Ohinata Y, Sano M, Shigeta M, Yamanaka K, Saitou M (2008) A comprehensive, non-invasive visualization of primordial germ cell development in mice by the Prdm1-mVenus and Dppa3-ECFP double transgenic reporter. *Reproduction* 136: 503 – 514

Ohinata Y, Ohta H, Shigeta M, Yamanaka K, Wakayama T, Saitou M (2009) A signaling principle for the specification of the germ cell lineage in mice. *Cell* 137: 571 – 584

Ohta H, Kurimoto K, Okamoto I, Nakamura T, Yabuta Y, Miyauchi H, Yamamoto T, Okuno Y, Hagiwara M, Shirane K *et al* (2017) In vitro expansion of mouse primordial germ cell-like cells recapitulates an epigenetic blank slate. *EMBO J* 36: 1888 – 1907

Ooi SK, Qiu C, Bernstein E, Li K, Jia D, Yang Z, Erdjument-Bromage H, Tempst P, Lin SP, Allis CD *et al* (2007) DNMT3L connects unmethylated lysine 4 of histone H3 to de novo methylation of DNA. *Nature* 448: 714 – 717

Picelli S, Faridani OR, Bjorklund AK, Winberg G, Sagasser S, Sandberg R (2014) Full-length RNA-seq from single cells using Smart-seq2. *Nat Protoc* 9: 171 – 181

Posfai E, Kunzmann R, Brochard V, Salvaing J, Cabuy E, Roloff TC, Liu Z, Tardat M, van Lohuizen M, Vidal M *et al* (2012) Polycomb function during oogenesis is required for mouse embryonic development. *Genes Dev* 26: 920 – 932

Qing T, Shi Y, Qin H, Ye X, Wei W, Liu H, Ding M, Deng H (2007) Induction of oocyte-like cells from mouse embryonic stem cells by co-culture with ovarian granulosa cells. *Differentiation* 75: 902 – 911

Saitou M, Hayashi K (2021) Mammalian in vitro gametogenesis. *Science* 374: eaaz6830

Saksouk N, Barth TK, Ziegler-Birling C, Olova N, Nowak A, Rey E, Mateos-Langerak J, Urbach S, Reik W, Torres-Padilla ME *et al* (2014) Redundant mechanisms to form silent chromatin at pericentromeric regions rely on BEND3 and DNA methylation. *Mol Cell* 56: 580 – 594

Sasaki K, Yokobayashi S, Nakamura T, Okamoto I, Yabuta Y, Kurimoto K, Ohta H, Moritoki Y, Iwatani C, Tsuchiya H *et al* (2015) Robust in vitro induction of human germ cell fate from pluripotent stem cells. *Cell Stem Cell* 17: 178 – 194

Seisenberger S, Andrews S, Krueger F, Arand J, Walter J, Santos F, Popp C, Thienpont B, Dean W, Reik W (2012) The dynamics of genome-wide DNA methylation reprogramming in mouse primordial germ cells. *Mol Cell* 48: 849 – 862

Seki Y, Hayashi K, Itoh K, Mizugaki M, Saitou M, Matsui Y (2005) Extensive and orderly reprogramming of genome-wide chromatin modifications associated with specification and early development of germ cells in mice. *Dev Biol* 278: 440 – 458

Seki Y, Yamaji M, Yabuta Y, Sano M, Shigeta M, Matsui Y, Saga Y, Tachibana M, Shinkai Y, Saitou M (2007) Cellular dynamics associated with the genome-wide epigenetic reprogramming in migrating primordial germ cells in mice. *Development* 134: 2627 – 2638

Sendžikaitė G, Kelsey G (2019) The role and mechanisms of DNA methylation in the oocyte. *Essays Biochem* 63: 691 – 705

Severino J, Bauer M, Mattimoe T, Arecco N, Cozzuto L, Lorden P, Hamada N, Nosaka Y, Nagaoka SI, Audergon P *et al* (2022) Controlled X-chromosome dynamics defines meiotic potential of female mouse in vitro germ cells. *EMBO J* 41: e109457

Shimamoto S, Nishimura Y, Nagamatsu G, Hamada N, Kita H, Hikabe O, Hamazaki N, Hayashi K (2019) Hypoxia induces the dormant state in oocytes through expression of Foxo3. *Proc Natl Acad Sci USA* 116: 12321 – 12326

Shin SW, Vogt EJ, Jimenez-Movilla M, Baibakov B, Dean J (2017) Cytoplasmic cleavage of DPPA3 is required for intracellular trafficking and cleavage-stage development in mice. *Nat Commun* 8: 1643

Shirane K, Toh H, Kobayashi H, Miura F, Chiba H, Ito T, Kono T, Sasaki H (2013) Mouse oocyte methylomes at base resolution reveal genome-wide accumulation of non-CpG methylation and role of DNA methyltransferases. *PLoS Genet* 9: e1003439

Smallwood SA, Tomizawa S, Krueger F, Ruf N, Carli N, Segonds-Pichon A, Sato S, Hata K, Andrews SR, Kelsey G (2011) Dynamic CpG Island methylation landscape in oocytes and preimplantation embryos. *Nat Genet* 43: 811 – 814

Stäubli A, Peters AH (2021) Mechanisms of maternal intergenerational epigenetic inheritance. *Curr Opin Genet Dev* 67: 151 – 162

Stewart KR, Veselovska L, Kim J, Huang J, Saadeh H, Tomizawa S, Smallwood SA, Chen T, Kelsey G (2015) Dynamic changes in histone modifications precede de novo DNA methylation in oocytes. *Genes Dev* 29: 2449 – 2462

Suzuki S, Nozawa Y, Tsukamoto S, Kaneko T, Manabe I, Imai H, Minami N (2015) CHD1 acts via the Hmgpi pathway to regulate mouse early embryogenesis. *Development* 142: 2375 – 2384

Toyooka Y, Tsunekawa N, Akasu R, Noce T (2003) Embryonic stem cells can form germ cells in vitro. *Proc Natl Acad Sci USA* 100: 11457 – 11462

Tucci V, Isles AR, Kelsey G, Ferguson-Smith AC (2019) Genomic imprinting and physiological processes in mammals. *Cell* 176: 952 – 965

Yamashiro C, Sasaki K, Yabuta Y, Kojima Y, Nakamura T, Okamoto I, Yokobayashi S, Murase Y, Ishikura Y, Shirane K *et al* (2018) Generation of human oogonia from induced pluripotent stem cells in vitro. *Science* 362: 356 – 360

Yokobayashi S, Liang CY, Kohler H, Nestorov P, Liu Z, Vidal M, van Lohuizen M, Roloff TC, Peters AH (2013) PRC1 coordinates timing of sexual differentiation of female primordial germ cells. *Nature* 495: 236 – 240

Yoshino T, Suzuki T, Nagamatsu G, Yabukami H, Ikegaya M, Kishima M, Kita H, Imamura T, Nakashima K, Nishinakamura R *et al* (2021) Generation of ovarian follicles from mouse pluripotent stem cells. *Science* 373: eabe0237

Yusa K, Zhou L, Li MA, Bradley A, Craig NL (2011) A hyperactive piggyBac transposase for mammalian applications. *Proc Natl Acad Sci USA* 108: 1531 – 1536

© 2023 The Authors

Zhang Y, Jurkowska R, Soeroes S, Rajavelu A, Dhayalan A, Bock I, Rathert P, Brandt O, Reinhardt R, Fischle W *et al* (2010) Chromatin methylation activity of Dnmt3a and Dnmt3a/3L is guided by interaction of the ADD domain with the histone H3 tail. *Nucleic Acids Res* 38: 4246–4253

Zhang C, Wang M, Li Y, Zhang Y (2022a) Profiling and functional characterization of maternal mRNA translation during mouse maternal-to-zygotic transition. *Sci Adv* 8: eabj3967

Zhang S, Tao W, Han JJ (2022b) 3D chromatin structure changes during spermatogenesis and oogenesis. *Comput Struct Biotechnol J* 20: 2434–2441

License: This is an open access article under the terms of the Creative Commons Attribution License, which permits use, distribution and reproduction in any medium, provided the original work is properly cited.

Expanded View Figures

Figure EV1. Development of PSC-derived oocytes during IVP and IVD, and assessment of FBS.

A Differentiation of BVSC-ESCs to PGCLCs. Expression of Blimp1-Venus and Stella-ECFP as well as bright-field images are shown from day 0 to 8 of the culture. Blimp1-Venus and Stella-ECFP started their expression at days 4 and 6, respectively. Scale bar, 100 μm.

B Representative development of a BVSC-ESC-derived rOvary from day 10 to 31 of the culture. Stella-ECFP expression transiently decreased around day 17, followed by emergence of round oocytes expressing Stella-ECFP. Scale bar, 100 μm.

C A representative morphology of BVSC-iPSC-derived follicles at day 31, and *in vivo*-derived follicles at 3, 6 and 9 dpp. Stella-ECFP expression (cyan) was merged with the bright-field image of BVSC-iPSC-derived follicles. Scale bar, 50 μm.

D Representative morphology of rOvaries at day 31 of the culture. Nine FBS and one serum replacement (a–j) were tested to assess follicle formation by the IVD culture. Bright-field images merged with Stella-ECFP (cyan) are shown. The commercial companies and catalog numbers of respective FBS and a serum replacement are as follows: a, Life Technologies, A3161001; b, Life Technologies, A3160801; c, GE Healthcare, SH30071.02; d, GE Healthcare, SV30160.02; e, Sigma, F0926; f, Life Technologies, A3160901; g, PAN Biotech, P30-1702; h, GE Healthcare, SH30084.02; i, Life Technologies, 10828-028; j, Equitech-Bio, SBSU30-0500. Scale bar, 200 μm.

E At day 31 of the culture, each rOvary was mechanically dissected by 30G needles to isolate single secondary follicles. The ratio of successful follicle isolation was calculated based on the number of isolated single secondary follicles divided by the number of secondary follicles attempted to isolate, which are shown in brackets. The data is based on two independent experiments.

Source data are available online for this figure.

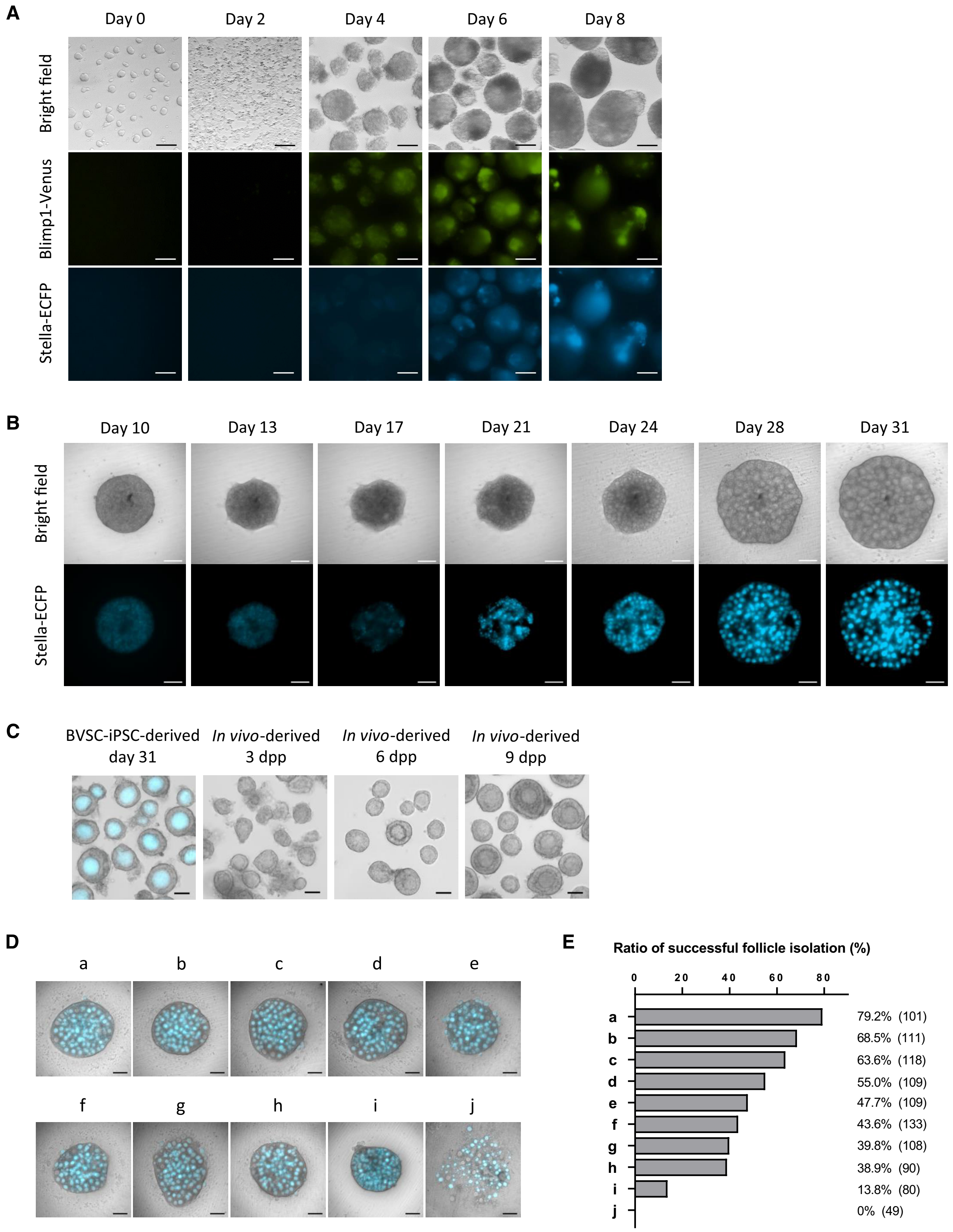

Figure EV1.

© 2023 The Authors

Figure EV2. Post-IVD development of PSC-derived oocytes and abnormal development of rOvaries/oocytes.

A IVG of BVSC-iPSC-derived follicles isolated from a rOvary. Bright-field images were merged with Stella-ECFP expression (cyan). Cultured follicles were treated with collagenase at day 33 of the culture. Some follicles expansively developed at day 43. Scale bar, 200 μm.

B Size of developed follicles at day 44 of the culture. The largest diameter of BVSC-iPSC-derived follicles in two conditions was measured; 1–3 follicles (left) and 4–10 follicles (right) placed on the same transwell membrane during IVG. Brackets represent number of counted follicles. The data of 4–10 follicles is identical to the data of iPSC-derived follicle in Fig 3A.

C MII oocytes containing first polar bodies derived from BVSC-ESCs at day 45 of the culture. Oocytes without Stella-ECFP expression were presumably derived from PGCs in E12.5 gonads contaminated at day 8 of the culture. Scale bar, 100 μm.

D Development of outgrowths from rOvaries. Bright-field images were merged with Stella-ECFP (left, cyan) and CAG-GFP (right, green). PGCLCs positive for both Blimp1-Venus and Stella-ECFP or for both SSEA1 and integrin-β3 were used for BVSC-iPSC-derived (left) or GFP-ESC-derived (right) rOvary respectively. Scale bar, 200 μm.

E Genotyping of outgrowths sampled from different six BVSC-iPSC-derived rOvaries. Arrowheads indicate amplified fragments targeting Blimp1-Venus, Stella-ECFP and endogenous Prdm14, respectively. All six outgrowths carried Blimp1-Venus and Stella-ECFP reporters, indicating the outgrowths were derived from BVSC-iPSCs.

F BVSC-iPSC-derived oocytes with cells on the inner side of zona pellucida, harvested at day 45 of the culture. The parts in white square (left) were enlarged to right images. While Stella-ECFP was detected in the ooplasm and polar body, the contaminating cells were negative for Stella-ECFP. Scale bar, 50 μm.

G A BVSC-iPSC-derived 2-cell embryo with cells on the inner side of zona pellucida, harvested 1 day after PA of the oocyte at day 45 of the culture. The part in a black square (left top) was enlarged to an image (right top). The asterisk indicates cells on the inner side of zona pellucida. Arrowheads indicate branched zona pellucida. Scale bar, 20 μm.

H Preimplantation development of BVSC-iPSC-derived oocytes after PA. Bright-field images were merged with Stella-ECFP expression (cyan). Embryos without Stella-ECFP expression presumably developed from E12.5 PGCs, which were possibly mixed with gonadal somatic cells for co-culture at day 8. Arrowheads indicate morulae. Scale bar, 50 μm.

Source data are available online for this figure.

© 2023 The Authors

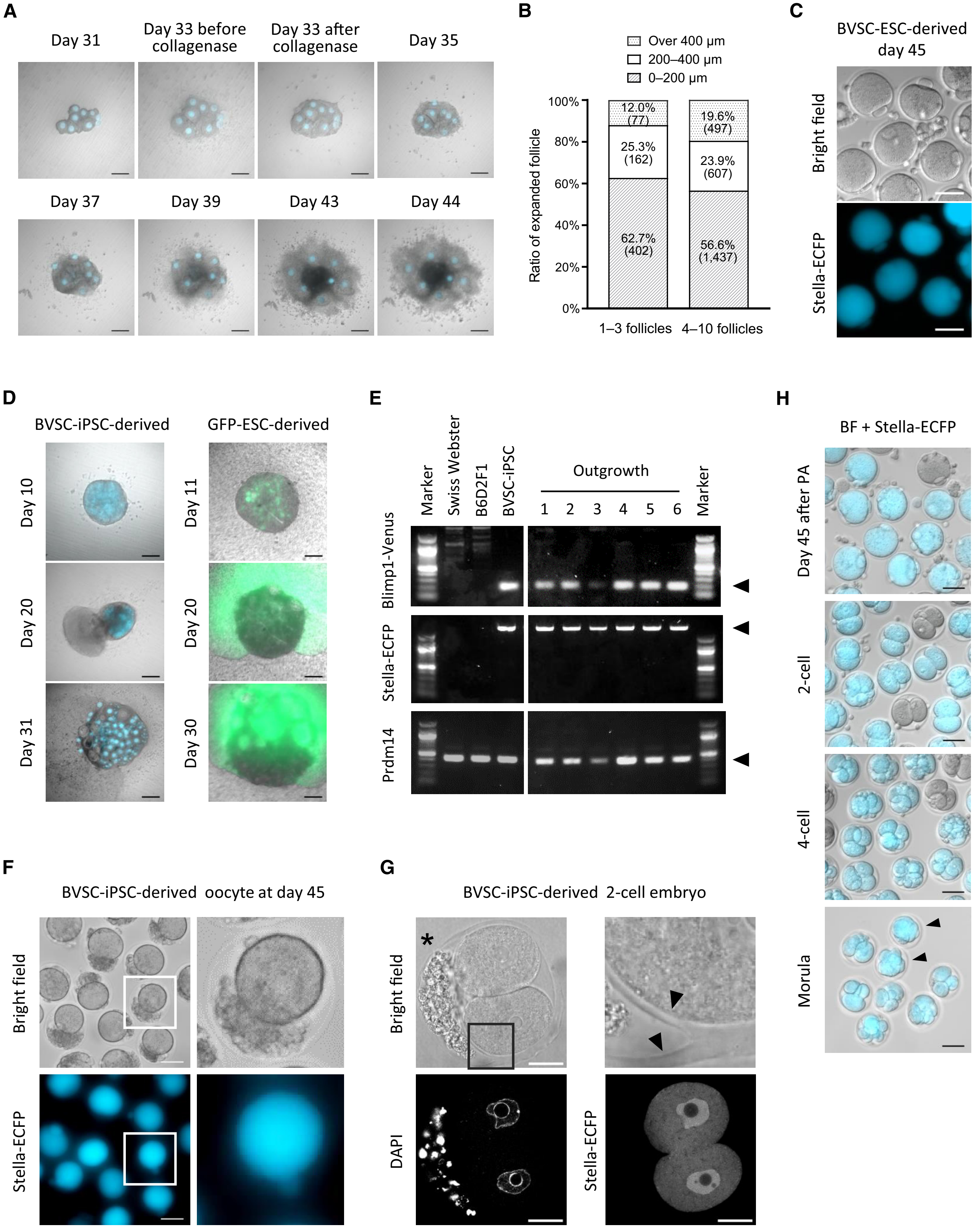

Figure EV2.

 © 2023 The Authors

Figure EV3. Immunostaining analysis of histone modification in 2-cell parthenotes and 5mC/5hmC in oocytes.

A Representative staining of H3K4me3 and H3K4ac in PA 2-cell embryos. Scale bar, 20 μm.
B Quantitative data of H3K4me3 and H3K4ac staining in PA 2-cell embryos. $N = 15$ (*in vivo*-derived) and 16 (iPSC-derived).
C Representative staining of H3K27me3 and H3K27ac in PA 2-cell embryos. Scale bar, 20 μm.
D Quantitative data of H3K27me3 and H3K27ac staining in PA 2-cell embryos. $N = 12$ (*in vivo*-derived) and 14 (iPSC-derived).
E Representative staining of 5mC and 5hmC in E12.5 gonad-derived oocytes after IVD (NSN oocytes) and IVG (SN oocytes). Scale bar, 10 μm.
F Quantitative data of 5mC and 5hmC in E12.5 gonad-derived oocytes after IVD (NSN oocytes) and IVG (SN oocytes). $N = 18$ (*in vivo*-derived, NSN), 18 (gonad-derived, NSN), 18 (*in vivo*-derived, SN) and 18 (gonad-derived, SN).

Data information: Bars represent mean ± SD. Statistical analysis was performed using an unpaired two-tailed *t*-test. *$P < 0.05$; **$P < 0.01$; ns, non-significant.
Source data are available online for this figure.

© 2023 The Authors

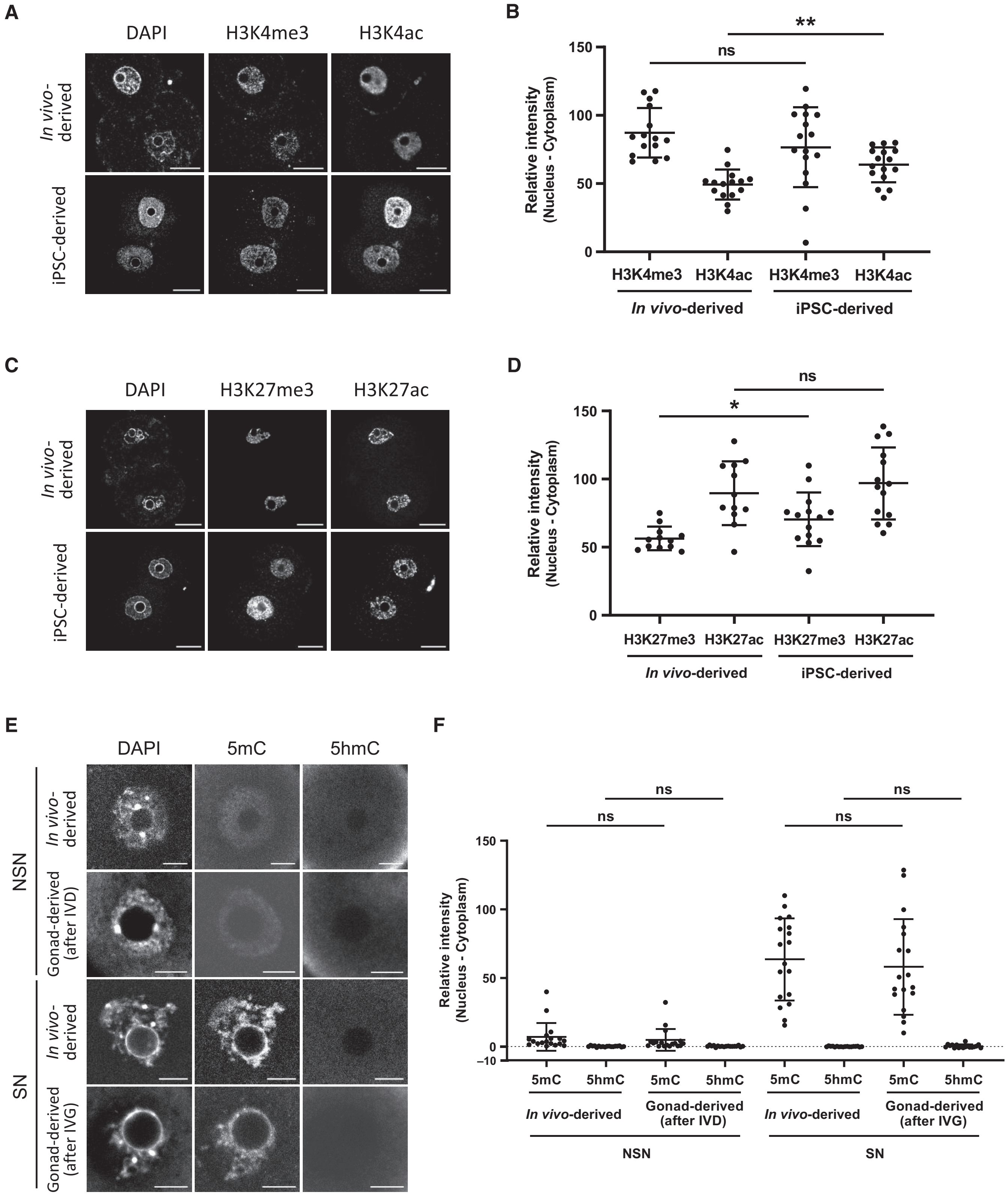

Figure EV3.

 © 2023 The Authors

Figure EV4. Promoter features of affected genes and comparison of genes differentially expressed in PGCLCs compared to PGCs.

A Principal Component Analysis (PCA) of bulk RNA-Seq samples from this study for d6 PGCLC (red), PGC in E12.5 female (blue) and published RNA-Seq datasets for d6 PGCLC (pink; Ohta *et al*, 2017) and PGC in E12.5 female (light blue; Sasaki *et al*, 2015). PCA was performed on relative expression calculated for the dataset from this study and datasets from Ohta *et al* (2017) and Sasaki *et al* (2015) separately.

B Scatter plot showing expression of genes in E12.5 PGCs versus d6 PGCLCs and numbers of differentially expressed genes (with FDR $\leq$ 5% and |log2(Fold-change)| $\geq$ 2).

C Scatter plots illustrating correlation between expression differences between d6 PGCLC and E12.5 female PGC profiled in this study and by authors (Sasaki *et al*, 2015) and (Ohta *et al*, 2017). Numbers of genes commonly up- and down-regulated genes in the two studies are marked in red and blue respectively (with FDR $\leq$ 5% and |log2(Fold-change)| $\geq$ 2). Numbers of genes with conflicting results between two studies are marked in black.

D Gene ontology enrichment (GO) analysis for down-regulated genes in PGCLC compared to PGC (related to Dataset EV2). Bubbles representing GO terms are scaled according to enrichments, colored according to statistical significance and positioned relative to each other to reflect similarities between significantly affected genes with corresponding GO terms (see Materials and Methods).

E GO enrichment analysis for genes with statistically significant expression response to *in vitro* development (related to Dataset EV5).

F Results of χ^2 tests and enrichments of genes controlled by CpG island (CGI) promoters and non-CpG island promoters (non-CGI). Enrichments with χ^2 test *P*-value bigger than 1% are considered statistically not significant and displayed as dots.

G Scatter plot illustrating enrichments (*X*-axis, log2 scale) and statistical significance (*Y*-axis, −log10 (adjusted *P*-value)) of transcription factor motifs in promoters of genes which are up- or down-regulated by each stage of *in vitro* development and display particular expression dynamics from GRO to FGO *in vivo* (Fig 5C and E).

H Scatter plots showing expression differences between E12.5 PGCs and d6 PGCLCs versus expression responses to each stage of *in vitro* development.

Source data are available online for this figure.

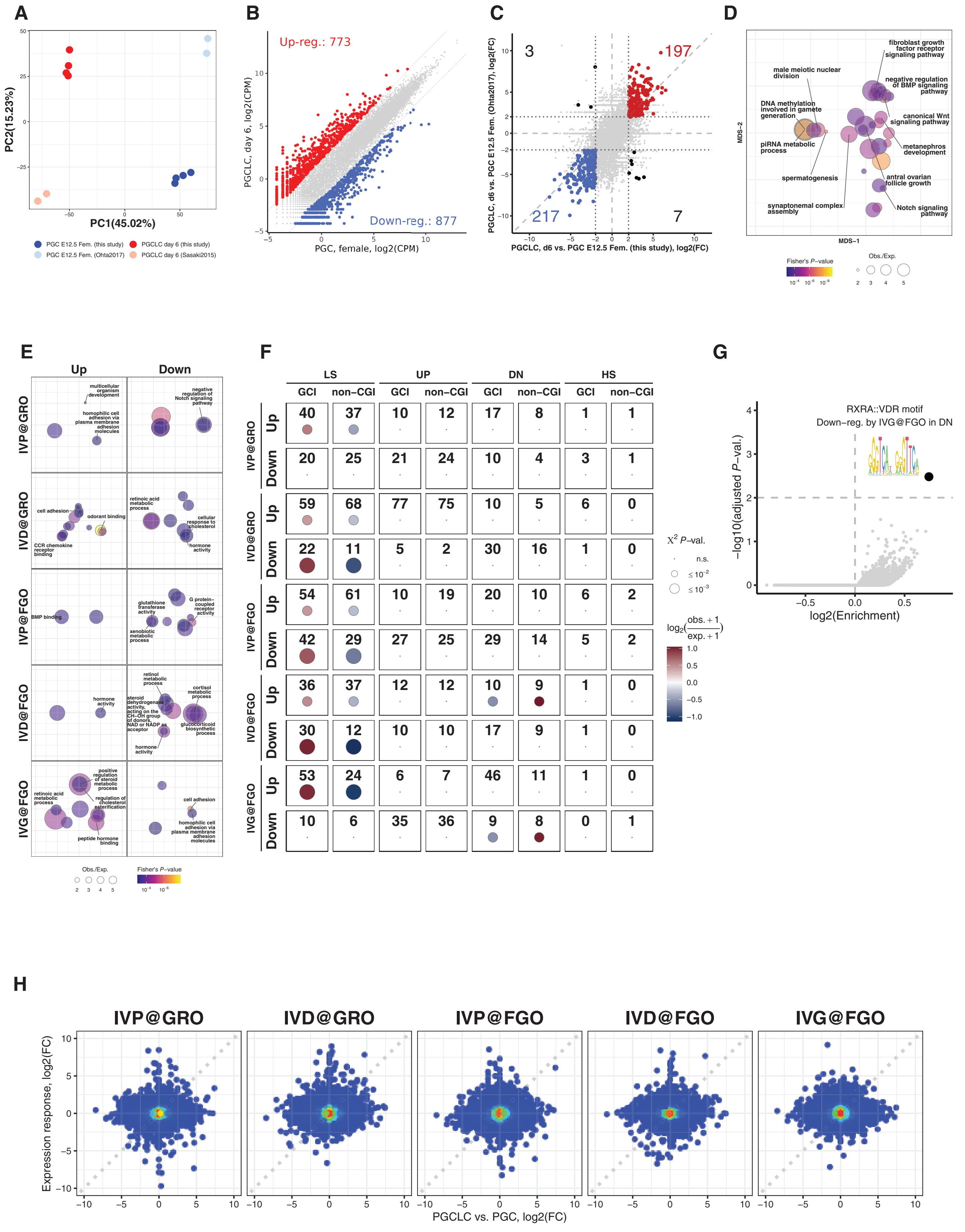

Figure EV4.

 © 2023 The Authors

Figure EV5. Investigation of chromatin features of promoters of genes affected by *in vitro* PGCLC and oogenesis procedure.

A Boxplots illustrating differences in sizes of oocytes belonging to different cohorts of GRO (GRO^{iPSC}, GRO^{PGC}, $GRO^{in\ vivo}$) and FGO (FGO^{iPSC}, FGO^{PGC}, $FGO^{6dpp\ Oo}$ and $FGO^{in\ vivo}$). Numbers of oocytes in each cohort are displayed below each boxplot.

B Investigation of possible confounding effect of oocyte size differences on results of differential expression analysis in GRO. Each point represents relative expression of a gene (*Y*-axis) in a particular GRO with corresponding size in *X*-axis. Left panel shows relative expression of all genes showing up-regulation dynamics (UP group) in GROs of different cohorts (GRO^{iPSC} in red, GRO^{PGC} in green, $GRO^{in\ vivo}$ in blue), middle and rights panels represent relative expression of genes belonging to UP2 and UP3 clusters shown in Fig 6B respectively.

C Enrichments (scaled and centered to Z-scores) of H3K27me3 in non-growing oocytes and FGO (data from Hanna *et al*, 2018b), H2AK119Ub1 in growing oocytes at day 7 and FGO (data from Mei *et al*, 2021) and H3K4me3 in non-growing oocytes and FGO (data from Hanna *et al*, 2018b) at CGI and non-CGI promoters (±1.5 kb) of genes with specific expression dynamics *in vivo* (LS, UP, DN, HS groups, see Fig 5C) and affected genes in UP cluster from Fig 6B. Upper/lower whiskers extend to the largest/smallest values no further than 1.5*IQR from the upper/lower hinge, where IQR is interquartile range or distance between 25th and 75th percentiles. Outliers are not displayed. The notches extend $1.5 * \text{IQR}/\sqrt{n}$ where *n* are numbers of CGI and non-CGI genes belonging to each group displayed below boxplots for H3K27me3 in NGO and the same for other stages and PTMs.

D Enrichments (scaled and centered to Z-scores) of H3K27me3 and H3K4me3 marks in PGC E13.5 (data from Kawabata *et al*, 2019) and d6 PGCLC (data from Kurimoto *et al*, 2015) at CGI and non-CGI promoters (±1.5 kb) of genes with specific expression dynamics *in vivo* (LS, UP, DN, HS groups, see Fig 5C) and affected genes in UP cluster from Fig 6B. Definitions of the central line, hinges, whiskers, notches as well numbers of genes in each group are the same as in Fig EV5C.

E Analogous to (C) but for affected genes in DN cluster from Fig 6B.

F Analogous to (D) but for affected genes in DN cluster from Fig 6B.

G Analogous to (C) but for affected genes in LS cluster from Fig 6B.

H Analogous to (D) but for affected genes in LS cluster from Fig 6B.

I Genomic snapshot of a representative gene *Psd2* belonging to the UP2 cluster (see Fig 6B) and distribution of several chromatin marks.

J Genomic snapshot of a representative gene *Lgi2* belonging to the UP3 cluster (see Fig 6B) and distribution of several chromatin marks.

Source data are available online for this figure.

© 2023 The Authors

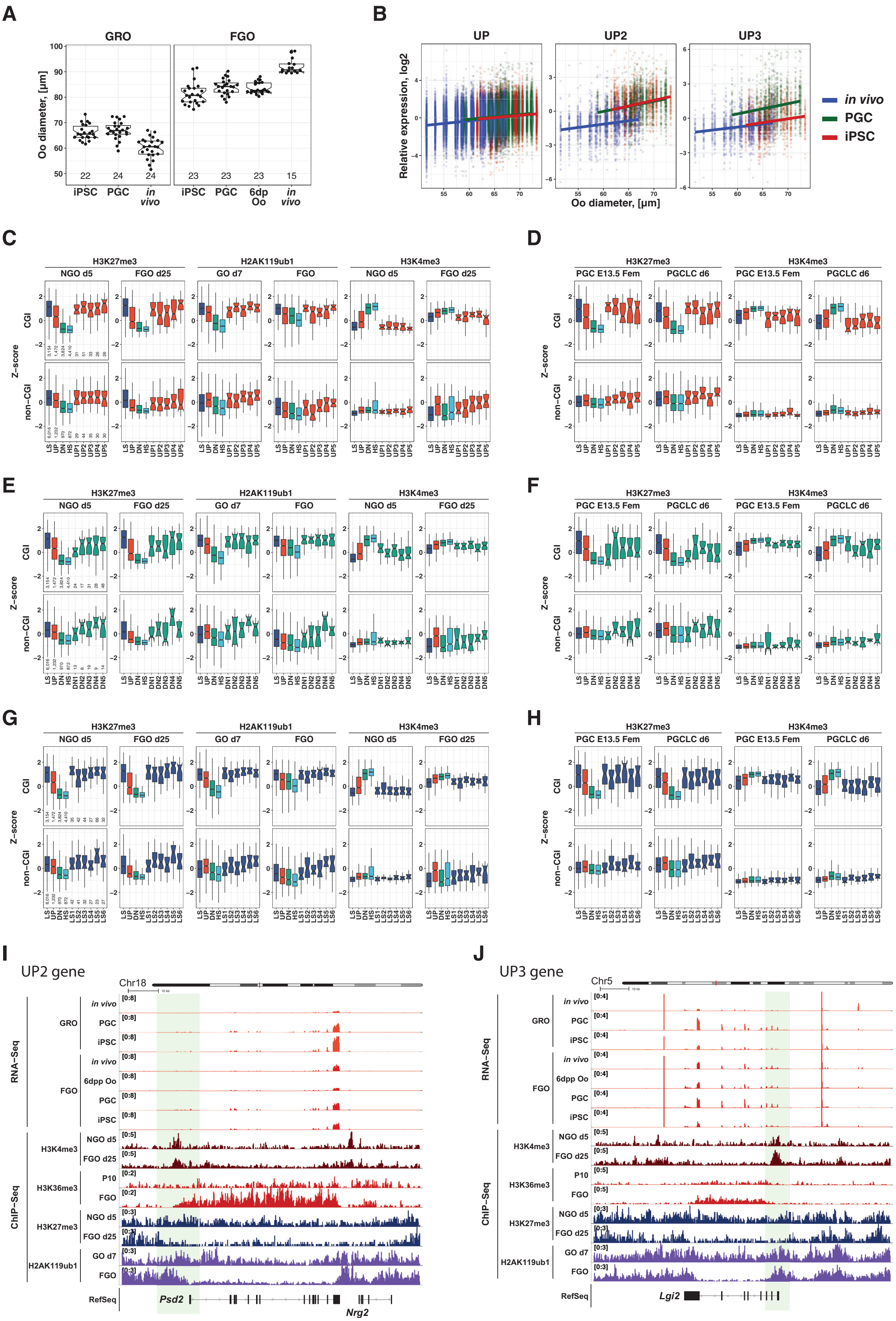

Figure EV5.

 © 2023 The Authors

Appendix

Epigenetic regulation limits competence of pluripotent stem cell-derived oocytes

Eishi Aizawa, Evgeniy A. Ozonov, Yumiko K. Kawamura, Charles-Etienne Dumeau, So Nagaoka, Tomoya S. Kitajima, Mitinori Saitou, Antoine H. F. M. Peters*, Anton Wutz*

*Correspondence: antoine.peters@fmi.ch (A.H.F.M.P.), awutz@ethz.ch (A.W.)

Table of Contents:

Appendix Figure S1 .... Page 2

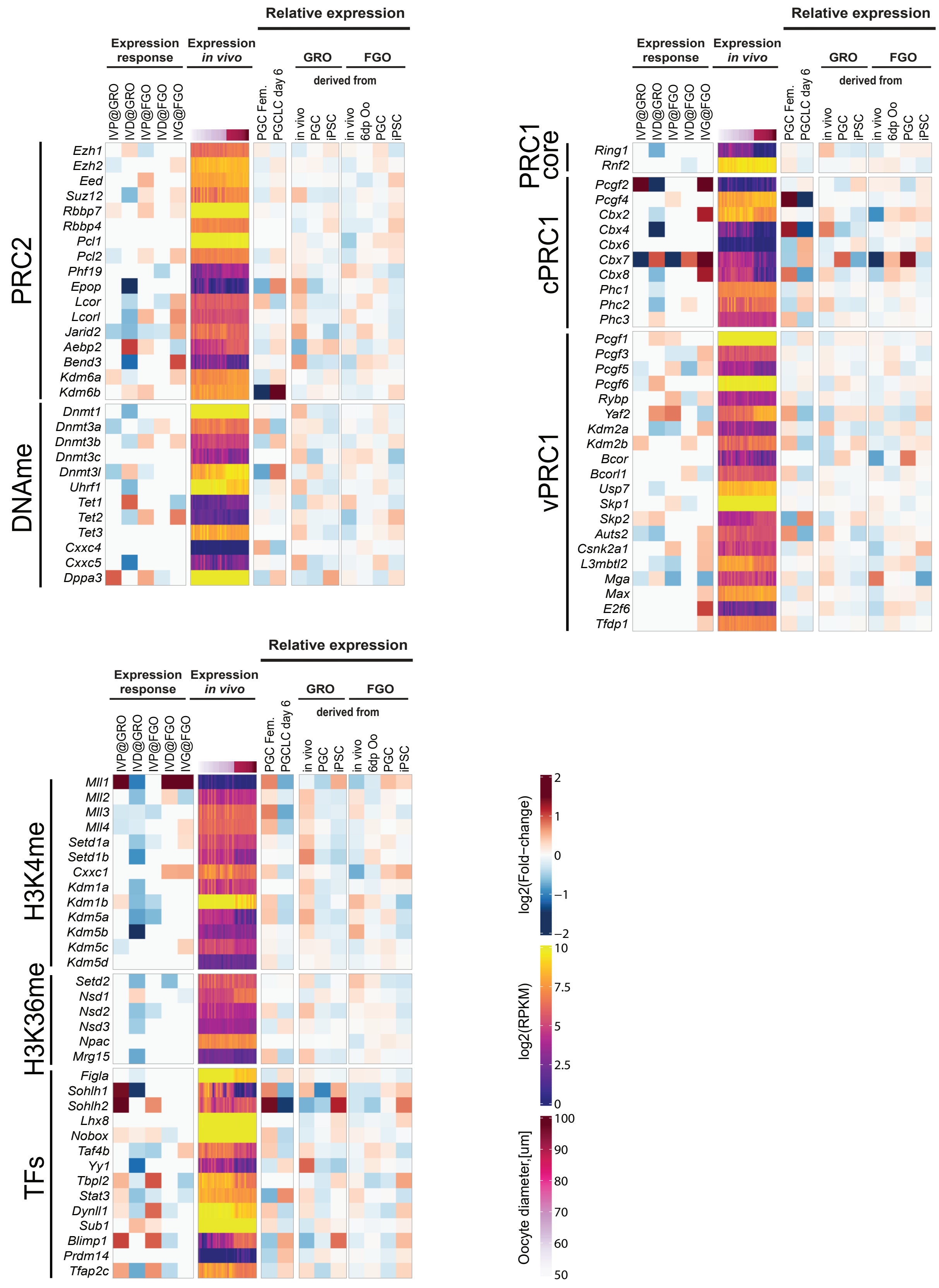

Appendix Figure S1. Expression of genes involved in epigenetic regulation and transcriptional reprogramming

Expression responses to each stage of *in vitro* development of genes involved in epigenetic regulation and transcriptional reprogramming (heatmap *Expression response*, all |*log*$_2$(*Fold-change*)| with *FDR* ≥ 5% are considered non-significant and set to 0). In addition, expression in *in vivo* oocytes (heatmap Expression *in vivo*), and relative expression in PGC and PGCLC, GRO and FGO are displayed (group of heatmaps Relative expression, data for biological replicates or single oocytes belonging to particular cohorts were merged). Relative expression for each gene was calculated as difference between expression level (*log*$_2$(RPKM)) and average expression calculated separately for PGC and PGCLC, GRO, and FGO.
