# Supplementary figures and images for: Epigenetic regulation limits competence of pluripotent stem cell‐derived oocytes
Source: EMBO J. 2023 Oct 18;42(23):e113955. doi: 10.15252/embj.2023113955 (PMC10690455; doi:10.15252/embj.2023113955)

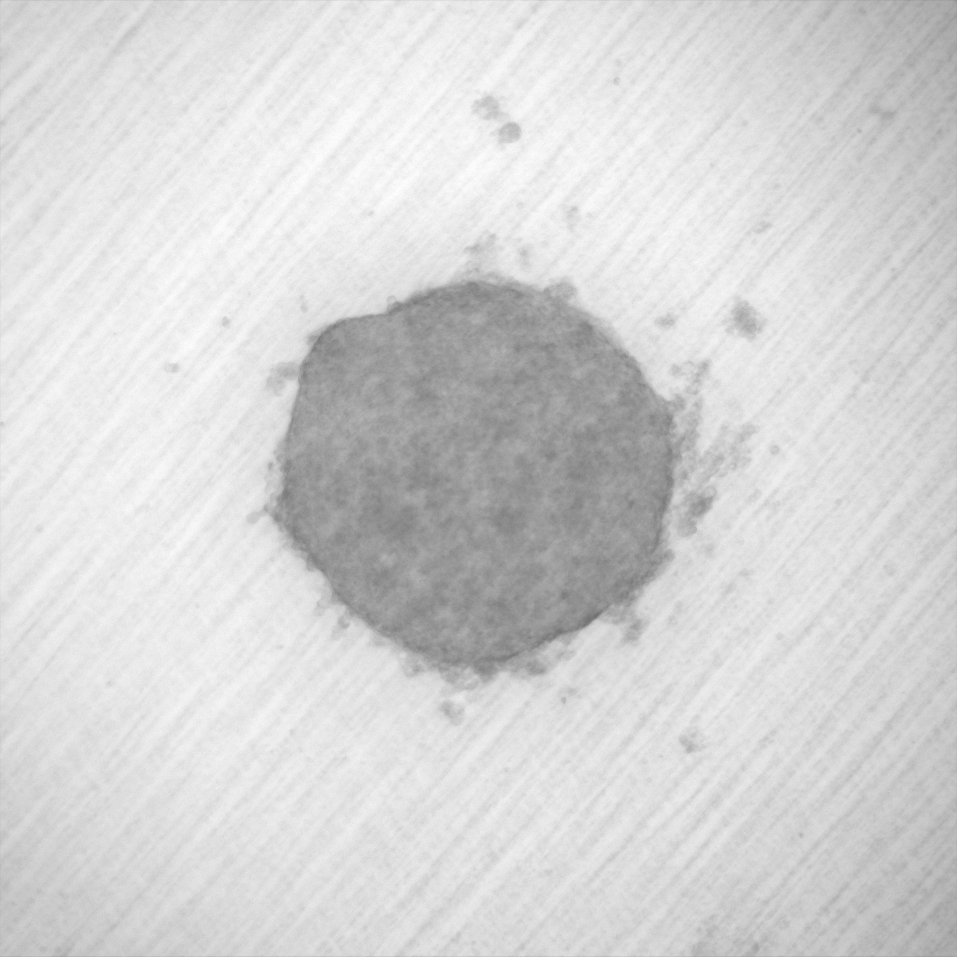

Supplement: Supplementary file 14 — Source Data for Figure 1 [file EMBJ-42-e113955-s019.zip › Figure_1/1C/Fig1C_d10_BF.tif]

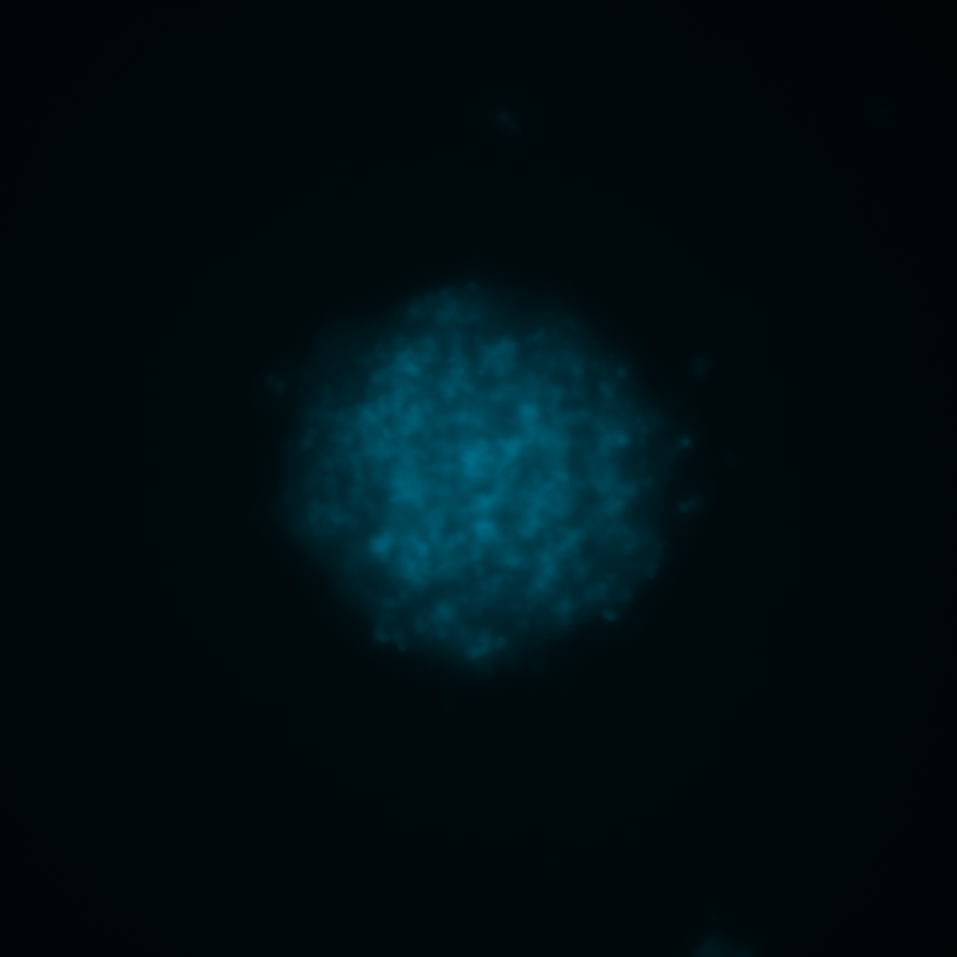

Supplement: Supplementary file 14 — Source Data for Figure 1 [file EMBJ-42-e113955-s019.zip › Figure_1/1C/Fig1C_d10_SC.tif]

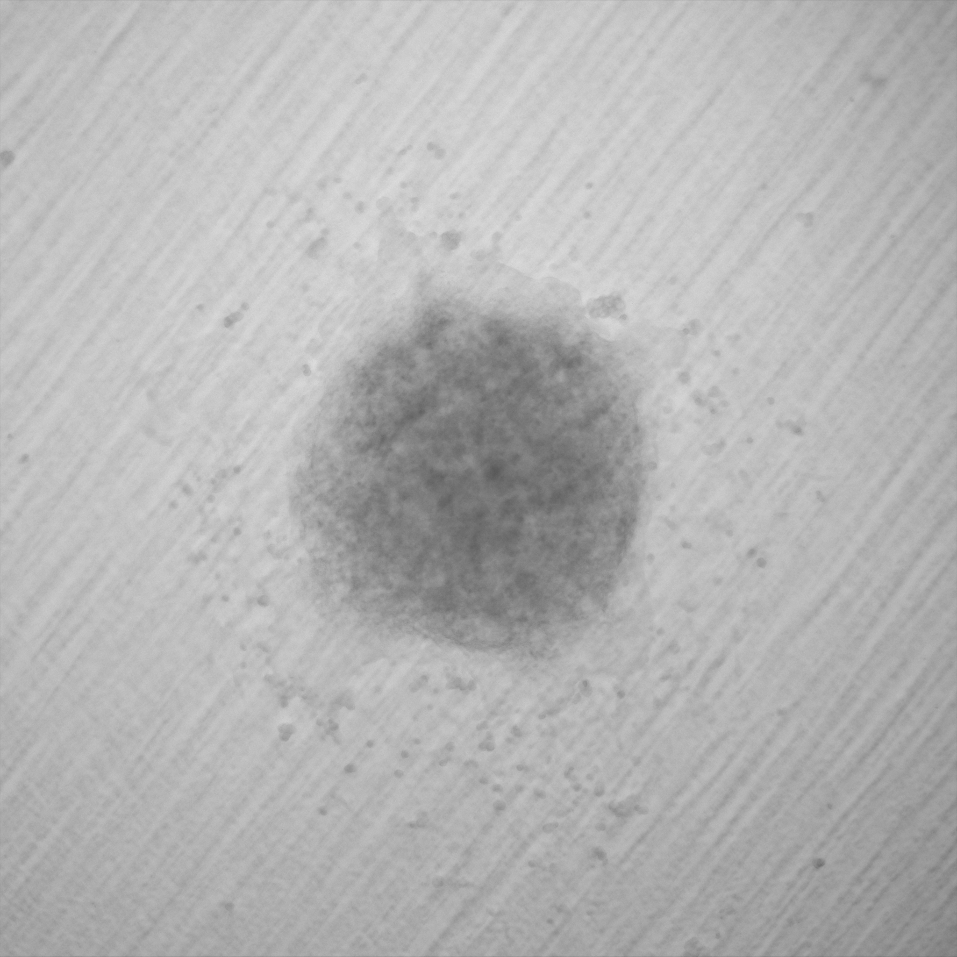

Supplement: Supplementary file 14 — Source Data for Figure 1 [file EMBJ-42-e113955-s019.zip › Figure_1/1C/Fig1C_d13_BF.tif]

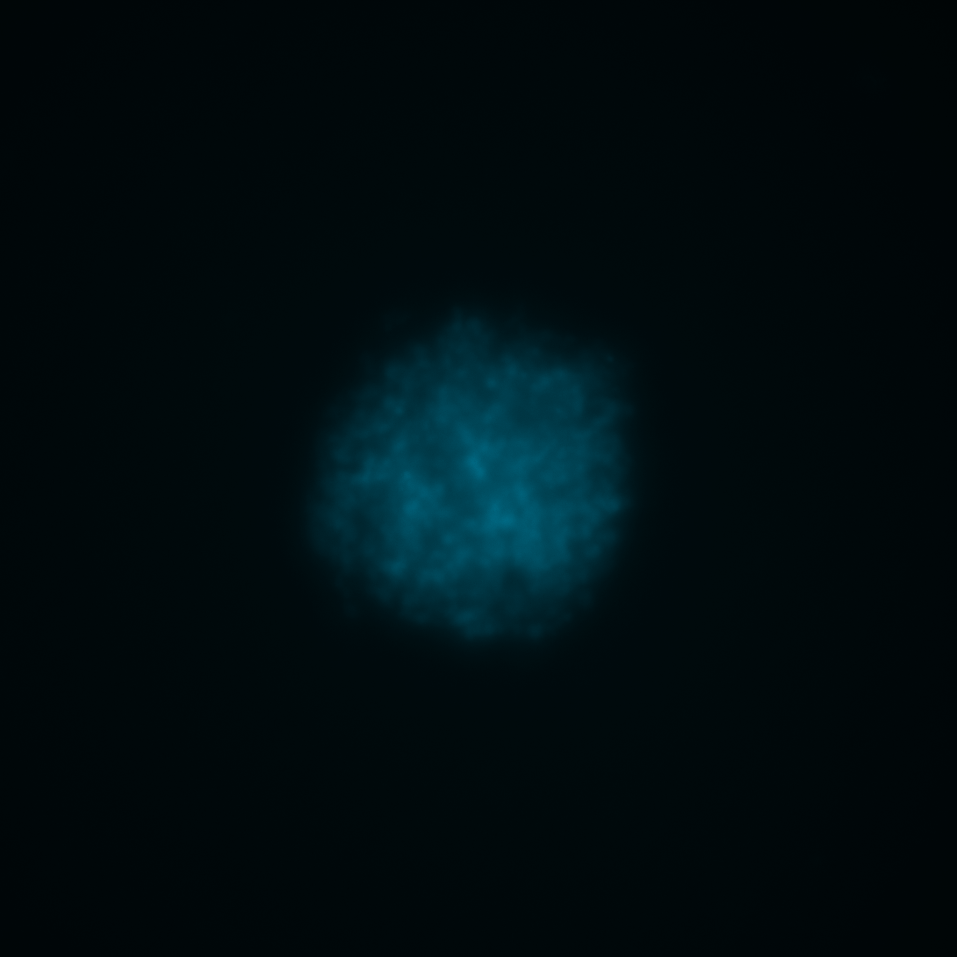

Supplement: Supplementary file 14 — Source Data for Figure 1 [file EMBJ-42-e113955-s019.zip › Figure_1/1C/Fig1C_d13_SC.tif]

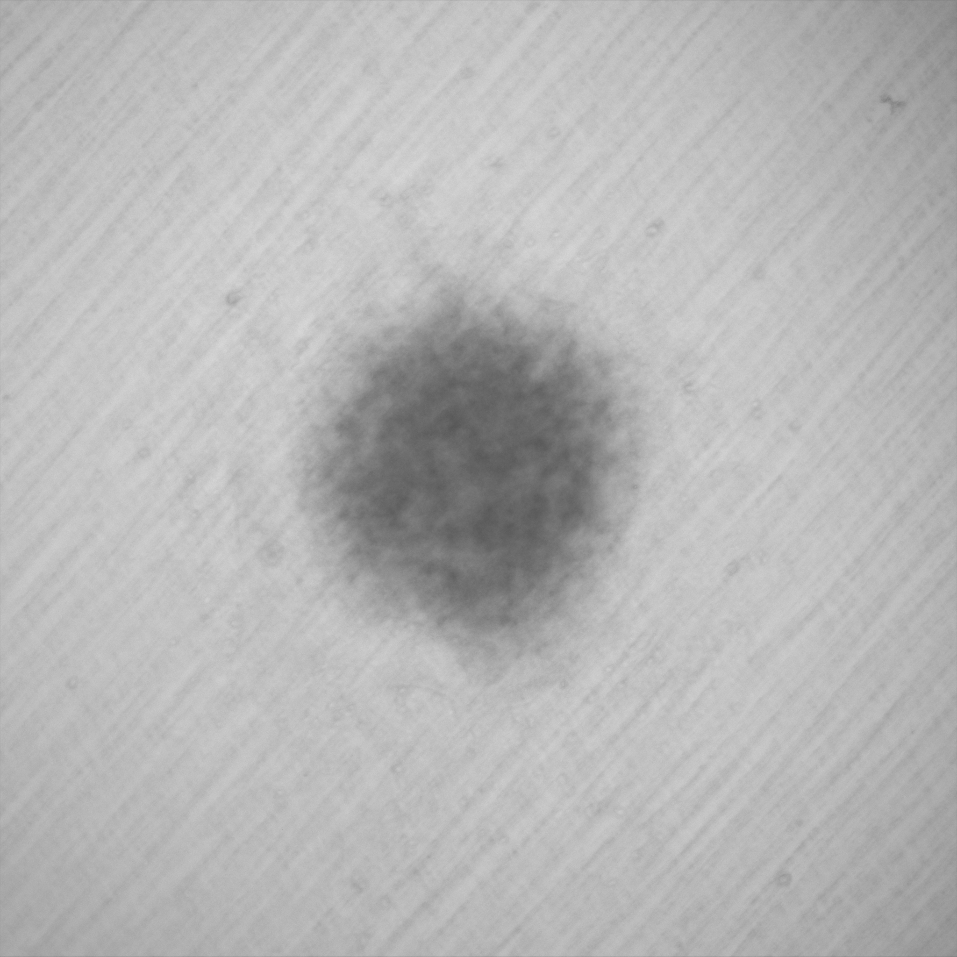

Supplement: Supplementary file 14 — Source Data for Figure 1 [file EMBJ-42-e113955-s019.zip › Figure_1/1C/Fig1C_d17_BF.tif]

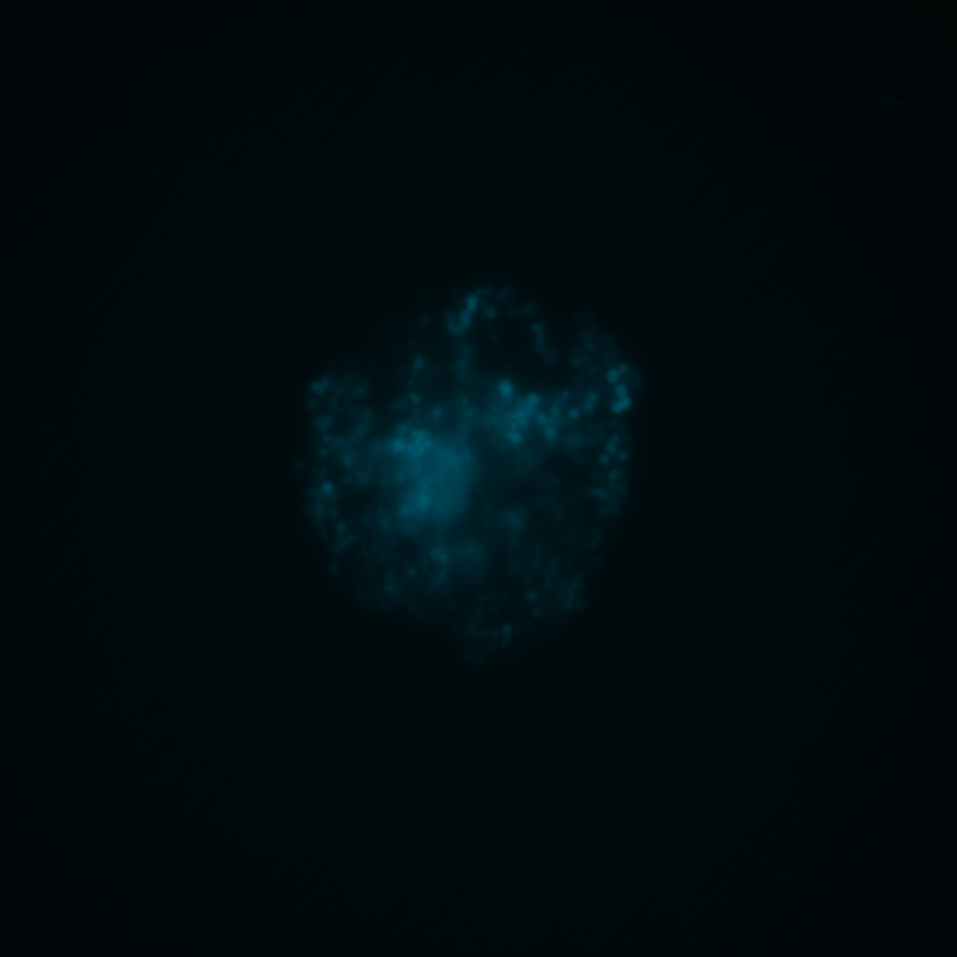

Supplement: Supplementary file 14 — Source Data for Figure 1 [file EMBJ-42-e113955-s019.zip › Figure_1/1C/Fig1C_d17_SC.tif]

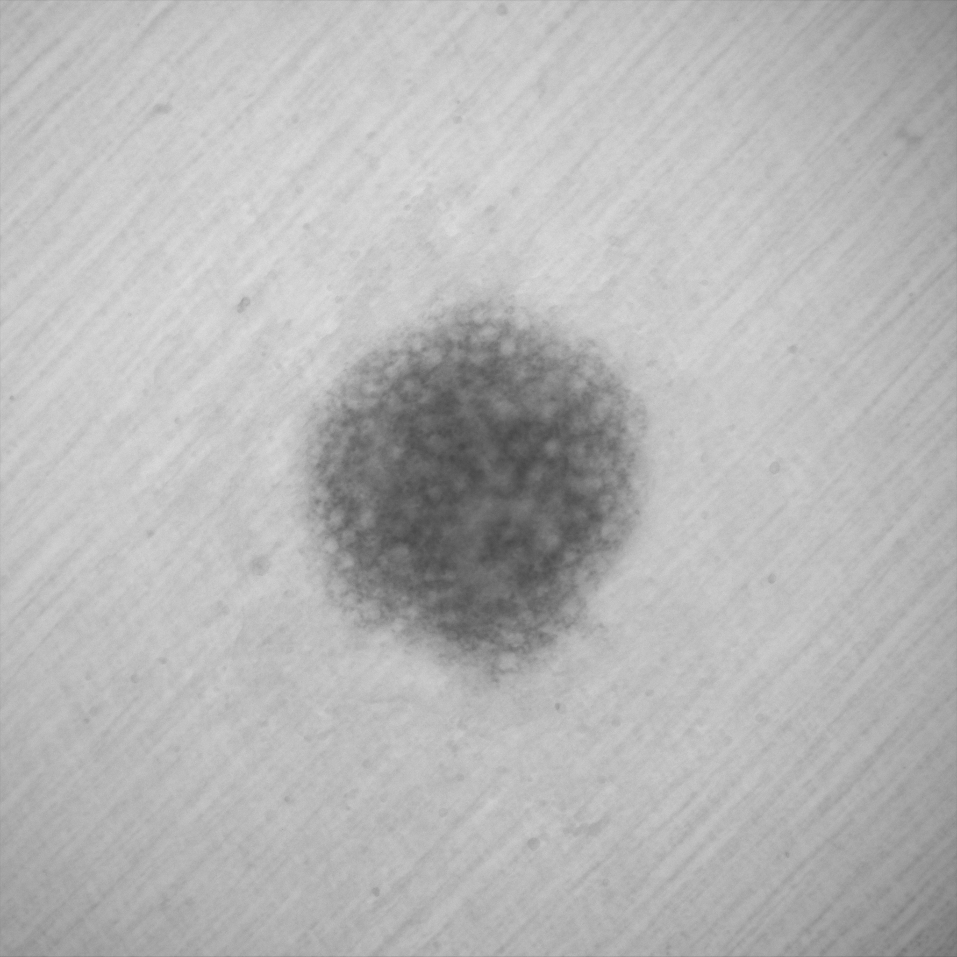

Supplement: Supplementary file 14 — Source Data for Figure 1 [file EMBJ-42-e113955-s019.zip › Figure_1/1C/Fig1C_d20_BF.tif]

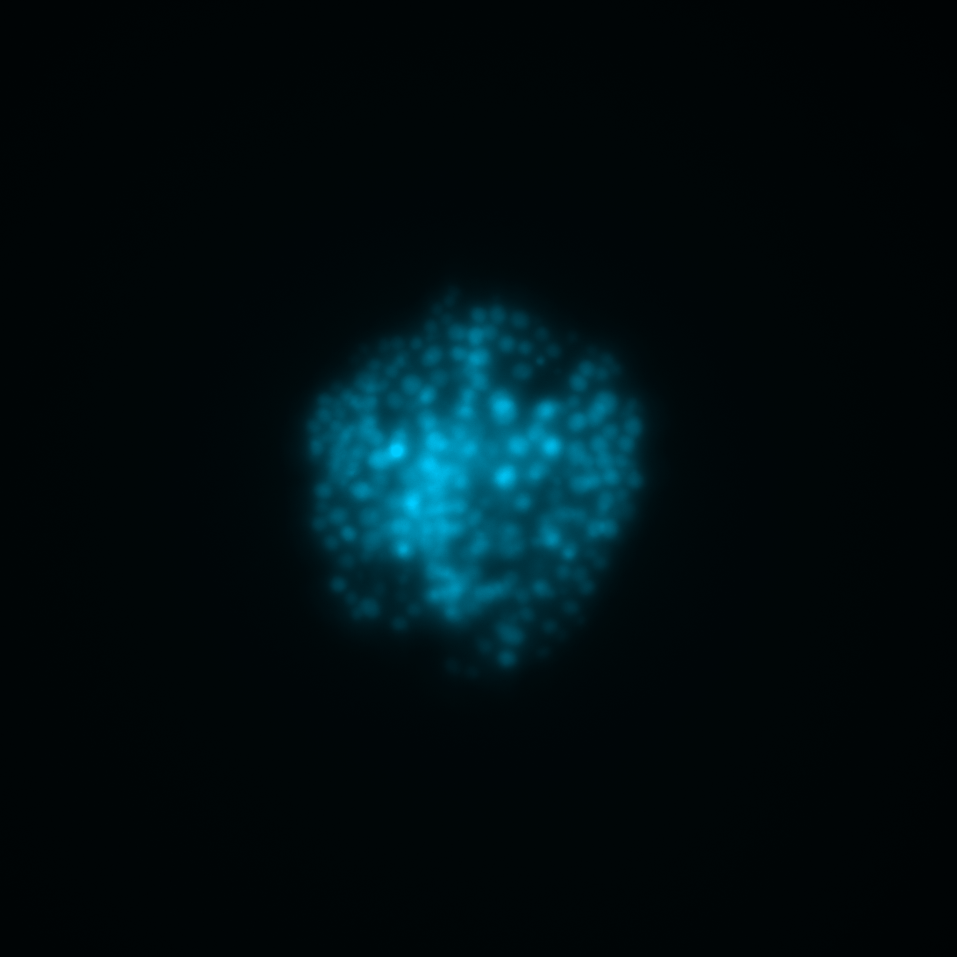

Supplement: Supplementary file 14 — Source Data for Figure 1 [file EMBJ-42-e113955-s019.zip › Figure_1/1C/Fig1C_d20_SC.tif]

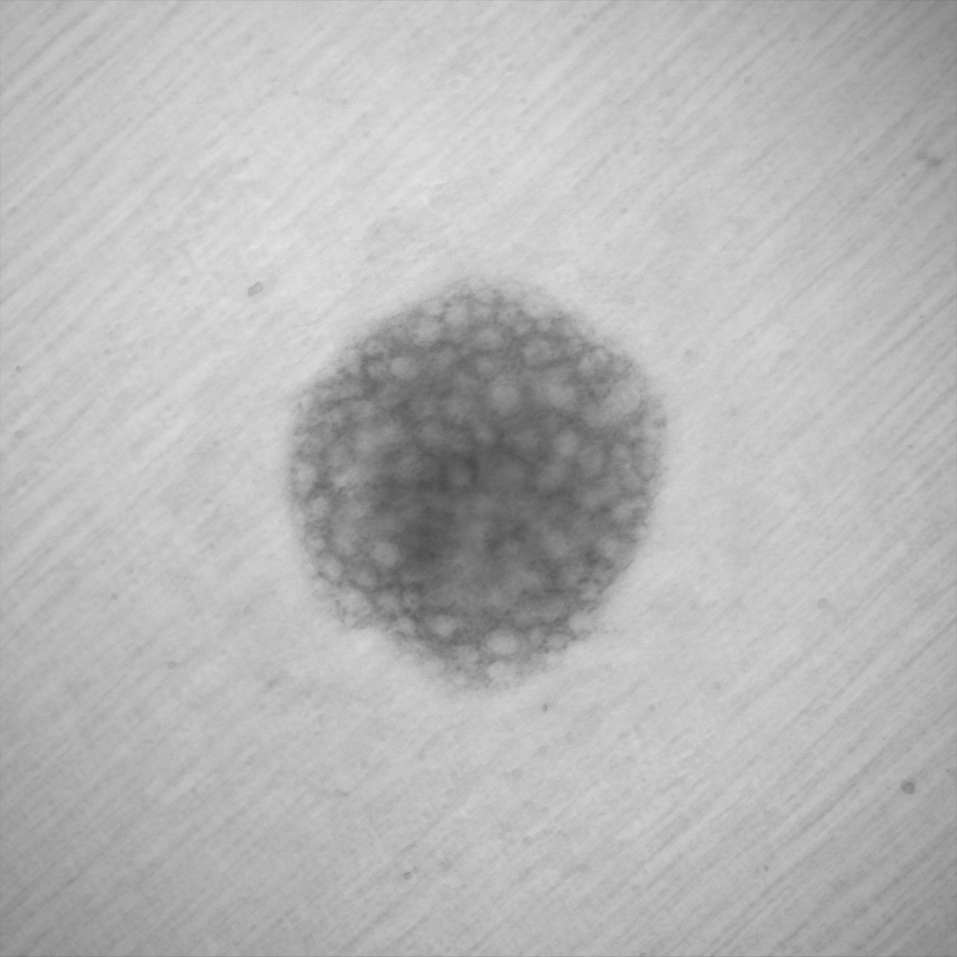

Supplement: Supplementary file 14 — Source Data for Figure 1 [file EMBJ-42-e113955-s019.zip › Figure_1/1C/Fig1C_d23_BF.tif]

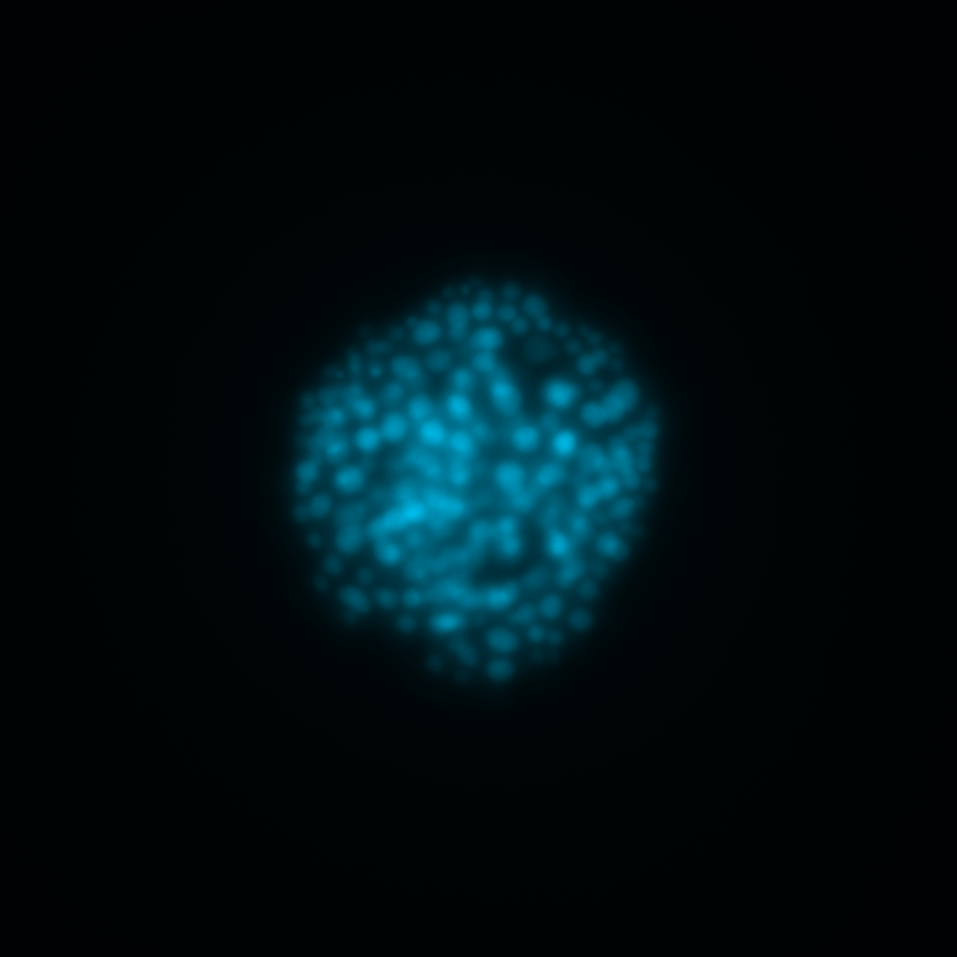

Supplement: Supplementary file 14 — Source Data for Figure 1 [file EMBJ-42-e113955-s019.zip › Figure_1/1C/Fig1C_d23_SC.tif]

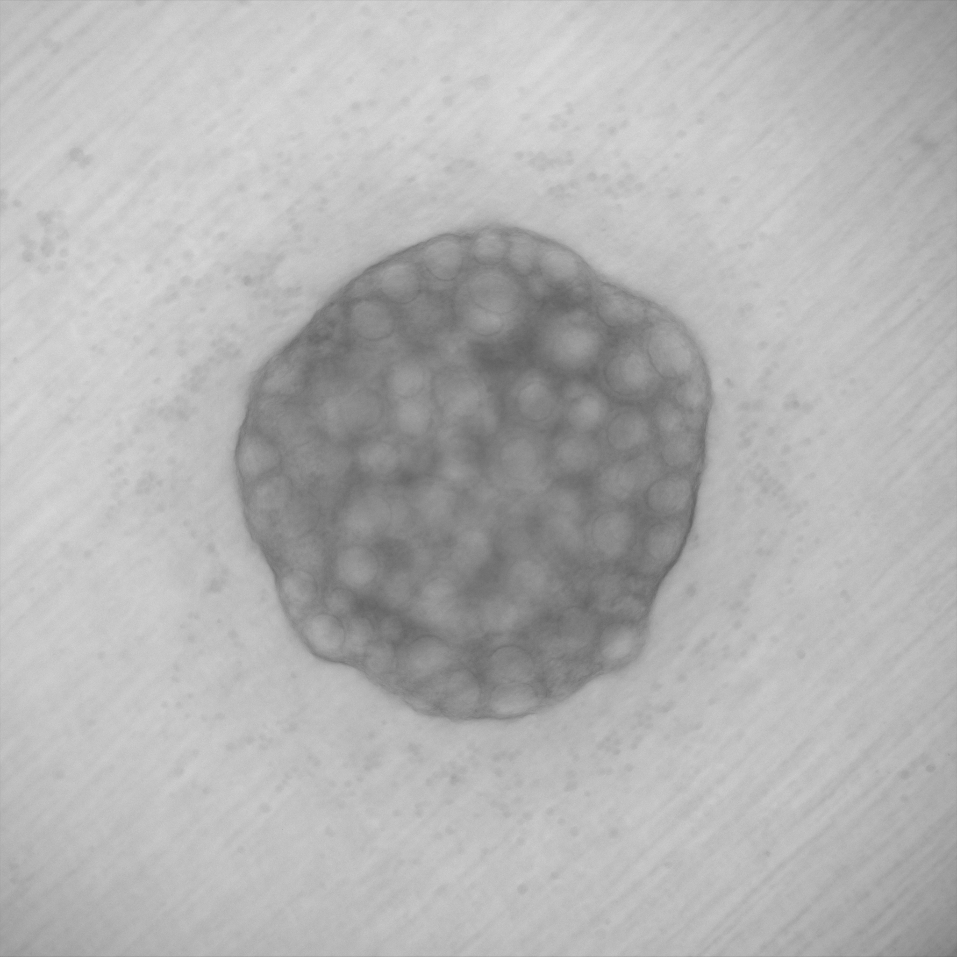

Supplement: Supplementary file 14 — Source Data for Figure 1 [file EMBJ-42-e113955-s019.zip › Figure_1/1C/Fig1C_d27_BF.tif]

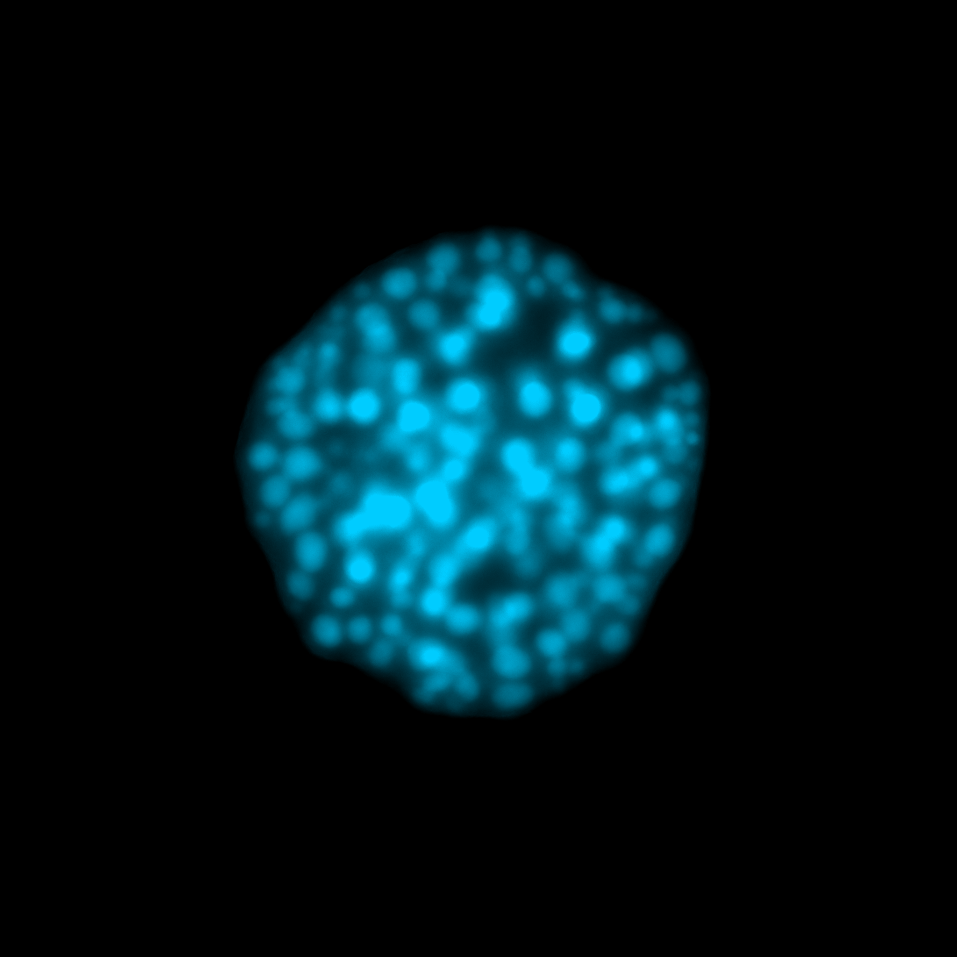

Supplement: Supplementary file 14 — Source Data for Figure 1 [file EMBJ-42-e113955-s019.zip › Figure_1/1C/Fig1C_d27_SC.tif]

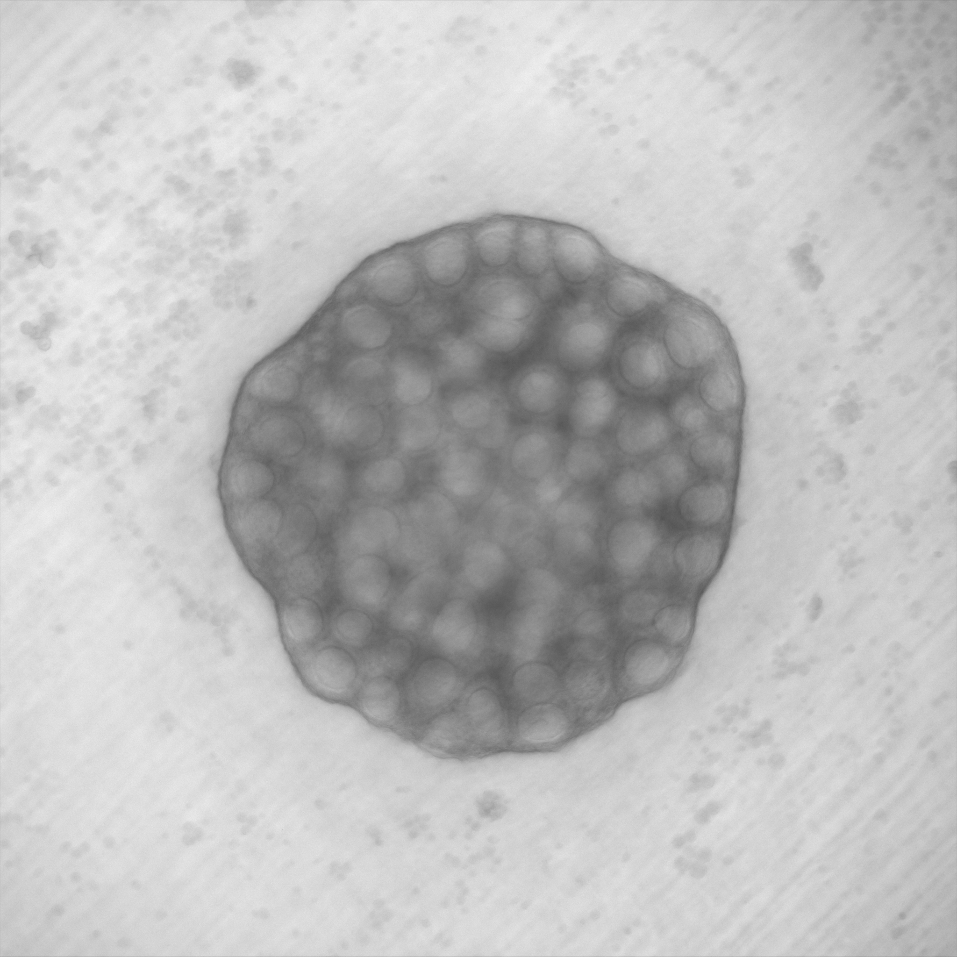

Supplement: Supplementary file 14 — Source Data for Figure 1 [file EMBJ-42-e113955-s019.zip › Figure_1/1C/Fig1C_d31_BF.tif]

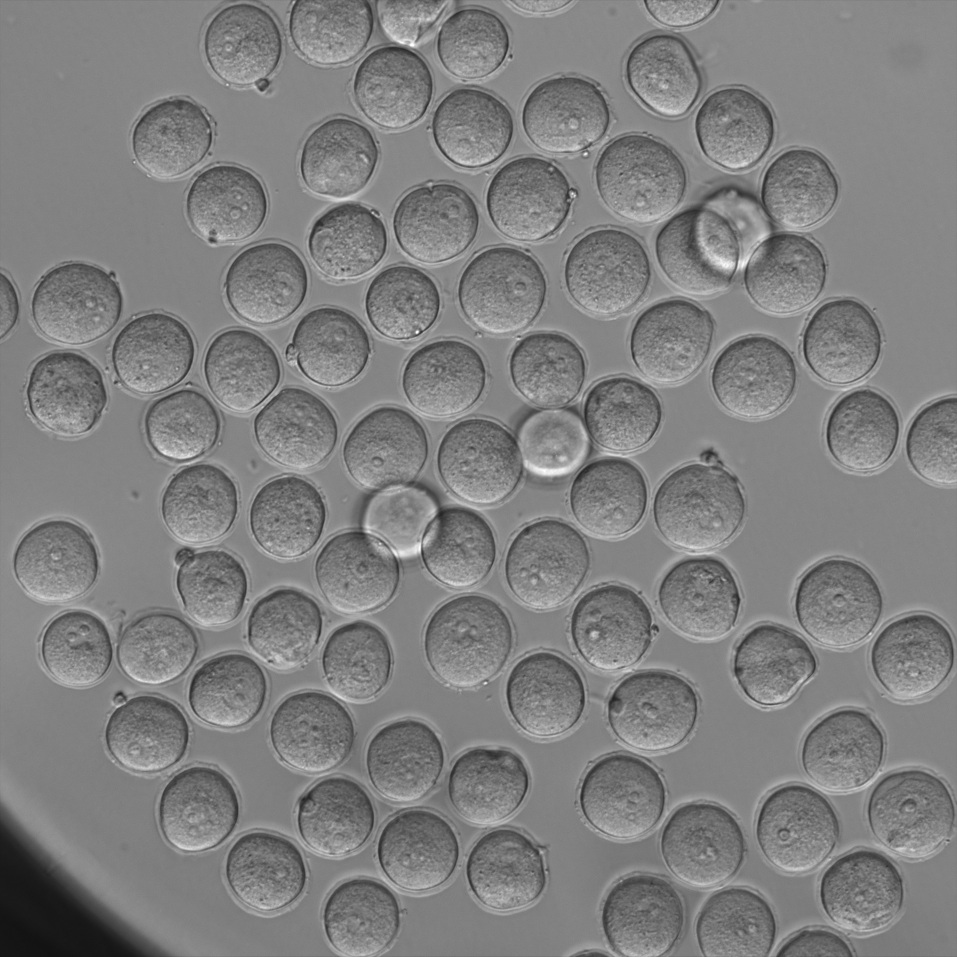

Supplement: Supplementary file 14 — Source Data for Figure 1 [file EMBJ-42-e113955-s019.zip › Figure_1/1C/Fig1C_d31_BF_oocytes.tif]

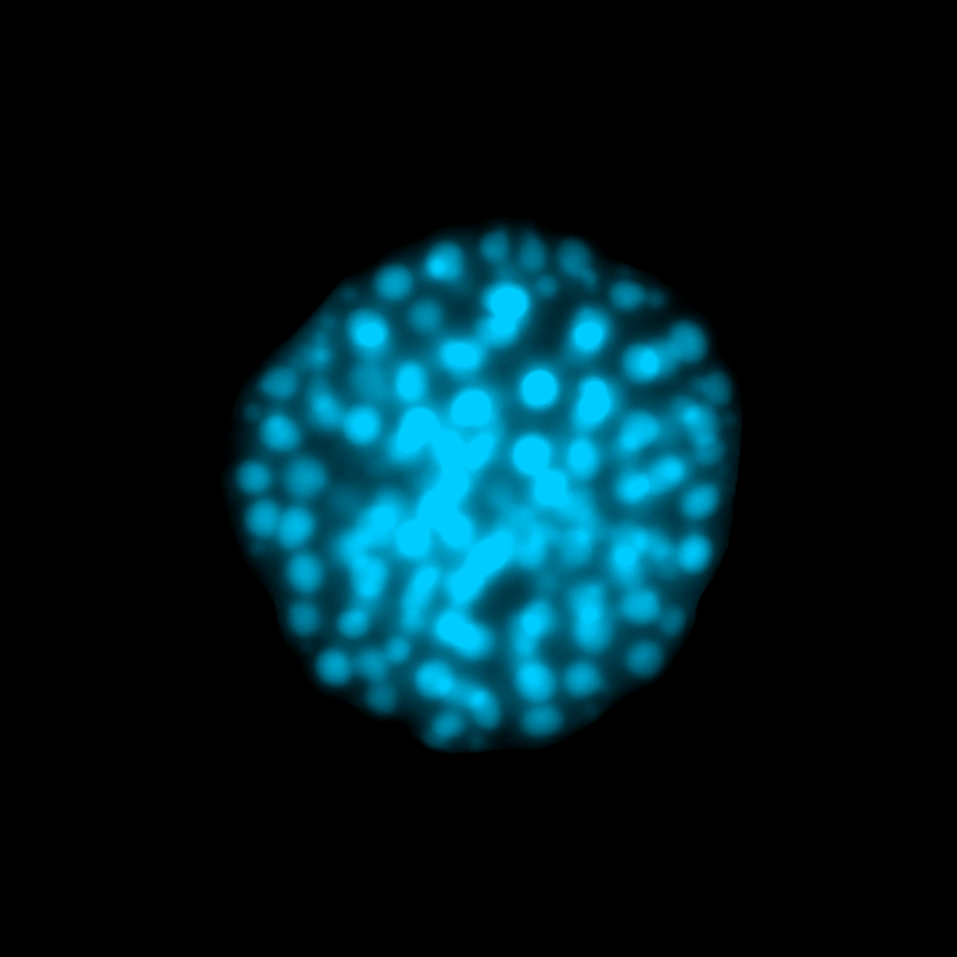

Supplement: Supplementary file 14 — Source Data for Figure 1 [file EMBJ-42-e113955-s019.zip › Figure_1/1C/Fig1C_d31_SC.tif]

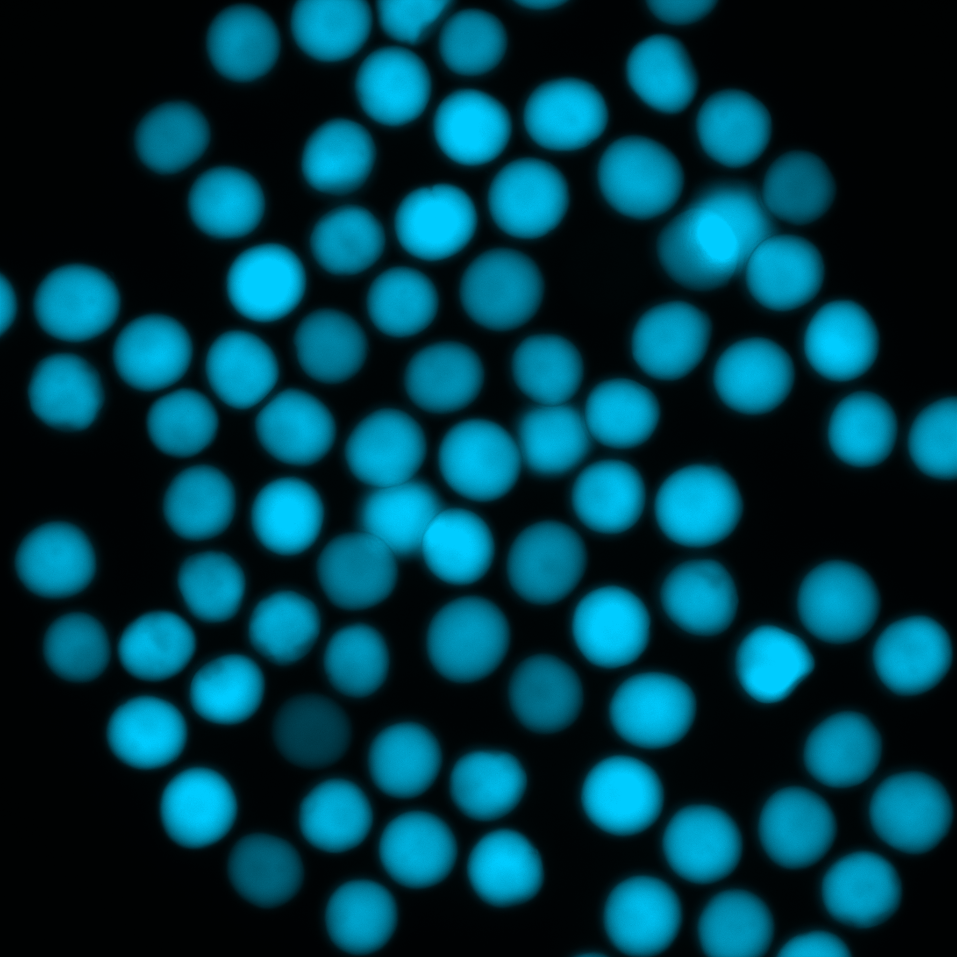

Supplement: Supplementary file 14 — Source Data for Figure 1 [file EMBJ-42-e113955-s019.zip › Figure_1/1C/Fig1C_d31_SC_oocytes.tif]

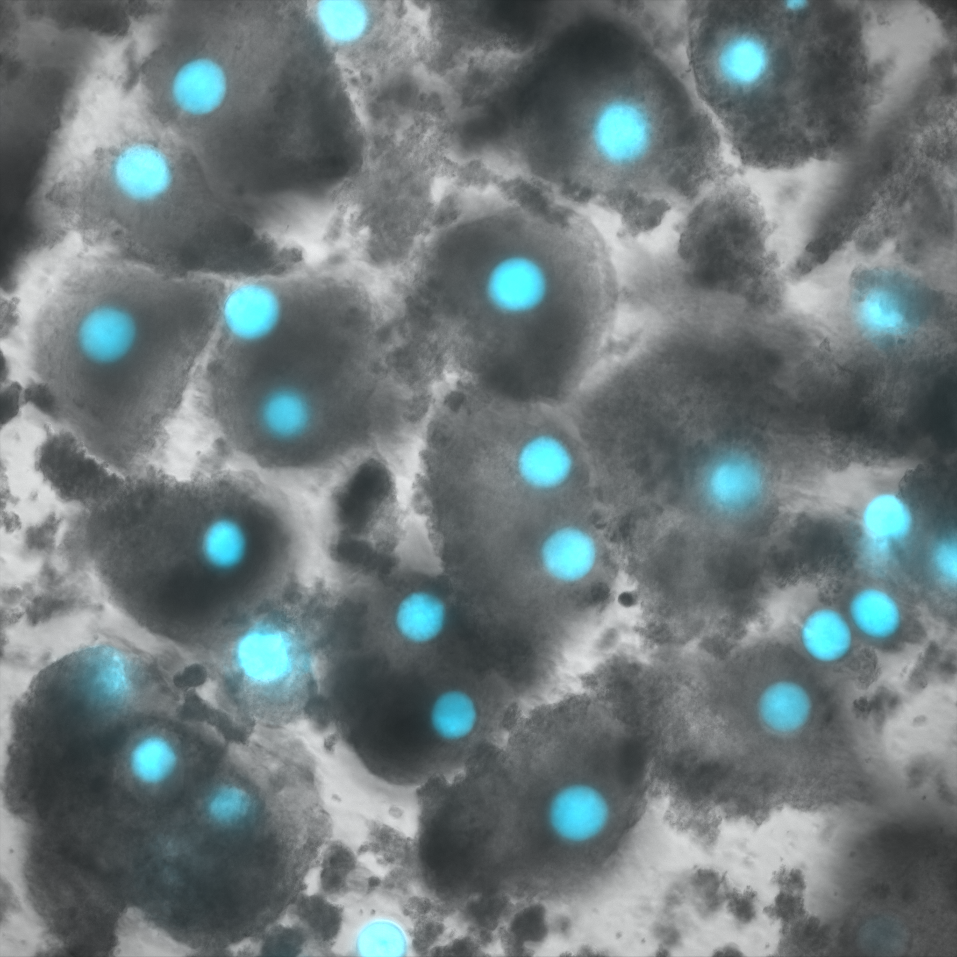

Supplement: Supplementary file 14 — Source Data for Figure 1 [file EMBJ-42-e113955-s019.zip › Figure_1/1E/Fig1E_d44.tif]

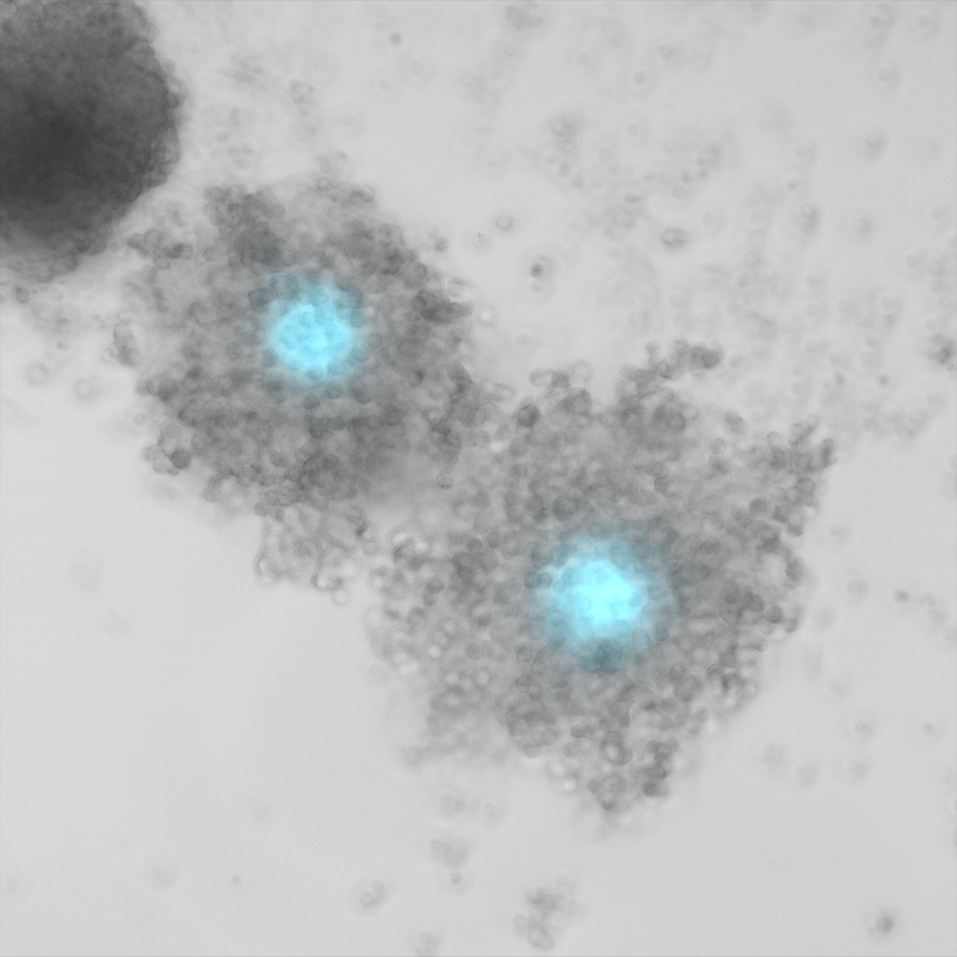

Supplement: Supplementary file 14 — Source Data for Figure 1 [file EMBJ-42-e113955-s019.zip › Figure_1/1E/Fig1E_d45_COC.tif]

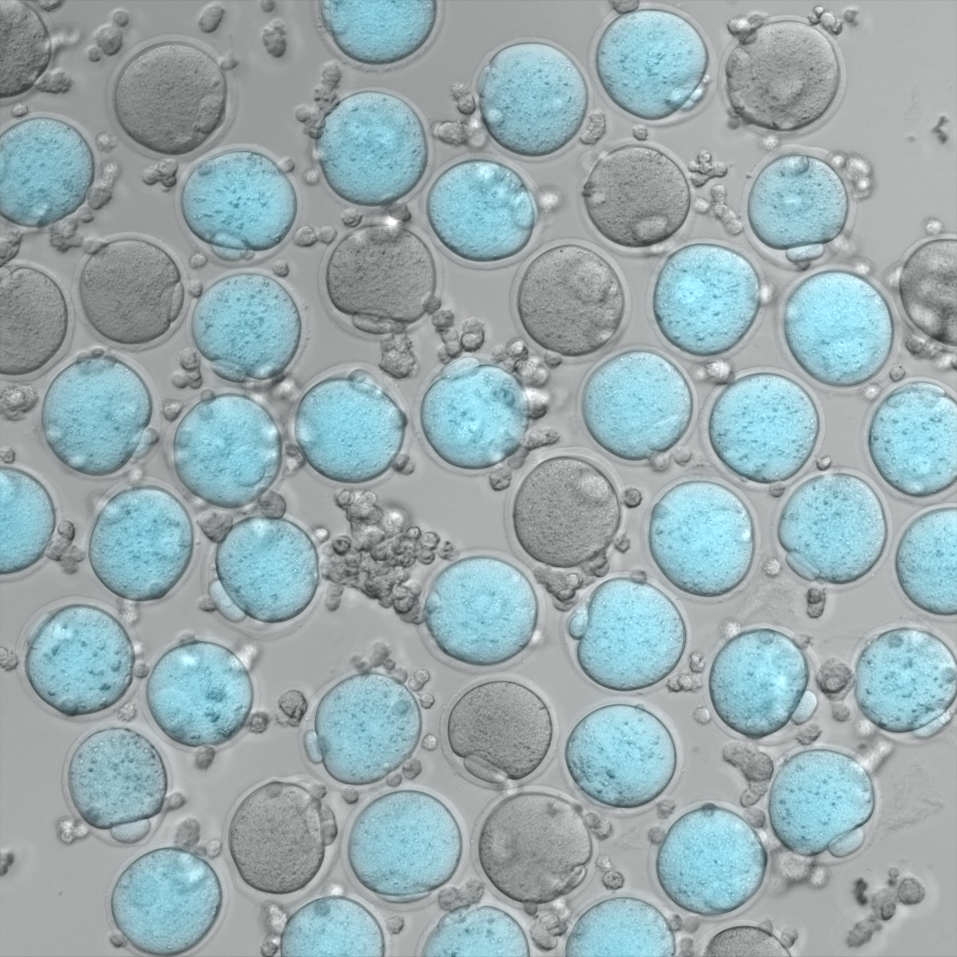

Supplement: Supplementary file 14 — Source Data for Figure 1 [file EMBJ-42-e113955-s019.zip › Figure_1/1E/Fig1E_d45_Oocytes.tif]

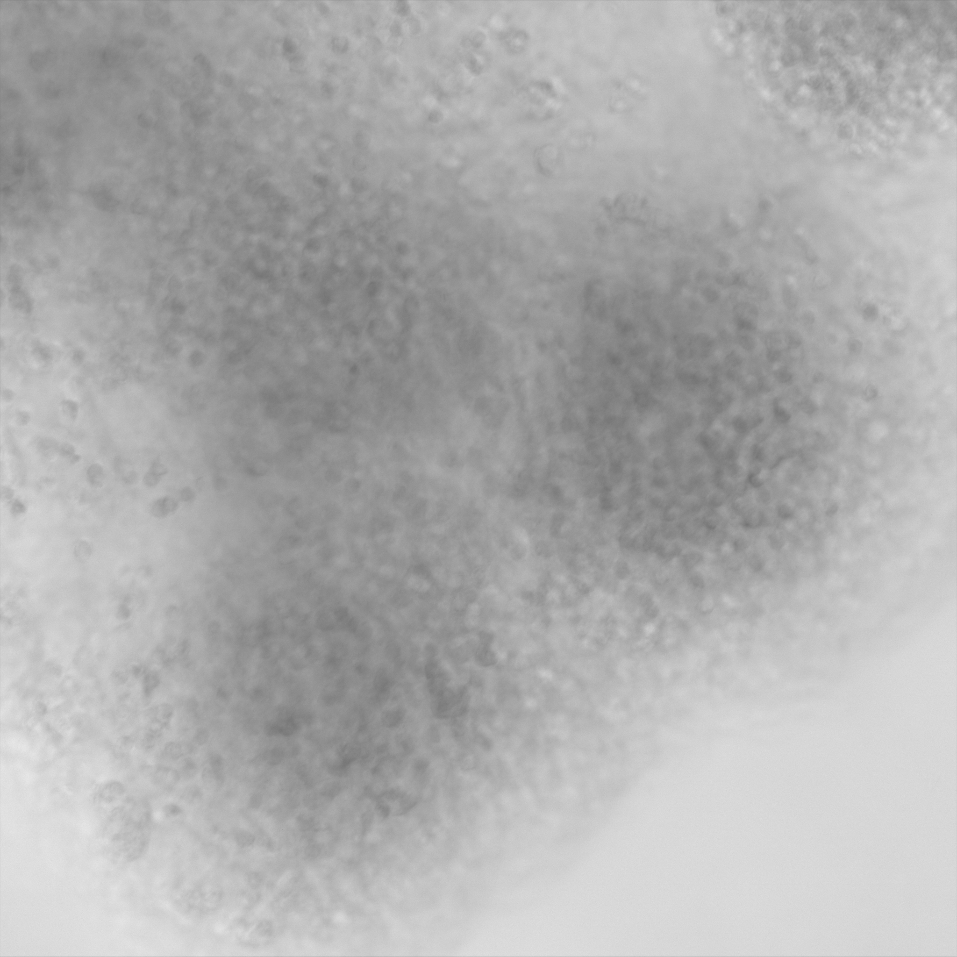

Supplement: Supplementary file 14 — Source Data for Figure 1 [file EMBJ-42-e113955-s019.zip › Figure_1/1E/Fig1E_vivo.tif]

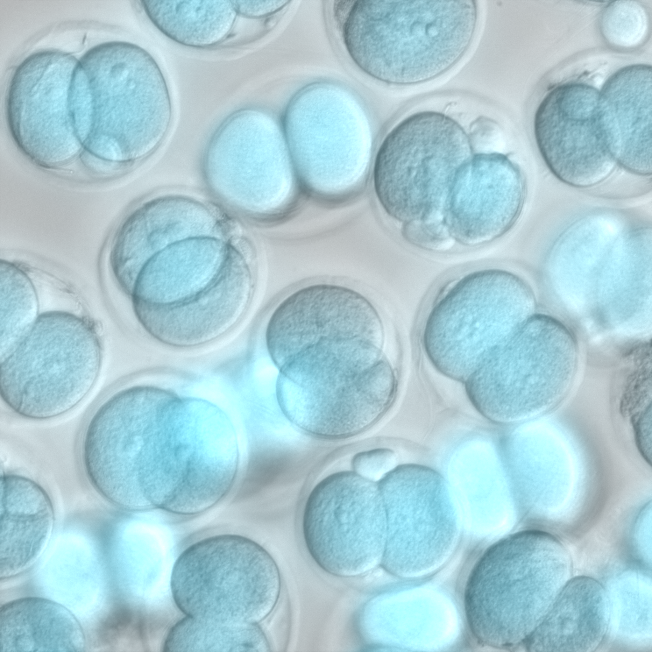

Supplement: Supplementary file 15 — Source Data for Figure 2 [file EMBJ-42-e113955-s011.zip › Figure_2/2B/Fig2B_2cell.tif]

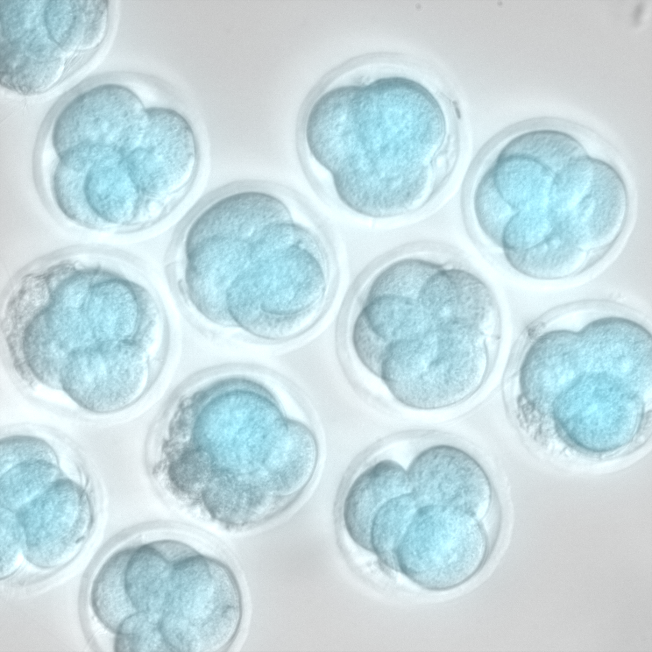

Supplement: Supplementary file 15 — Source Data for Figure 2 [file EMBJ-42-e113955-s011.zip › Figure_2/2B/Fig2B_4cell.tif]

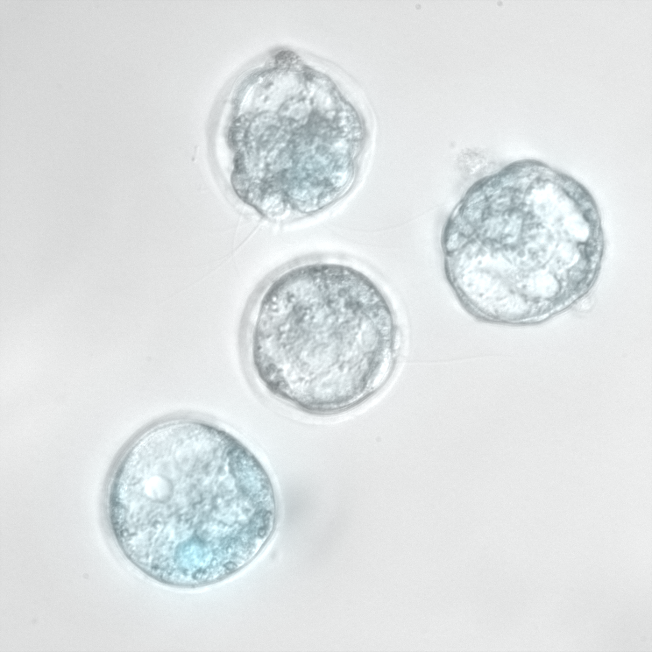

Supplement: Supplementary file 15 — Source Data for Figure 2 [file EMBJ-42-e113955-s011.zip › Figure_2/2B/Fig2B_Blast.tif]

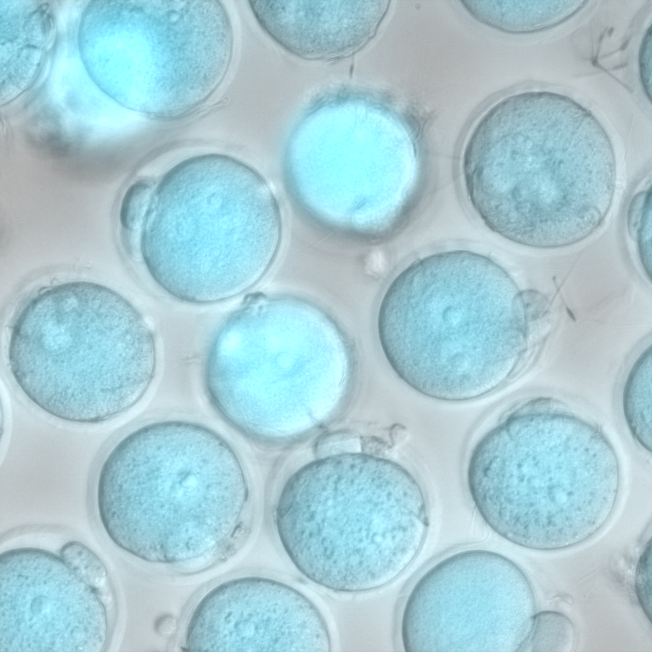

Supplement: Supplementary file 15 — Source Data for Figure 2 [file EMBJ-42-e113955-s011.zip › Figure_2/2B/Fig2B_IVF.tif]

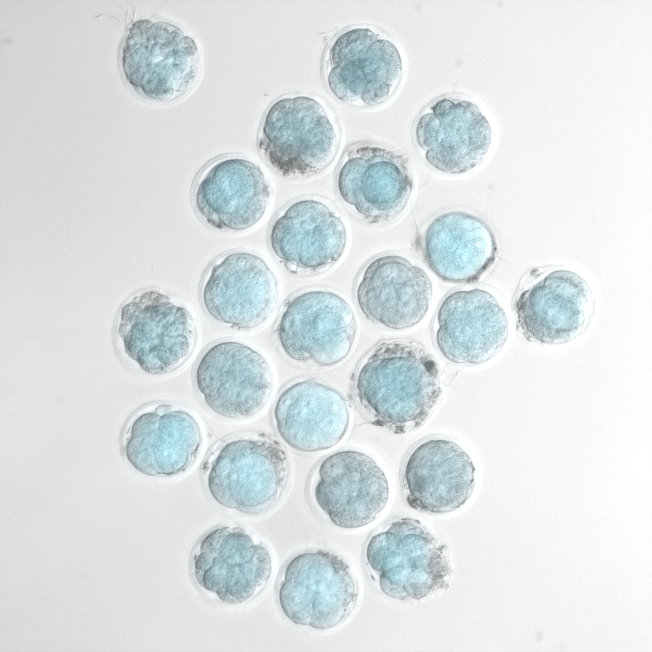

Supplement: Supplementary file 15 — Source Data for Figure 2 [file EMBJ-42-e113955-s011.zip › Figure_2/2B/Fig2B_Morula.tif]

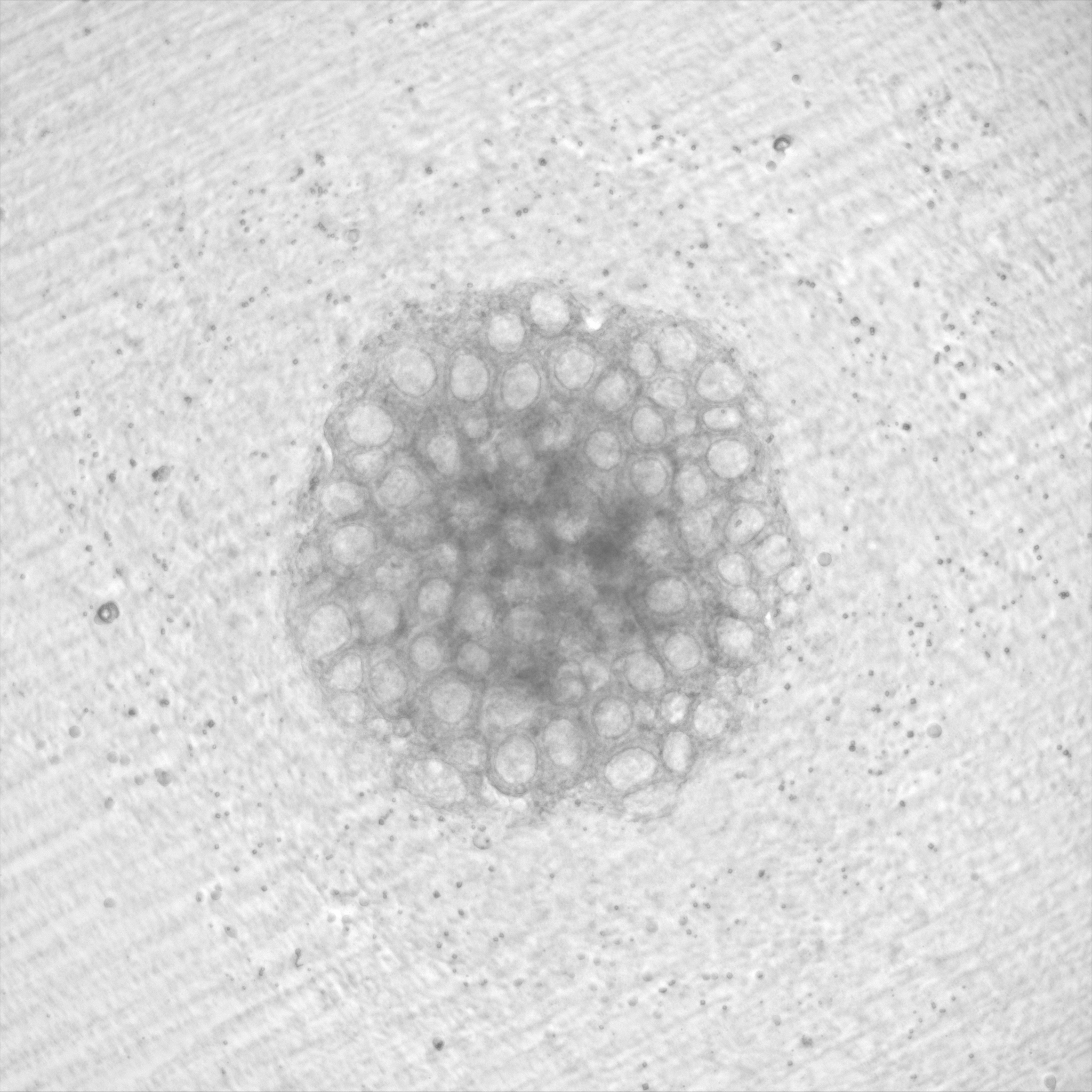

Supplement: Supplementary file 15 — Source Data for Figure 2 [file EMBJ-42-e113955-s011.zip › Figure_2/2C/Fig2C_Gonad-der_IVD_d16.tif]

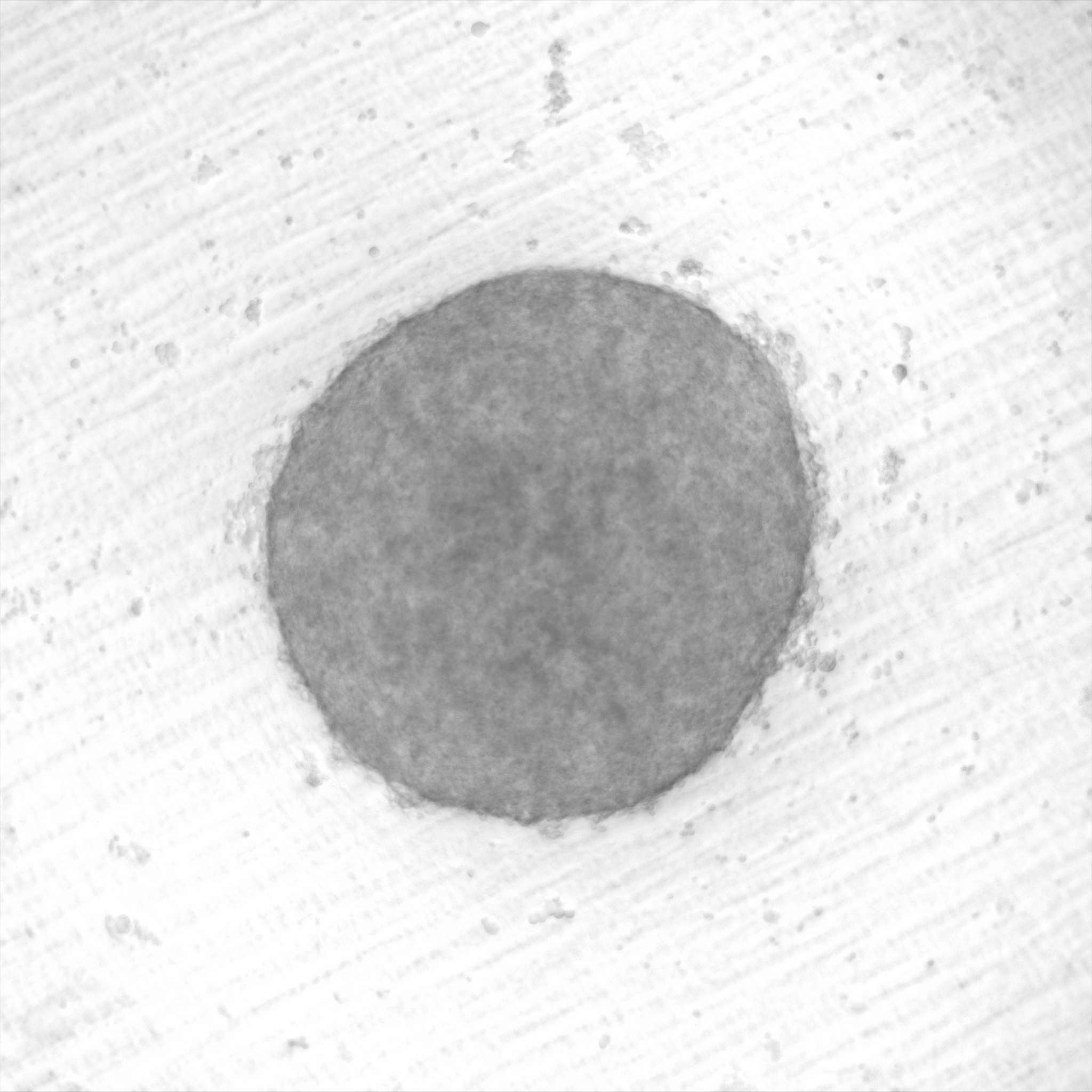

Supplement: Supplementary file 15 — Source Data for Figure 2 [file EMBJ-42-e113955-s011.zip › Figure_2/2C/Fig2C_Gonad-der_IVD_d2.tif]

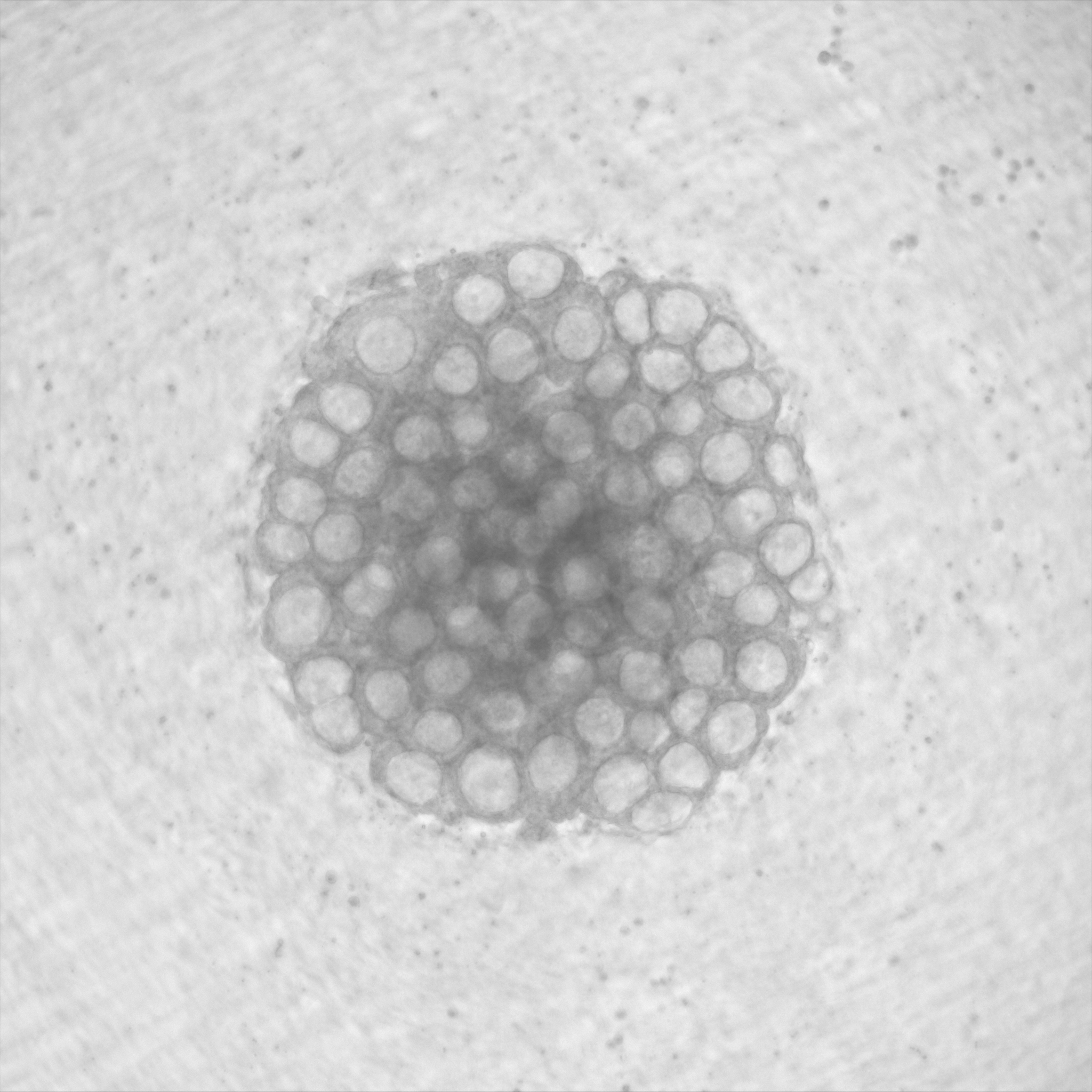

Supplement: Supplementary file 15 — Source Data for Figure 2 [file EMBJ-42-e113955-s011.zip › Figure_2/2C/Fig2C_Gonad-der_IVD_d23.tif]

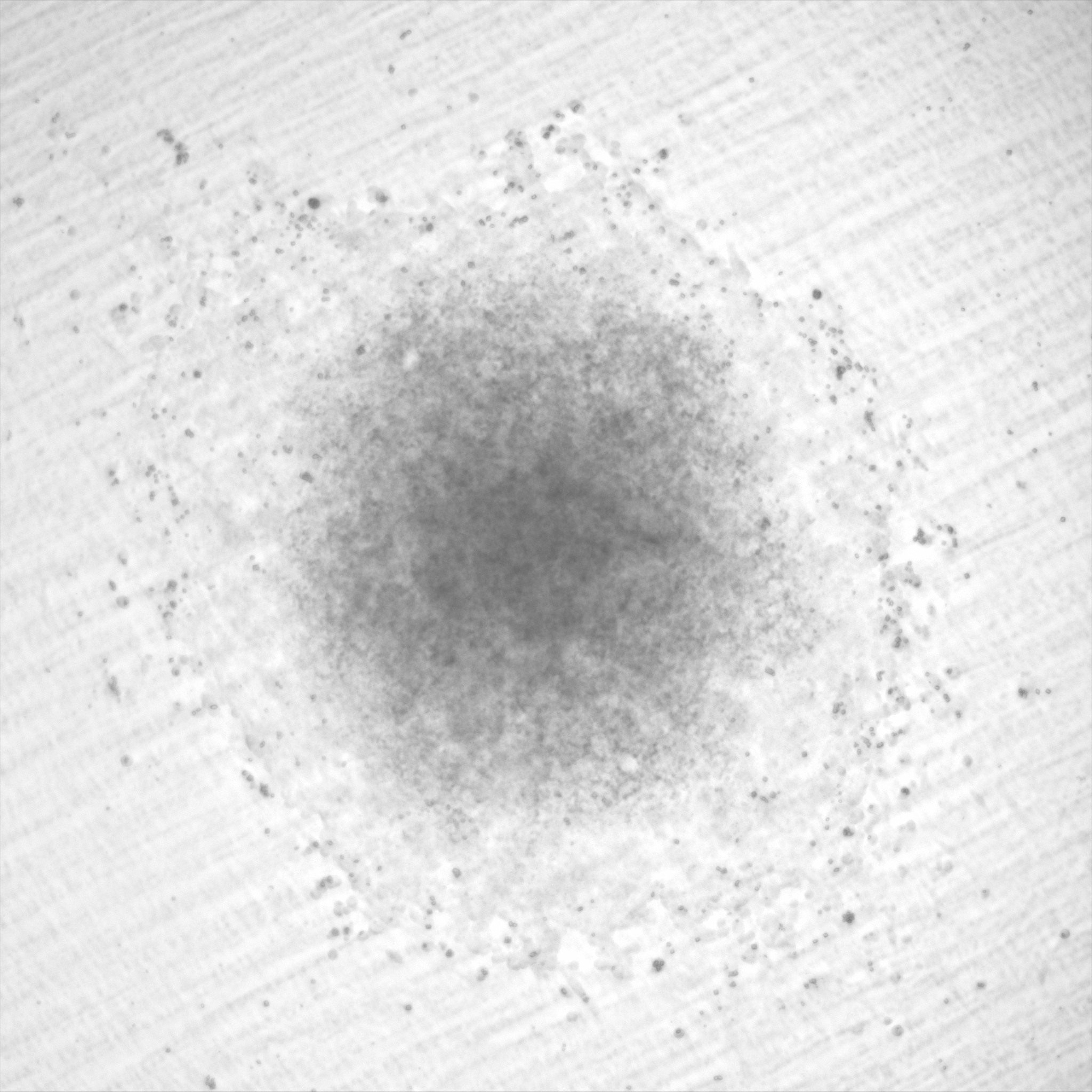

Supplement: Supplementary file 15 — Source Data for Figure 2 [file EMBJ-42-e113955-s011.zip › Figure_2/2C/Fig2C_Gonad-der_IVD_d8.tif]

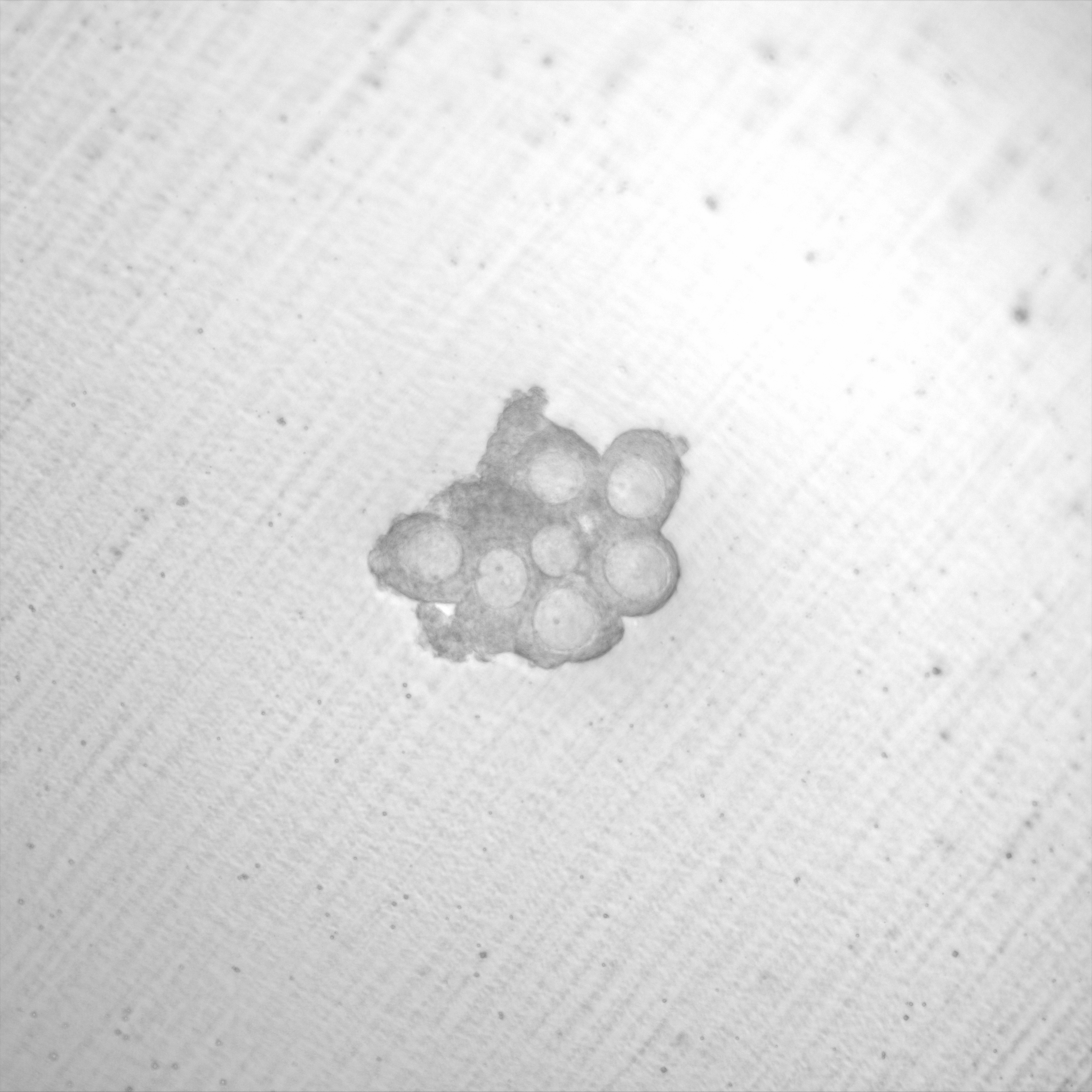

Supplement: Supplementary file 15 — Source Data for Figure 2 [file EMBJ-42-e113955-s011.zip › Figure_2/2C/Fig2C_Gonad-der_IVG_d23.tif]

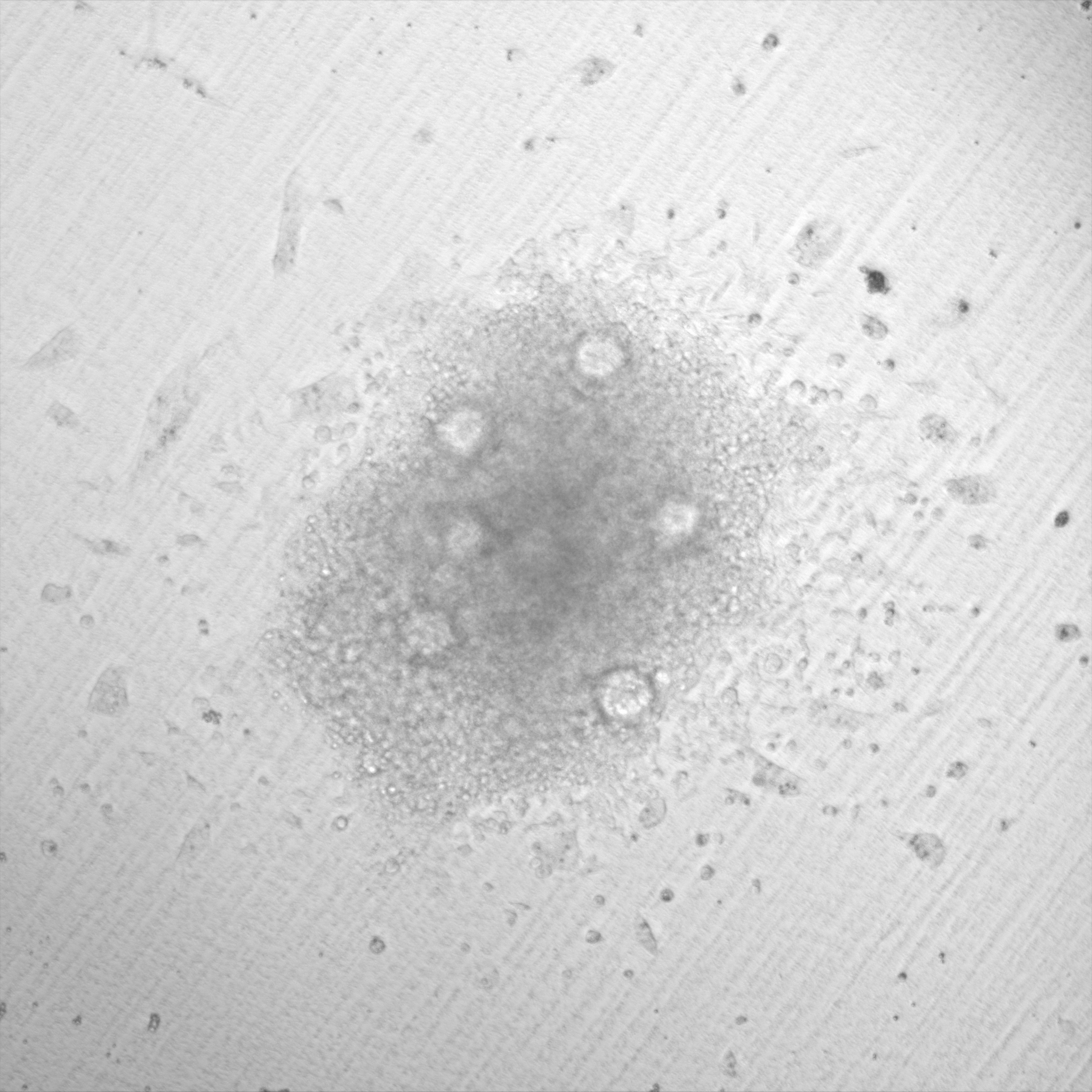

Supplement: Supplementary file 15 — Source Data for Figure 2 [file EMBJ-42-e113955-s011.zip › Figure_2/2C/Fig2C_Gonad-der_IVG_d29.tif]

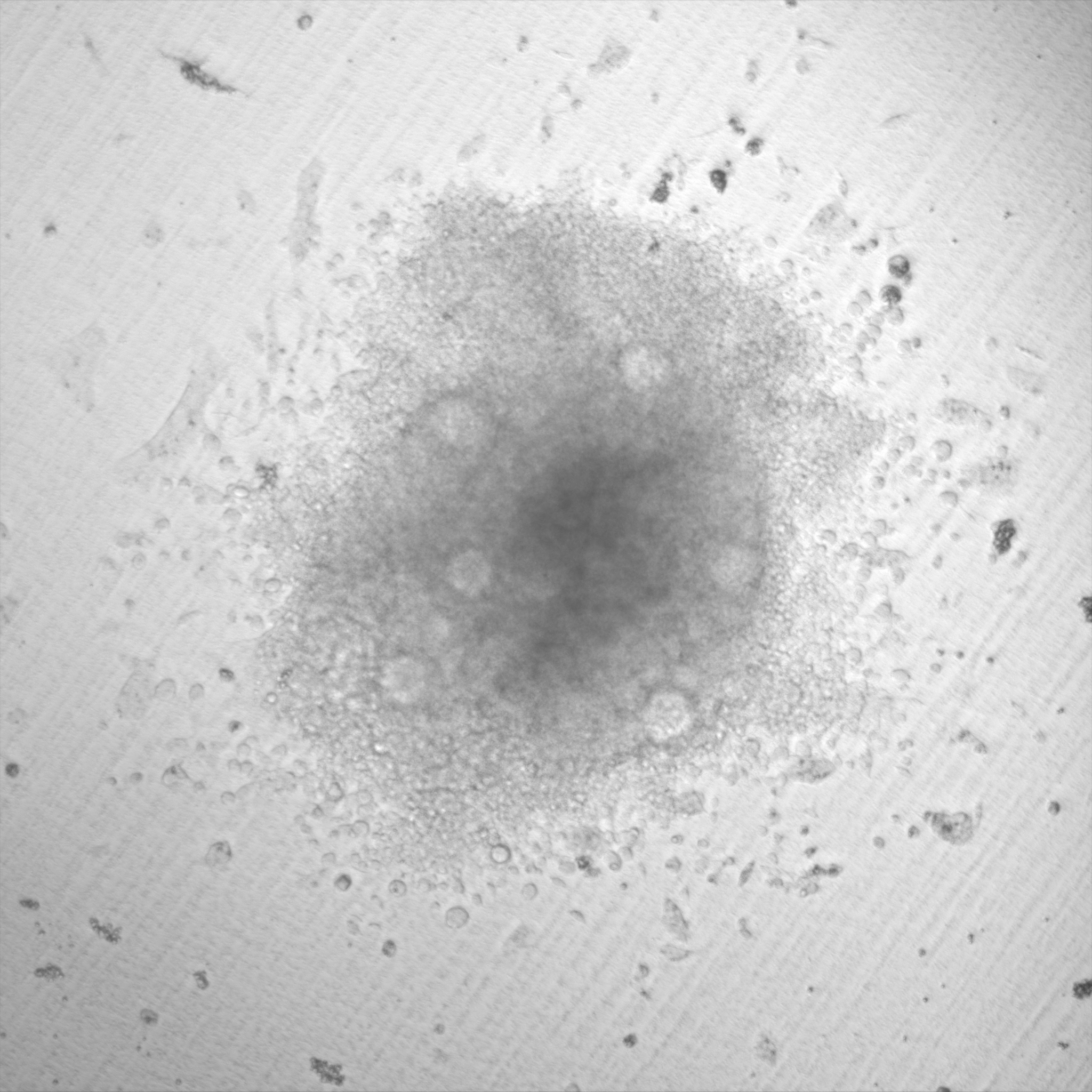

Supplement: Supplementary file 15 — Source Data for Figure 2 [file EMBJ-42-e113955-s011.zip › Figure_2/2C/Fig2C_Gonad-der_IVG_d31.tif]

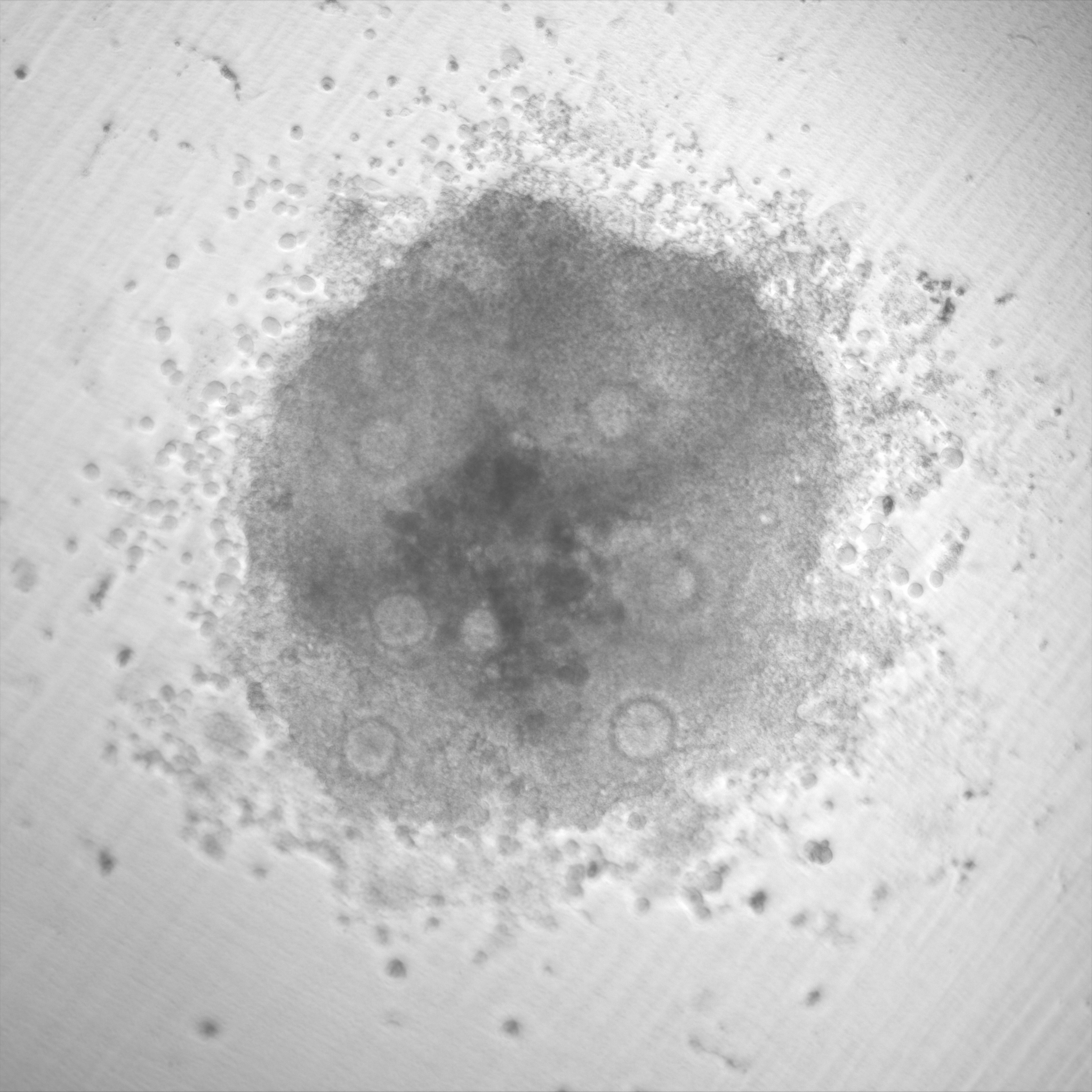

Supplement: Supplementary file 15 — Source Data for Figure 2 [file EMBJ-42-e113955-s011.zip › Figure_2/2C/Fig2C_Gonad-der_IVG_d34.tif]

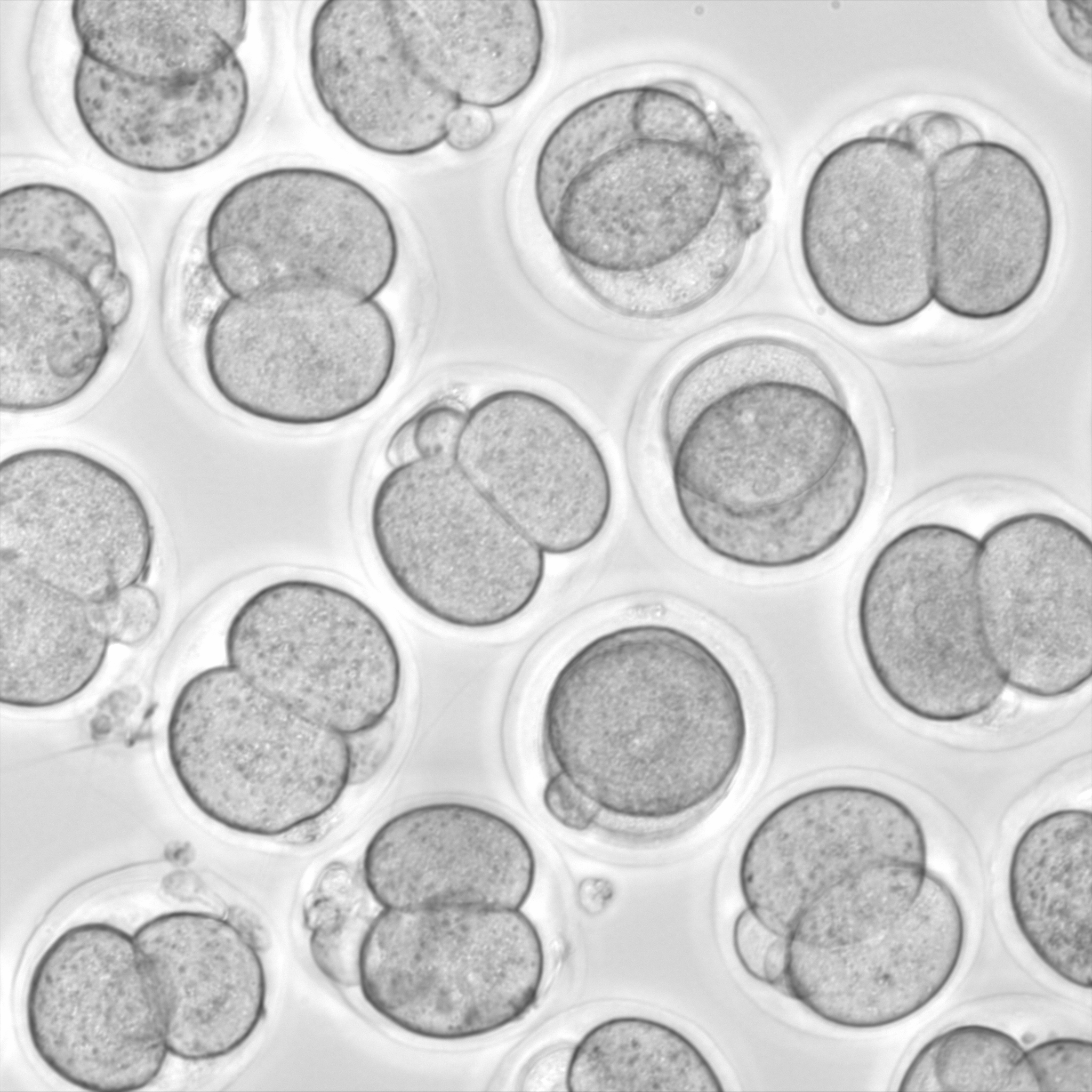

Supplement: Supplementary file 15 — Source Data for Figure 2 [file EMBJ-42-e113955-s011.zip › Figure_2/2C/Fig2C_Gonad-der_Preim_2cell.tif]

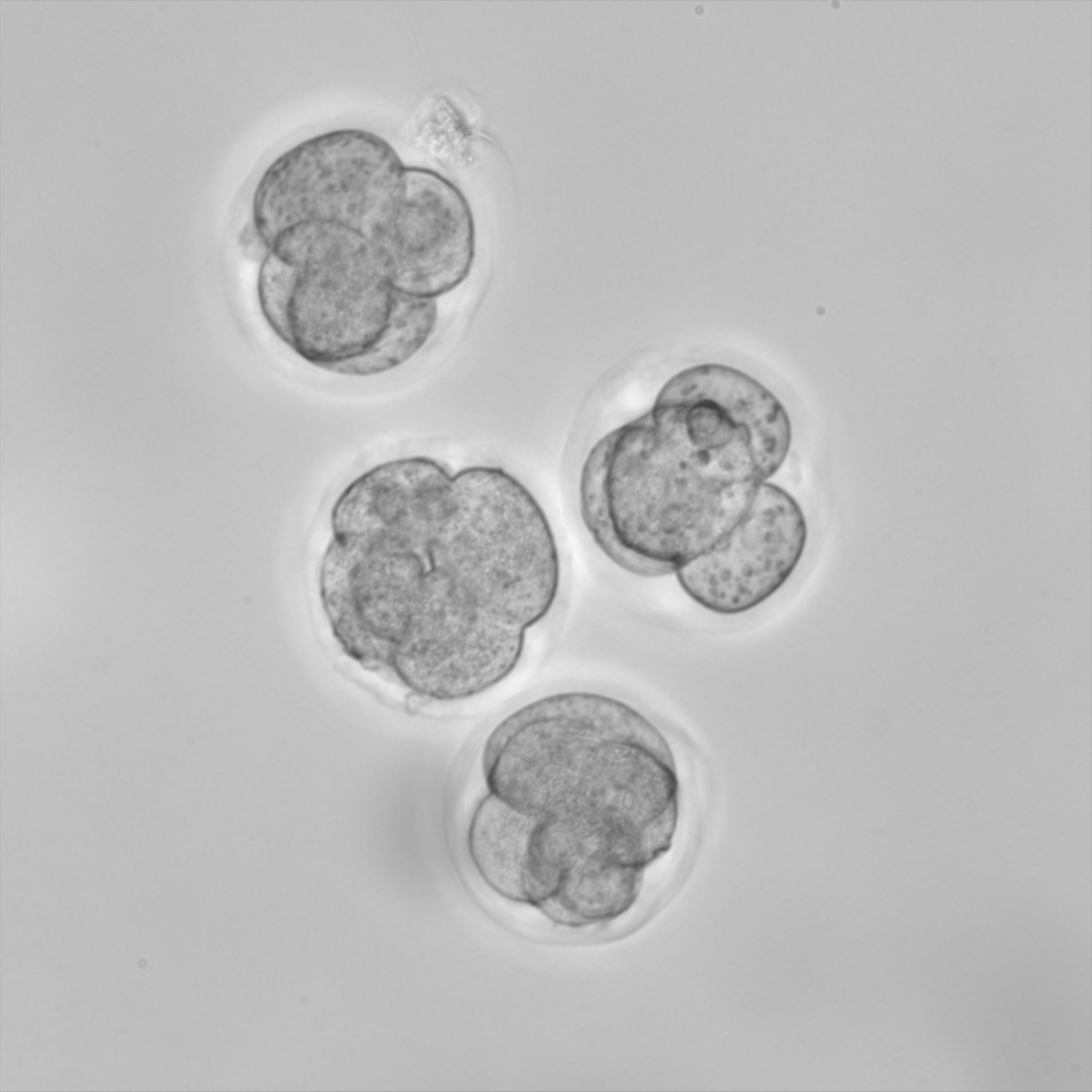

Supplement: Supplementary file 15 — Source Data for Figure 2 [file EMBJ-42-e113955-s011.zip › Figure_2/2C/Fig2C_Gonad-der_Preim_4cell.tif]

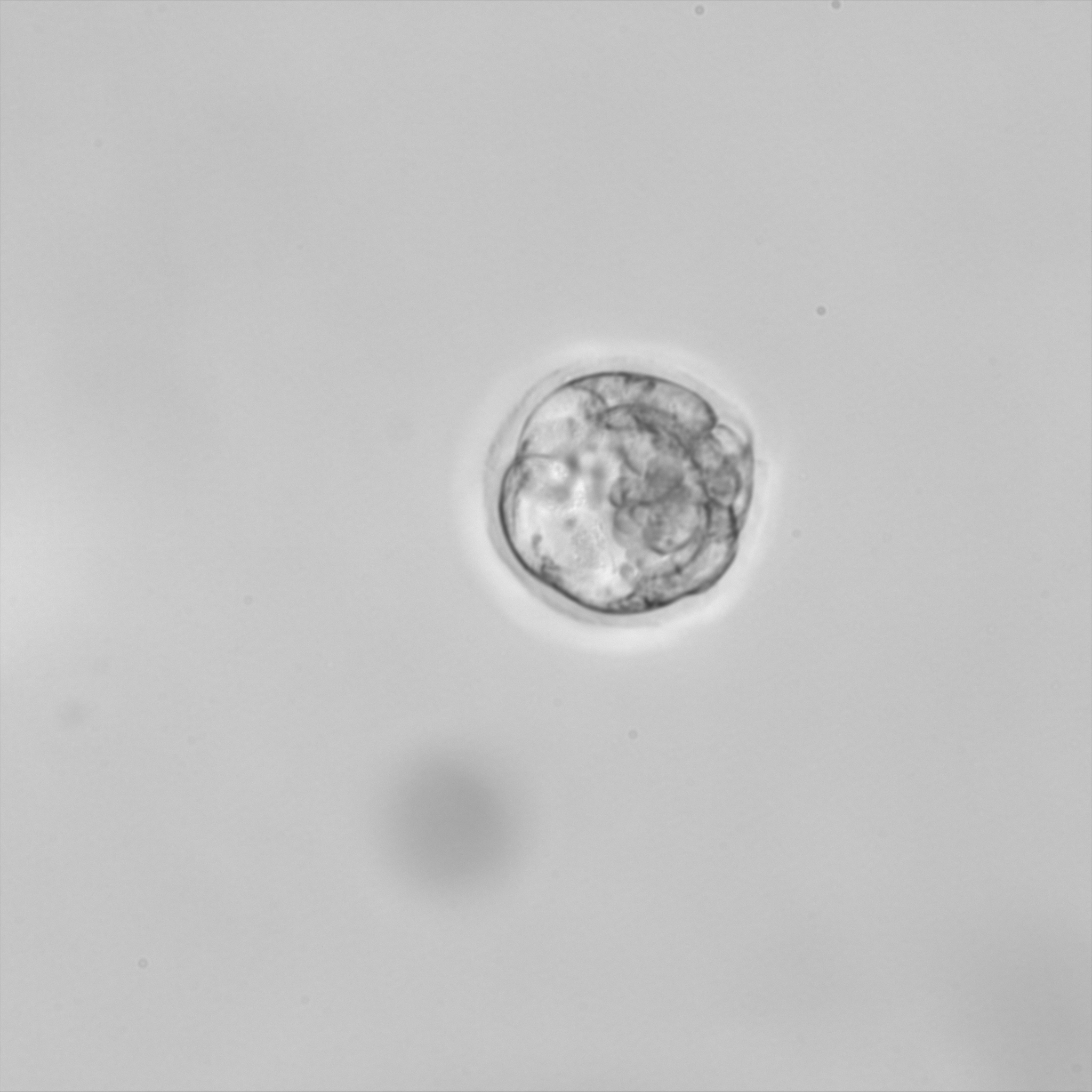

Supplement: Supplementary file 15 — Source Data for Figure 2 [file EMBJ-42-e113955-s011.zip › Figure_2/2C/Fig2C_Gonad-der_Preim_Blast.tif]

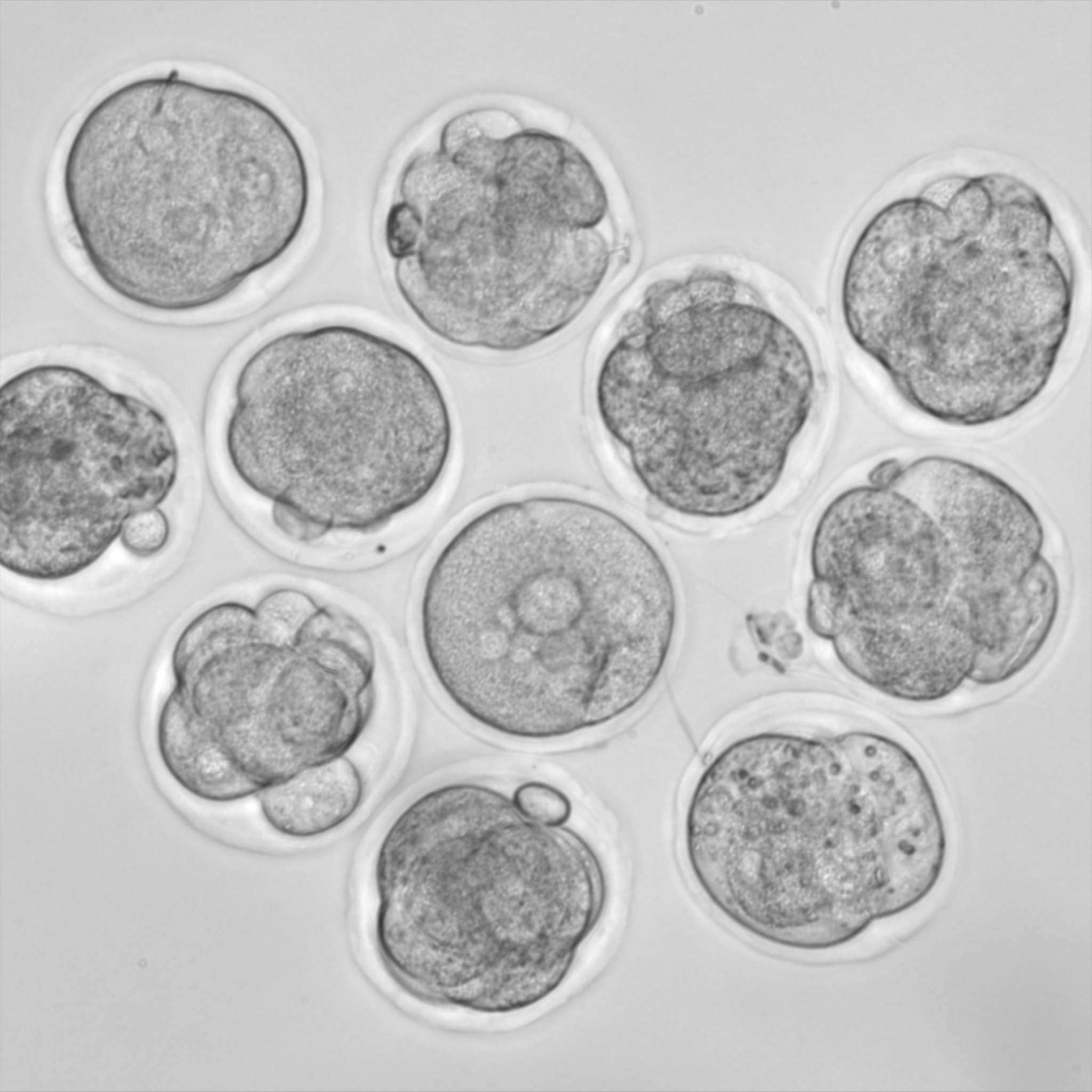

Supplement: Supplementary file 15 — Source Data for Figure 2 [file EMBJ-42-e113955-s011.zip › Figure_2/2C/Fig2C_Gonad-der_Preim_Morula.tif]

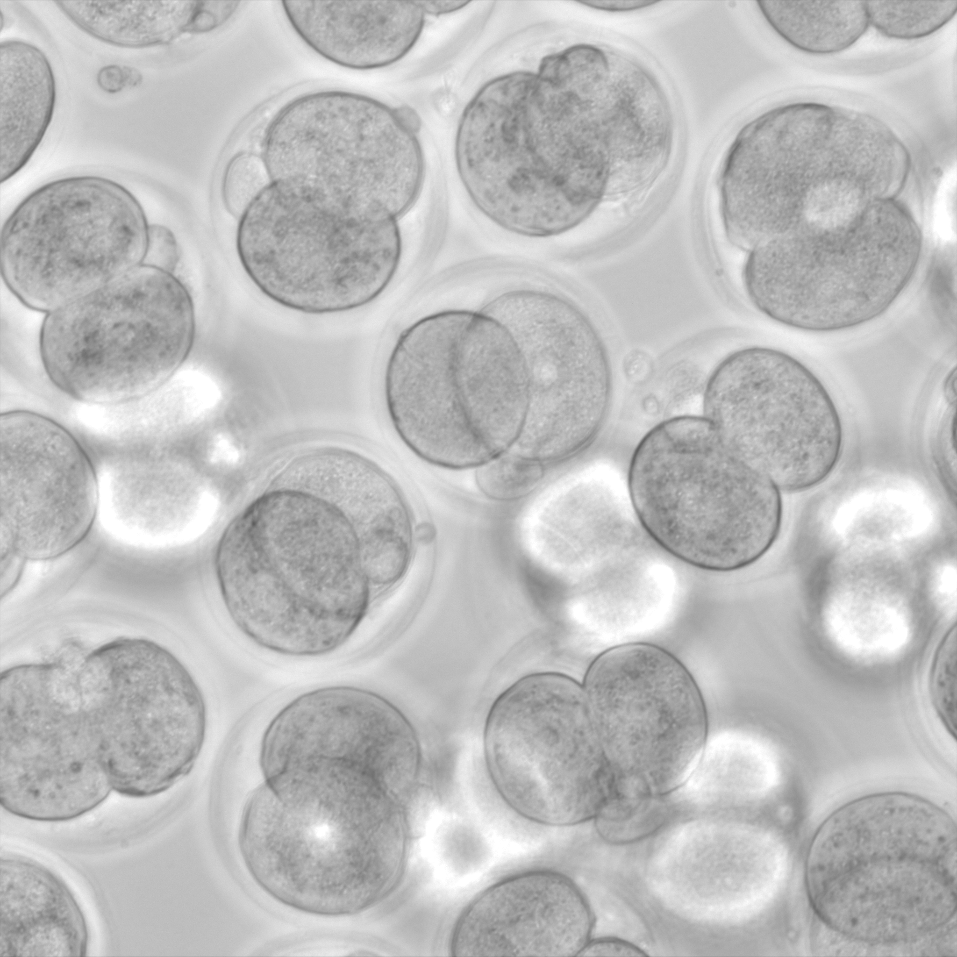

Supplement: Supplementary file 15 — Source Data for Figure 2 [file EMBJ-42-e113955-s011.zip › Figure_2/2D/Fig2D_Fol-der_2cell.tif]

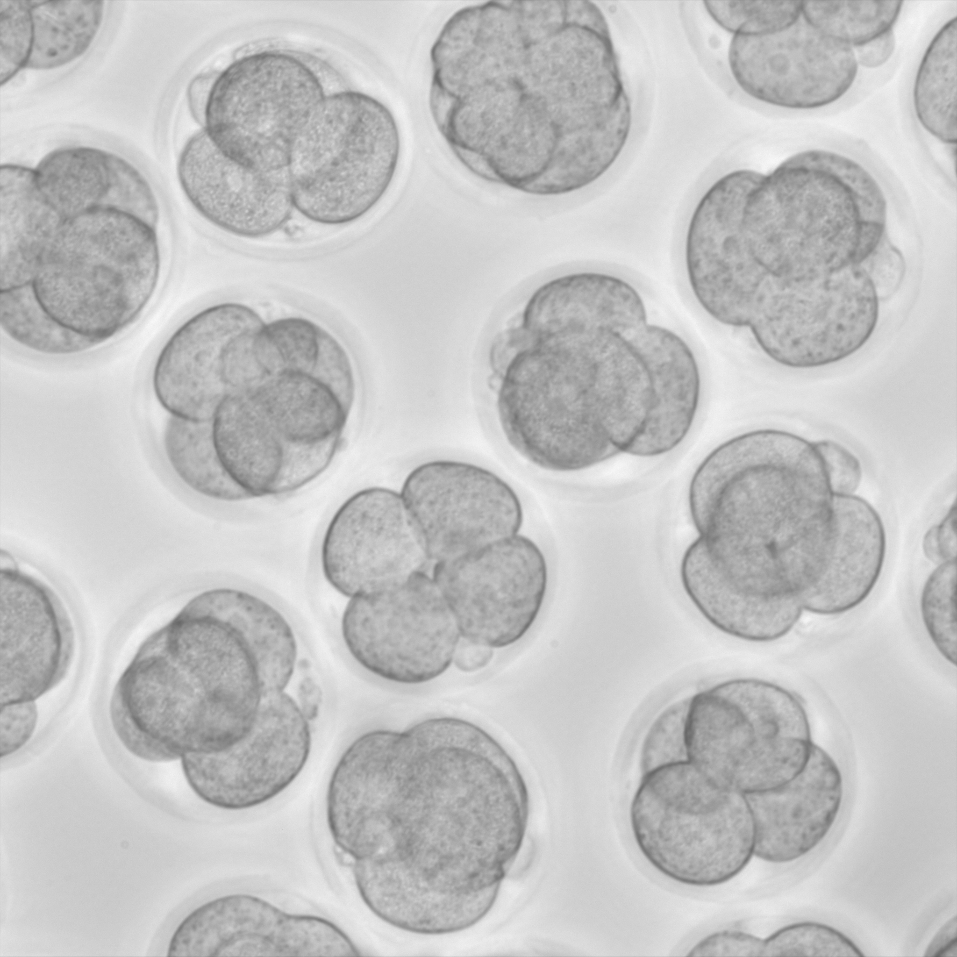

Supplement: Supplementary file 15 — Source Data for Figure 2 [file EMBJ-42-e113955-s011.zip › Figure_2/2D/Fig2D_Fol-der_4cell.tif]

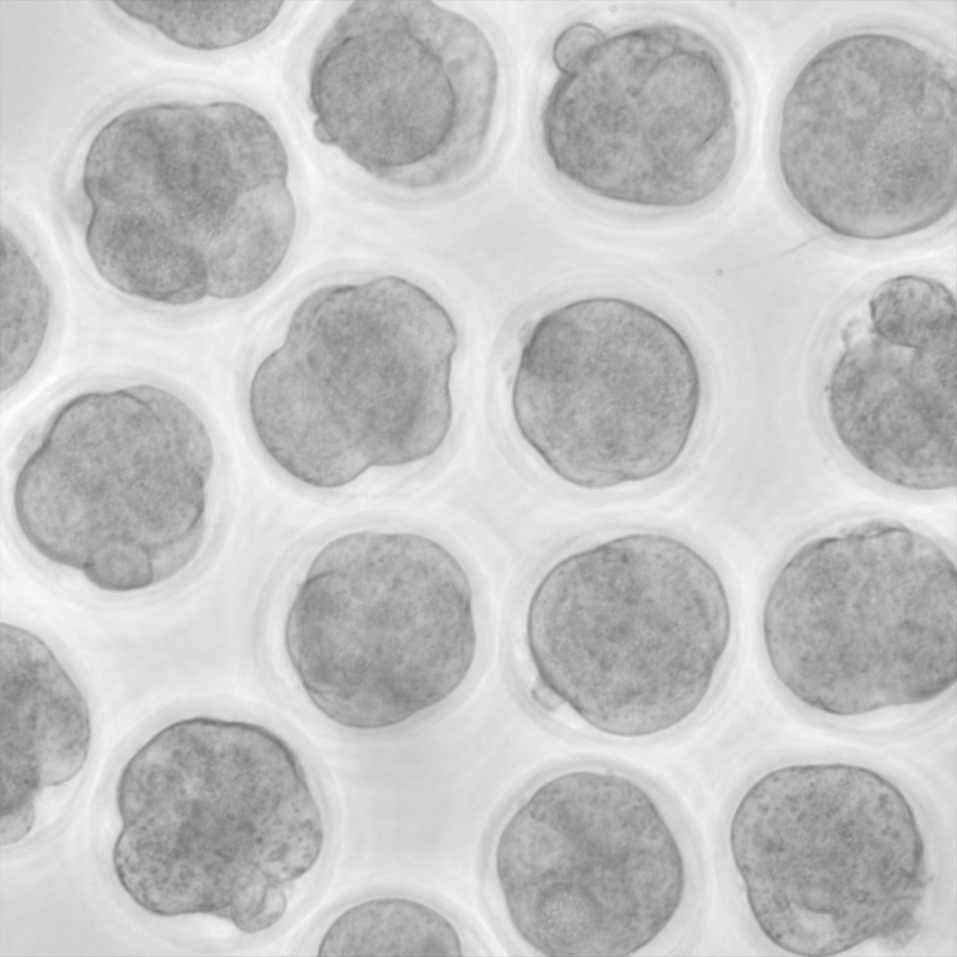

Supplement: Supplementary file 15 — Source Data for Figure 2 [file EMBJ-42-e113955-s011.zip › Figure_2/2D/Fig2D_Fol-der_8cell.tif]

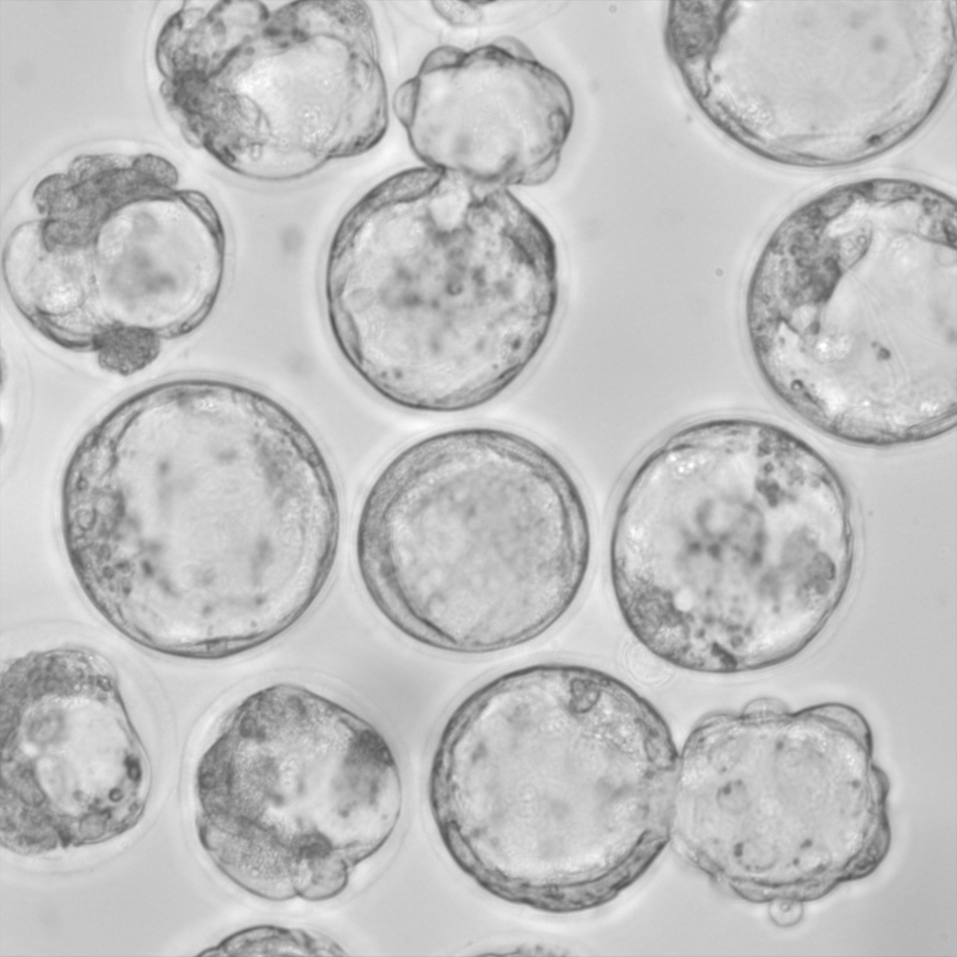

Supplement: Supplementary file 15 — Source Data for Figure 2 [file EMBJ-42-e113955-s011.zip › Figure_2/2D/Fig2D_Fol-der_Blast.tif]

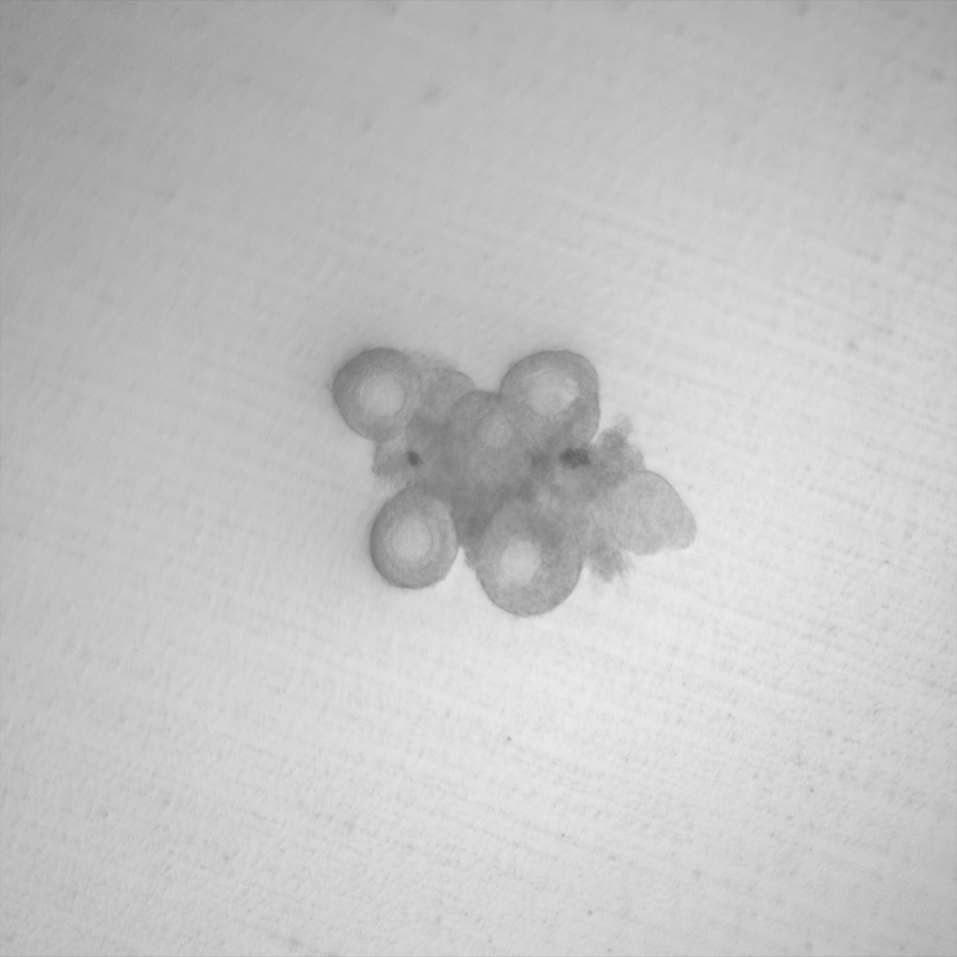

Supplement: Supplementary file 15 — Source Data for Figure 2 [file EMBJ-42-e113955-s011.zip › Figure_2/2D/Fig2D_Fol-der_IVG-d0.tif]

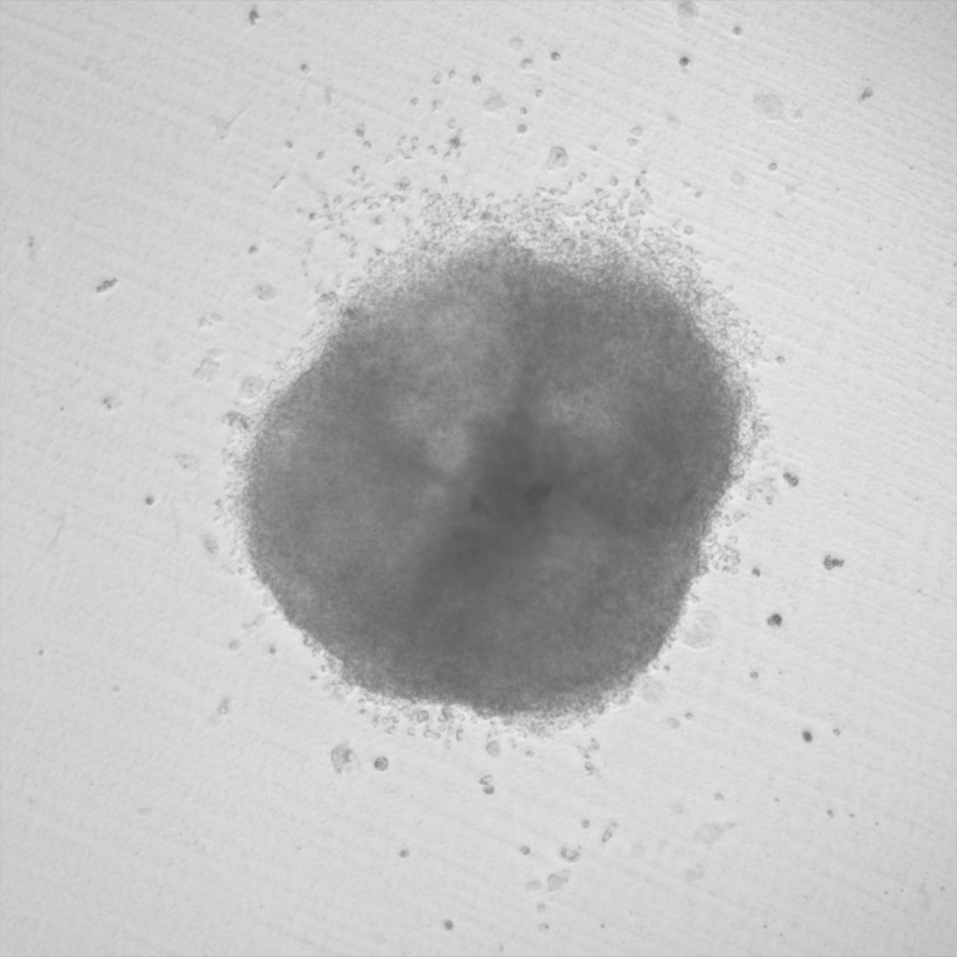

Supplement: Supplementary file 15 — Source Data for Figure 2 [file EMBJ-42-e113955-s011.zip › Figure_2/2D/Fig2D_Fol-der_IVG-d12.tif]

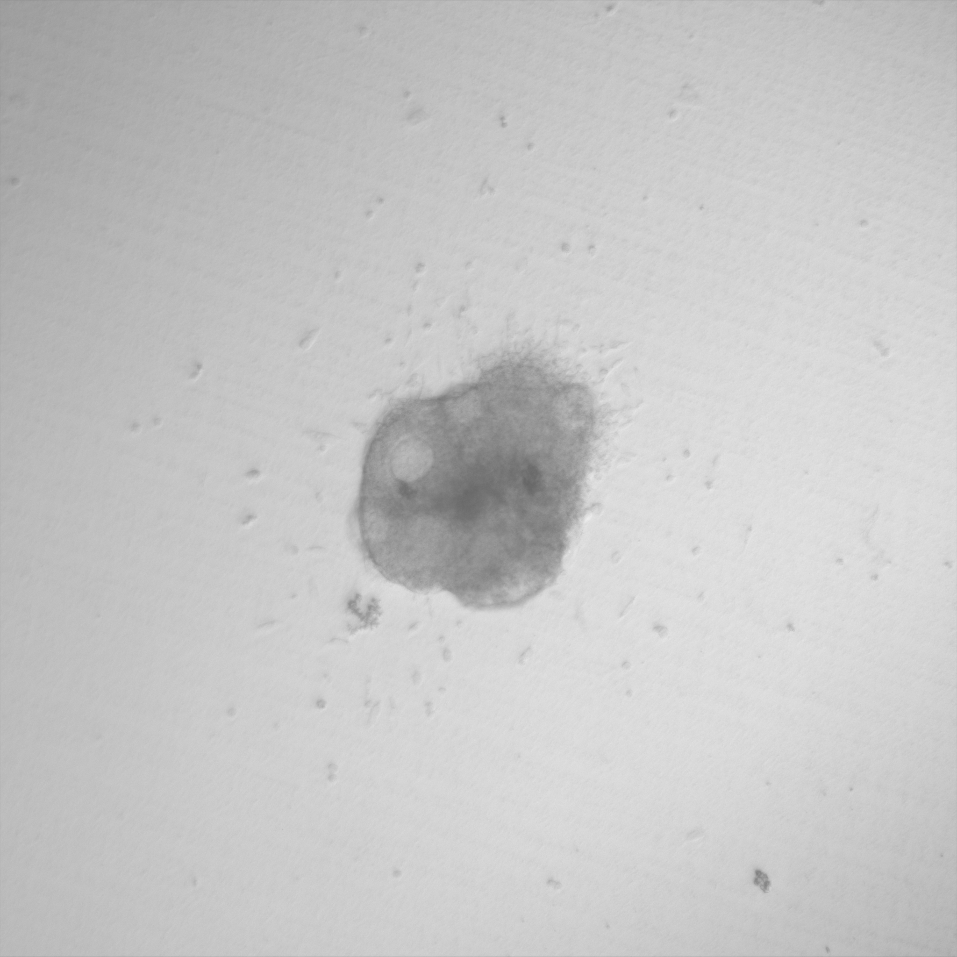

Supplement: Supplementary file 15 — Source Data for Figure 2 [file EMBJ-42-e113955-s011.zip › Figure_2/2D/Fig2D_Fol-der_IVG-d4.tif]

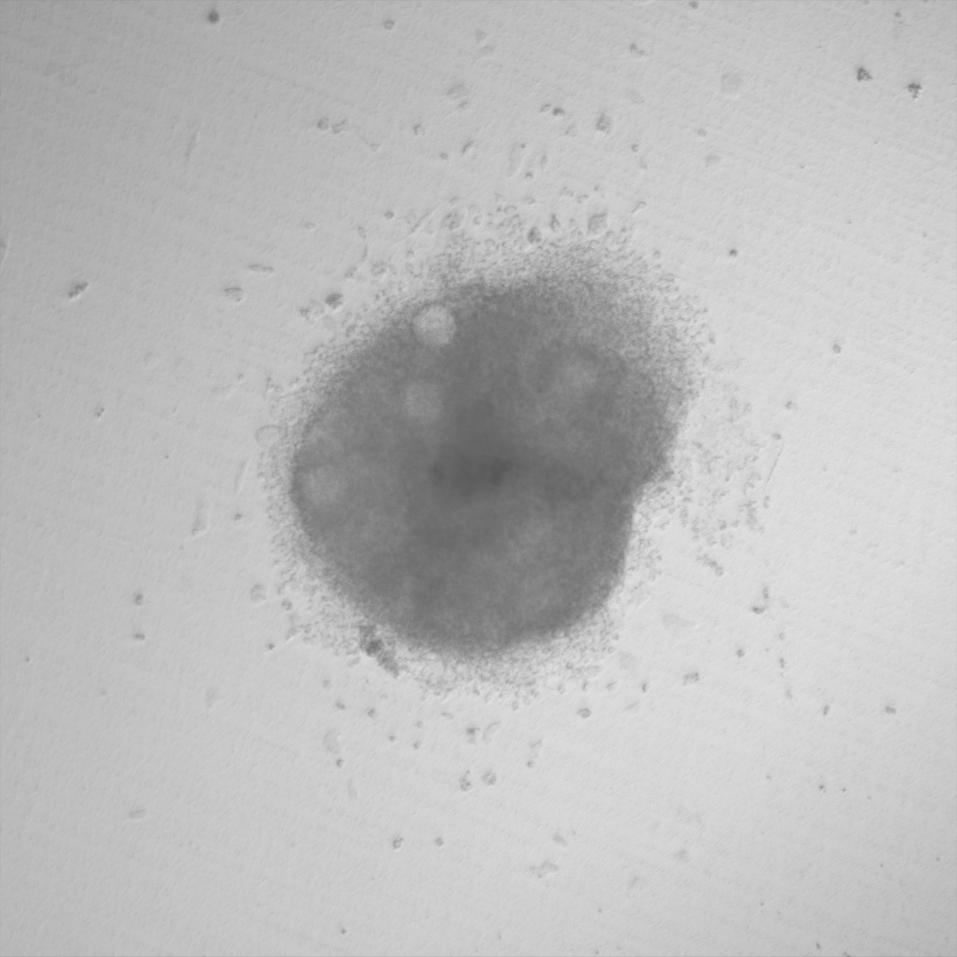

Supplement: Supplementary file 15 — Source Data for Figure 2 [file EMBJ-42-e113955-s011.zip › Figure_2/2D/Fig2D_Fol-der_IVG-d8.tif]

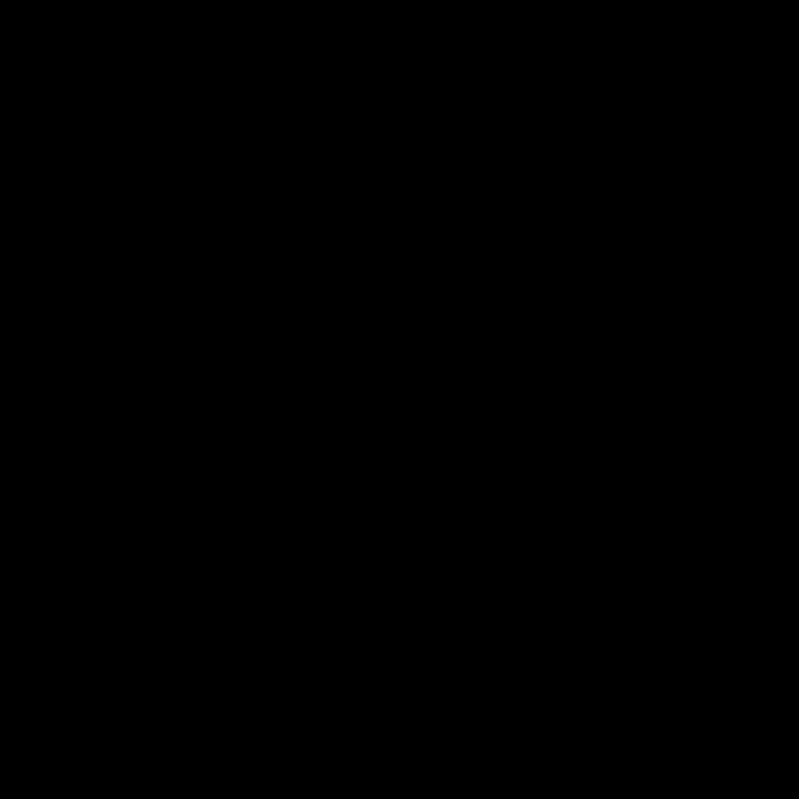

Supplement: Supplementary file 17 — Source Data for Figure 4 [file EMBJ-42-e113955-s009.zip › Figure_4/4A/Fig4A_iPSC-bottom_BrUTP.tif]

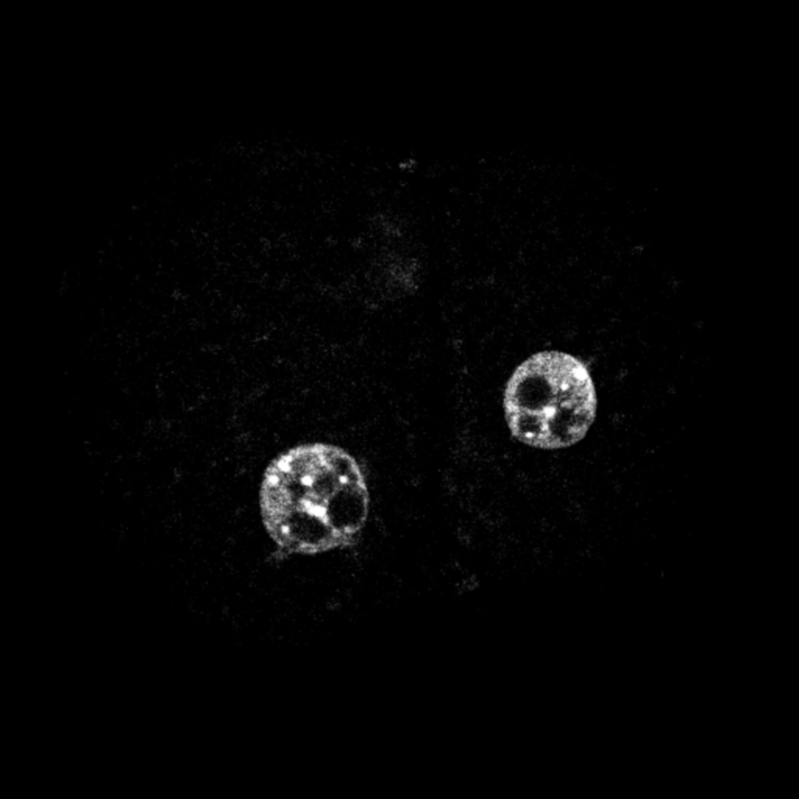

Supplement: Supplementary file 17 — Source Data for Figure 4 [file EMBJ-42-e113955-s009.zip › Figure_4/4A/Fig4A_iPSC-bottom_DAPI.tif]

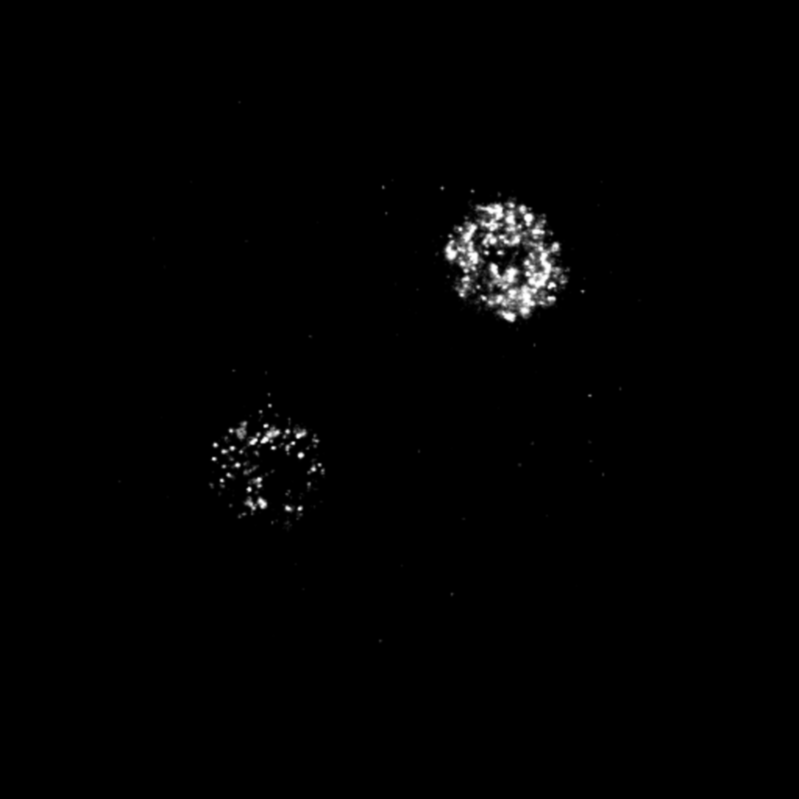

Supplement: Supplementary file 17 — Source Data for Figure 4 [file EMBJ-42-e113955-s009.zip › Figure_4/4A/Fig4A_iPSC-top_BrUTP.tif]

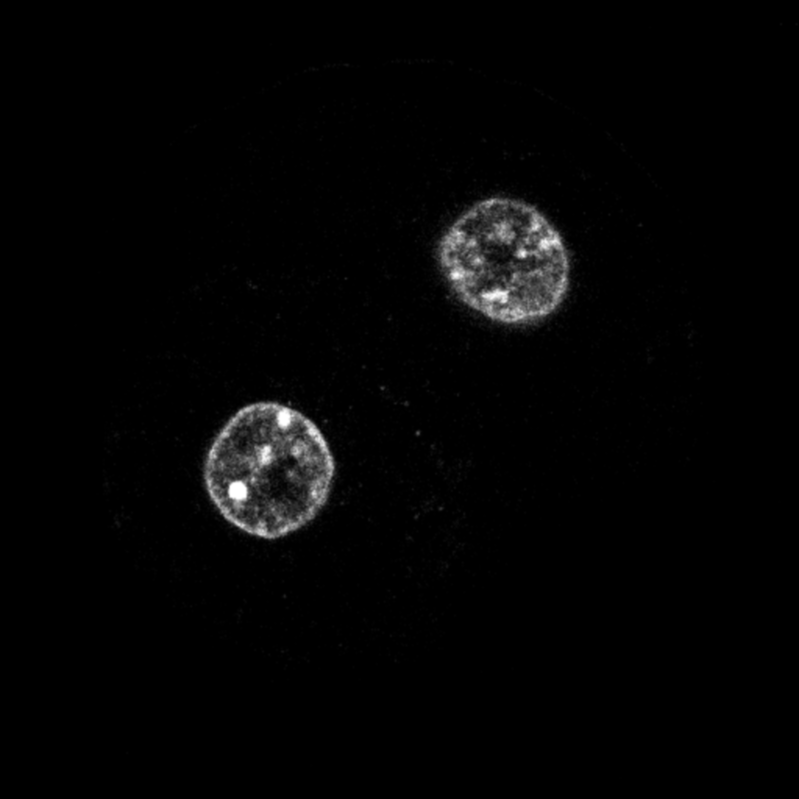

Supplement: Supplementary file 17 — Source Data for Figure 4 [file EMBJ-42-e113955-s009.zip › Figure_4/4A/Fig4A_iPSC-top_DAPI.tif]

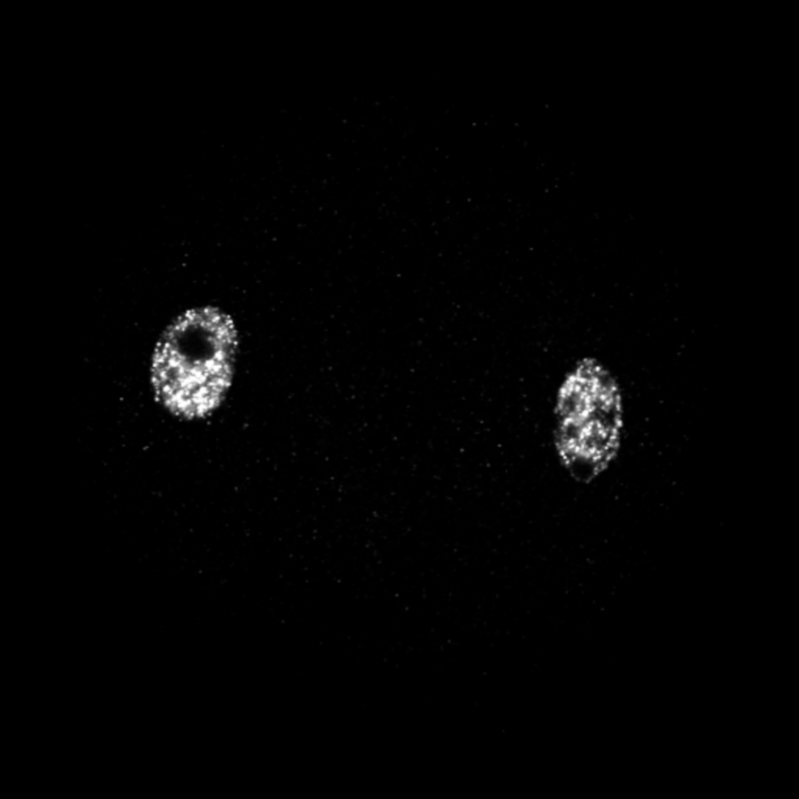

Supplement: Supplementary file 17 — Source Data for Figure 4 [file EMBJ-42-e113955-s009.zip › Figure_4/4A/Fig4A_vivo_BrUTP.tif]

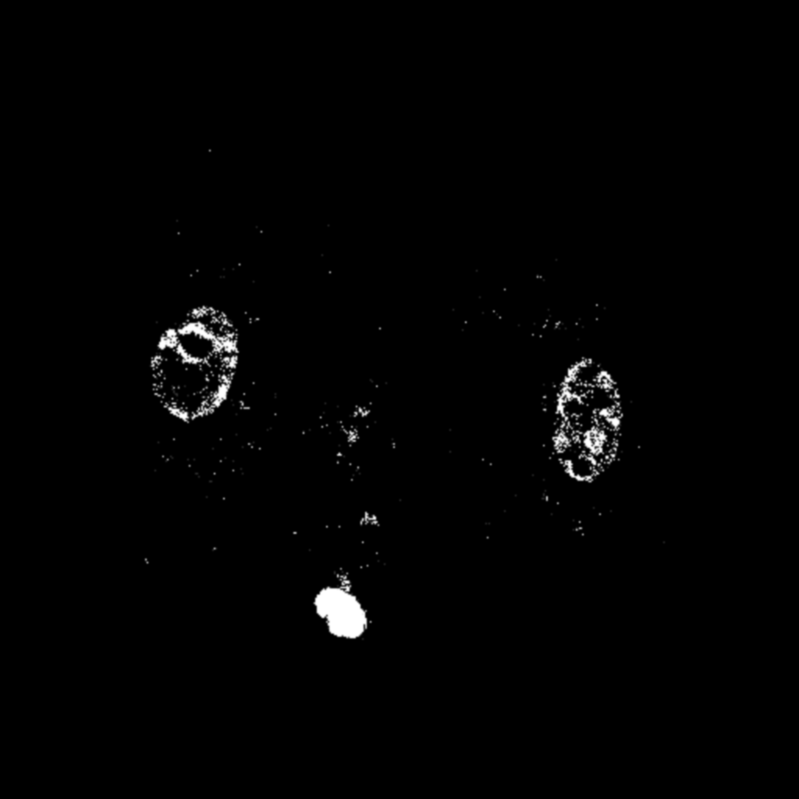

Supplement: Supplementary file 17 — Source Data for Figure 4 [file EMBJ-42-e113955-s009.zip › Figure_4/4A/Fig4A_vivo_DAPI.tif]

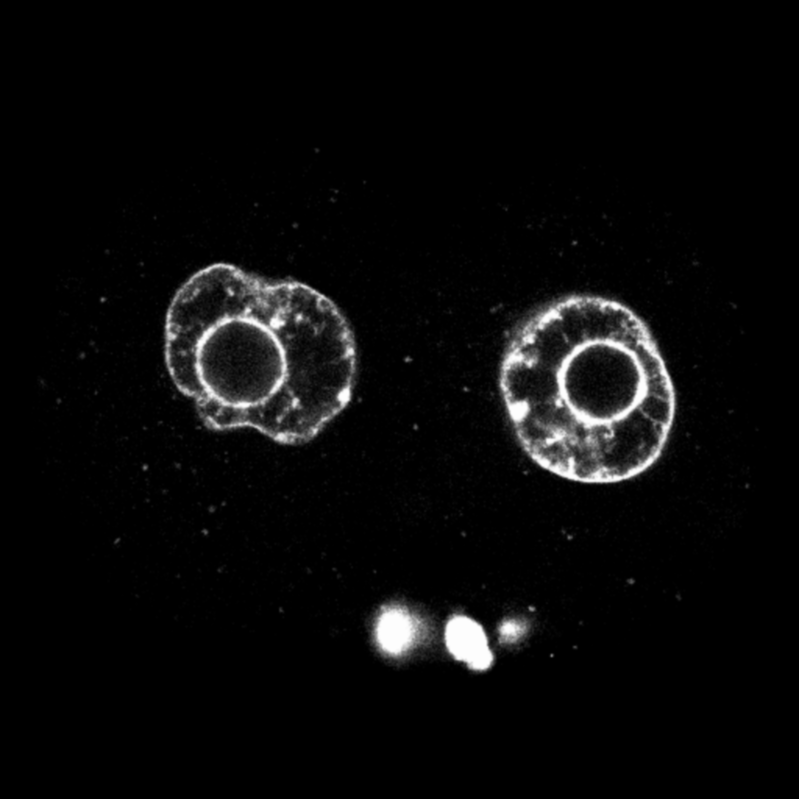

Supplement: Supplementary file 17 — Source Data for Figure 4 [file EMBJ-42-e113955-s009.zip › Figure_4/4C/Fig4C_iPSC-bottom_DAPI.tif]

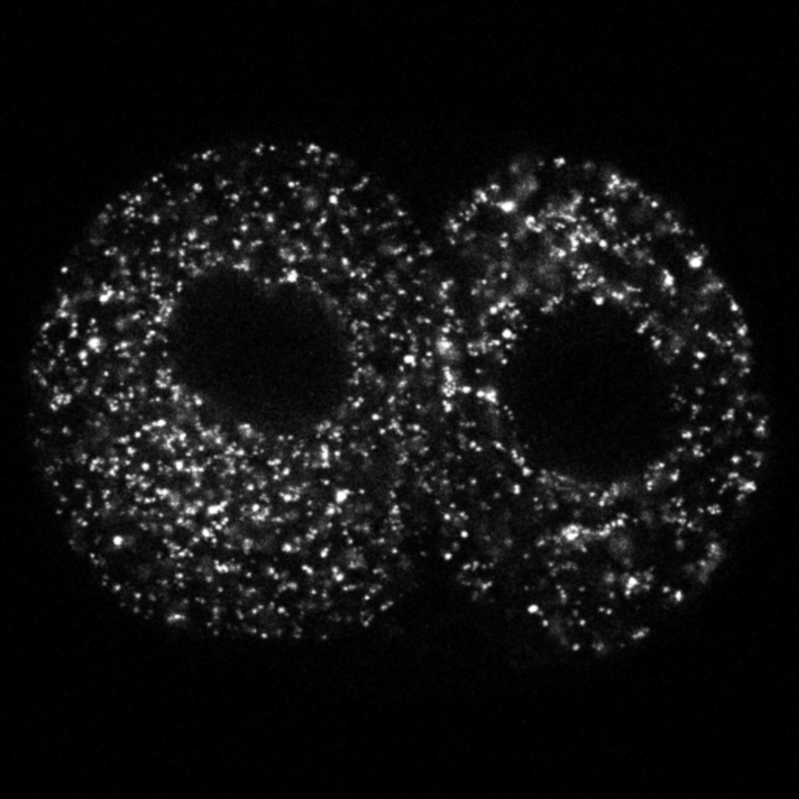

Supplement: Supplementary file 17 — Source Data for Figure 4 [file EMBJ-42-e113955-s009.zip › Figure_4/4C/Fig4C_iPSC-bottom_pPDH.tif]

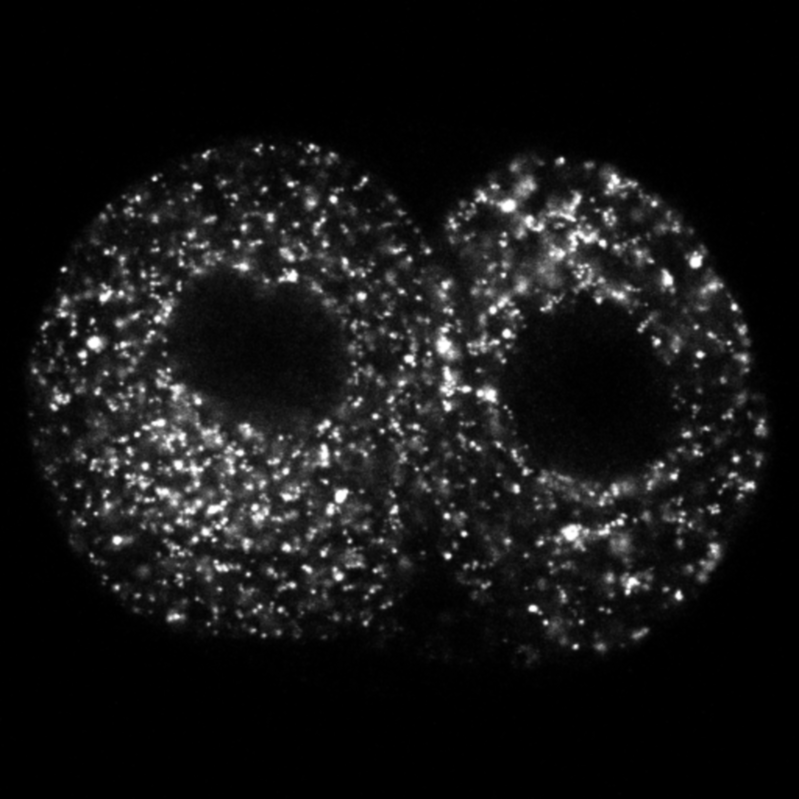

Supplement: Supplementary file 17 — Source Data for Figure 4 [file EMBJ-42-e113955-s009.zip › Figure_4/4C/Fig4C_iPSC-bottom_TotalPDH.tif]

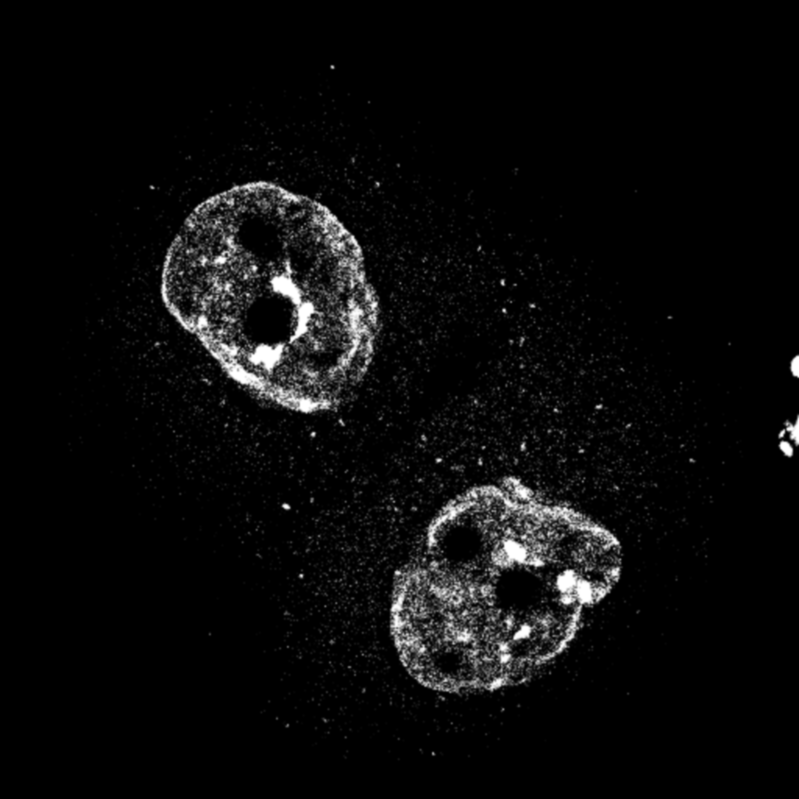

Supplement: Supplementary file 17 — Source Data for Figure 4 [file EMBJ-42-e113955-s009.zip › Figure_4/4C/Fig4C_iPSC-top_DAPI.tif]

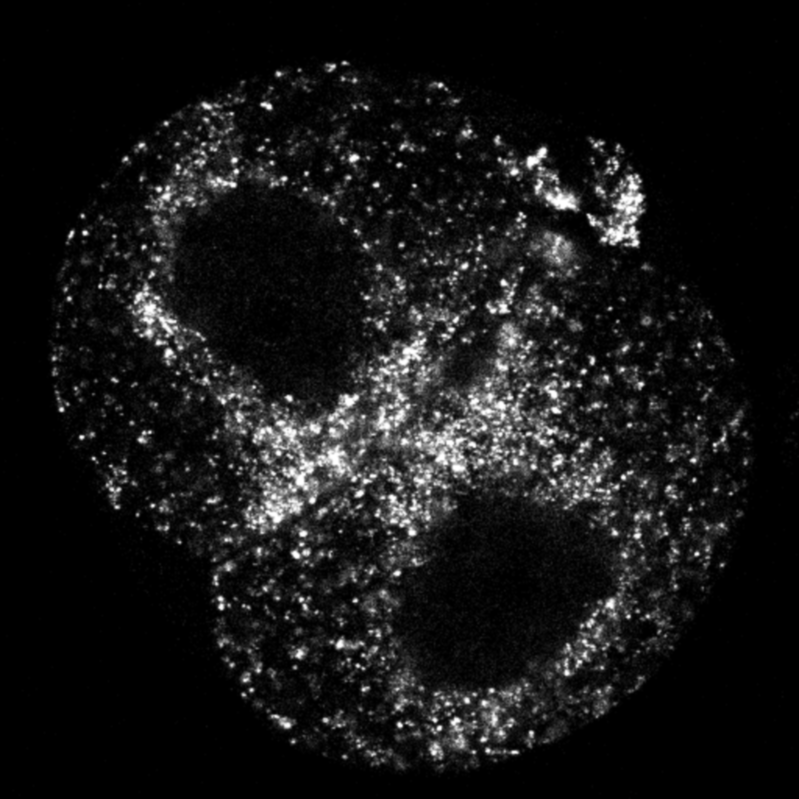

Supplement: Supplementary file 17 — Source Data for Figure 4 [file EMBJ-42-e113955-s009.zip › Figure_4/4C/Fig4C_iPSC-top_pPDH.tif]

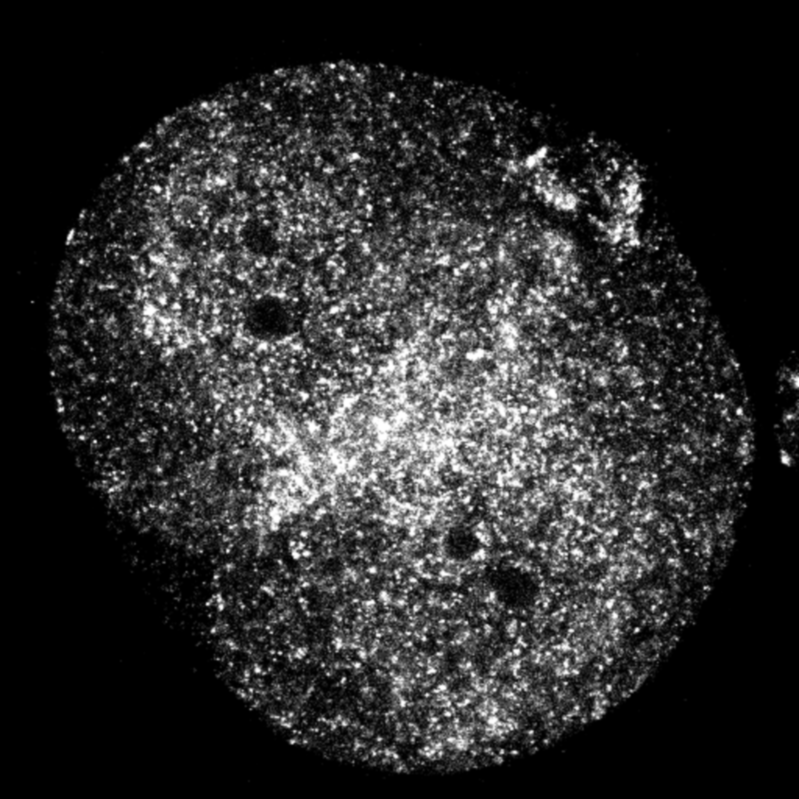

Supplement: Supplementary file 17 — Source Data for Figure 4 [file EMBJ-42-e113955-s009.zip › Figure_4/4C/Fig4C_iPSC-top_TotalPDH.tif]

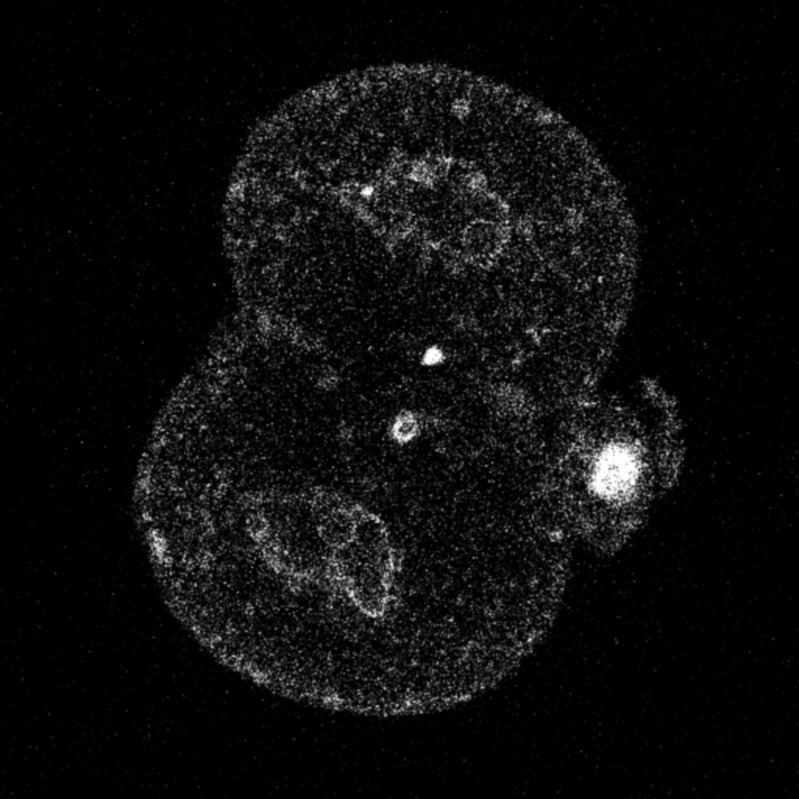

Supplement: Supplementary file 17 — Source Data for Figure 4 [file EMBJ-42-e113955-s009.zip › Figure_4/4C/Fig4C_vivo_DAPI.tif]

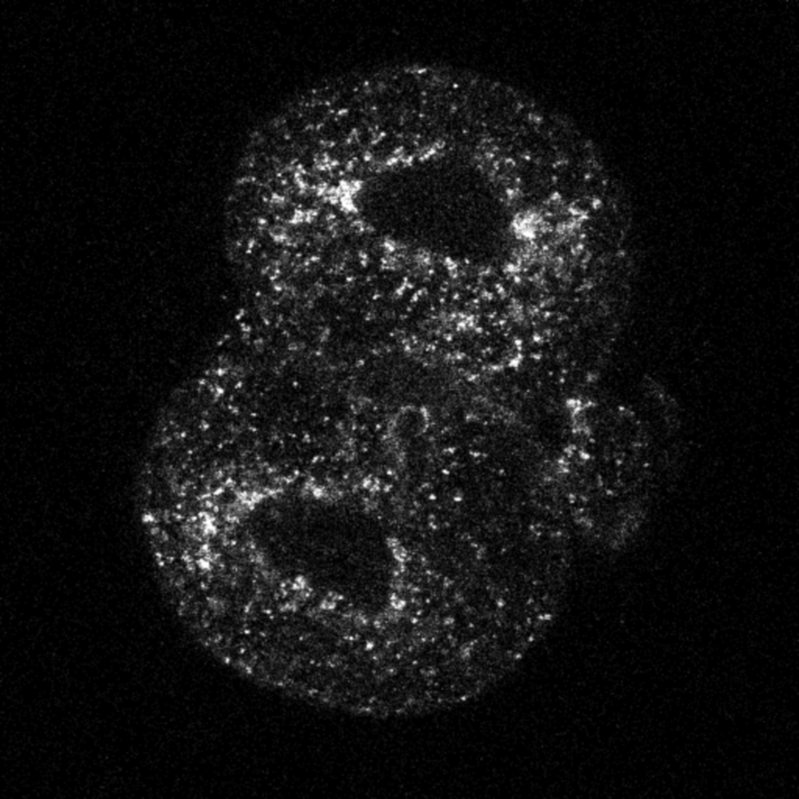

Supplement: Supplementary file 17 — Source Data for Figure 4 [file EMBJ-42-e113955-s009.zip › Figure_4/4C/Fig4C_vivo_pPDH.tif]

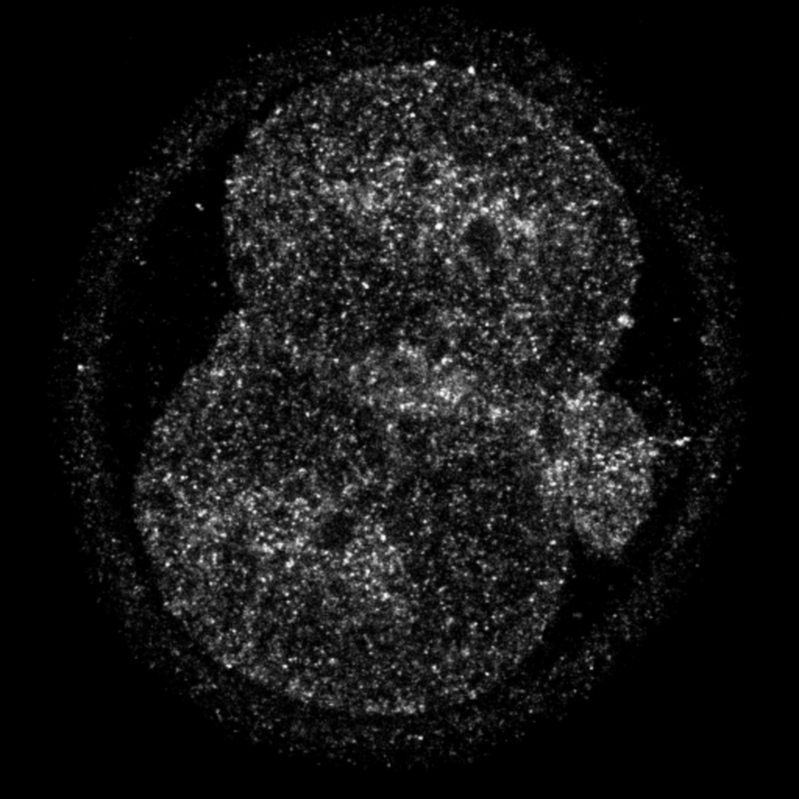

Supplement: Supplementary file 17 — Source Data for Figure 4 [file EMBJ-42-e113955-s009.zip › Figure_4/4C/Fig4C_vivo_TotalPDH.tif]

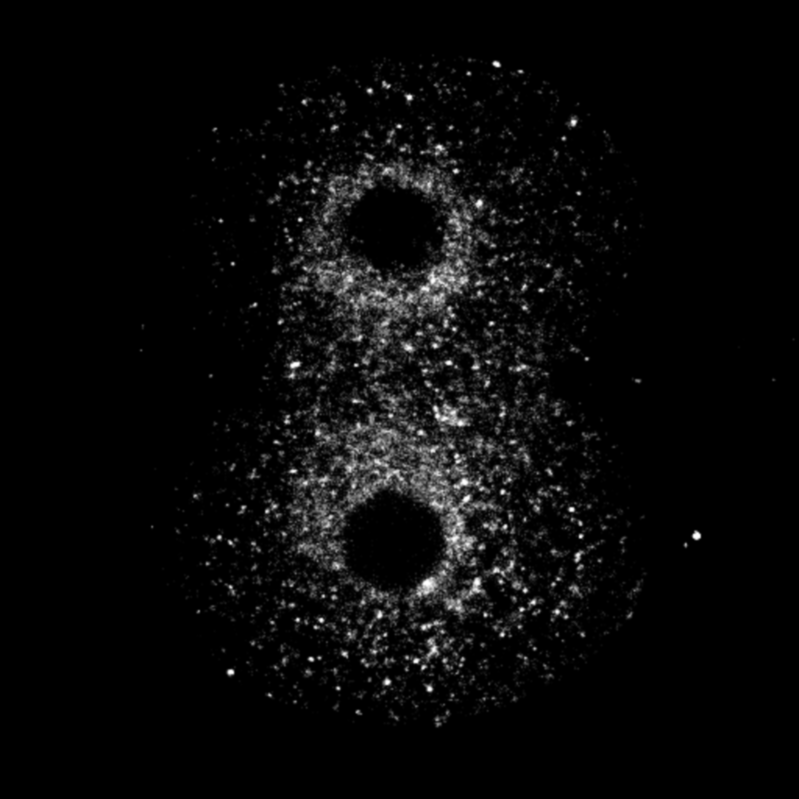

Supplement: Supplementary file 17 — Source Data for Figure 4 [file EMBJ-42-e113955-s009.zip › Figure_4/4E/Fig4E_iPSC_5hmC.tif]

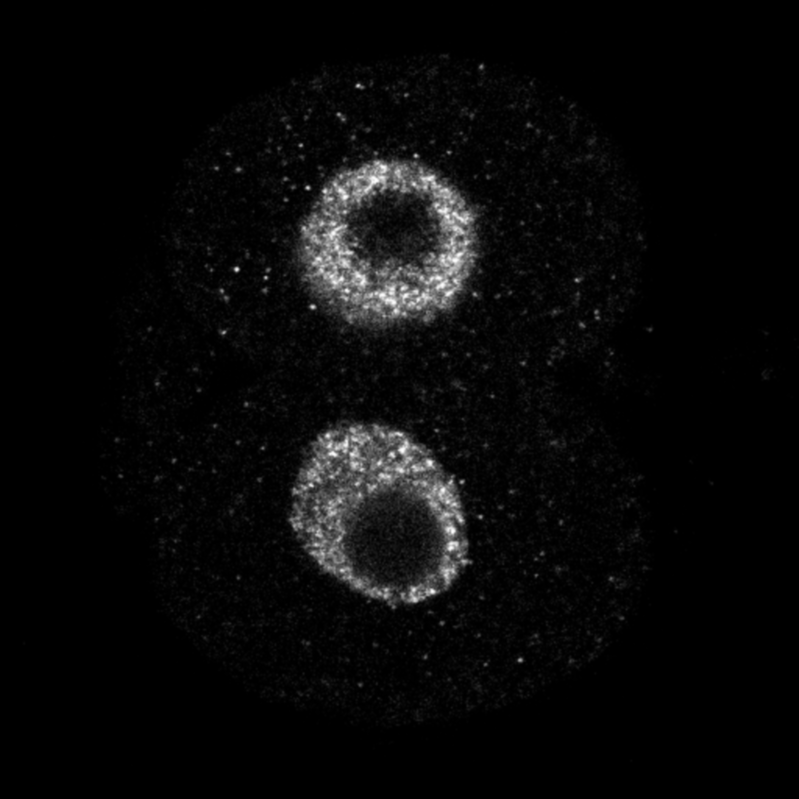

Supplement: Supplementary file 17 — Source Data for Figure 4 [file EMBJ-42-e113955-s009.zip › Figure_4/4E/Fig4E_iPSC_5mC.tif]

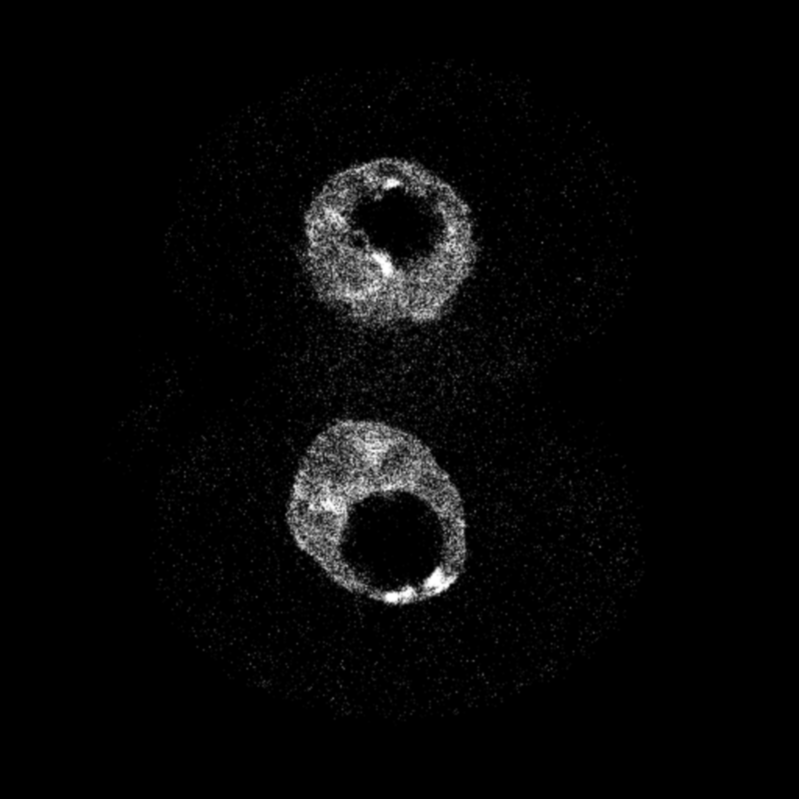

Supplement: Supplementary file 17 — Source Data for Figure 4 [file EMBJ-42-e113955-s009.zip › Figure_4/4E/Fig4E_iPSC_DAPI.tif]

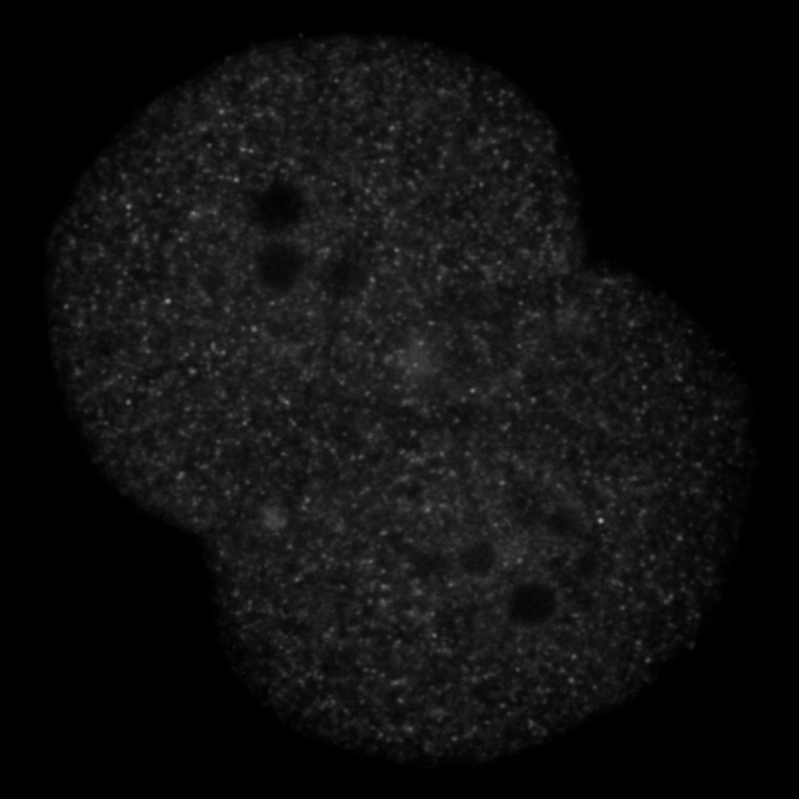

Supplement: Supplementary file 17 — Source Data for Figure 4 [file EMBJ-42-e113955-s009.zip › Figure_4/4E/Fig4E_vivo_5hmC.tif]

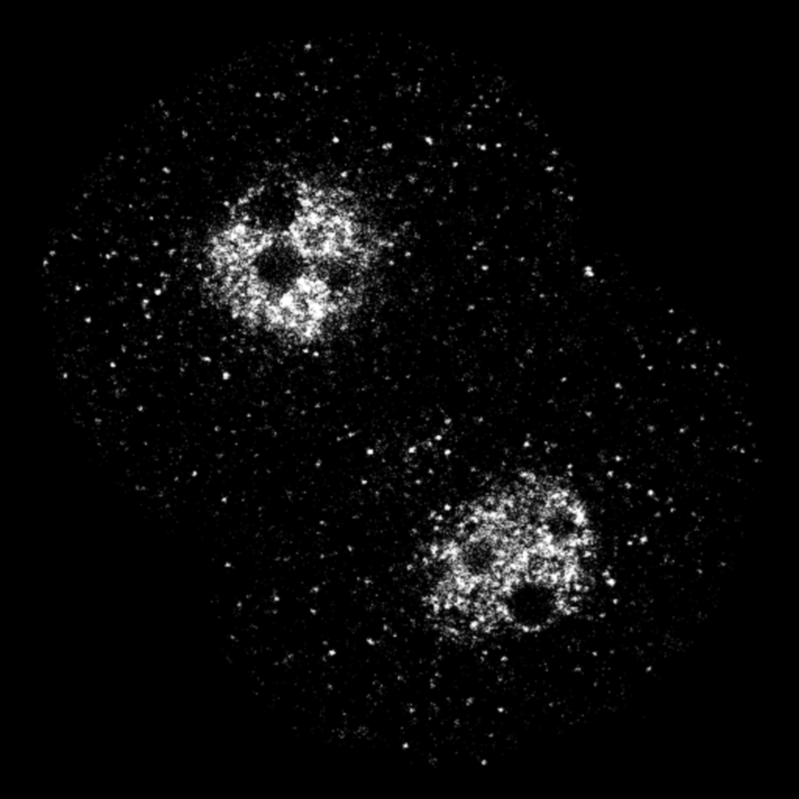

Supplement: Supplementary file 17 — Source Data for Figure 4 [file EMBJ-42-e113955-s009.zip › Figure_4/4E/Fig4E_vivo_5mC.tif]

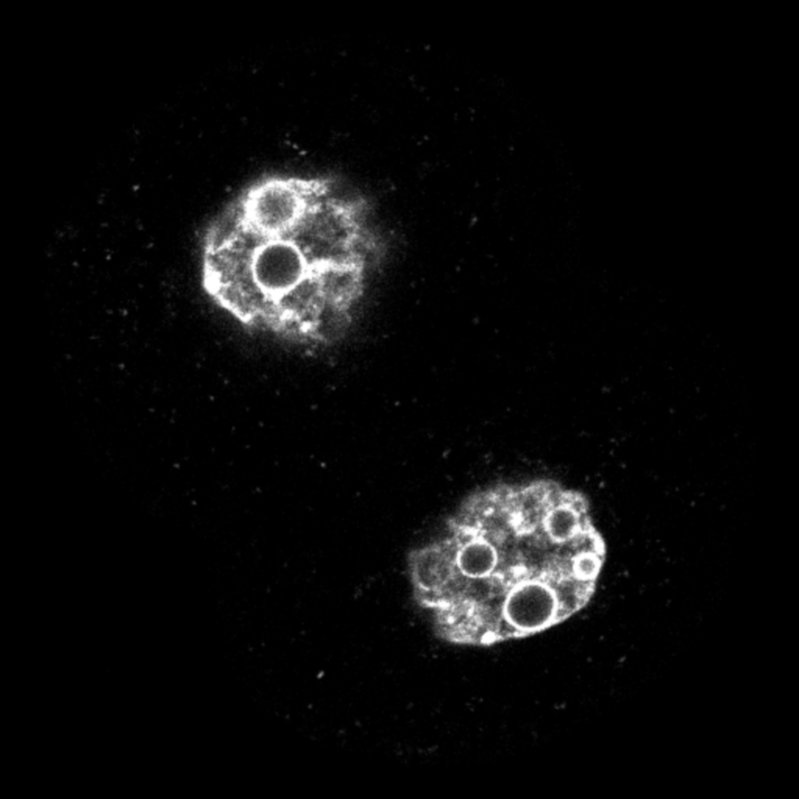

Supplement: Supplementary file 17 — Source Data for Figure 4 [file EMBJ-42-e113955-s009.zip › Figure_4/4E/Fig4E_vivo_DAPI.tif]

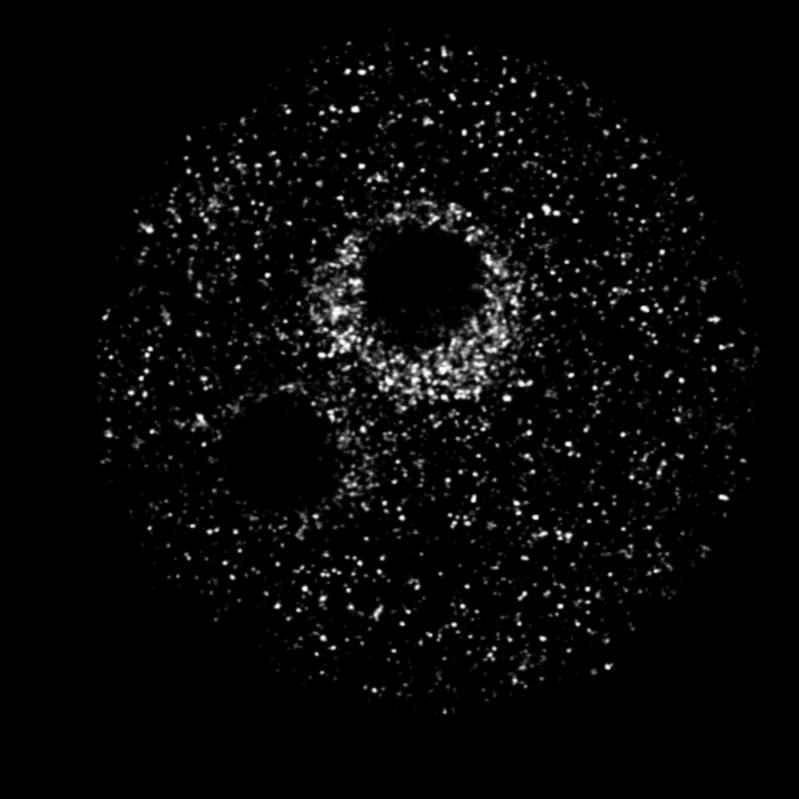

Supplement: Supplementary file 17 — Source Data for Figure 4 [file EMBJ-42-e113955-s009.zip › Figure_4/4E/Fig4E_Zygote_5hmC.tif]

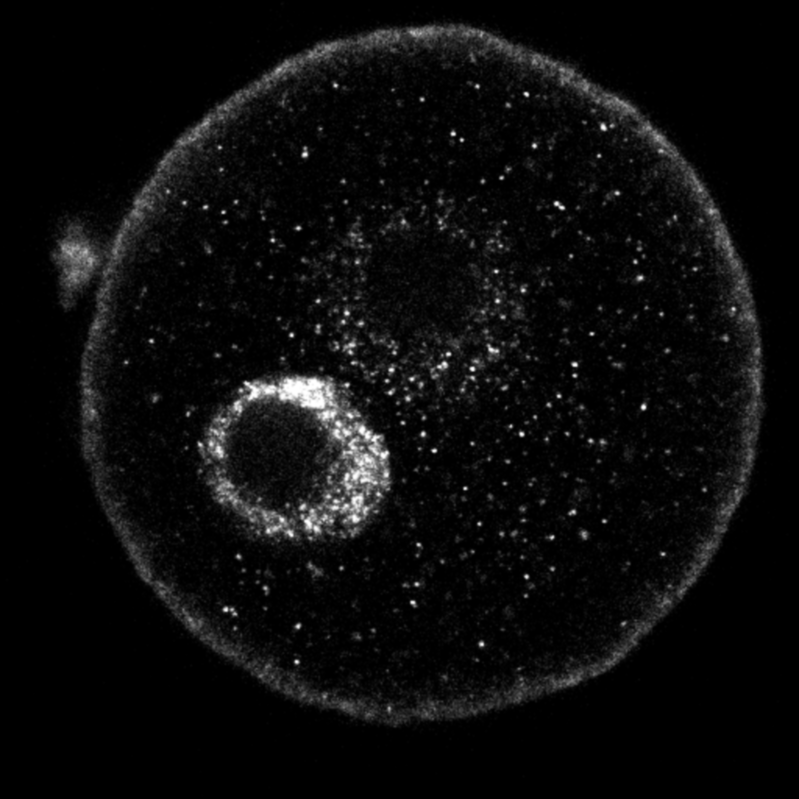

Supplement: Supplementary file 17 — Source Data for Figure 4 [file EMBJ-42-e113955-s009.zip › Figure_4/4E/Fig4E_Zygote_5mC.tif]

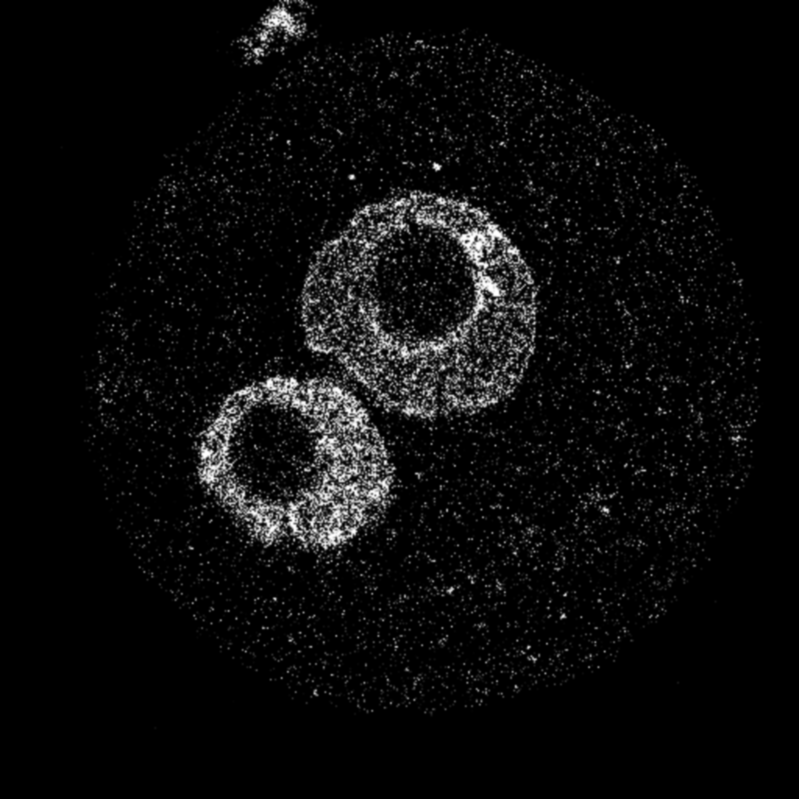

Supplement: Supplementary file 17 — Source Data for Figure 4 [file EMBJ-42-e113955-s009.zip › Figure_4/4E/Fig4E_Zygote_DAPI.tif]

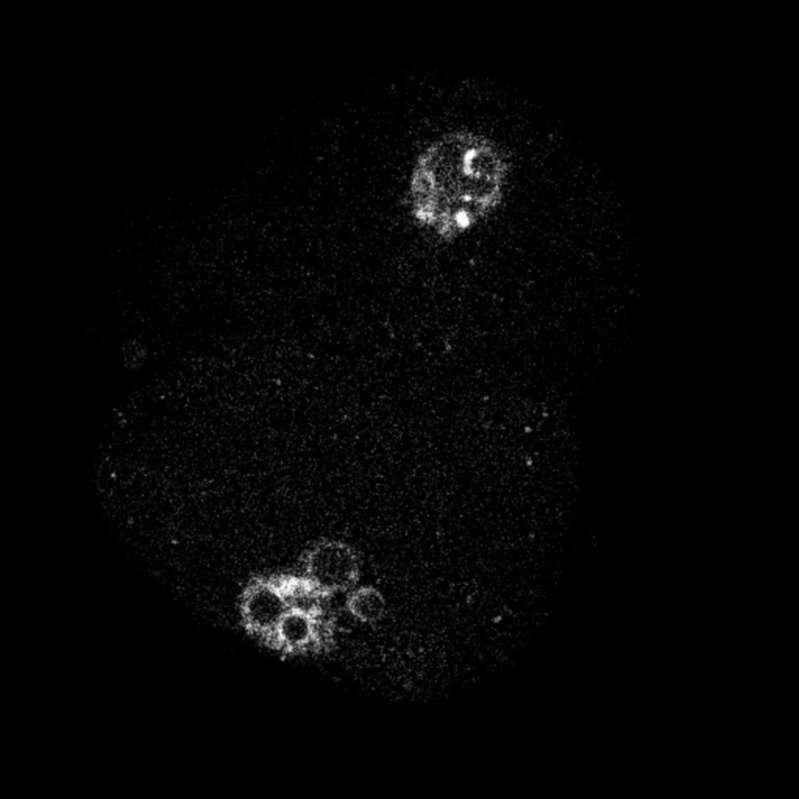

Supplement: Supplementary file 17 — Source Data for Figure 4 [file EMBJ-42-e113955-s009.zip › Figure_4/4G/Fig4G_iPSC-bottom_DAPI.tif]

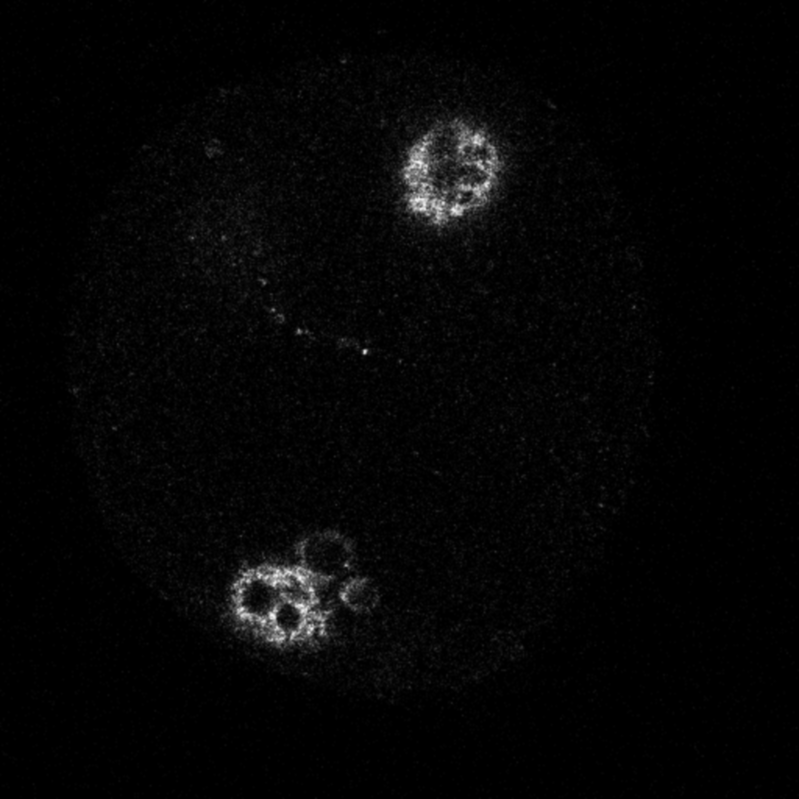

Supplement: Supplementary file 17 — Source Data for Figure 4 [file EMBJ-42-e113955-s009.zip › Figure_4/4G/Fig4G_iPSC-bottom_H3K9me2.tif]

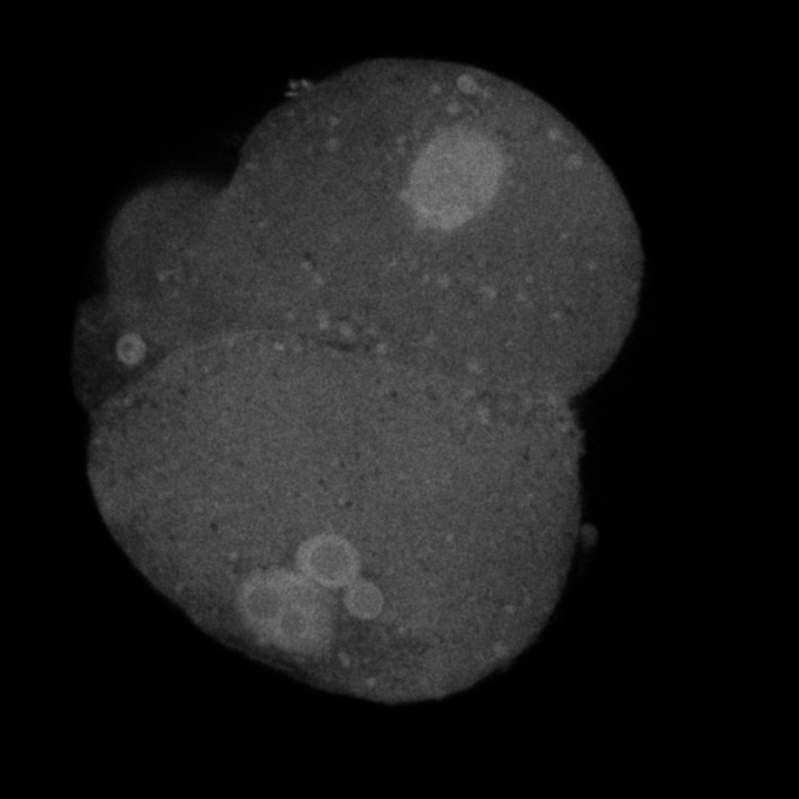

Supplement: Supplementary file 17 — Source Data for Figure 4 [file EMBJ-42-e113955-s009.zip › Figure_4/4G/Fig4G_iPSC-bottom_Stella-ECFP.tif]

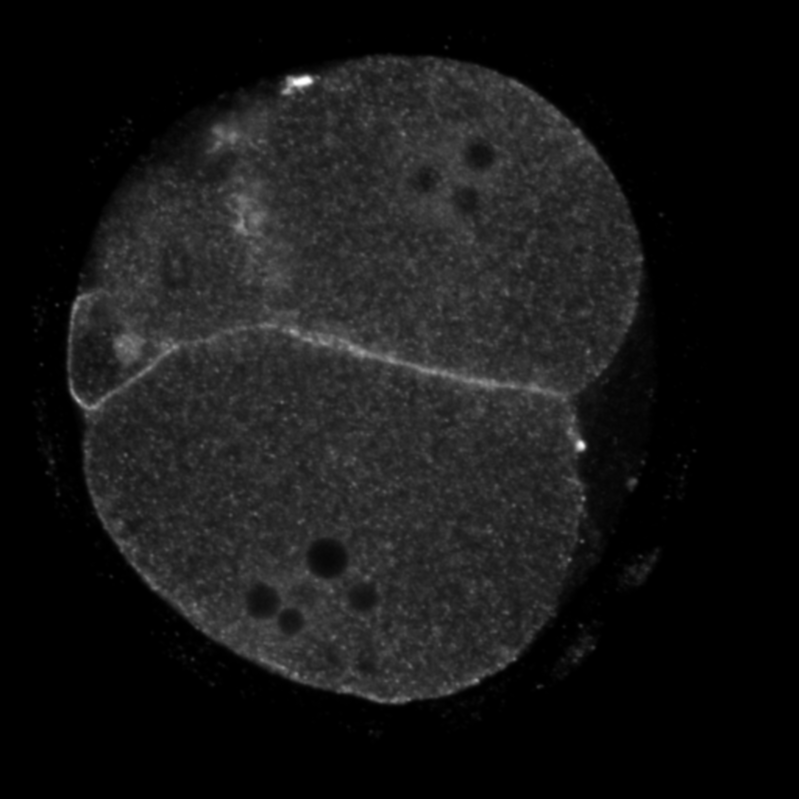

Supplement: Supplementary file 17 — Source Data for Figure 4 [file EMBJ-42-e113955-s009.zip › Figure_4/4G/Fig4G_iPSC-bottom_STELLA.tif]

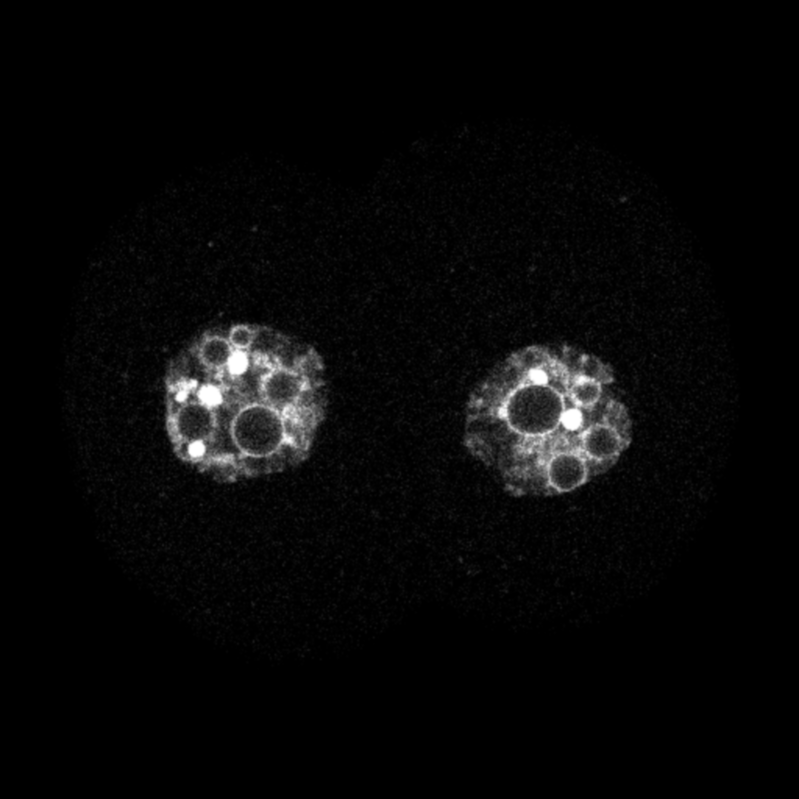

Supplement: Supplementary file 17 — Source Data for Figure 4 [file EMBJ-42-e113955-s009.zip › Figure_4/4G/Fig4G_iPSC-top_DAPI.tif]

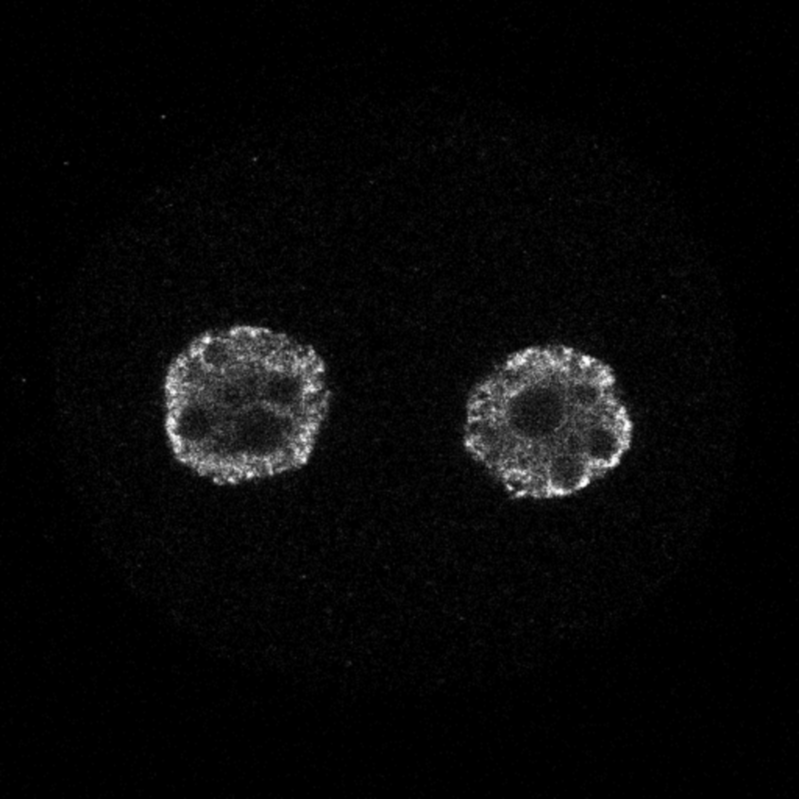

Supplement: Supplementary file 17 — Source Data for Figure 4 [file EMBJ-42-e113955-s009.zip › Figure_4/4G/Fig4G_iPSC-top_H3K9me2.tif]

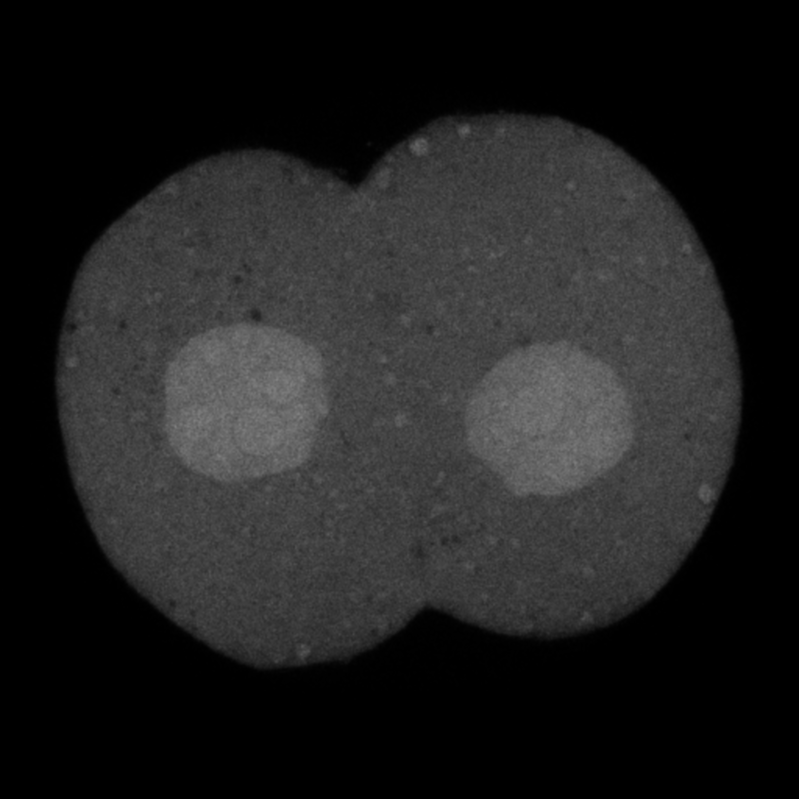

Supplement: Supplementary file 17 — Source Data for Figure 4 [file EMBJ-42-e113955-s009.zip › Figure_4/4G/Fig4G_iPSC-top_Stella-ECFP.tif]

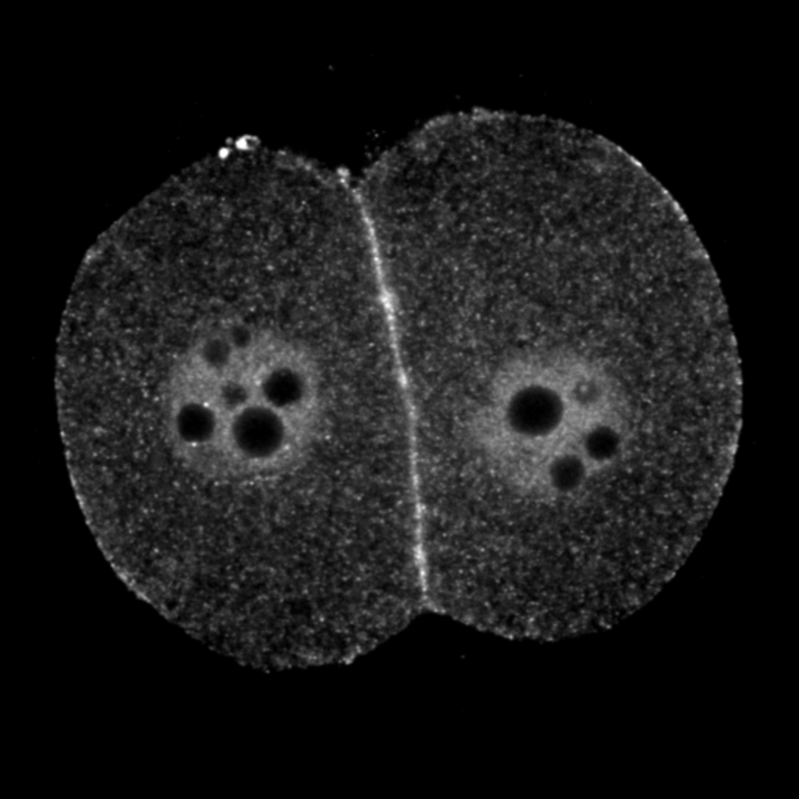

Supplement: Supplementary file 17 — Source Data for Figure 4 [file EMBJ-42-e113955-s009.zip › Figure_4/4G/Fig4G_iPSC-top_STELLA.tif]

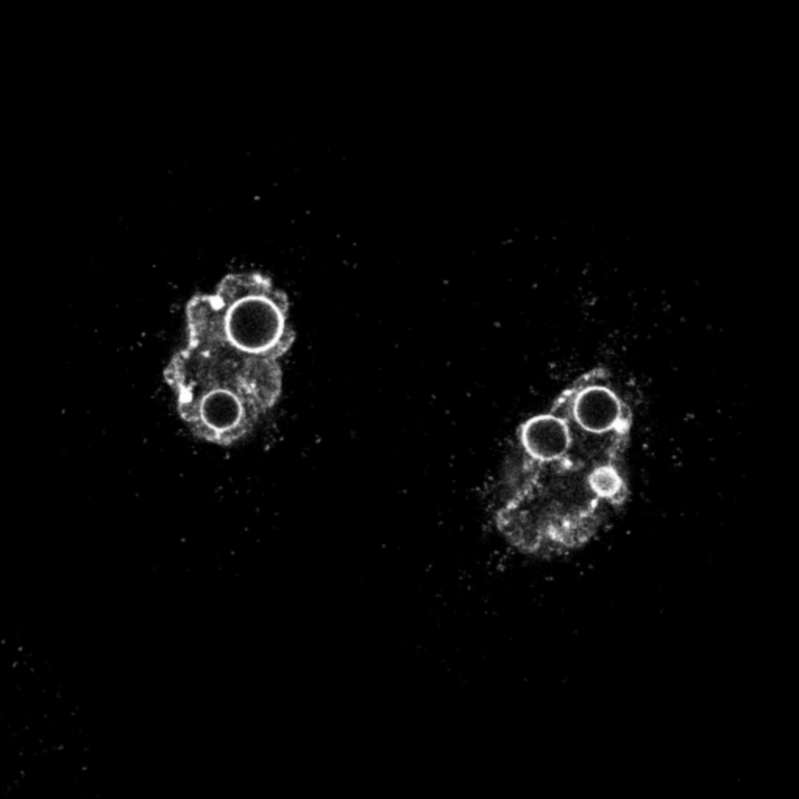

Supplement: Supplementary file 17 — Source Data for Figure 4 [file EMBJ-42-e113955-s009.zip › Figure_4/4G/Fig4G_vivo_DAPI.tif]

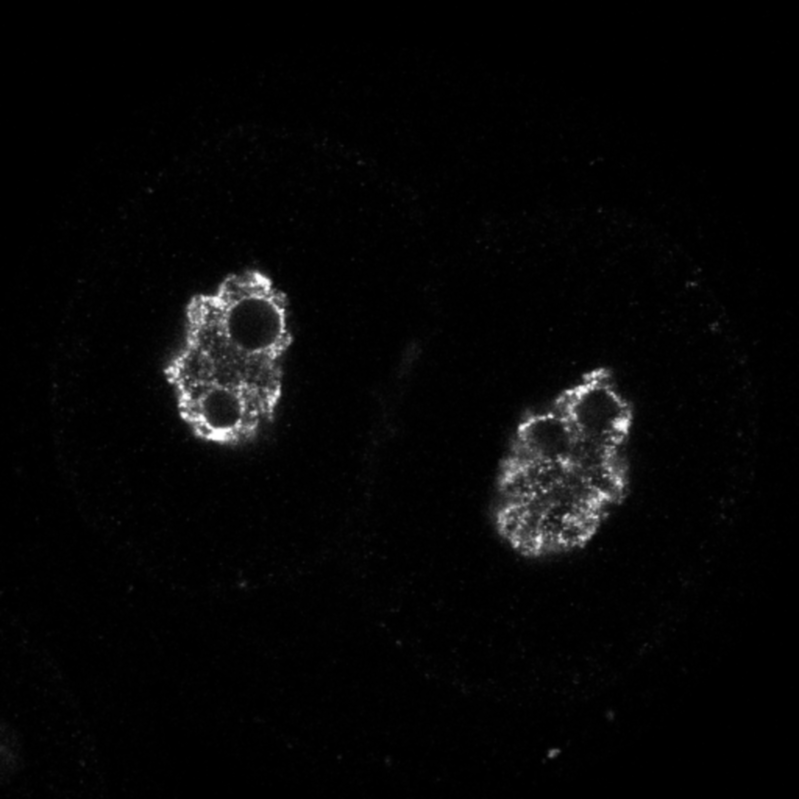

Supplement: Supplementary file 17 — Source Data for Figure 4 [file EMBJ-42-e113955-s009.zip › Figure_4/4G/Fig4G_vivo_H3K9me2.tif]

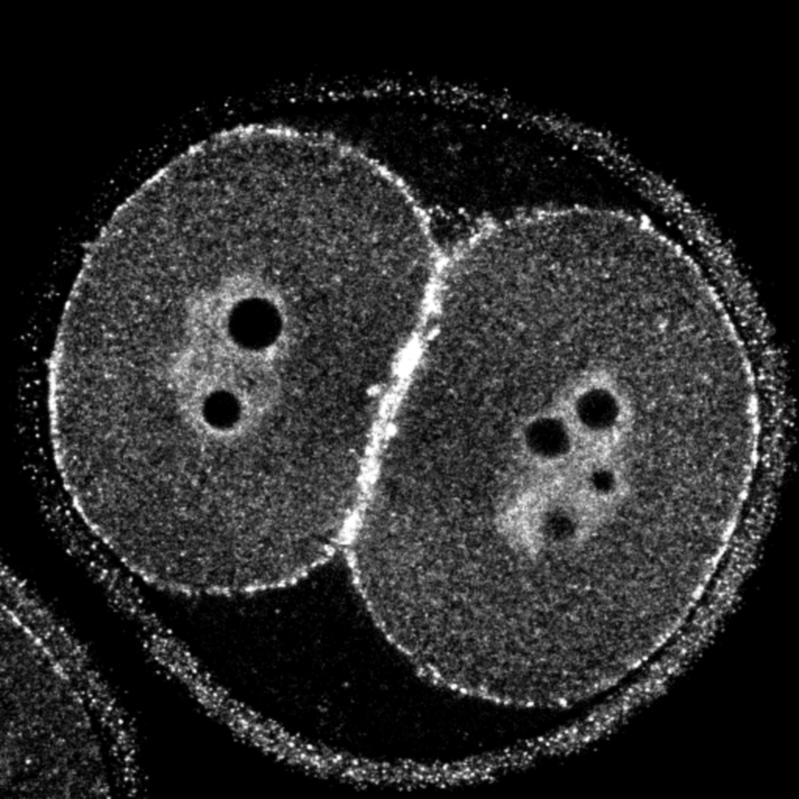

Supplement: Supplementary file 17 — Source Data for Figure 4 [file EMBJ-42-e113955-s009.zip › Figure_4/4G/Fig4G_vivo_STELLA.tif]
