# Supplementary material for: Epigenetic regulation limits competence of pluripotent stem cell‐derived oocytes
Source: EMBO J. 2023 Oct 18;42(23):e113955. doi: 10.15252/embj.2023113955 (PMC10690455; doi:10.15252/embj.2023113955)
Supplement: Supplementary file 20 [file EMBJ-42-e113955-s010.pdf]

THE EMBO JOURNAL

Epigenetic regulation limits competence of pluripotent stem cell-derived oocytes

Eishi Aizawa, Evgeniy Ozonov, Yumiko Kawamura, Charles-Etienne Dumeau, So Nagaoka, Tomoya Kitajima, Mitinori Saitou, Antoine Peters, and Anton Wutz
DOI: 10.15252/embj.2023113955

Corresponding authors: Anton Wutz (awutz@ethz.ch) , Antoine Peters (antoine.peters@fmi.ch)

Review Timeline:

Submission Date:	7th Mar 23
Editorial Decision:	3rd May 23
Revision Received:	3rd Aug 23
Editorial Decision:	10th Sep 23
Revision Received:	18th Sep 23
Accepted:	18th Sep 23

Editor: Daniel Klimmeck

Transaction Report:

(Note: With the exception of the correction of typographical or spelling errors that could be a source of ambiguity, letters and reports are not edited. Depending on transfer agreements, referee reports obtained elsewhere may or may not be included in this compilation. Referee reports are anonymous unless the Referee chooses to sign their reports.)

1st Editorial Decision **3rd May 2023**

Dear Anton, dear Antoine,

Thank you again for the submission of your manuscript (EMBOJ-2023-113955) to The EMBO Journal, as well as for your patience with our response at this time of the year which got protracted due to delayed referee input and discussions in the editorial team. Your study has been sent to two reviewers with expertise in developmental biology and epigenetics and we have received feedback from both of them, which I enclose below.

As you will see, the referees acknowledge the potential interest and novelty of your comparative analysis of in vitro oocyte specification protocols, although they also express several issues that will have to be conclusively addressed before they can be supportive of publication of your manuscript in The EMBO Journal. I judge the comments of the referees to be generally reasonable and given their overall interest, we are in principle happy to invite you to revise your manuscript experimentally to address the referees' comments.

As you may have seen on our web page, we generally allow three months as standard revision time. As a matter of policy, competing manuscripts published during this period will not negatively impact on our assessment of the conceptual advance presented by your study. However, we request that you contact the editor as soon as possible upon publication of any related work, to discuss how to proceed. Should you foresee a problem in meeting this three-month deadline, please let us know in advance and we may be able to grant an extension.

When submitting your revised manuscript, please carefully review the instructions below.

Please feel free to approach me any time should you have any questions related to this.

Thank you for the opportunity to consider your work for publication.

I look forward to your revision.

Best regards,

Daniel

Daniel Klimmeck, PhD
Senior Editor
The EMBO Journal

Instruction for the preparation of your revised manuscript:

1) a .docx formatted version of the manuscript text (including legends for main figures, EV figures and tables). Please make sure that the changes are highlighted to be clearly visible.

2) individual production quality figure files as .eps, .tif, .jpg (one file per figure).

3) a .docx formatted letter INCLUDING the reviewers' reports and your detailed point-by-point response to their comments. As part of the EMBO Press transparent editorial process, the point-by-point response is part of the Review Process File (RPF), which will be published alongside your paper.

4) a complete author checklist, which you can download from our author guidelines (https://wol-prod-cdn.literatumonline.com/pb-assets/embo-site/Author Checklist%20-%20EMBO%20J-1561436015657.xlsx). Please insert information in the checklist that is also reflected in the manuscript. The completed author checklist will also be part of the RPF.

5) Please note that all corresponding authors are required to supply an ORCID ID for their name upon submission of a revised manuscript.

6) It is mandatory to include a 'Data Availability' section after the Materials and Methods. Before submitting your revision, primary datasets produced in this study need to be deposited in an appropriate public database, and the accession numbers and database listed under 'Data Availability'. Please remember to provide a reviewer password if the datasets are not yet public (see https://www.embopress.org/page/journal/14602075/authorguide#datadeposition).
In case you have no data that requires deposition in a public database, please state so in this section. Note that the Data

Availability Section is restricted to new primary data that are part of this study.
*** Note - All links should resolve to a page where the data can be accessed. ***

7) Our journal encourages inclusion of *data citations in the reference list* to directly cite datasets that were re-used and obtained from public databases. Data citations in the article text are distinct from normal bibliographical citations and should directly link to the database records from which the data can be accessed. In the main text, data citations are formatted as follows: "Data ref: Smith et al, 2001" or "Data ref: NCBI Sequence Read Archive PRJNA342805, 2017". In the Reference list, data citations must be labeled with "[DATASET]". A data reference must provide the database name, accession number/identifiers and a resolvable link to the landing page from which the data can be accessed at the end of the reference. Further instructions are available at .

8) At EMBO Press we ask authors to provide source data for the main and EV figures. Our source data coordinator will contact you to discuss which figure panels we would need source data for and will also provide you with helpful tips on how to upload and organize the files.

Numerical data can be provided as individual .xls or .csv files (including a tab describing the data). For 'blots' or microscopy, uncropped images should be submitted (using a zip archive or a single pdf per main figure if multiple images need to be supplied for one panel). Additional information on source data and instruction on how to label the files are available at .

9) We replaced Supplementary Information with Expanded View (EV) Figures and Tables that are collapsible/expandable online (see examples in https://www.embopress.org/doi/10.15252/embj.201695874). A maximum of 5 EV Figures can be typeset. EV Figures should be cited as 'Figure EV1, Figure EV2" etc. in the text and their respective legends should be included in the main text after the legends of regular figures.

- For the figures that you do NOT wish to display as Expanded View figures, they should be bundled together with their legends in a single PDF file called *Appendix*, which should start with a short Table of Content. Appendix figures should be referred to in the main text as: "Appendix Figure S1, Appendix Figure S2" etc. See detailed instructions regarding expanded view here: .

- Additional Tables/Datasets should be labelled and referred to as Table EV1, Dataset EV1, etc. Legends have to be provided in a separate tab in case of .xls files. Alternatively, the legend can be supplied as a separate text file (README) and zipped together with the Table/Dataset file.

10) When assembling figures, please refer to our figure preparation guideline in order to ensure proper formatting and readability in print as well as on screen:
http://bit.ly/EMBOPressFigurePreparationGuideline

Please remember: Digital image enhancement is acceptable practice, as long as it accurately represents the original data and conforms to community standards. If a figure has been subjected to significant electronic manipulation, this must be noted in the figure legend or in the 'Materials and Methods' section. The editors reserve the right to request original versions of figures and the original images that were used to assemble the figure.

11) For data quantification: please specify the name of the statistical test used to generate error bars and P values, the number (n) of independent experiments (specify technical or biological replicates) underlying each data point and the test used to calculate p-values in each figure legend. The figure legends should contain a basic description of n, P and the test applied. Graphs must include a description of the bars and the error bars (s.d., s.e.m.).

Please remember: Digital image enhancement is acceptable practice, as long as it accurately represents the original data and conforms to community standards. If a figure has been subjected to significant electronic manipulation, this must be noted in the figure legend or in the 'Materials and Methods' section. The editors reserve the right to request original versions of figures and the original images that were used to assemble the figure.

Further information is available in our Guide For Authors: https://www.embopress.org/page/journal/14602075/authorguide

We realize that it is difficult to revise to a specific deadline. In the interest of protecting the conceptual advance provided by the work, we recommend a revision within 3 months (1st Aug 2023). Please discuss the revision progress ahead of this time with the editor if you require more time to complete the revisions.

Referee #1:

The manuscript from Aizawa et al. investigates the causes underlying the low developmental potential of mouse oocytes derived in vitro from pluripotent stem cells. In vitro models hold great potential to investigate mammalian female germline development and early embryogenesis, however their use as research tools has been thus far quite limited. This study provides a valuable comparison across various stages of in vitro culture and tries to disentangle the effect of each step of the culture process on mature oocyte quality. Overall, the study is well performed and carefully analysed, with balanced conclusions. We consider it an important contribution that will definitely be of interest to the fields of mammalian oogenesis and epigenetics. And more broadly, to all those wishing to use in vitro oocyte models for their research. We recommend it for publication in EMBO Journal and also suggest some points that could substantiate the findings and improve precision of the manuscript.

Aizawa et al. carried out a careful comparison between in vivo oocytes and those derived i) from induced Pluripotent Stem Cells (iPSCs), ii) from E12.5 gonads and iii) from 6dpp follicles. This enabled them to disentangle the relative effects of in vitro PGC differentiation (IVP), in vitro oocyte differentiation (IVD), in vitro follicle growth (IVG) and in vitro maturation (IVM) on oocyte quality, both from a developmental point of view and at the molecular level. First of all, they were able to generate morphologically mature oocytes from PSCs, thus successfully replicating the results from other laboratories (ie, Hikabe et al., 2016). Being these highly challenging protocols, this is in itself a valuable result from this study, which may encourage broader use of these in vitro culture models. Secondly, and most importantly, their comparison between different conditions and culture steps is key to evaluate the potential of this technology for laboratory research and for the clinic and is not currently available in published literature. From comparing the relative efficiencies of oocyte maturation and fertilisation, the authors find that IVD is the most critical step of the entire differentiation protocol, affecting developmental competence of oocytes derived from iPSCs. At the molecular level, they find that genes normally up-regulated during oocyte development are more susceptible to de-regulation especially during IVD. Most of these genes appear to be Polycomb targets, thus identifying a potentially fragile epigenetic mechanism that contributes to the poor developmental potential of in vitro-derived oocytes. Finally, by analysing zygotes/2-cell embryos derived from in vivo versus in vitro oocytes, they find compromised transcriptional activation of in vitro-derived embryos and aberrant 5hmC on maternal chromosomes, suggesting a ZGA failure as a potential underlying cause of failed development of in vitro-derived oocytes.

The comments below represent suggestions and/or queries that may be considered but are generally at the discretion of the authors.

Major points:
1. Figure 3: Were oocytes that do not progress to 2-cell stage arrested at the PN stage or not fertilised at all? Given the low rate of progression to 2-cell stage for E12.5 gonadal- and BVSC-iPCS-derived oocytes, it is important to understand at what point this failure occurs.
2. Line 208: please provide a quantification of what fraction of oocytes extruded the first polar body
3. Line 209 and Figure 3B: since this manuscript carefully replicates challenging culture systems and compares results across conditions, it would be valuable if authors could add efficiency numbers from published studies side-by-side to data from their experiments, if published studies provide such information.
4. Figure EV4E shows slightly reduced H3K27me3 levels in iPSC-derived 2-cell embryos compared to in vivo-derived, whilst the text and quantification in EV4F argue for the opposite. Likewise, the slight increase in H3K4ac from Figure EV4D does not seem apparent in the corresponding image in Figure EV4C. The images should be replaced with others more representative of the quantification.
5. Figure EV4C shows different nuclear morphology between in vivo and iPSC-derived 2 cell embryos, which might stem from slightly offset developmental timing and possibly affect levels of chromatin modifications. The images should be replaced with others at a more similar stage
6. What are the levels of 5mC in in vitro vs in vivo derived oocytes? Are they higher in in vitro oocytes, thus possibly explaining the aberrant high 5hmC levels in the embryo from Figure 4E? Please provide immunofluorescence data.
7. The schematic in Figure 5A provides an overview of which samples are analysed and the relative comparisons, which is very helpful in guiding the reader through what each of them indicates. However, the following panels and also Figure 6 mostly indicate samples as "A2" "B2" etc, which is often hard to link back to figure 5A. We would therefore suggest improving the labels by either replacing letters with more meaningful names or by adding information about the comparison.
8. Figure 6F shows expression levels of components of PRC1 and PRC2. It would be useful to add expression levels of the subunits of other epigenetic regulator complexes (ie, DNA methylation, etc) to get a more comprehensive picture of epigenetic regulators during in vitro oogenesis.
9. Clusters UP2 and UP3 show premature activation during IVD and appear to be enriched for PRC1- and PRC2-mediated histone modifications. Is there any (epi)genomic feature of these genes that could potentially explain their susceptibility to in vitro culture? Ie, do they locate preferentially at the boundaries of large Polycomb domains established during oogenesis (as the two screenshots in Figure EV6I-J would suggest)?
10. Provide GO analysis of UP1 and UP2 clusters
11. In the published version of the manuscript, authors should include count tables for all relevant gene sets identified as differentially expressed with relative fold-changes and statistical significance for all comparisons

Minor points:
12. Related to #09: ChIP-seq or CUT&RUN-seq for H3K27me3 and H2AK119ub on in vitro derived oocytes would greatly substantiate the findings on aberrant Polycomb regulation as it would address the question of whether the oocyte epigenome is correctly established during in vitro oogenesis. However, we understand the challenges and limitations of low-input chromatin profiling combined with this culture protocol and do not deem this experiment essential for publication.
13. Line 196: it is not clear if they cultured one cluster of 1-3 or 4-10 oocytes in each well or multiple clusters together. Also, how does this differ from standard culture conditions?
14. Lines 706-712: this step of the protocol is not entirely clear, please rephrase.
15. Figure 3: the label "activation" should be replaced with "parthenogenetic activation" to facilitate readability
16. H3K4me3 is often referred to as an "activating mark", however studies in oocytes suggest that this mark might have an unusually repressive function in this cell type (ie, Zhang et al., 2016), so this should be rephrased.

****
Jamie Hackett & Marzia Munafo;. Please do not remove this note.
My standard policy is to sign ALL peer review reports, irrespective of my comments or recommendations. Further communication related to this should go via the editor.
****

Referee #2:

The authors provide an in-depth analysis of the challenges in studying the female germline in mammals and the development of in vitro culture systems to overcome experimental limitations. The study highlights the successful generation of germ cells and mature gametes, including primordial germ cells and mature metaphase II oocytes, from pluripotent stem cells (PSCs) using a cocktail of growth factors. However, the study also identifies the variable potential for embryogenesis of oocytes developed in vitro, with a substantially lower success rate of full-term development compared to embryos generated using oocytes from superovulated mice.

The authors also explore epigenetic regulations critical for successful preimplantation development and the potential causes that impair the integrity of oocytes during culture. The results of a detailed comparison between oocyte development in vitro and in vivo identify critical culture steps and specific molecular factors, such as the impaired regulation of genes normally repressed by Polycomb group proteins, that are misregulated in in vitro generated oocytes. The manuscropt provides insights into the importance of epigenetic regulation at an early stage of oocyte differentiation for successful preimplantation development and identifies potential improvements for in vitro culture systems.

Overall, this research is a comprehensive analysis of the challenges and opportunities in studying the female germline in mammals and the development of in vitro culture systems. The study provides insights into the molecular mechanisms and limitations of in vitro oocyte development and identifies critical steps for future improvement. The study has significant implications for reproductive biology and stem cell research, and its findings could lead to new avenues in assisted reproduction and conservation of endangered species.

Major comments:
1. The author titled the study as "Epigenetic regulation limits competence of ...", but only performed transcriptome sequencing. It would be more novel to include the H3K4ac, H3K4me3 profile of the oocytes from the in vitro differentiation system.
2. The author raised concerns about the in vitro culture system but did not propose any improvement methods. These methods have also been described in previous publications (Hirabe et al, Nature, 2016; Yoshino et al, Science, 2021).
3. During oocyte maturation, the author only focused on the transcriptional levels, while we know that transcription is suppressed during oocyte maturation, and the true regulation of its maturation occurs at the translational level. The author did not study the differences in oocyte differentiation from the perspectives of proteomics or translatomics.
4. It would be important to include a comparison of the expression of transcription factors between in vivo and in vitro model systems.

Minor comments
5. In line 388, the authors refer to the gene in vitro expressions referencing Figs 5C and D. However, these figures appear to only include in vivo data
6. In line 389-393, author should clearly distinguish between 'all genes' and 'affected genes'. Are all genes shown in the in vivo data? I have trouble understanding the difference between the following sets - IVP@FGO+IVD@FGO+IVG@FGO

Response to reviewers' comments

Referee #1:

Major points:

1. Figure 3: Were oocytes that do not progress to 2-cell stage arrested at the PN stage or not fertilised at all? Given the low rate of progression to 2-cell stage for E12.5 gonadal- and BVSC-iPCS-derived oocytes, it is important to understand at what point this failure occurs.

Response: We thank the reviewer for raising this important question. In response we counted the number of pronuclei in oocytes after IVF. Two pronuclei were observed in 70.3% (52 / 74) of iPSC-derived oocytes and in 73.2% (52 / 71) of *in vivo*-derived oocytes. Considering the similarity of these ratios, we think *in vitro*-derived oocytes possess similar competence of fertilization as *in vivo*-derived oocytes, but the developmental arrest occurred mainly at the 2-cell stage. We revised the manuscript by adding these points in lines 229-234.

2. Line 208: please provide a quantification of what fraction of oocytes extruded the first polar body

Response: The fractions of oocytes with first polar bodies were 22.1% (52 / 235) for BVSC-ESC-derived oocytes and 12.7% (722 / 5,701) for BVSC-iPSC-derived oocytes. We described this quantification in lines 200-202. Also, the number of BVSC-ESC-derived oocytes has been added in Table 1.

3. Line 209 and Figure 3B: since this manuscript carefully replicates challenging culture systems and compares results across conditions, it would be valuable if authors could add efficiency numbers from published studies side-by-side to data from their experiments, if published studies provide such information.

Response: We thank the reviewer for the comment. As far as we checked, the report by Hikabe (Hikabe et al., 2016) is the only published study which took a similar protocol as our study and reported the developmental efficiency. Therefore, we showed the data from Hikabe et al in Figure 3B to compare with our data. Also, the number of oocytes/embryos and their developmental efficiency reported in Hikabe et al has been added to Table 1 for comparison. In addition, the data from Yoshino et al., 2021 has been added to Table 1 for reference. Although the protocol of Yoshino et al to generate oocytes using FOSLCs is different from ours and Hikabe et al, we think the data of Yoshino et al is also useful to discern the efficiency of oocyte/embryo development.

4. Figure EV4E shows slightly reduced H3K27me3 levels in iPSC-derived 2-cell embryos compared to in vivo-derived, whilst the text and quantification in EV4F argue for the opposite. Likewise, the slight increase in H3K4ac from Figure EV4D does not seem apparent in the corresponding image in Figure EV4C. The images should be replaced with others more representative of the quantification.

Response: We thank the reviewer for the suggestion. We replaced images in new Fig EV3A, which representatively show a higher signal of H3K4ac in the iPSC-derived 2-cell embryo. Likewise, images in new Fig EV3C were replaced to demonstrate a higher signal of H3K27me3, which corresponds to the quantification in new Fig EV3D.

5. Figure EV4C shows different nuclear morphology between in vivo and iPSC-derived 2 cell embryos,

which might stem from slightly offset developmental timing and possibly affect levels of chromatin modifications. The images should be replaced with others at a more similar stage
Response: We agree with the reviewer's point that the different developmental timing possibly affects levels of chromatin modifications. In response, we replaced representative images in new Fig EV3A to demonstrate signals in similar morphological nuclei of *in vivo*-derived and iPSC-derived 2-cell embryos.

6. What are the levels of 5mC in in vitro vs in vivo derived oocytes? Are they higher in in vitro oocytes, thus possibly explaining the aberrant high 5hmC levels in the embryo from Figure 4E? Please provide immunofluorescence data.
Response: We thank the reviewer for raising this important question. In response, we performed immunostaining analysis of 5mC and 5hmC on a new set of *in vitro*-generated and *in vivo*-derived oocytes after IVD and IVG. We used oocytes from E12.5 gonads to generate *in vitro*-derived samples allowing the generation of sufficient numbers of oocytes for the experiments required in the revision. The results of analysis are now shown in new Fig EV3E and EV3F. Most E12.5 gonad-derived oocytes after IVD and IVG exhibited non-surrounded nucleolus (NSN) and surrounded nucleolus (SN) states, respectively, in their nuclei. Therefore, we selected only NSN oocytes after IVD and SN oocytes after IVG for analysis. We observed comparable levels of 5mC signals between *in vitro*-derived and *in vivo*-derived oocytes. In contrast, the 5hmC signal was undetectable in *in vitro*-derived oocytes as well as *in vivo*-derived oocytes. These data indicate that ectopic 5hmC in *in vitro*-derived 2-cell parthenotes presumably emerged not during oocyte development but after parthenogenetic activation, possibly because of the mis-protection from TET3 activity. Our data on mislocalization of STELLA supports this idea. We additionally described these points in lines 321-331.

7. The schematic in Figure 5A provides an overview of which samples are analysed and the relative comparisons, which is very helpful in guiding the reader through what each of them indicates. However, the following panels and also Figure 6 mostly indicate samples as "A2" "B2" etc, which is often hard to link back to figure 5A. We would therefore suggest improving the labels by either replacing letters with more meaningful names or by adding information about the comparison.
Response: We thank the reviewer for this suggestion and agree that chosen labeling was not very intuitive. Therefore, we have updated the labels for the different oocyte cohorts as follows: GRO^{iPSC} , FGO^{iPSC}(A2, A3); GRO^{PGC}, FGO^{PGC} (B2, B3); $FGO^{6dpp\ Oo}$ (C3); $GRO^{in\ vivo}$, $FGO^{in\ vivo}$ (D2, D3). Figures (Fig 5A, 5B, 6B, 6E, EV5A, EV5I, EV5J) and tables (Datasets EV3, EV4) were updated accordingly.

8. Figure 6F shows expression levels of components of PRC1 and PRC2. It would be useful to add expression levels of the subunits of other epigenetic regulator complexes (ie, DNA methylation, etc) to get a more comprehensive picture of epigenetic regulators during in vitro oogenesis.
Response: We agree with the reviewer that it would be useful for a reader to get a broader overview of impact of *in vitro* oogenesis on genes involved in other epigenetic pathways. Therefore, we expanded our list of genes for other epigenetic complexes and transcription factors and added an Appendix Figure S1 with transcriptional responses and a simplified version of expression changes where we merge expression values for biological replicates (PGC and PGCLC) or oocytes belonging to the same cohorts. In addition, we have updated Figure 6F by selecting epigenetic regulators and transcription factors which have statistically significant expression response to *in vitro* oogenesis or expression difference between PGCLC and PGC.

9. Clusters UP2 and UP3 show premature activation during IVD and appear to be enriched for PRC1- and PRC2-mediated histone modifications. Is there any (epi)genomic feature of these genes that could potentially explain their susceptibility to in vitro culture? Ie, do they locate preferentially at the boundaries of large Polycomb domains established during oogenesis (as the two screenshots in Figure EV6I-J would suggest)?

Response: We thank the reviewer for raising this interesting point. To address this question, we carried out analysis of H3K27me3 domains with respect to the relative and absolute positions of affected genes to borders of H3K27me3 domains. In particular, we performed calling of broad H3K27me3 peak domains separately for non-growing oocytes and FGO (data from Hanna, Taudt, et al, 2018) using epic2 tool (Stovner & Saetrom, 2019; DOI: 10.1093/bioinformatics/btz232) (see two left columns in Fig 1A below). Next, we plotted H3K27me3 enrichment profiles within and around such domains (10kb upstream and downstream of left and right domain borders respectively), merged for two stages (and normalized to domain length) and illustrated the positions of transcription start sites of affected genes with respect to domain borders (see columns 3-5 in Fig 1A). Finally, we analyzed the relative positions of TSS overlapping H3K27me3 domains by comparing them to all genes belonging to clusters with characteristic behavior in WT oocytes (LS, UP, DN clusters from Fig 5D in the manuscript) (see column 6 in Fig 1A) and illustrated this as empirical cumulative distributions in Fig 1B below.

In addition, we compared genomic distances of genes belonging to clusters in Fig 6B of the manuscript to the nearest H3K27me3 domain border to all genes with the same WT expression behavior (see Fig 1C below). We noticed neither strong patterns in the locations of these genes towards edges of H3K27me3 domains (fig 1B) nor strong biases in distances to nearest domain edges. Perhaps, to address this interesting point thoroughly one would need higher temporal resolution of H3K27me3 dynamics throughout the course of oogenesis.

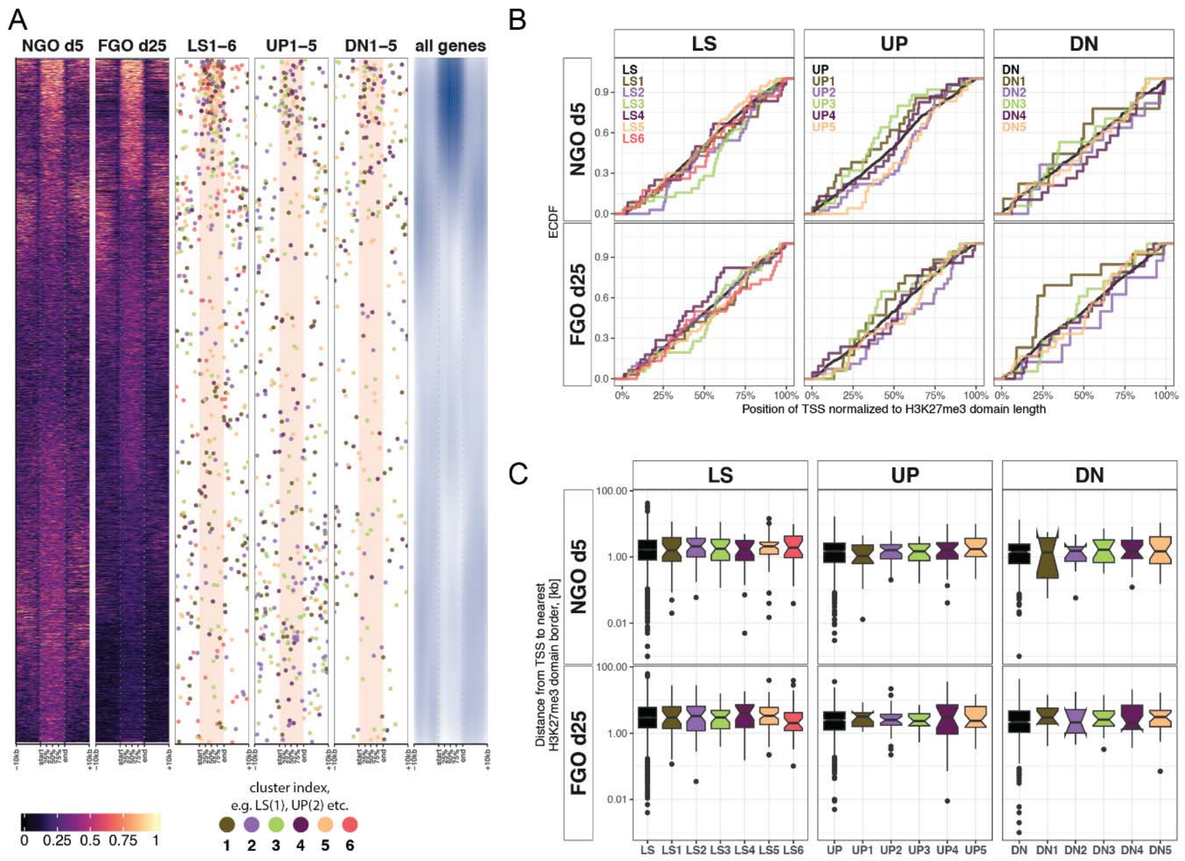

Figure 1. (A) H3K27me3 enrichment profiles within and around (+/- 10kb) H3K27me3 domains in non-growing oocytes and FGO (data from (Hanna, Taudt et al., 2018)) (columns 1-2) and positions of transcription start sites (TSS) of genes affected by in vitro development belonging to clusters in Fig 6B in the manuscript (heatmaps LS1-6, UP1-5 and DN1-5) (columns 3-5). Heatmap "all genes" represents densities of all TSSs within and around H3K27me3 domains (column 6). Positions within H3K27me3 domains were scaled by domain widths and represent relative positions to edges of domains. Positions outside of H3K27me3 domains represent absolute genomic distances (10kb upstream and downstream of domains starts and ends).

(B) Cumulative distributions of relative positions within H3K27me3 domains in non-growing oocytes and FGO of TSSs of genes with specific expression dynamics in vivo (LS, UP and DN, see Fig 5C) and affected genes belonging to clusters from Fig 6B in the manuscript.

(C) Boxplots of absolute genomic distances from TSSs of genes specific expression dynamics in vivo (LS, UP and DN, see Fig 5C in the manuscript) and affected genes belonging to clusters from Fig 6B in the manuscript to nearest edge of H3K27me3 domains in non-growing oocytes and FGO.

10. Provide GO analysis of UP1 and UP2 clusters

Response: We carried out GO enrichment analysis of genes belonging to each cluster in the Fig 6B and included the results as Dataset EV7.

11. In the published version of the manuscript, authors should include count tables for all relevant gene

sets identified as differentially expressed with relative fold-changes and statistical significance for all comparisons

Response: We included Dataset EV4 which contains comprehensive gene annotation (including relationship to Fig 5C and 6B), expression responses with statistical significance to *in vitro* oogenesis as well as aggregated expression values for each oocyte cohort.

Minor points:

12. Related to #09: ChIP-seq or CUT&RUN-seq for H3K27me3 and H2AK119ub on in vitro derived oocytes would greatly substantiate the findings on aberrant Polycomb regulation as it would address the question of whether the oocyte epigenome is correctly established during in vitro oogenesis. However, we understand the challenges and limitations of low-input chromatin profiling combined with this culture protocol and do not deem this experiment essential for publication.

Response: We appreciate the reviewer's thoughtful and constructive suggestion. We agree with the reviewer's suggestion that ChIP-seq or CUT&RUN-seq for histone marks would substantiate our findings on aberrant Polycomb regulation. As the reviewer pointed out, the limited number of samples would be a concern. Moreover, the partial heterogeneity in transcriptional responses observed between oocytes undergoing a similar *in vitro* growth regime indicates the principle need for a single cell assay. Unfortunately, we have not developed such a low-input chromatin analysis assay. Therefore, we focused our present study on phenotypic and expression analysis.

13. Line 196: it is not clear if they cultured one cluster of 1-3 or 4-10 oocytes in each well or multiple clusters together. Also, how does this differ from standard culture conditions?

Response: Multiple clusters of 1-3 and 4-10 follicles were cultured and evaluated together in the same well. This explanation was added in lines 723-725. The original culture condition reported by Hikabe et al. used individually separated follicles for IVG. Also, they reported the culture of the whole rOvary, resulting in a limited development of follicles, particularly only at the edge of the rOvary (Extended Fig 3a in Hikabe et al, 2016). Our data indicates that an intermediate condition (clusters of 4-10 follicles) between conditions of individual follicles and whole rOvaries yielded the best follicle development during IVG.

14. Lines 706-712: this step of the protocol is not entirely clear, please rephrase.

Response: We rephrased the steps of the protocol to improve the description in lines 729-741.

15. Figure 3: the label "activation" should be replaced with "parthenogenetic activation" to facilitate readability

Response: The label "activation" was replaced with parthenogenetic activation (PA) in Fig 3. Likewise, "activation" was also replaced in Fig 2A and Table 1.

16. H3K4me3 is often referred to as an "activating mark", however studies in oocytes suggest that this mark might have an unusually repressive function in this cell type (ie, Zhang et al., 2016), so this should be rephrased.

Response: we thank the reviewer for raising this point. We indeed agree with the reviewer that mature GV oocytes harbor rather broad domains of H3K4me3 beyond the typical enrichments observed around promoters in other cell types. In our current study, we only assess the impact of promoter associated enrichments on gene expression, and do not consider the possible functions of H3K4me3 throughout the

remaining of the genome. Nonetheless, to avoid vagueness, we rephrased the main text as follows: from "activating marks" into "marks associated with transcriptional activity".

Referee #2:

Major comments:

1. The author titled the study as "Epigenetic regulation limits competence of ...", but only performed transcriptome sequencing. It would be more novel to include the H3K4ac, H3K4me3 profile of the oocytes from the in vitro differentiation system.

Response: We thank the reviewer for the positive assessment of our manuscript and for providing constructive feedback for further improving our manuscript. We agree with the reviewer that chromatin analysis of oocytes under the *in vitro* system would provide novel and interesting insights. Considering the limited number of oocytes available, however, we have not set up low-input chromatin analysis. We therefore would leave this analysis for a comprehensive future investigation and focus our present study on a comparison of *in vitro* and *in vivo* oocyte development by phenotypic and expression analysis, which identifies critical culture steps and modes of gene regulation.

2. The author raised concerns about the in vitro culture system but did not propose any improvement methods. These methods have also been described in previous publications (Hirabe et al, Nature, 2016; Yoshino et al, Science, 2021).

Response: We thank the reviewer for the thoughtful comment. Our study indicated that IVD is the major cause of epigenetic instability in oocytes and low competence for preimplantation development. Considering that IVD is initiated by co-culture of E12.5 gonadal somatic cells and d6 PGCLCs (comparable to migrating E9.5 PGCs), we think this developmental gap is the possible target for the improvement. Ohta et al. (EMBO J. 2017 Jul 3;36(13):1888-1907) reported the in vitro expansion of PGCLCs progressed their epigenetic reprogramming as in gonadal PGCs. Therefore, we assume that this extended culture of PGCLCs before the co-culture with gonadal somatic cells would bridge the developmental gap and relieve the epigenetic instability of oocytes generated by the *in vitro* culture system. We added this thought in Discussion of the manuscript (Lines 581-586).

Also, previous publications employed single follicle culture during IVG. This approach is based on the report that a whole rOvary culture during IVG resulted in limited development of follicles only at the edge of the rOvary (Extended Fig 3a in Hikabe et al, 2016). Our data proposes the intermediate condition (clusters of 4-10 follicles) between single follicles and whole rOvaries yield better follicle development during IVG (Fig EV2B). Although this improvement is not drastic, we believe our finding contributes to steady progress of the *in vitro* culture system.

3. During oocyte maturation, the author only focused on the transcriptional levels, while we know that transcription is suppressed during oocyte maturation, and the true regulation of its maturation occurs at the translational level. The author did not study the differences in oocyte differentiation from the perspectives of proteomics or translatomics.

Response: We thank the reviewer for providing the constructive suggestion. Our study aimed at monitoring possible changes in gene expression during IVD, as measured in GROs, as well as during IVG, as measured in FGOs (prior to their transcriptional shut-down), as the final output of the lengthy process. It has not been the purpose to study the impact of *in vitro* oogenesis on the process of mRNA translation during oocyte maturation corresponding to the transition from FGO to MII. We assume low sample proteomics would be technically challenging to identify the fraction between *in vivo* and *in vitro* samples, therefore we deemed the proteomics beyond our scope. Nevertheless, assessing translatability is an interesting avenue, therefore we would leave this analysis for a future investigation.

4. It would be important to include a comparison of the expression of transcription factors between in vivo and in vitro model systems.

Response: We thank the reviewer for this valuable suggestion. We agree that it would be interesting for readers to see the impact of *in vitro* oogenesis on expression of certain transcription factors important for transcriptional regulation during oogenesis. To address this point, we expanded our set of selected genes in Fig 6F by including transcription factors investigated previously in Hamazaki et al, 2021. We updated Fig6F and included Appendix Figure S1 to allow readers to assess impact of *in vitro* oogenesis on genes involved in epigenetic regulation as well as on expression of key transcription factors for oogenesis.

Minor comments
5. In line 388, the authors refer to the gene in vitro expressions referencing Figs 5C and D. However, these figures appear to only include in vivo data.
Response: We rephrased the sentence introducing the analysis to make it more transparent that the aim was to put altered transcriptional changes during *in vitro* development in the context of transcriptional dynamics occurring during *in vivo* development.

6. In line 389-393, author should clearly distinguish between 'all genes' and 'affected genes'. Are all genes shown in the in vivo data? I have trouble understanding the difference between the following sets - IVP@FGO+IVD@FGO+IVG@FGO.
Response: We edited the main text as well as the Methods section regarding computational analysis and selection of exonic regions for genes to make the source and the process of selection of transcripts more transparent. Indeed, the Fig 5D shows expression of all genes annotated in the Bioconductor annotation package TxDb.Mmusculus.UCSC.mm10.knownGene (v3.2.2) after selection of a random transcript isoform for each gene in order to generate a set of promoter regions. Affected genes are a subset of all genes with statistically significant (with cutoffs FDR ≤ 5% and $|\log2$ (Fold-change)$|$ ≥2) expression response to any step of *in vitro* development.
IVP@FGO, IVD@FGO and IVG@FGO are expression responses quantified as log2(fold-change) of each gene to steps (effects) of *in vitro* development at FGO stage. Concretely, these are coefficients in generalized linear model (GLM) which were fit for each gene aimed at explaining expression data in terms of presence/absence of each step (covariates in the GLM) of *in vitro* development applied to each oocyte cohorts investigated in this study (see Fig 5A and Methods). For simplicity, we do not include interactions between covariates into the model, therefore expression differences between FGO^{iPSC} and $FGO^{\textit{in vivo}}$ are modelled as linear combination of each effect, i.e. IVP@FGO + IVD@FGO + IVG@FGO (Fig 5A).

Dear Anton, dear Antoine,

Thank you for submitting your revised manuscript (EMBOJ-2023-113955R) to The EMBO Journal, as well as for your patience with our response at this time of the year. Your amended study was sent back to the referees for their re-evaluation, and we have received comments from one of them, which I enclose below. Please note that while referee #2 was not able to reassess your amended study at this time, we have editorially evaluated your response to the issues raised and found them to addressed satisfactorily.

As you will see, the other expert stated that the work has been substantially improved by the revisions and s/he is now broadly in favour of publication.

Thus, we are pleased to inform you that your manuscript has been accepted in principle for publication in The EMBO Journal.

We now need you to take care of a number of minor issues related to formatting and data presentation as detailed below, which should be addressed at re-submission.

Please contact me at any time if you have additional questions related to below points.

Thank you for giving us the chance to consider your manuscript for The EMBO Journal. I look forward to your final revision.

Again, please contact me at any time if you need any help or have further questions.

Best regards,

Daniel

Daniel Klimmeck PhD
Senior Editor
The EMBO Journal

****

Formatting changes required for the revised version of the manuscript:

>> Adjust the title of the 'Declaration of Interests' section to 'Disclosure and Competing Interests Statement'.

>> Author Contributions: Please remove the author contributions information from the manuscript text. Note that CRediT has replaced the traditional author contributions section as of now because it offers a systematic machine-readable author contributions format that allows for more effective research assessment. and use the free text boxes beneath each contributing author's name to add specific details on the author's contribution.
More information is available in our guide to authors.
https://www.embopress.org/page/journal/14602075/authorguide

>> Manuscript text composition - order:
>>>> Please remove Supporting Information details from the manuscript file.
>>>> Remove "Tables" and Table 1 legend from p. 51; Table 1 should be renamed to an EV Table and uploaded as Expanded View .
>>>> Figure legends should go after the References in the manuscript file.
>>>> Condense Expanded View Table EV1 to only one full page.

>> Appendix file: please amend the ToC on the first page by adding page numbers. The Reagents and Tools Table should be removed from the file and uploaded individually making sure to follow the specific Word/Excel format required as detailed in our Guide for Reagents tables; Dataset legends also need to be removed.

Change the nomenclature to Appendix Figure S1-S2... and Appendix Table S1-... and adjust the corresponding callouts in the text.

>> Dataset EV Legends: Dataset legends need to be removed from the Appendix file as each legend should be provided in each

Dataset Excel file as a separate sheet.

>> Data accessibility section: please remove the referee token and make sure privacy is released for the GEO dataset.

>> Funding information: please update the information provided in our online system. Currently missing: Novartis Research Foundation.

>>Enter a separate 'Statistical Analysis' section in the 'Material and Methods' part, detailing all algorithms and significance measures applied.

>> Consider additional changes and comments from our production team as indicated by the .doc file enclosed and leave changes in track mode.

Further information is available in our Guide For Authors: https://www.embopress.org/page/journal/14602075/authorguide

----

Referee #1:

The authors have addressed or adequately responded to our comments and suggestions. The revised manuscript represents an important progression for in vitro gametogenesis from both a technical standpoint and considering the biological insight, and we support its timely publication.

The authors addressed the remaining editorial issues.

2nd Revision - Editorial Decision 18th Sep 2023

Dear Dr Wutz, dear Dr Peters,

Thank you for submitting the revised version of your manuscript. I have now evaluated your amended manuscript and concluded that the remaining minor concerns have been sufficiently addressed.

Thus, I am pleased to inform you that your manuscript has been accepted for publication in the EMBO Journal.

Please note that it is EMBO Journal policy for the transcript of the editorial process (containing the arbitrator's report) to be published as an online supplement to each paper.

Also, in case you might NOT want the transparent process file published at all, you will also need to inform us via email immediately. More information is available here:
https://www.embopress.org/page/journal/14602075/authorguide#transparentprocess

----

Please note that in order to be able to start the production process, our publisher will need and contact you shortly regarding the page charge authorisation and licence to publish forms.

Authors of accepted peer-reviewed original research articles may choose to pay a fee in order for their published article to be made freely accessible to all online immediately upon publication. The EMBO Open fee is fixed at $6,540 USD / £5,310 GBP / €5,900 EUR (+ VAT where applicable).

We offer two licenses for Open Access papers, CC-BY and CC-BY-NC-ND.
For more information on these licenses, please visit:
http://creativecommons.org/licenses/by-nc-nd/3.0/deed.en_US

Should you be planning a Press Release on your article, please get in contact with embojournal@wiley.com as early as possible, in order to coordinate publication and release dates.

Notably, please be reminded that under the DEAL agreement of European scientific institutions with our publisher Wiley, you could be eligible for free publication of your article in the open access format. Please contact either the administration at your institution or Wiley (embojournal@wiley.com) to clarify further questions.

On a different note, I would like to alert you that EMBO Press is currently developing a new format for a video-synopsis of work published with us, which essentially is a short, author-generated film explaining the core findings in hand drawings, and, as we believe, can be very useful to increase visibility of the work. This has proven to offer a nice opportunity for exposure i.p. for the first author(s) of the study. Please see the following link for representative examples and their integration into the article web page:
https://www.embopress.org/video_synopses
https://www.embopress.org/doi/full/10.15252/embj.2019103932

Please let me know, should you be interested to engage in commissioning a similar video synopsis for your work. According operation instructions are available and intuitive.

Finally, we have noted that the submitted version of your article is also posted on the preprint platform bioRxiv. We would appreciate if you could alert bioRxiv on the acceptance of this manuscript at The EMBO Journal in order to allow for an update of the entry status. Thank you in advance!

If you have any questions, please do not hesitate to call or email the Editorial Office.

Thank you again for this contribution to The EMBO Journal and congratulations on a successful publication! Please consider us again in the future for your most exciting work.

Best regards,

Daniel Klimmeck

Daniel Klimmeck, PhD
Senior Editor
The EMBO Journal
EMBO
Postfach 1022-40
Meyerhofstrasse 1
D-69117 Heidelberg
contact@embojournal.org
Submit at: http://emboj.msubmit.net

t

EMBO Press Author Checklist

Corresponding Author Name: Luigi Naldini
Journal Submitted to: The EMBO Journal
Manuscript Number: EMBOJ-2023-114188

USEFUL LINKS FOR COMPLETING THIS FORM
The EMBO Journal - Author Guidelines
EMBO Reports - Author Guidelines
Molecular Systems Biology - Author Guidelines
EMBO Molecular Medicine - Author Guidelines

Reporting Checklist for Life Science Articles (updated January
This checklist is adapted from Materials Design Analysis Reporting (MDAR) Checklist for Authors. MDAR establishes a minimum set of requirements in transparent reporting in the life sciences (see Statement of Task: 10.31222/osf.io/9sm4x). Please follow the journal's guidelines in preparing your
Please note that a copy of this checklist will be published alongside your article.

Abridged guidelines for figures
1. Data
The data shown in figures should satisfy the following conditions:
- ➞ the data were obtained and processed according to the field's best practice and are presented to reflect the results of the experiments in an accurate and unbiased manner.
- ➞ ideally, figure panels should include only measurements that are directly comparable to each other and obtained with the same assay.
- ➞ plots include clearly labeled error bars for independent experiments and sample sizes. Unless justified, error bars should not be shown for technical
- ➞ if n<5, the individual data points from each experiment should be plotted. Any statistical test employed should be justified.
- ➞ Source Data should be included to report the data underlying figures according to the guidelines set out in the authorship guidelines on Data

2. Captions
Each figure caption should contain the following information, for each panel where they are relevant:
- ➞ a specification of the experimental system investigated (eg cell line, species name).
- ➞ the assay(s) and method(s) used to carry out the reported observations and measurements.
- ➞ an explicit mention of the biological and chemical entity(ies) that are being measured.
- ➞ an explicit mention of the biological and chemical entity(ies) that are altered/varied/perturbed in a controlled manner.
- ➞ the exact sample size (n) for each experimental group/condition, given as a number, not a range;
- ➞ a description of the sample collection allowing the reader to understand whether the samples represent technical or biological replicates (including how many animals, litters, cultures, etc.).
- ➞ a statement of how many times the experiment shown was independently replicated in the laboratory.
- ➞ definitions of statistical methods and measures:
  - common tests, such as t-test (please specify whether paired vs. unpaired), simple χ2 tests, Wilcoxon and Mann-Whitney tests, can be unambiguously identified by name only, but more complex techniques should be described in the methods section;
  - are tests one-sided or two-sided?
  - are there adjustments for multiple comparisons?
  - exact statistical test results, e.g., P values = x but not P values < x;
  - definition of 'center values' as median or average;
  - definition of error bars as s.d. or s.e.m.

Please complete ALL of the questions below.
Select "Not Applicable" only when the requested information is not relevant for your study.

Materials

Newly Created Materials	Information included in the manuscript?	In which section is the information available? (Reagents and Tools Table, Materials and Methods, Figures, Data Availability Section)
New materials and reagents need to be available; do any restrictions apply?	Yes	Material and methods/Extended View Content/Data Availability Section

Antibodies	Information included in the manuscript?	In which section is the information available? (Reagents and Tools Table, Materials and Methods, Figures, Data Availability Section)
For **antibodies** provide the following information:<br>- Commercial antibodies: RRID (if possible) or supplier name, catalogue number and or/clone number<br>- Non-commercial: RRID or citation	Yes	Expanded View Content

DNA and RNA sequences	Information included in the manuscript?	In which section is the information available? (Reagents and Tools Table, Materials and Methods, Figures, Data Availability Section)
**Short novel DNA or RNA including primers, probes**: provide the sequences.	Yes	Expanded View Content

Cell materials	Information included in the manuscript?	In which section is the information available? (Reagents and Tools Table, Materials and Methods, Figures, Data Availability Section)
**Cell lines:** Provide species information, strain. Provide accession number in repository **OR** supplier name, catalog number, clone number, and/**OR** RRID.	Yes	Expanded View Content
**Primary cultures:** Provide species, strain, sex of origin, genetic modification status.	Yes	Methods
Report if the cell lines were recently **authenticated** (e.g., by STR profiling) and tested for mycoplasma contamination.	Yes	Expanded View Content. Cell line was bought as described.

Experimental animals	Information included in the manuscript?	In which section is the information available? (Reagents and Tools Table, Materials and Methods, Figures, Data Availability Section)
**Laboratory animals or Model organisms:** Provide species, strain, sex, age, genetic modification status. Provide accession number in repository **OR** supplier name, catalog number, clone number, **OR** RRID.	Yes	Expanded View Content (ref to previous work)
**Animal observed in or captured from the field:** Provide species, sex, and age where possible**.**	Not Applicable	No animals were captured from the field
Please detail **housing and husbandry conditions**.	Yes	Expanded View Content

Plants and microbes	Information included in the manuscript?	In which section is the information available? (Reagents and Tools Table, Materials and Methods, Figures, Data Availability Section)
**Plants:** provide species and strain, ecotype and cultivar where relevant, unique accession number if available, and source (including location for collected wild specimens).	Not Applicable	No plants were used
**Microbes:** provide species and strain, unique accession number if available, and source.	Not Applicable	No microbes were used

Human research participants	Information included in the manuscript?	In which section is the information available? (Reagents and Tools Table, Materials and Methods, Figures, Data Availability Section)
If collected and within the bounds of privacy constraints report on age, sex and gender or ethnicity for all study participants.	Yes	Methods - Human subjects research

Core facilities	Information included in the manuscript?	In which section is the information available? (Reagents and Tools Table, Materials and Methods, Figures, Data Availability Section)
If your work benefited from core facilities, was their service mentioned in the acknowledgments section?	Yes	Aknowledgements

Design

Study protocol	Information included in the manuscript?	In which section is the information available? (Reagents and Tools Table, Materials and Methods, Figures, Data Availability Section)
If study protocol has been **pre-registered, provide DOI in the manuscript**. For clinical trials, provide the trial registration number **OR** cite DOI.	Not Applicable	Not a clinical study
Report the **clinical trial registration number** (at ClinicalTrials.gov or equivalent), where applicable.	Not Applicable	Not a clinical study

Laboratory protocol	Information included in the manuscript?	In which section is the information available? (Reagents and Tools Table, Materials and Methods, Figures, Data Availability Section)
Provide DOI OR other citation details if **external detailed step-by-step protocols** are available.	Yes	Expanded View Content > Methods > Nanopore sequencing

Experimental study design and statistics	Information included in the manuscript?	In which section is the information available? (Reagents and Tools Table, Materials and Methods, Figures, Data Availability Section)
Include a statement about **sample size** estimate even if no statistical methods were used.	Not Applicable	Not applicable
Were any steps taken to minimize the effects of subjective bias when allocating animals/samples to treatment (e.g. **randomization procedure**)? If yes, have they been described?	Not Applicable	Not applicable
Include a statement about **blinding** even if no blinding was done.	Not Applicable	Not applicable
Describe **inclusion/exclusion criteria** if samples or animals were excluded from the analysis. Were the criteria pre-established?<br><br>If sample or data points were omitted from analysis, report if this was due to attrition or intentional exclusion and provide justification.	Not Applicable	Not applicable
For every figure, are **statistical tests** justified as appropriate? Do the data meet the assumptions of the tests (e.g., normal distribution)? Describe any methods used to assess it. Is there an estimate of variation within each group of data? Is the variance similar between the groups that are being statistically compared?	Yes	Non parametrical tests were used throughout the manuscript

Sample definition and in-laboratory replication	Information included in the manuscript?	In which section is the information available? (Reagents and Tools Table, Materials and Methods, Figures, Data Availability Section)
In the figure legends: state number of times the experiment was **replicated** in laboratory.	Yes	Figure legends
In the figure legends: define whether data describe **technical or biological replicates**.	Yes	Figure legends

Ethics

Ethics	Information included in the manuscript?	In which section is the information available? (Reagents and Tools Table, Materials and Methods, Figures, Data Availability Section)
Studies involving **human participants**: State details of **authority granting ethics approval** (IRB or equivalent committee(s), provide reference number for approval.	Yes	Methods
Studies involving **human participants**: Include a statement confirming that **informed consent** was obtained from all subjects and that the experiments conformed to the principles set out in the WMA Declaration of Helsinki and the Department of Health and Human Services Belmont Report.	Yes	Methods
Studies involving **human participants: For publication of patient photos**, include a statement confirming that consent to publish was obtained.	Not Applicable	Not applicable
Studies involving experimental **animals**: State details of **authority granting ethics approval** (IRB or equivalent committee(s), provide reference number for approval. Include a statement of compliance with ethical regulations.	Yes	Expanded View Content
Studies involving **specimen and field samples:** State if relevant **permits** obtained, provide details of authority approving study; if none were required, explain why.	Not Applicable	Not applicable

Dual Use Research of Concern (DURC)	Information included in the manuscript?	In which section is the information available? (Reagents and Tools Table, Materials and Methods, Figures, Data Availability Section)
Could your study fall under dual use research restrictions? Please check biosecurity documents and list of **select agents and toxins** (CDC): https://www.selectagents.gov/sat/list.htm	Not Applicable	NO
If you used a select agent, is the security level of the lab appropriate and reported in the manuscript?	Not Applicable	
If a study is subject to dual use research of concern regulations, is the name of the **authority granting approval and reference number** for the regulatory approval provided in the manuscript?	Not Applicable	

Reporting

The MDAR framework recommends adoption of discipline-specific guidelines, established and endorsed through community initiatives. Journals have their own policy about requiring specific guidelines and recommendations to complement MDAR.

Adherence to community standards	Information included in the manuscript?	In which section is the information available? (Reagents and Tools Table, Materials and Methods, Figures, Data Availability Section)
State if relevant guidelines or checklists (e.g., **ICMJE, MIBBI, ARRIVE, PRISMA**) have been followed or provided.	Not Applicable	Not applicable
For **tumor marker prognostic studies,** we recommend that you follow the **REMARK** reporting guidelines (see link list at top right). See author guidelines, under 'Reporting Guidelines'. Please confirm you have followed these guidelines.	Not Applicable	Not applicable
For **phase II and III randomized controlled trials**, please refer to the **CONSORT** flow diagram (see link list at top right) and submit the CONSORT checklist (see link list at top right) with your submission. See author guidelines, under 'Reporting Guidelines'. Please confirm you have submitted this list.	Not Applicable	Not applicable

Data Availability

Data availability	Information included in the manuscript?	In which section is the information available? (Reagents and Tools Table, Materials and Methods, Figures, Data Availability Section)
Have **primary datasets** been deposited according to the journal's guidelines (see 'Data Deposition' section) and the respective accession numbers provided in the Data Availability Section?	Yes	Data availability section; zip files with source data uploaded with submission
Were **human clinical and genomic datasets** deposited in a public access-controlled repository in accordance to ethical obligations to the patients and to the applicable consent agreement?	Yes	
Are **computational models** that are central and integral to a study available without restrictions in a machine-readable form? Were the relevant accession numbers or links provided?	Not Applicable	Proprietary Bionano Access software and Oxford Nanopore MinKnow were used for data analysis
If publicly available data were reused, provide the respective **data citations in the reference list**.	Not Applicable	Not applicable
